# Base-Mediated Rearrangement of α-Dithioacetyl Propargylamines via Expansion of Dithioacetyl Ring: Synthesis of Medium-Sized *S,S*-Heterocycles

**DOI:** 10.1021/acs.orglett.3c01118

**Published:** 2023-05-26

**Authors:** Mert Dinc, Eda Ismailoglu, Zeynep Mert, Kerem Kaya, Melda Tayanc, Baris Yucel

**Affiliations:** Istanbul Technical University, Science Faculty, Department of Chemistry, Maslak, Istanbul 34469, Türkiye

## Abstract

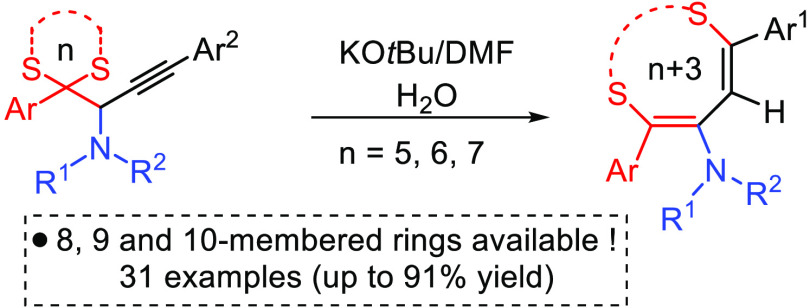

Base-mediated rearrangement of 1,3-dithianyl-substituted propargylamines in DMF via expansion of the dithiane ring has been reported. The rearrangement provided 9-membered amino-functionalized sulfur-containing heterocycles (dithionine derivatives) in good yields under mild conditions. Propargylamines bearing 5-membered 1,3-dithiolane and 7-membered 1,3-dithiepane rings rearranged in a similar manner yielding 8- and 10-membered *S,S*-heterocycles, respectively.

Sulfur-containing heterocycles are of particular interest in organic synthesis since they are widely present in pharmaceuticals, agrochemicals, natural products, and organic electronic materials.^1a,1b^ Although thiophene and 5- and 6-membered *S*-heterocycles are the most encountered units in biologically active compounds, medium-sized sulfur rings with extra heteroatoms and amino substituents are essential structural components of several US FDA-approved drugs, pharmaceutically relevant molecules, and natural compounds (Figure 1).^2a−2d^ On the other hand, construction of medium-sized rings via intramolecular cyclization reactions is more challenging due to unfavorable transannular interactions and torsional strains.^3a−3c^ Several methods are frequently applied for the synthesis of medium-size ring structures such as metal-catalyzed^4a,4b^ or -mediated cyclization reactions,^5^ ring-closing metathesis,^6^ radical cyclization,^7^ and ring-expansion reactions.^8a−8d^

**Figure 1:**
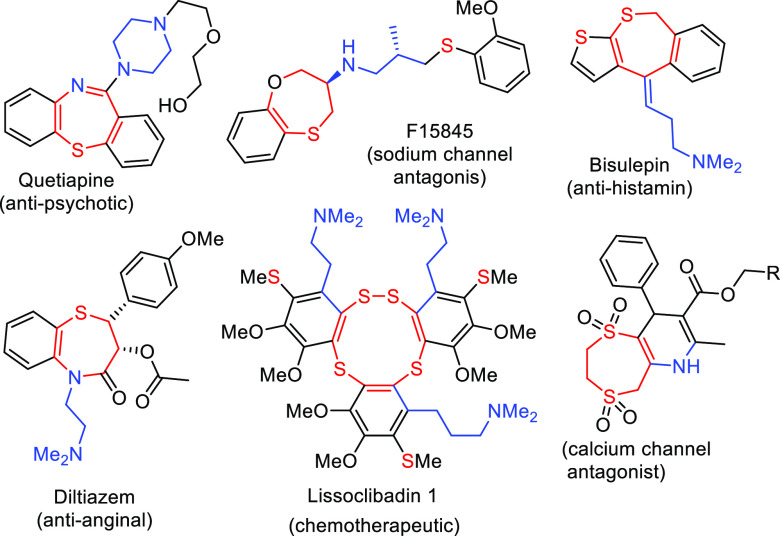
Examples of biologically active amino-functionalized sulfur-containing heterocycles.

1,3-Dithiane derivatives are valuable tools that have long been employed in organic synthesis as acyl anion equivalents and as a protecting group for carbonyl compounds. They are also valuable precursors for the synthesis of sulfur-containing acyclic compounds and heterocycles. 1,3-Dithianes have been converted into *S,S*-heterocycles of various sizes (between 7- and 11-membered), generally by following two different routes (Scheme 1). In the first one, dithiane derivatives bearing a leaving group on their side chain undergo ring expansion via initially formed bicyclic sulfonium ion and succeeding thionium intermediates. After elimination of a proton in these intermediates, the reaction affords, in general, simple *S,S*-heterocyclic products lacking functional groups as a mixture of stereoisomers.^9a−9c^ Depending on the presence of a nucleophile in the medium, the thionium ion is trapped to form the ring without a double bond.^10a,10b^

**Scheme 1:**
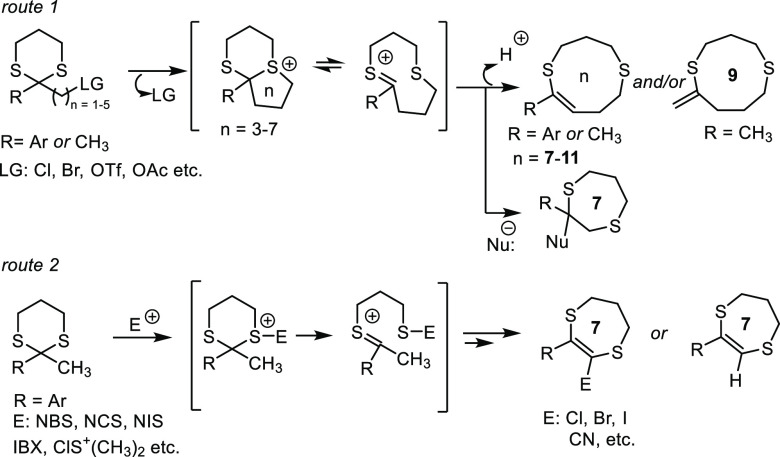
Different Routes for Expansion of the 1,3-Dithiane Ring

In the second route, 2-alkyl-2-aryl-1,3-dithianes undergo ring expansion with various electrophiles, resulting in 7-membered dithiepine derivatives through formation of a thionium intermediate.^11a,11b^ Functionally substituted dithiepines are accessible based on the amount and type of electrophiles used as well as the substituent on the aryl group. However, to the best of our knowledge, larger rings generated by this method were not reported.

Expansion of the 1,3-dithiane ring was rarely observed under catalytic conditions.^12a−12c^ Propargylic 1,3-dithianes have recently been converted via an Au-catalyzed reaction into 8-membered dithio-substituted cyclic allenes (eq 1, Scheme 2a) and dithiocine derivatives (eq 2, Scheme 2a) depending on the type of substituent at the 2 position of the dithiane ring. The reaction mechanism likely involves the formation of a Au–carbene intermediate followed by a 1,2-sulfur shift to produce the cyclic allene.^13^ We recently found that electrophilic iodocyclization of 1-(1,3-dithian-2-yl)propargylamines leads to 3-amino-4-iodothiophenes via iodide-induced ring fragmentation of bicyclic sulfonium ion formed as an intermediate product (eq 3, Scheme 2b).^14^ During our investigation, we noticed that under strongly basic conditions 1-(1,3-dithian-2-yl)propargylamines undergo rearrangement initiated by the abstraction of propagylic proton and followed by expansion of the dithiane ring (eq 4, Scheme 2b). After careful tuning of the reaction conditions, we were able to conduct this transformation using 0.5 equiv of KO*t*Bu in DMF to achieve different substituted amino functionalized 9-membered *S,S*-heterocycles in good yields.

**Scheme 2:**
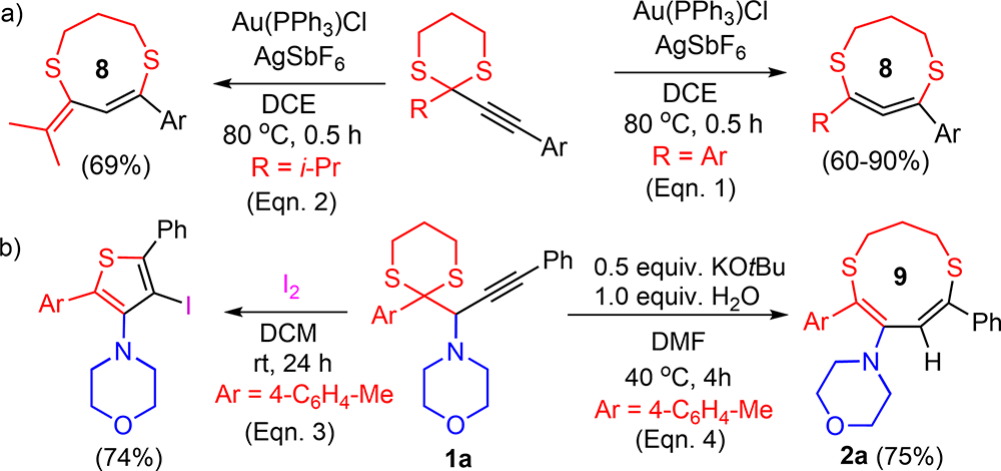
Rearrangement of Propargylic 1,3-Dithianes via Expansion of the Dithiane Ring

Initially, heating **1a** with 1.0 equiv of KO*t*Bu in DMF at 40 °C for 4 h furnished the product **2a** in 35% yield. We envisaged that addition of a proton source to the reaction mixture might improve the yield of the products. Thus, varied amounts of H_2_O/*t*BuOH were added to the reaction mixture at different temperatures. The best yield of the product **2a** (71–75%) was obtained with the addition of 1.0 equiv of H_2_O at 40 °C with 0.5 equiv of KO*t*Bu as base. Performing the reaction in DMSO as solvent furnished a 65% yield of **2a**, while use of THF, CH_3_CN, and CPME did not yield the product. Changing the solvent from DMF to DMA (dimethylacetamide) gave the product in a comparable 76% yield under the optimized conditions. Use of NaO*t*Bu and NaO*t*Am as base in the reaction in place of KO*t*Bu afforded the product **2a** in 70% and 69% yields, respectively, while use of KOH or KHMDS gave a lower yield of the product (Table S1).

Under the optimized conditions, the scope and limitations of the rearrangement reaction were tested with diversely substituted 1-(1,3-dithian-2-yl)propargylamines. The 1,3-dithianyl-substituted propargylamines were prepared by a Au-catalyzed coupling reaction of 1,3-dithiane-2-carbaldehydes, alkynes, and secondary amines described by us earlier^14^ (see Table S2).

As depicted in Scheme 3a, 1,3-dithianyl-substituted propargylamines produced the corresponding 9-membered *S,S-*heterocycles (dithionine derivatives) **2a**–**m** in good yields (46–84%), irrespective of the electronic properties of aryls on the dithiane ring and on the propargyl moiety. Heteroaryl-substituted propargylamines afforded the dithionines **2n** and **2o** in 77% and 59% yields, respectively. Propargylamines having amine groups other than morpholine proceeded well under the optimized conditions and gave the corresponding 9-membered rings **2p**–**u** in good yields (52–77%). Considering the formation of KOH by combination of KO*t*Bu and water in the reaction medium, we tested 1.0 mmol scale reactions of propargylamines (**1a**, **1e**, **1h**, and **1o**) with 0.5 mmol of KOH (80%, w/w) under the standard conditions. The reactions produced the corresponding heterocycles **2a**, **2e**, **2h**, and **2o** in good yields, respectively.

**Scheme 3:**
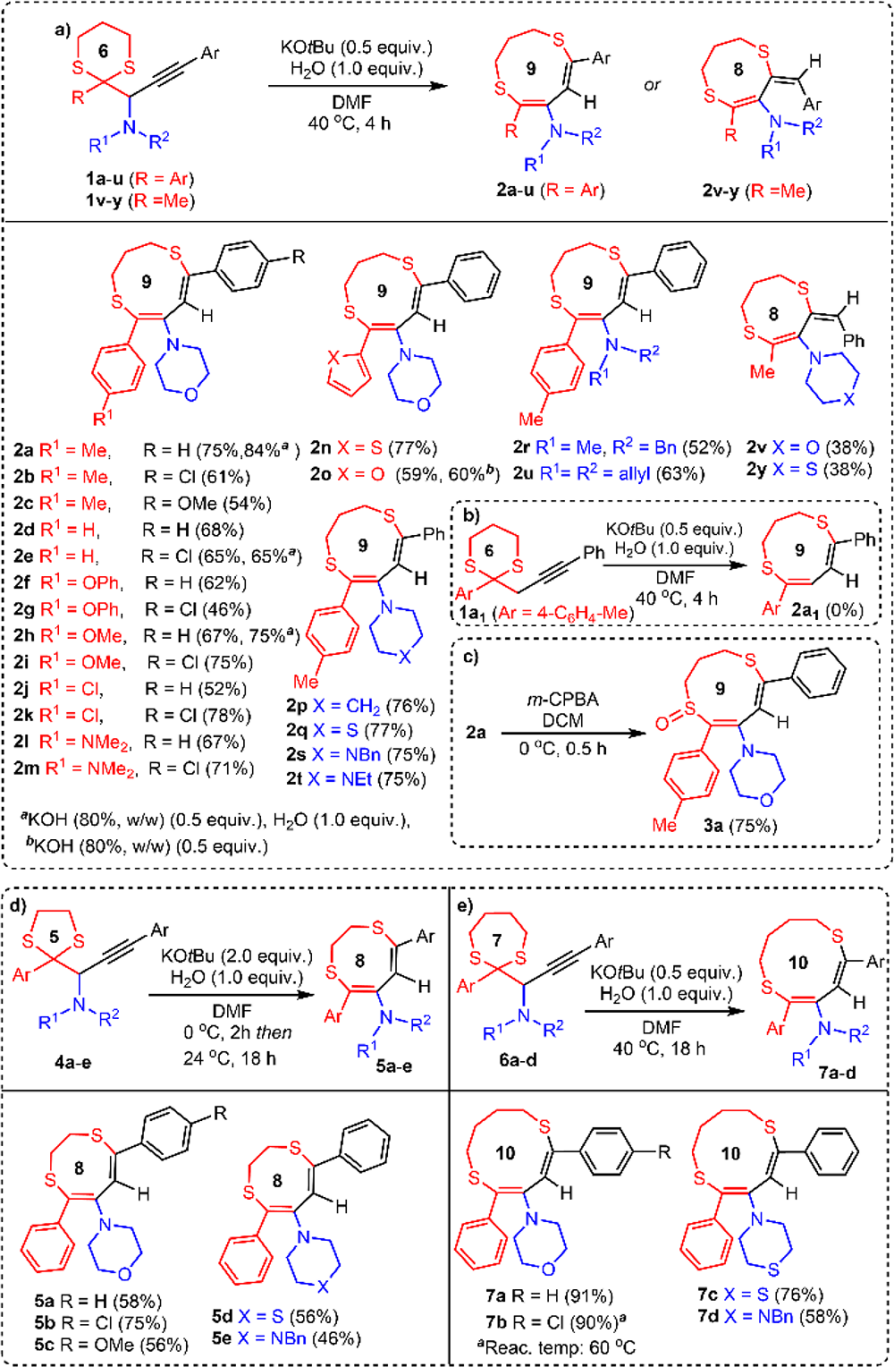
Substrate Scope for 8-, 9-, and 10-Membered Rings

We performed the reaction of **1o** without water to see the reaction performance in the presence of KOH. The reaction gave **2o** in 60% yield, similar to the reaction performed with KO*t*Bu. Replacing the aryl group on dithiane with a methyl group resulted in the formation of 8-membered rings **2v** and **2y** with an exocyclic double bond in 38% yield. The formation of a 9-membered ring structure was not observed in these reactions. To understand the effect of amine group in the reaction, we synthesized a propargyl substituted dithiane derivative **1a**_**1**_ without an amine group. Under the standard conditions, this substrate did not yield the 9-membered *S,S*-heterocycle **2a**_**1**_ (Scheme 3b).

Considering the presence of sulfoxide and sulfone moieties in bioactive molecules, oxidation of **2a** to the sulfoxide was attempted (Scheme 3c). Reaction of **2a** with 2.0 equiv of *m*-CPBA provided the sulfoxide **3a** in 75% yield. However, use of 1.0 equiv of *m*-CPBA was insufficient for the consumption of **2a**, while using more than 2.0 equiv of *m*-CPBA resulted in a mixture of several unidentifiable compounds. The structure of sulfoxide **3a** was unequivocally established by single-crystal X-ray structure analysis.

Reactants **4a**–**e** and **6a**–**d** containing the 5- and 7-membered dithiolane and dithiepane rings furnished the rearranged products **5a**–**e** and **7a**–**d**, respectively (Scheme 3d,e). Fine tuning of the reaction conditions (see Table S7) was required for the synthesis of 8-membered dithiocine derivatives **5a**–**e** from 1,3-dithiolanyl-substituted propargylamines **4a**–**e**. The best yields were obtained when a solution of KO*t*Bu (2.0 equiv) in DMF was slowly added to the solution of propargylamine at 0 °C. This protocol perhaps minimizes the dithiolane ring fragmentation observed in the presence of strong bases.^15a,15b^

Since the reaction gave poor yields when performed without water, we conducted deuterium-labeling experiments to gain insight into the mechanism of the rearrangement reaction. Under the standard conditions, the reaction of **1a** yielded dithionine **D-2a** with 50% deuteration of the methine group when D_2_O was used as a proton source (Scheme 4a). Similarly, 40% D was incorporated into the methine carbon of exocylic double bond in 8-membered ring **D**-**2v** (Scheme 4b).

**Scheme 4:**
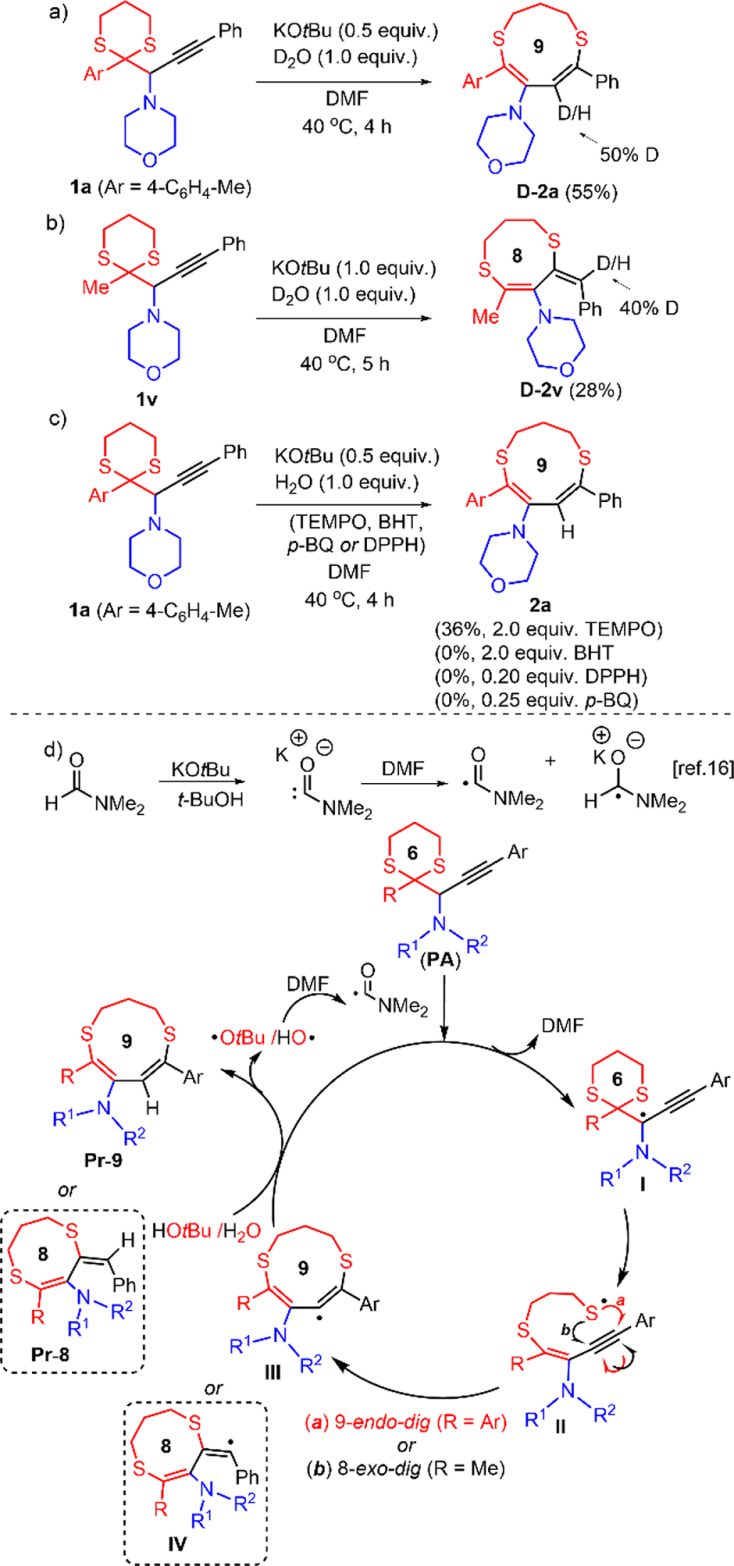
Control Experiments and Plausible Mechanistic Pathway

Recently, several reactions, involving the use of strong bases in DMF, DMSO, and DMAc solvents, were proposed to proceed via an electron-transfer mechanism.^16−18^ In these reactions, deprotonation of the solvent produces the corresponding anion which was transformed to the carbamoyl radical (from DMF), dimsyl radical (from DMSO), or dimethylcarbamoyl radical (from DMAc) by a subsequent single-electron transfer to the solvent molecule.^16^ Furthermore, electron transferred from the carbamoyl, dimsyl, and dimethylcarbamoyl anion to various species such as aryl iodides, aryl methyl sulfones, and benzil derivatives had resulted in the formation of the corresponding radicals.^17^ Sliwka et al. showed that the formation of long-lived-radical species by the addition of small quantities of strong bases to DMF and DMSO at room temperature.^18^

Since the KO*t*Bu-DMF system was closely linked to the electron-transfer process, we carried out additional control experiments in the presence of radical scavengers (Scheme 4c). The reaction of **1a** with 2.0 equiv of TEMPO under the standard conditions produced **2a** in 36% yield, which was lower than the optimum yield (75%) of the reaction. On the other hand, the formation of **2a** was completely prevented with the addition of 2.0 equiv of BHT (2,6-di-*tert*-butyl-4-methylphenol) and DPPH (2,2-diphenyl-1-picrylhydrazyl radical). The cyclization did not occur in the presence of BHT since it rapidly reacted with KO*t*Bu to form potassium phenoxide. We then tested different amounts of the radical scavengers and *p*-BQ (*p*-benzoquinone) in the reaction of **1a** with 0.5 and 1.0 equiv of KO*t*Bu under the standard conditions (see Table S8). The reaction was completely suppressed in the presence of 0.20 equiv of DPPH and 0.25 equiv of *p*-BQ, indicative of the rearrangement following a radical pathway.

Based on the experimental results and the literature precedence,^16^ we propose a plausible reaction pathway as depicted in Scheme 4d.^19^ The first step involves the deprotonation of DMF by KO*t*Bu, which results in the carbamoyl anion. This anion then likely produces a carbamoyl radical and a radical anion. The carbamoyl radical abstracts the propargylic hydrogen leading to formation of intermediate **I**. The dissociation of the dithiane ring in intermediate **I** results in sulfur-centered radical intermediate **II**, which undergoes regioselective *9-endo-dig* radical cyclization to generate a 9-membered heterocylic intermediate **III**. The intermediate **III** abstracts hydrogen either from H_2_O or from the other potential proton source such as *t*-BuOH to provide the 9-membered ring product **Pr**-**9**. When the R group is methyl, the intermediate **II** undergoes *8-exo-dig* cyclization, generating intermediate **IV**, leading to the formation of the 8-membered ring with an exocyclic double bond (**Pr**-**8**). Hydroxyl radical and possible *tert*-butoxy radical may regenerate the carbamoyl radical by removing hydrogen from DMF.

In summary, a new method based on KO*t*Bu-DMF-promoted rearrangement of dithioacetyl-substituted propargylamines under mild conditions was developed. The rearrangement reaction involves the expansion of the dithioacetal ring and proceeds via an exclusive *endo-dig* radical cyclization process to afford amino-functionalized *S,S*-heterocycles of various ring size.

## Data Availability

The data underlying this study are available in the published article and its online Supporting Information.

## Supporting Information Available

All experimental procedures and NMR, HRMS spectra data, X-ray crystallographic analysis for compounds **2a**–**7a** and **7b**, and copies of ^1^H NMR and ^13^C NMR, ^13^C-APT NMR spectra (PDF)
FAIR data, including the primary NMR FID files, for compounds **1′a**–**i**, **4′a**, **5′a**, **1aa**–**ad**, **1a**–**y**, **2a**–**y**, **3a**, **4a**–**e**, **5a**–**e**, **6a**–**d**, **7a**–**d**, **D**-**2a**, **D**-**2v** (ZIP)
FAIR data, including the primary NMR FID files, for compounds **APT_01****1′a**–**i**, **4′a**, **5′a**, **1aa**–**ad**, **1a**–**y**, **2a**–**y**, **3a**, **4a**–**e**, **5a**–**e**, **6a**–**d**, **7a**–**d**, **D**-**2a**, **D**-**2v** (ZIP)
FAIR data, including the primary NMR FID files, for compounds **CARBON_01****1′a**–**i**, **4′a**, **5′a**, **1aa**–**ad**, **1a**–**y**, **2a**–**y**, **3a**, **4a**–**e**, **5a**–**e**, **6a**–**d**, **7a**–**d**, **D**-**2a**, **D**-**2v** (ZIP)
