# Supplementary material for: Base-Mediated Rearrangement of α-Dithioacetyl Propargylamines via Expansion of Dithioacetyl Ring: Synthesis of Medium-Sized S,S-Heterocycles
Source: Org Lett. 2023 May 26;25(22):4028–32. doi: 10.1021/acs.orglett.3c01118 (PMC10262264; doi:10.1021/acs.orglett.3c01118)

SUPPORTING INFORMATION

Base Mediated Rearrangement of α-Dithioacetyl Propargylamines via Expansion of Dithioacetyl Ring: Synthesis of Medium-sized *S,S*-heterocycles

Mert Dinc, Eda Ismailoğlu, Zeynep Mert, Kerem Kaya, Melda Tayanc, Baris Yucel*

Istanbul Technical University, Science Faculty, Department of Chemistry, Maslak 34469, Istanbul, Türkiye.

TABLE OF CONTENTS

1. General Methods ……………………………………………………………………………………..S2

2. Optimization of reaction conditions for base mediated rearrangement of 1-(1,3-dithian-2-yl)propargylamines ……......S3

3. Synthesis of 1,3-dithiane-2-carbaldehyde derivatives (**1'a**–**i**)……………………..S4

4. Synthesis of 1-(1,3-dithian-2-yl)propargylamines (**1a**–**y** and **1aa**–**ag**)…... ……………….S6

5. Synthesis of 2-(3-phenylprop-2-yn-1-yl)-2-(*p*-tolyl)-1,3-dithiane (**1a$_1$**)……………….S11

6. Synthesis of 2-phenyl-1,3-dithiolane-2-carbaldehyde (**4'a**) and 2-phenyl-1,3-dithiepan-2-carbadehyde (**5'a**)………..S12

7. Synthesis of 1-(1,3-dithiolan-2-yl)propargylamines (**4a**–**e**)…………..S14

8. Synthesis of 1-(1,3-dithiepan-2-yl)propargylamines (**6a**–**d**) ……………. S18

9. Synthesis of 9-membered *S,S*-heterocyles (**2a**–**u**) via rearrangement of 1-(1,3-dithian-2-yl)propargylamines (**1a**–**u**)……………S21

10. Synthesis of 8-membered *S,S*-heterocyles (**2v**–**y**) via rearrangement of 1-(1,3-dithian-2-yl)propargylamines (**1v**–**y**) ……………… S35

11. Synthesis of 8-membered *S,S*-heterocyles (**5a**–**e**) via rearrangement of 1-(1,3-dithiolan-2-yl)propargylamines (**4a**–**e**)……………S37

12. Synthesis of 10-membered *S,S*-heterocyles (**7a**–**d**) via rearrangement of 1-(1,3-dithiepan-2-yl)propargylamines (**6a**–**d**)……………...S41

13. Oxidation of 9-membered ring **2a**, synthesis of sulfoxide **3a**…………S44

14. Mechanistic Studies …………………..S45

15. Proposed anionic mechanism……………......S52

14. X-ray Crystallography Data for compound **2a**–**7a**, **2v** and **7b**……………..S52

15. References……………..S61

16. NMR Spectra of compounds……………..S62

1. General Methods. All reagents were used as purchased from commercial suppliers without further purification unless otherwise indicated. Air- and moisture-sensitive solutions were handled under nitrogen and transferred via syringe. Tetrahydrofuran (THF) was freshly distilled from sodium/benzophenone ketyl. Anhydrous methanol was purchased from Merck and stored in glovebox. Anhydrous dimethyl sulfoxide (DMSO) and *N*-dimethylformamide (DMF) were purchased from Acros-Organics and used without further purification. All solvents used (MeOH, DMSO and DMF) were stored over activated molecular sieves (3 or 4 Å). Molecular sieves were pre-dried at 300 ^oC for 24 h immediately before use. Solvents for column chromatography, ethyl acetate and hexanes were distilled in a rotary evaporator. TLC was performed with Merck TLC Silicagel60 F_{254} plates and detection was under UV light at 254 nm. Chromatographic separations were performed with Merck Silica 60 (200–400 or 70–230 mesh). NMR spectra were recorded with a Varian Inova 500 (500 MHz for ^1H and 125 MHz for ^{13}C NMR) instruments. Chemical shifts δ were given in ppm relative to residual peaks of deuterated solvents and coupling constants, *J*, were given in Hertz. The following abbreviations are used to describe spin multiplicities in ^1H NMR spectra: s = singlet; bs = broad singlet; d = doublet; t = triplet; q = quartet; dd = doublet of doublets; m = multiplets. Multiplicities in ^{13}C NMR spectra were determined by DEPT (Distortionless Enhancement by Polarization Transfer) or APT (Attached Proton Test) measurements. High resolution mass spectra (HRMS) were obtained on a Waters Synapt Q-TOF-MS spectrometer.

2. Optimization of reaction conditions for base mediated rearrangement of 1-(1,3-dithian-2-yl)propargylamines

Table S1. Optimization of reaction conditions for product 2a

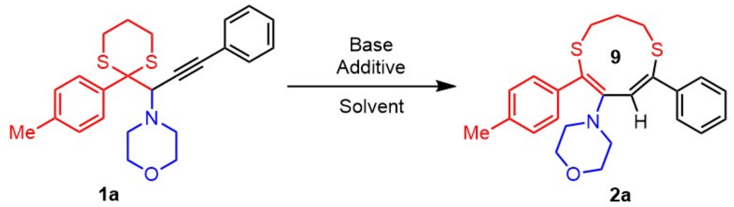

entry	solvent	base (equiv.)	additive (equiv.)	temp./time (°C / h)	Yield **2a** (%)
1	DMF	KO*t*Bu (1.0)	-	40 °C/4 h	35%
2	DMF	KO*t*Bu (0.5)	-	40 °C/4 h	52%
3	DMF	KO*t*Bu (1.0)	H_2O (1.0)	40 °C/4 h	64%
**4**[a]	**DMF**	**KO*t*Bu (0.5)**	**H_2O (1.0)**	**40 °C/4 h**	**71%**
**5**[b]	**DMF**	**KO*t*Bu (0.5)**	**H_2O (1.0)**	**40 °C/4 h**	**70%**
**6**[c]	**DMF**	**KO*t*Bu (0.5)**	**H_2O (1.0)**	**40 °C/4 h**	**75%**
7	DMF	KO*t*Bu (0.5)	H_2O (0.5)	40 °C/4 h	67%
8	DMF	KO*t*Bu (0.5)	H_2O (2.0)	40 °C/4 h	56%
9	DMF	KO*t*Bu (0.25)	H_2O (1.0)	40 °C/8 h	*trace*
10	DMF	KO*t*Bu (0.5)	H_2O (1.0)	40 °C/6 h	61%
11	DMF	KO*t*Bu (0.5)	H_2O (1.0)	24 °C/12 h	59%
12	DMF	KO*t*Bu (0.5)	H_2O (1.0)	60 °C/2 h	57%
13	DMF	KO*t*Bu (0.5)	*t*-BuOH (1.0)	40 °C/4 h	57%
14	DMF	KO*t*Bu (0.5)	*t*-BuOH (2.0)	40 °C/4 h	60%
15	DMF	KO*t*Bu (0.5)	*t*-BuOH (2.0)	60 °C/4 h	60%
16	DMSO	KO*t*Bu (0.5)	H_2O (1.0)	40 °C/5 h	58%
17	DMSO	KO*t*Bu (0.5)	*t*-BuOH (1.0)	40 °C/5 h	47%
18[d]	DMF/DMSO	KO*t*Bu (0.5)	H_2O (1.0)	40 °C/4 h	65%
19[e]	DMF/THF	KO*t*Bu (0.5)	H_2O (1.0)	40 °C/4 h	66%
20[f]	THF/DMF	KO*t*Bu (0.5)	H_2O (1.0)	40 °C/4 h	-
21	ACN	KO*t*Bu (0.5)	H_2O (1.0)	40 °C/4 h	-
22	CPME	KO*t*Bu (0.5)	H_2O (1.0)	40 °C/4 h	-
23	DMAc	KO*t*Bu (0.5)	H_2O (1.0)	40 °C/4 h	76%
24	DMF	KOH (0.5)	H_2O (1.0)	40 °C/4 h	55%
25	DMF	KOH (0.5)	*t*-BuOH (2.0)	40 °C/4 h	60%
26	DMF	KHMDS (0.5)	H_2O (1.0)	40 °C/4 h	67%
27	DMF	KHMDS (0.5)	-	40 °C/4 h	42%
28	DMF	Cs_2CO_3 (0.5)	-	40 °C/4 h	-
29	DMF	NaO*t*Bu (0.5)	H_2O (1.0)	40 °C/4 h	70%
30	DMF	NaO*t*Am (0.5)	H_2O (1.0)	40 °C/4 h	69%
31	DMF	-	H_2O (1.0)	40 °C/4 h	-

[a]0.25 mmol **1a**; [b]0.50 mmol **1a** [c]1.00 mmol **1a**; [d]5/1 (v/v) DMF/DMSO; [e]10/1 (v/v) DMF/THF; [f]5/1 (v/v) THF/DMF

3. Synthesis of 1,3-dithiane-2-carbaldehyde derivatives (1'a–i)

1,3-Dithiane-2-carbaldehydes were synthesized according to the published procedure (General Procedure B) given below. For NMR data and spectra of compounds **1'a**, **b** and **1'd–i** please see: ref. S4, (*Eur. J. Org. Chem.* 2021, 29, 4107-4124).[S1]

Scheme S1. Synthesis of 1,3-dithiane-2-carbaldehyde derivatives (**1'a–i**)[S2]

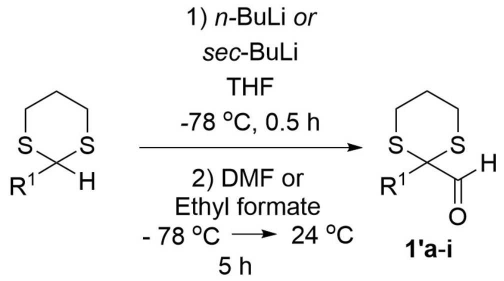

1'a: R^1 = 4-C_6H_4-Me (%74)
1'b: R^1 = Ph (%67)
1'c: R^1 = 4-C_6H_4-OPh (%70)
1'd: R^1 = 4-C_6H_4-OMe (%89)
1'e: R^1 = 4-C_6H_4-Cl (%51)
1'f: R^1 = 4-C_6H_4-NMe_2 (%81)
1'g: R^1 = 2-thienyl (%52)
1'h: R^1 = 2-furyl (%35)
1'i: R^1 = Me (%77)
1'j: R^1 = 4-C_6H_4-F (%0)
1'k: R^1 = 4-C_6H_4-CF_3 (%0)
1'l: R^1 = 2-C_6H_4-Me (%0)

3.1. General Procedure A: To a solution of 1,3-dithiane derivative (1.0 equiv.) in THF, cooled to −78 ºC under nitrogen atmosphere, was added dropwise a solution of *n*-butyllithium (1.2 equiv.) or *sec*-butyllithium (1.2 equiv.). The solution was stirred for 30 min. at −78 ºC and then ethyl formate (5.0 equiv.) or DMF (5.0 equiv.) was added dropwise at this temperature. The resulting mixture was slowly allowed to warm to room temperature and stirred for 5 h. The reaction mixture was quenched with water and extracted with ether. The organic phase was dried over $MgSO_4$, filtrated and the solvent was removed in a rotatory evaporator. The remaining residue was loaded onto a silica gel column and purified by flash chromatography.

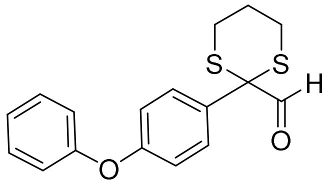

2-(4-Phenoxyphenyl)-1,3-dithiane-2-carbaldehyde (1'c): According to general procedure A, to a solution of 2-(4-phenoxyphenyl)-1,3-dithiane (4.5 mmol, 1.3 g) in THF (40 mL) at −78 °C under nitrogen, *n*-BuLi (2.1 mL of a 2.5 M of hexane solution, 5.4 mmol) was added. The solution was stirred for 30 min. at −78 °C and then ethyl formate (22.5 mmol, 1.6 mL) was added. The resulting mixture was stirred for 5 h and the crude material was obtained as described in the general procedure and purified by flash chromatography on silica gel using hexanes as eluent to yield the product **1'c** (0.98 g, 70%) as a white solid. ^{1}H NMR (500 MHz, $CDCl_3$): δ 9.14 (s, 1H), 7.42–7.45 (m, 2H), 7.34–7.38 (m, 2H), 7.15 (t, J = 7.4 Hz, 1H), 7.00–7.04 (m, 4H), 3.12–3.19 (m, 2H), 2.71 (dt, J = 3.3, 13.5 Hz, 2H), 2.11–2.17 (m, 1H), 1.84–1.93 (m, 1H) ppm; ^{13}C-APT NMR (125 MHz, $CDCl_3$): δ 186.6, 158.5, 156.2, 129.9, 129.2, 124.0, 119.6, 118.8, 61.1, 28.1, 23.7 ppm; HRMS [TOF MS ES+]: m/z $[M + H]^+$ calcd. for $C_{17}H_{16}O_2S_2$ 317.0670, found 317.0680 (3.2 ppm).

4. Synthesis of 1-(1,3-dithian-2-yl)propargylamines (1a-y and 1aa–ag)

1-(1,3-Dithian-2-yl)propargylamines were synthesized according to the published procedure (General Procedure B) given below. For NMR data and spectra of compounds **1a–d**, **1h–t** and **1v, 1aa–ac** please see: ref. S1, (*Eur. J. Org. Chem.* 2021, 29, 4107-4124).

Table S2. Substrate scope of 1-(1,3-dithian-2-yl)propargylamines (**1a-y** and **1aa–ag**)

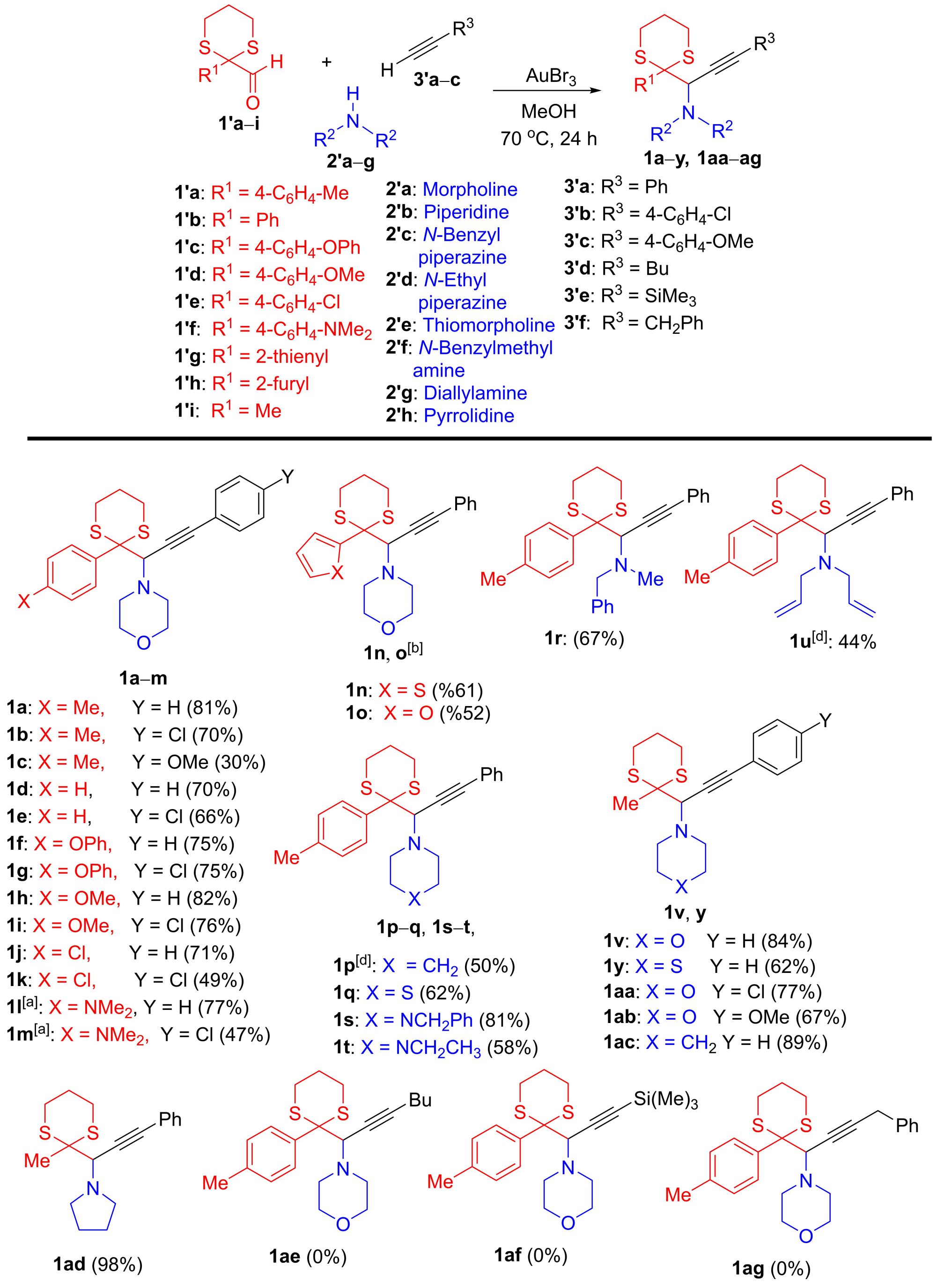

[a]: 80 °C, 24 h, [b]: 60 °C, 30 h, [c]: 80 °C, 24 h, [d]: 50 °C, 72 h

4.1. General Procedure B: An oven-dried 15 mL screw-cap reaction vial equipped with a stirring bar was charged with 1,3-dithiane-2-carbaldehyde derivative (1.0 mmol, 1.0 equiv.), secondary amine (2.0 mmol, 2.0 equiv.), and alkyne (3.0 mmol, 3.0 equiv.) and then the vial was brought into a glovebox. The reaction vial was charged with $AuBr_3$ (0.1 mmol), activated molecular sieves (300 mg, 3 Å) and anhydrous MeOH (0.5 mL). The vial was tightly closed, wrapped with a strip of Parafilm, and taken out of the glovebox. After the reaction mixture was stirred for the given time at the stated temperature in an oil bath, the resulting mixture was filtered through a small pad of Celite. The pad was washed with CH_2Cl_2 and then the obtained organic solution was mixed with silica gel. The solvent was evaporated in a rotatory evaporator. The remaining solid residue was loaded onto a silica gel column and purified by flash chromatography.

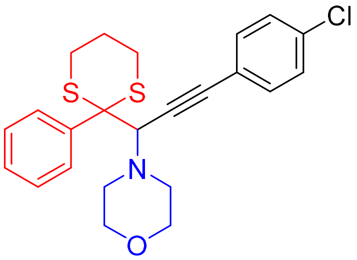

4-(3-(4-Chlorophenyl)-1-(2-phenyl-1,3-dithian-2-yl)prop-2-yn-1-yl)morpholine (1e): The reaction was performed following General Procedure B with 2-phenyl-1,3-dithiane-2-carbaldehyde (**1'b**) (1.0 mmol, 224 mg), morpholine (2.0 mmol, 174 mg) and 1-chloro-4-ethynylbenzene (3.0 mmol, 410 mg). In glovebox, the reaction vial was charged with $AuBr_3$ (0.1 mmol, 43.6 mg), activated molecular sieves (300 mg, 3 Å) and anhydrous MeOH (0.5 mL). The reaction was stirred for 24 h at 70 °C in an oil bath. The workup was performed following the general procedure. The crude material was purified by flash chromatography on silica gel using 20:1 hexanes/ethyl acetate as eluent to yield the product **1e** (280 mg, 66%) as a yellowish-white solid. ^{1}H NMR (500 MHz, $CDCl_3$): δ 8.15–8.18 (m, 2H), 7.34–7.40 (m, 4H), 7.26–7.30 (m, 3H), 4.05 (s, 1H), 3.59–3.67 (m, 4H), 2.68–2.74 (m, 2H), 2.56–2.66 (m, 2H), 2.40–2.48 [m, 2.40 (bs), 4H], 1.84–1.96 (m, 2H) ppm; ^{13}C-APT NMR (125 MHz, $CDCl_3$): δ 138.2, 134.3, 132.9, 130.7, 128.6, 128.0, 127.4, 121.2, 89.3, 83.1, 69.2, 66.9, 64.8, 64.8, 52.3, 27.8, 27.2, 25.4 ppm; HRMS [TOF MS ES+]: m/z $[M + H]^+$ calcd. for $C_{23}H_{25}NOS_2Cl$ 430.1066, found 430.1066 (0.0 ppm).

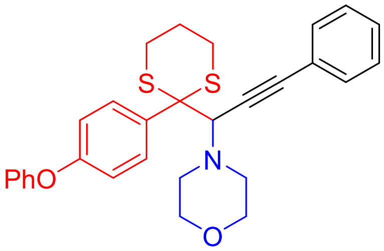

4-(1-(2-(4-phenoxyphenyl)-1,3-dithian-2-yl)-3-phenylprop-2-yn-1-yl)morpholine (1f): The reaction was performed following General Procedure B with 2-(4-phenoxyphenyl)-1,3-dithiane-2-carbaldehyde (**1'c**) (1.0 mmol, 316 mg), morpholine (2.0 mmol, 174 mg) and phenyl acetylene (3.0 mmol, 306 mg). In glovebox, the reaction vial was charged with $AuBr_3$ (0.1 mmol, 43.6 mg), activated molecular sieves (300 mg, 3 Å) and anhydrous MeOH (0.5 mL). The reaction was stirred for 24 h at 70 °C in an oil bath. The workup was performed following the general procedure. The crude material was purified by flash chromatography on silica gel using 20:1 hexanes/ethyl acetate as eluent to yield the product **1f** (366 mg, 75%) as a yellowish oil. ^{1}H NMR (500 MHz, $CDCl_3$): δ 8.11–8.14 (m, 2H), 7.41–7.45 (m, 2H), 7.33–7.37 (m, 2H), 7.28–7.32 (m, 3H), 7.12-7.14 (m, 1H), 7.03–7.05 (m, 2H), 6.98–7.01 (m, 2H), 4.07 (s, 1H), 3.61–3.68 (m, 4H), 2.63–2.77 (m, 4H), 2.49–2.53 (m, 2H), 2.45 (bs, 2H), 1.96–2.01 (m, 1H), 1.85–1.94 (m, 1H) ppm; ^{13}C-APT NMR (125 MHz, $CDCl_3$): δ 156.8, 156.7, 132.8, 132.4, 131.7, 129.8, 128.3, 128.3, 123.5, 122.8, 119.2, 117.7, 90.5, 82.0, 69.3, 67.1, 64.4, 27.9, 27.3, 25.5 ppm, [one CH_2 peak was not observed or co-incident]; HRMS [TOF MS ES+]: m/z $[M + H]^+$ calcd. for $C_{29}H_{30}NO_2S_2$ 488.1718, found 488.1718 (0.0 ppm).

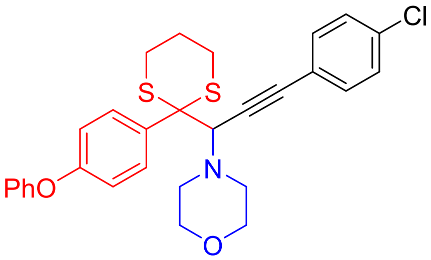

4-(3-(4-Chlorophenyl)-1-(2-(4-phenoxyphenyl)-1,3-dithian-2-yl)prop-2-yn-1-yl)morpholine (1g): The reaction was performed following General Procedure B with 2-(4-phenoxyphenyl)-1,3-dithiane-2-carbaldehyde (**1'c**) (1.0 mmol, 316 mg), morpholine (2.0 mmol, 174 mg) and 1-chloro-4-ethynylbenzene (3.0 mmol, 410 mg). In glovebox, the reaction vial was charged with $AuBr_3$ (0.1 mmol, 43.6 mg), activated molecular sieves (300 mg, 3 Å) and anhydrous MeOH (0.5 mL). The reaction was stirred for 24 h at 70 °C in an oil bath. The workup was performed following the general procedure. The crude material was purified by flash chromatography on silica gel using 20:1 hexanes/ethyl acetate as eluent to yield the product **1g** (391 mg, 75%) as a yellowish oil. ^{1}H NMR (500 MHz, $CDCl_3$): δ 8.08–8.11 (m, 2H), 7.34–7.38 (m, 4H), 7.26–7.29 (m, 2H), 7.14 (t, J= 7.4 Hz,

1H), 7.02–7.06 (m, 2H), 6.97–7.00 (m, 2H), 4.05 (s, 1H), 3.60–3.68 (m, 4H), 2.63–2.77 (m, 4H), 2.44–2.51 (m, 4H), 1.96–2.01 (m, 1H), 1.84–1.92 (m, 1H) ppm; ^{13}C-APT NMR (125 MHz, $CDCl_3$): δ 156.9, 156.7, 134.4, 132.7, 132.7, 132.3, 129.8, 128.6, 123.6, 121.2, 119.2, 117.7, 89.4, 83.2, 69.3, 67.0, 64.3, 27.9, 27.3, 25.5 ppm, [one CH_2 peak was not observed or co-incident]; HRMS [TOF MS ES+]: m/z $[M + H]^+$ calcd. for $C_{29}H_{29}NO_2S_2Cl$ 522.1328, found 522.1328 (0.0 ppm).

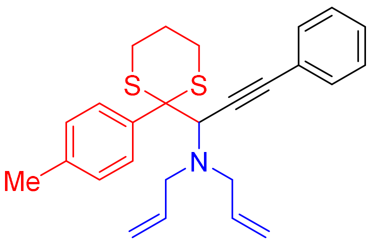

***N*-Allyl-*N*-(3-phenyl-1-(2-(*p*-tolyl)-1,3-dithian-2-yl)prop-2-yn-1-yl)prop-2-en-1-amine (1u):** The reaction was performed following General Procedure B with 2-*p*-tolyl-1,3-dithiane-2-carbaldehyde (**1'a**) (1.0 mmol, 238 mg), diallylamine (2.0 mmol, 194 mg) and phenyl acetylene (3.0 mmol, 306 mg). In glovebox, the reaction vial was charged with $AuBr_3$ (0.1 mmol, 43.6 mg), activated molecular sieves (300 mg, 3 Å) and anhydrous MeOH (0.25 mL). The reaction was stirred for 72 h at 50 °C in an oil bath. The workup was performed following the general procedure. The crude material was purified by flash chromatography on silica gel using 20:1 hexanes/ethyl acetate as eluent to yield the product **1u** (148 mg, 44%) as a yellowish oil. ^{1}H NMR (500 MHz, $CDCl_3$): δ 8.04 (d, J = 8.3 Hz, 2H), 7.44–7.46 (m, 2H), 7.30–7.32 (m, 3H), 7.19 (d, J = 8.4 Hz, 2H), 5.80–5.88 (m, 2H), 5.11–5.18 (m, 4H), 4.36 (s, 1H), 3.01–3.04 (m, 2H), 2.81–2.86 (m, 2H), 2.64–2.75 (m, 2H), 2.58–2.61 (m, 2H), 2.37 (s, 3H), 1.87–1.93 (m, 2H) ppm; ^{13}C-APT NMR (125 MHz, $CDCl_3$): δ 136.9, 136.2, 135.2, 131.7, 130.8, 128.7, 128.2, 128.1, 123.1, 117.3, 88.9, 83.5, 64.8, 64.1, 55.6, 27.8, 27.2, 25.4, 20.9 ppm; HRMS [TOF MS ES+]: m/z [M + H]+ calcd. for $C_{26}H_{30}NS_2$ 420.1820, found 420.1819 (− 0.2 ppm).

4-(1-(2-Methyl-1,3-dithian-2-yl)-3-phenylprop-2-yn-1-yl)thiomorpholine (1y): The reaction was performed following General Procedure B with 2-methyl-1,3-dithane-2-carbaldehyde (**1'**) (1.0 mmol, 162 mg), thiomorpholine (2.0 mmol, 206 mg) and phenyl acetylene (3.0 mmol, 306 mg). In glovebox, the reaction vial was charged with $AuBr_3$ (0.1 mmol, 43.6 mg), activated molecular sieves (300 mg, 3 Å) and anhydrous MeOH (0.5 mL). The reaction was stirred for 24 h at 70 °C in an oil bath. The workup was performed following the general procedure. The crude material was purified by flash

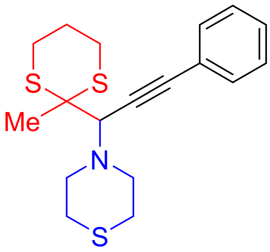

chromatography on silica gel using 20:1 hexanes/ethyl acetate as eluent to yield the product **1y** (218 mg, 62%) as a yellowish-white solid. ^{1}H NMR (500 MHz, $CDCl_3$): δ 7.45–7.47 (m, 2H), 7.30–7.31 (m, 3H), 3.83 (s, 1H), 3.25–3.29 (m, 2H), 2.82–2.99 (m, 6H), 2.68–2.77 (m, 4H), 1.89–2.04 (m, 2H), 1.79 (s, 3H) ppm; ^{13}C-APT NMR (125 MHz, $CDCl_3$): δ 131.7, 128.2, 122.8, 88.9, 83.7, 68.7, 54.7, 54.2, 28.3, 27.1, 26.6, 26.5, 25.1 ppm, [one CH signal belonging to phenyl was not observed or co-incident with the peak appeared at 128.2 ppm]; HRMS [TOF MS ES+]: m/z $[M + H]^+$ $C_{18}H_{24}NS_3$ calcd. for 350.1071, found 350.1069 (−0.6 ppm).

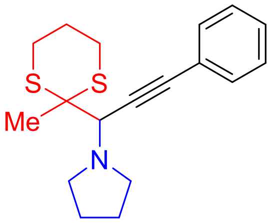

1-(1-(2-methyl-1,3-dithian-2-yl)-3-phenylprop-2-yn-1-yl)pyrrolidine (1ad): The reaction was performed following General Procedure B with 2-methyl-1,3-dithane-2-carbaldehyde (**1'**) (1.0 mmol, 162 mg), pyrrolidine (2.0 mmol, 142 mg) and phenyl acetylene (3.0 mmol, 306 mg). In glovebox, the reaction vial was charged with $AuBr_3$ (0.1 mmol, 43.6 mg), activated molecular sieves (300 mg, 3 Å) and anhydrous MeOH (0.25 mL). The reaction was stirred for 24 h at 70 °C in an oil bath. The workup was performed following the general procedure. The crude material was purified by flash chromatography on silica gel using 20:1 hexanes/ethyl acetate as eluent to yield the product **1ad** (318 mg, 98%) as a yellowish-white solid. ^{1}H NMR (500 MHz, $CDCl_3$): δ ^{1}H NMR (500 MHz, $CDCl_3$): δ 7.46–7.48 (m, 2H), 7.28–7.31 (m, 3H), 4.26 (s, 1H, CH), 2.84–2.96 (m, 8H), 2.00–2.08 (m, 1H), 1.89–1.97 (m, 1H), 1.87 (s, 3H, Me), 1.77–1.79 (m, 4H) ppm; ^{13}C-APT NMR (125 MHz, $CDCl_3$): δ 131.8, 128.2, 128.1, 123.1, 88.5, 84.1, 63.9, 54.4, 51.6, 26.8, 26.7, 26.0, 25.4, 24.2 ppm.

5. Synthesis of 2-(3-phenylprop-2-yn-1-yl)-2-(*p*-tolyl)-1,3-dithiane (1a$_1$)

Scheme S2. Synthesis of (**1a$_1$**) and its cyclization reaction under standard conditions

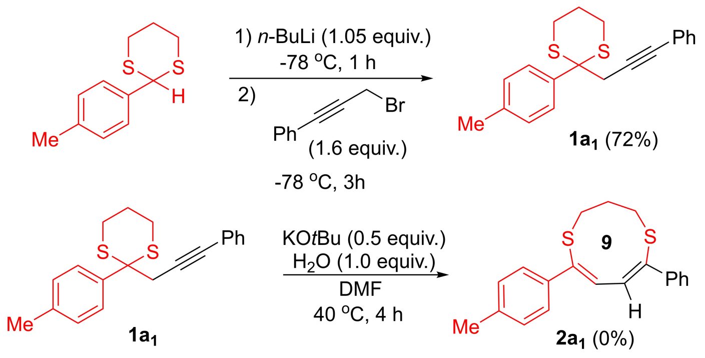

Synthesis of **1a$_1$**: To a solution of 2-(*p*-tolyl)-1,3-dithiane (1.0 g, 4.75 mmol) in THF (20 mL), cooled to −78 °C under nitrogen atmosphere, was added dropwise a solution of *n*-BuLi (2.0 mL of a 2.5 M of hexane solution, 5.0 mmol). The solution was stirred for 1 h at −78 °C and then (3-bromoprop-1-yn-1-yl)benzene (7.7 mmol, 1.5 g) was added dropwise at this temperature [(3-bromoprop-1-yn-1-yl)benzene was synthesized according to procedure in *J. Am. Chem. Soc.* **2009**, *131*, 9178–9179]. The resulting mixture was stirred for 3 h at −78 °C. The reaction mixture was quenched with water and extracted with ether. The organic phase was dried over $MgSO_4$, filtrated and the solvent was removed in a rotatory evaporator. The remaining residue was loaded onto a silica gel column and purified by flash chromatography on silica gel using 20:1 hexanes/ethyl acetate as eluent to yield the product **1a$_1$** (1.1 g, 72%) as a colorless oil. ^{1}H NMR (500 MHz, $CDCl_3$): δ 7.93 (d, J = 8.2 Hz, 2H), 7.32–7.36 (m, 2H), 7.23–7.29 (m, 3H), 7.22 (d, J = 8.2 Hz, 2H), 3.16 (s, 2H, CH_2), 2.72–2.81 (m, 4H), 2.38 (s, 3H), 1.95–2.00 (m, 2H) ppm; ^{13}C-APT NMR (125 MHz, $CDCl_3$): δ 137.6, 137.1, 131.6, 129.1, 128.8, 128.0, 127.8, 123.4, 84.4, 84.3, 57.5, 35.9, 27.9, 24.8, 20.9 ppm.

6. Synthesis of 2-phenyl-1,3-dithiolane-2-carbaldehyde (4'a) and 2-phenyl-1,3-dithiepan-2-carbaldehyde (5'a)

2-Phenyl-1,3-dithiolane-2-carbaldehyde (**4'a**) and 2-phenyl-1,3-dithiepan-2-carbaldehyde (**5'a**) were synthesized following the procedures given below. Alcohols **4'a-OH** and **5'a-OH** were prepared according to the published procedure, please see: *J. Am. Chem. Soc.* **2018**, *140*, 2629−2642.

Scheme S3. Synthesis of 2-phenyl-1,3-dithiolane-2-carbaldehyde (**4'a**) and 2-phenyl-1,3-dithiepan-2-carbaldehyde (**5'a**)

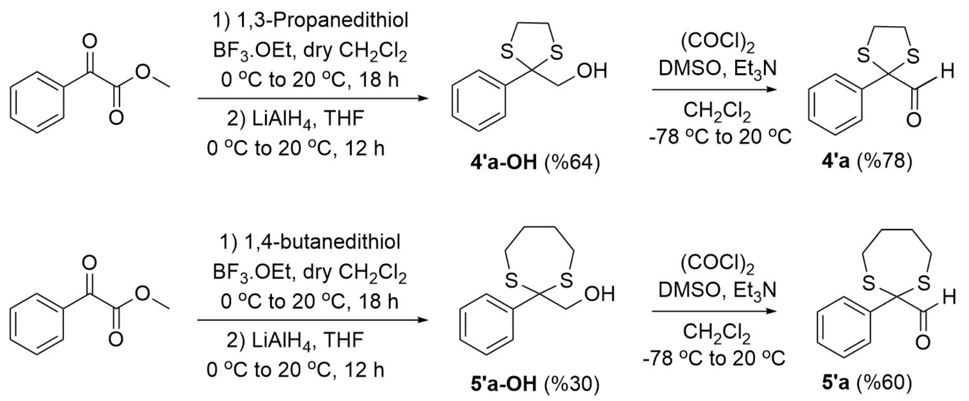

2-Phenyl-1,3-dithiolane-2-carbaldehyde (**4'a**): Dimethyl sulfoxide (4.7 mL, 66.5 mmol, 5.0 equiv.) was added slowly to oxalyl chloride (2.28 mL, 26.60 mmol, 2.0 equiv.) in CH_2Cl_2 (40 mL) at -70 °C. After 20 min, the alcohol **4'a-OH** (2.83 gr, 13.30 mmol, 1.0 equiv.) in CH_2Cl_2 (10 mL) was added slowly. After 1 h, Et_3N (9.27 mL, 66.5 mmol, 5.0 equiv.) was added and the mixture was allowed to warm to room temperature. The reaction mixture was quenched with water and extracted with CH_2Cl_2. The organic phase was dried over Na_2SO_4, filtrated and the solvent was removed in a rotatory evaporator. The remaining residue was loaded onto a silica gel column and purified by flash chromatography on silica gel using 10:1 hexanes/ethyl acetate as eluent to yield the product **4'a** (2.2 g, 78%) as a colorless oil. ^{1}H NMR (500 MHz, $CDCl_3$): δ 9.28 (s, 1H), 7.48–7.59 (m, 2H), 7.34–7.40 (m, 3H), 3.33–3.51 (m, 4H) ppm; ^{13}C-APT NMR (125 MHz, $CDCl_3$): δ 186.2, 134.7, 128.8, 128.7, 128.2, 76.9, 39.9 ppm. HRMS [TOF MS ES+]: m/z $[M + H]^+$ calcd. for $C_{10}H_{11}OS_2$ 211.0251, found 211.0252 (0.5 ppm).

2-Phenyl-1,3-dithiepan-2-carbaldehyde (**5'a**): Dimethyl sulfoxide (1.6 mL, 22.5 mmol, 5.0 equiv.) was added slowly to oxalyl chloride (0.77 mL, 9.0 mmol, 2.0 equiv.) in CH_2Cl_2 (20 mL) at -70 °C. After 20 min, the alcohol **5'a-OH** (1.08 gr, 4.5 mmol, 1.0 equiv.) in CH_2Cl_2 (10 mL) was added slowly. After 1 h, Et_3N (3.13 mL, 12.5 mmol, 5.0 equiv.) was added and the mixture was allowed to warm to room temperature. The reaction mixture was quenched with water and extracted with CH_2Cl_2. The organic phase was dried over Na_2SO_4, filtrated and the solvent was removed in a rotatory evaporator. The remaining residue was loaded onto a silica gel column and purified by flash chromatography on silica gel using 10:1 hexanes/ethyl acetate as eluent to yield the product **5'a** (1.43 g, 60%) as a white solid. ^{1}H NMR (500 MHz, $CDCl_3$): δ 9.25 (s, 1H), 7.55–7.58 (m, 2H), 7.39–7.42 (m, 2H), 7.32–7.35 (m, 1H), 2.84–2.98 (m, 4H), 2.00–2.06 (m, 4H) ppm; ^{13}C-APT NMR (125 MHz, $CDCl_3$): δ 187.4, 137.4, 128.9, 128.6, 127.9, 71.6, 31.1, 29.0 ppm. HRMS [TOF MS ES+]: m/z $[M + H]^+$ calcd. for $C_{12}H_{15}OS_2$ 239.0564, found 239.0573 (3.8 ppm).

7. Synthesis of 1-(1,3-dithiolan-2-yl)propargylamines (4a-e)

1-(1,3-Dithiolan-2-yl)propargylamines were synthesized according to the procedure (General Procedure C) given below.

Table S3. Substrate scope of 1-(1,3-dithiolan-2-yl)propargylamines (**4a-e**)

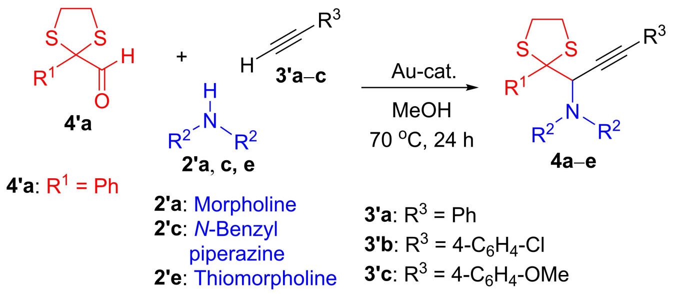

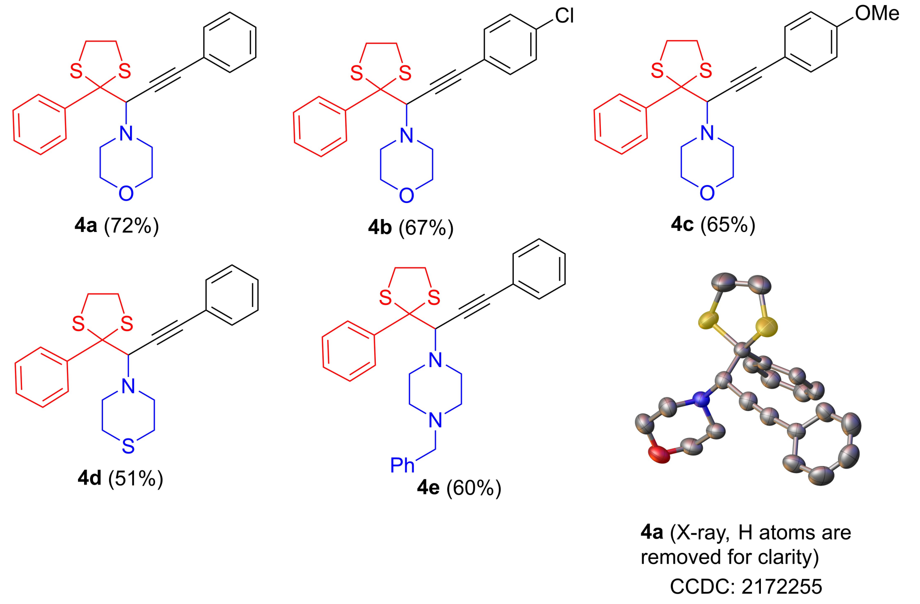

7.1. General Procedure C: An oven-dried 15 mL screw-cap reaction vial equipped with a stirring bar was charged with 1,3-dithiolane-2-carbaldehyde derivative (1.0 equiv.), secondary amine (2.0 equiv.), and alkyne (3.0 equiv.) and then the vial was brought into a glovebox. The reaction vial was charged with Au catalyst (10 mol% or 15 mol%), activated molecular sieves (3 Å) and anhydrous MeOH. The vial was tightly closed, wrapped with a strip of Parafilm, and taken out of the glovebox. After the reaction mixture was stirred for the given time at the stated temperature, the resulting mixture was filtered through a small pad of Celite. The pad was washed with CH_2Cl_2 and then the obtained organic

solution was mixed with silica gel. The solvent was evaporated in a rotatory evaporator. The remaining solid residue was loaded onto a silica gel column and purified by flash chromatography.

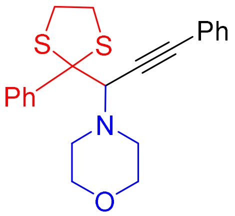

4-(3-Phenyl-1-(2-phenyl-1,3-dithiolan-2-yl)prop-2-yn-1-yl)morpholine (4a): The reaction was performed following General Procedure C with 2-phenyl-1,3-dithiolane-2-carbaldehyde (**4'a**) (1.0 mmol, 214 mg), morpholine (2.0 mmol, 174 mg) and phenyl acetylene (3.0 mmol, 306 mg). In glovebox, the reaction vial was charged with $AuCl_4K$ (0.15 mmol, 56.7 mg), molecular sieves (300 mg, 3 Å) and anhydrous MeOH (0.5 mL). The reaction was stirred for 24 h at 70 ºC in an oil bath. The workup was performed following the general procedure. The crude material was purified by flash chromatography on silica gel using 20:1 hexanes/ethyl acetate as eluent to yield the product **4a** (275 mg, 72%) as a yellowish white solid. ^{1}H NMR (500 MHz, $CDCl_3$): δ 7.89–7.92 (m, 2H), 7.41–7.44 (m, 2H), 7.21–7.32 (m, 6H), 4.22 (s, 1H), 3.53–3.60 (m, 4H), 3.32–3.42 (m, 2H), 3.17–3.24 (m, 2H), 2.48–2.52 (m, 2H), 2.27 (bs, 2H) ppm; ^{13}C-APT NMR (125 MHz, $CDCl_3$): δ 142.4, 131.6, 129.2, 128.2, 127.4, 127.0, 122.8, 89.1, 84.9, 79.4, 71.2, 67.2, 52.6, 39.5, 38.6 ppm. HRMS [TOF MS ES+]: m/z $[M + H]^+$ calcd. for $C_{22}H_{24}NOS_2$ 382.1299, found 382.1308 (2.4 ppm).

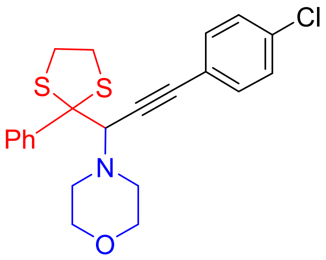

4-(3-(4-Chlorophenyl)-1-(2-phenyl-1,3-dithiolan-2-yl)prop-2-yn-1-yl)morpholine (4b): The reaction was performed following General Procedure C with 2-phenyl-1,3-dithiolane-2-carbaldehyde (**4'a**) (1.21 mmol, 254 mg), morpholine (2.42 mmol, 210 mg) and 1-chloro-4-ethynylbenzene (3.63 mmol, 495 mg). In glovebox, the reaction vial was charged with $AuBr_3$ (0.12 mmol, 53 mg), molecular sieves (300 mg, 3 Å) and anhydrous MeOH (0.6 mL). The reaction was stirred for 24 h at 70 ºC in an oil bath. The workup was performed following the general procedure. The crude material was purified by flash chromatography on silica gel using 20:1 hexanes/ethyl acetate as eluent to yield the product **4b** (278 mg, 67%) as a yellowish white solid. ^{1}H NMR (500 MHz, $CDCl_3$): δ 7.89–7.91 (m, 2H), 7.36–7.38 (m, 2H), 7.24–7.31 (m, 5H), 4.22 (s, 1H), 3.56–3.62 (m, 4H), 3.40–3.45 (m, 1H), 3.33–3.38 (m, 1H), 3.19–3.27 (m, 2H), 2.48–2.52 (m, 2H), 2.28 (bs, 2H) ppm; ^{13}C-APT NMR (125 MHz, $CDCl_3$): δ 142.2, 134.3, 132.8, 129.1, 128.6,

127.5, 127.1, 121.2, 87.9, 85.9, 79.2, 71.2, 67.2, 52.6, 39.5, 38.6 ppm. HRMS [TOF MS ES+]: m/z $[M + H]^+$ calcd. for $C_{22}H_{23}NOS_2Cl$ 416.0910, found 416.0910 (0.0 ppm).

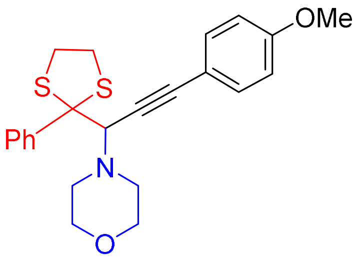

4-(3-(4-Methoxyphenyl)-1-(2-phenyl-1,3-dithiolan-2-yl)prop-2-yn-1-yl)morpholine (4c): The reaction was performed following General Procedure C with 2-phenyl-1,3-dithiolane-2-carbaldehyde (**4'a**) (1.0 mmol, 214 mg), morpholine (2.0 mmol, 174 mg) and 4-ethynylanisole (3.0 mmol, 396 mg). In glovebox, the reaction vial was charged with $AuBr_3$ (0.1 mmol, 43.7 mg), molecular sieves (300 mg, 3 Å) and anhydrous MeOH (0.5 mL). The reaction was stirred for 24 h at 70 ºC in an oil bath. The workup was performed following the general procedure. The crude material was purified by flash chromatography on silica gel using 20:1 hexanes/ethyl acetate as eluent to yield the product **4c** (269 mg, 65%) as a yellowish white solid. ^1H NMR (500 MHz, $CDCl_3$): δ 7.91 (d, J = 7.5 Hz, 2H), 7.37 (d, J = 8.6 Hz, 2H), 7.24–7.29 (m, 3H), 6.84 (d, J = 8.7 Hz, 2H), 4.20 (s, 1H), 3.82 (s, 3H), 3.54–3.60 (m, 4H), 3.39–3.44 (m, 1H), 3.32–3.37 (m, 1H), 3.18–3.25 (m, 2H), 2.48–2.51 (m, 2H), 2.22 (bs, 2H) ppm; ^{13}C-APT NMR (125 MHz, $CDCl_3$): δ 159.6, 142.4, 133.0, 129.2, 127.4, 127.0, 114.9, 113.9, 89.0, 83.4, 79.5, 71.2, 67.3, 55.3, 52.7, 39.5, 38.6 ppm. HRMS [TOF MS ES+]: m/z $[M + H]^+$ calcd. for $C_{23}H_{26}NO_2S_2$ 412.1405, found 412.1405 (0.0 ppm).

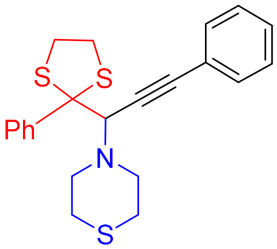

4-(3-Phenyl-1-(2-phenyl-1,3-dithiolan-2-yl)prop-2-yn-1-yl)-thiomorpholine (4d): The reaction was performed following General Procedure C with 2-phenyl-1,3-dithiolane-2-carbaldehyde (**4'a**) (1.0 mmol, 214 mg), thiomorpholine (2.0 mmol, 206 mg) and phenyl acetylene (3.0 mmol, 306 mg). In glovebox, the reaction vial was charged with $AuBr_3$ (0.10 mmol, 43.7 mg), molecular sieves (300 mg, 3 Å) and anhydrous MeOH (0.5 mL). The reaction was stirred for 24 h at 70 ºC in an oil bath. The workup was performed following the general procedure. The crude material was purified by flash chromatography on silica gel using 20:1 hexanes/ethyl acetate as eluent to yield the product **4d** (203 mg, 51%) as a yellowish white solid. ^1H NMR (500 MHz, $CDCl_3$): δ 7.90 (d, J = 7.0 Hz, 2H), 7.43–7.45 (m, 2H), 7.25–7.34 (m, 6H), 4.22 (s, 1H), 3.39–3.45 (m, 1H), 3.33–3.37 (m, 1H), 3.19–3.27

(m, 2H), 2.72–2.76 (m, 2H), 2.50–2.56 (m, 6H) ppm; ^{13}C-APT NMR (125 MHz, $CDCl_3$): δ 142.2, 131.6, 129.2, 128.3, 127.5, 127.0, 122.8, 88.8, 85.1, 79.7, 72.3, 54.9, 39.6, 38.5, 28.4 ppm. HRMS [TOF MS ES+]: m/z $[M + H]^+$ calcd. for $C_{22}H_{24}NS_3$ 398.1071, found 398.1071 (0.0 ppm).

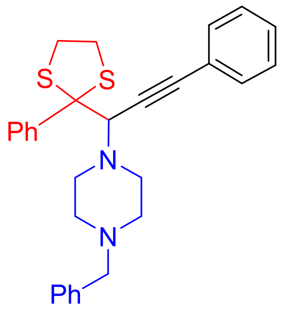

1-Benzyl-4-(3-phenyl-1-(2-phenyl-1,3-dithiolan-2-yl)prop-2-yn-1-yl)piperazine (4e): The reaction was performed following General Procedure C with 2-phenyl-1,3-dithiolane-2-carbaldehyde (**4'a**) (0.68 mmol, 146 mg), *N*-benzylpiperazine (1.36 mmol, 240 mg) and phenyl acetylene (2.04 mmol, 210 mg). In glovebox, the reaction vial was charged with $AuBr_3$ (0.068 mmol, 30.0 mg), molecular sieves (200 mg, 3 Å) and anhydrous MeOH (0.4 mL). The reaction was stirred for 24 h at 70 ºC in an oil bath. The workup was performed following the general procedure. The crude material was purified by flash chromatography on silica gel using 10:1 hexanes/ethyl acetate as eluent to yield the product **4e** (193 mg, 60%) as a yellowish oil. ^{1}H NMR (500 MHz, $CDCl_3$): δ 8.00 (d, J = 7.6 Hz, 2H), 7.48–7.50 (m, 2H), 7.26–7.36 (m, 11H), 4.35 (s, 1H), 3.51–3.54 (m, 2H), 3.43–3.48 (m, 1H), 3.35–3.40 (m, 1H), 3.21–3.29 (m, 2H), 2.43–2.61 (m, 8H) ppm; ^{13}C-APT NMR (125 MHz, $CDCl_3$): δ 142.3, 138.1, 131.5, 129.2, 129.0, 128.2, 128.1, 128.0, 127.3, 126.9, 126.8, 122.9, 88.8, 85.3, 77.3, 70.6, 62.9, 53.4, 39.4, 38.4 ppm, [one CH_2 peak was not observed or co-incident]. HRMS [TOF MS ES+]: m/z $[M + H]^+$ calcd. for $C_{29}H_{31}N_2S_2$ 471.1929, found 471.1928 (−0.2 ppm).

8. Synthesis of 1-(1,3-dithiepan-2-yl)propargylamines (6a-d)

1-(1,3-Dithiepan-2-yl)propargylamines were synthesized according to the procedure (General Procedure D) given below.

Table S4. Substrate scope of 1-(1,3-dithiepan-2-yl)propargylamines (**6a-d**)

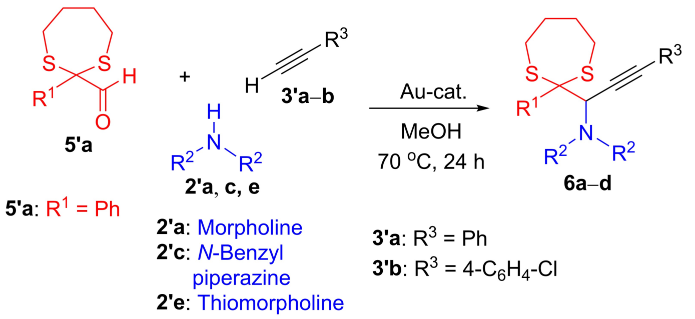

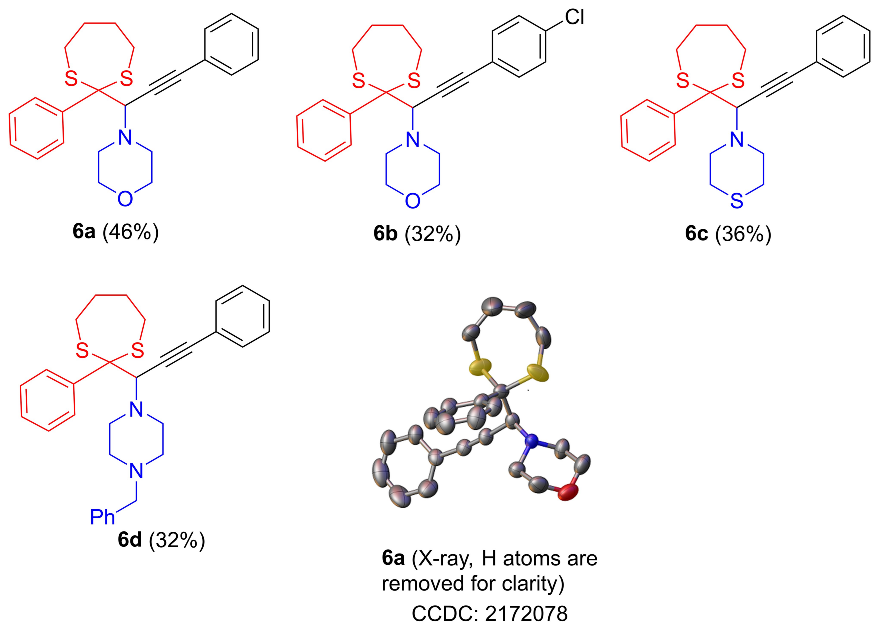

8.1. General Procedure D: An oven-dried 15 mL screw-cap reaction vial equipped with a stirring bar was charged with 1,3-dithiepan-2-carbaldehyde derivative (1.0 equiv.), secondary amine (2.0 equiv.), and alkyne (3.0 equiv.) and then the vial was brought into a glovebox. The reaction vial was charged with Au catalyst (10 mol% or 15 mol%), activated molecular sieves (3 Å) and anhydrous MeOH. The vial was tightly closed, wrapped with a strip of Parafilm, and taken out of the glovebox. After the

reaction mixture was stirred for the given time at the stated temperature, the resulting mixture was filtered through a small pad of Celite. The pad was washed with CH_2Cl_2 and then the obtained organic solution was mixed with silica gel. The solvent was evaporated in a rotatory evaporator. The remaining solid residue was loaded onto a silica gel column and purified by flash chromatography.

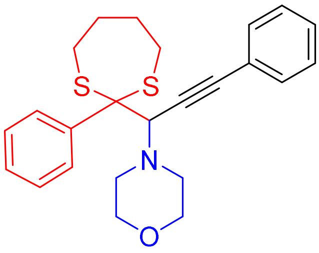

4-(3-Phenyl-1-(2-phenyl-1,3-dithiepan-2-yl)prop-2-yn-1-yl)morpholine (6a): The reaction was performed following General Procedure D with 2-phenyl-1,3-dithiepan-2-carbaldehyde (**5'a**) (1.0 mmol, 238 mg), morpholine (2.0 mmol, 174 mg) and phenyl acetylene (3.0 mmol, 306 mg). In glovebox, the reaction vial was charged with $AuCl_4K$ (0.15 mmol, 56.7 mg), molecular sieves (300 mg, 3 Å) and anhydrous MeOH (0.5 mL). The reaction was stirred for 24 h at 70 °C in an oil bath. The workup was performed following the general procedure. The crude material was purified by flash chromatography on silica gel using 20:1 hexanes/ethyl acetate as eluent to yield the product **6a** (188 mg, 46%) as a yellowish white solid. ^{1}H NMR (500 MHz, $CDCl_3$): δ 8.01–8.04 (m, 2H), 7.42–7.45 (m, 2H), 7.31–7.36 (m, 5H), 7.25–7.28 (m, 1H), 4.05 (s, 1H), 3.58–3.63 (m, 4H), 3.09–3.16 (m, 2H), 2.73–2.79 (m, 1H), 2.65–2.71 (m, 1H), 2.52–2.57 (m, 2H), 2.45–2.49 (m, 2H), 1.87–2.03 (m, 3H), 1.77–1.84 (m, 1H) ppm; ^{13}C-APT NMR (125 MHz, $CDCl_3$): δ 141.7, 131.6, 129.2, 128.2, 128.1, 127.6, 127.4, 122.9, 89.1, 84.3, 72.4, 70.9, 67.2, 52.4, 31.1, 30.9, 29.9, 29.5 ppm. HRMS [TOF MS ES+]: m/z $[M + H]^+$ calcd. for $C_{24}H_{28}NOS_2$ 410.1612, found 410.1621 (2.2 ppm).

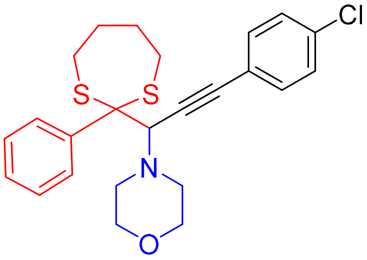

4-(3-(4-Chlorophenyl)-1-(2-phenyl-1,3-dithiepan-2-yl)prop-2-yn-1-yl)morpholine (6b): The reaction was performed following General Procedure D with 2-phenyl-1,3-dithiepan-2-carbaldehyde (**5'a**) (0.87 mmol, 208 mg), morpholine (1.74 mmol, 151 mg) and 1-chloro-4-ethynylbenzene (2.61 mmol, 330 mg). In glovebox, the reaction vial was charged with $AuBr_3$ (0.15 mmol, 65 mg), molecular sieves (260 mg, 3 Å) and anhydrous MeOH (1.0 mL). The reaction was stirred for 24 h at 70 °C in an oil bath. The workup was performed following the general procedure. The crude material was purified by flash chromatography

on silica gel using 30:1 hexanes/ethyl acetate as eluent to yield the product **6b** (153 mg, 36%) as a yellowish oil. ^{1}H NMR (500 MHz, $CDCl_3$): δ 8.01 (d, J = 7.8 Hz, 2H), 7.33–7.36 (m, 4H), 7.25–7.29 (m, 3H), 4.04 (s, 1H), 3.58–3.63 (m, 4H), 3.07–3.15 (m, 2H), 2.72–2.77 (m, 1H), 2.64–2.69 (m, 1H), 2.53–2.57 (m, 2H), 2.44–2.48 (m, 2H), 1.86–2.01 (m, 3H), 1.76–1.83 (m, 1H) ppm; ^{13}C-APT NMR (125 MHz, $CDCl_3$): δ 141.5, 133.9, 132.7, 129.1, 128.4, 127.4, 127.3, 121.3, 87.9, 85.3, 72.2, 70.8, 66.9, 52.3, 30.9, 30.8, 29.8, 29.4 ppm. HRMS [TOF MS ES+]: m/z $[M + H]^+$ calcd. for $C_{24}H_{27}NOS_2Cl$ 444.1223, found 444.1223 (0.0 ppm).

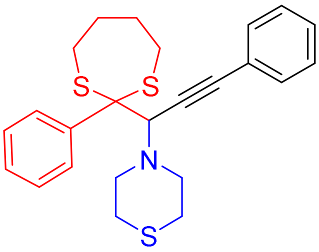

4-(3-Phenyl-1-(2-phenyl-1,3-dithiepan-2-yl)prop-2-yn-1-yl)thiomorpholine (6c): The reaction was performed following General Procedure D with 2-phenyl-1,3-dithiepan-2-carbaldehyde (**5'a**) (1.0 mmol, 238 mg), thiomorpholine (2.0 mmol, 200 mg) and phenyl acetylene (3.0 mmol, 306 mg). In glovebox, the reaction vial was charged with $AuBr_3$ (0.15 mmol, 64 mg), molecular sieves (300 mg, 3 Å) and anhydrous MeOH (0.5 mL). The reaction was stirred for 24 h at 70 ºC in an oil bath. The workup was performed following the general procedure. The crude material was purified by flash chromatography on silica gel using 20:1 hexanes/ethyl acetate as eluent to yield the product **6c** (153 mg, 36%) as a yellowish white solid. ^{1}H NMR (500 MHz, $CDCl_3$): δ 8.00 (d, J = 7.7 Hz, 2H), 7.42–7.44 (m, 2H), 7.26–7.36 (m, 6H), 4.03 (s, 1H), 3.07–3.15 (m, 2H), 2.65–2.81 (m, 6H), 2.52–2.56 (m, 4H), 1.87–2.03 (m, 3H), 1.76–1.84 (m, 1H) ppm; ^{13}C-APT NMR (125 MHz, $CDCl_3$): δ 141.6, 131.6, 129.3, 128.2, 128.1, 127.5, 127.4, 123.1, 88.8, 84.6, 72.7, 71.9, 54.8, 31.1, 30.9, 29.9, 29.5, 28.2 ppm. HRMS [TOF MS ES+]: m/z $[M + H]^+$ calcd. for $C_{24}H_{28}NS_3$ 426.1384, found 426.1385 (0.2 ppm).

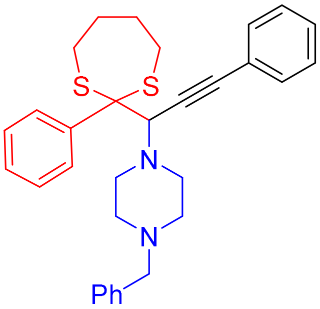

1-Benzyl-4-(3-phenyl-1-(2-phenyl-1,3-dithiepan-2-yl)prop-2-yn-1-yl)piperazine (6d): The reaction was performed following General Procedure D with 2-phenyl-1,3-dithiepan-2-carbaldehyde (**5'a**) (1.0 mmol, 238 mg), *N*-benzylpiperazine (2.0 mmol, 352 mg) and phenyl acetylene (3.0 mmol, 306 mg). In glovebox, the reaction vial was charged with $AuBr_3$ (0.15 mmol, 65 mg), molecular sieves (300 mg, 3 Å) and anhydrous MeOH (0.5

mL). The reaction was stirred for 24 h at 70 ºC in an oil bath. The workup was performed following the general procedure. The crude material was purified by flash chromatography on silica gel using 10:1 hexanes/ethyl acetate as eluent to yield the product **6d** (162 mg, 32%) as a yellowish oil. ^{1}H NMR (500 MHz, $CDCl_3$): δ 8.04 (d, *J* = 7.7 Hz, 2H), 7.43–7.45 (m, 2H), 7.30–7.36 (m, 9H), 7.22–7.28 (m, 2H), 4.11 (s, 1H), 3.48 (s, 2H), 3.07–3.18 (m, 2H), 2.74–2.80 (m, 1H), 2.66–2.71 (m, 1H), 2.50–2.56 (m, 4H), 2.40 (bs, 4H), 1.98–2.05 (m, 1H), 1.89–1.95 (m, 2H), 1.76–1.84 (m, 1H) ppm; ^{13}C-APT NMR (125 MHz, $CDCl_3$): δ 141.7, 138.2, 131.6, 129.2, 129.1, 128.1, 128.0, 127.9, 127.5, 127.3, 126.9, 123.2, 88.9, 84.7, 72.5, 70.4, 63.0, 53.4, 31.1, 30.9, 29.9, 29.4 ppm.

9. Synthesis of 9-membered *S,S*-heterocyles (2a–u) via rearrangement of 1-(1,3-dithian-2-yl)propargylamines (1a–u)

9.1. General procedure E: An oven-dried 15 mL screw-cap reaction vial equipped with a stirring bar was charged with 1-(1,3-dithian-2-yl)propargylamine derivative (1.0 equiv.) and H_2O (1.0 equiv.) by a micropipette and then the vial was brought into a glovebox. The reaction vial was charged with KO*t*Bu (0.5 equiv.) and anhydrous DMF. The vial was tightly closed, wrapped with a strip of Parafilm, and taken out of the glovebox. The reaction mixture was stirred for the given time at the stated temperature. If the reaction was performed at 0.25–0.30 mmol scale or less, the resulting solution was directly loaded onto a column and purified by flash chromatography on silica gel using hexanes/ethyl acetate mixture as eluent to yield the product. Otherwise, the resulting mixture was diluted with ethyl acetate (100 mL) and washed with water (2 × 25 mL). The organic phase was dried over Na_2SO_4, filtrated and the solvent was removed in a rotatory evaporator. The remaining residue was loaded onto a silica gel column and purified by using hexanes/ethyl acetate mixture as eluent to yield the product.

9.2. General Procedure F: An oven-dried 15 mL screw-cap reaction vial equipped with a stirring bar was charged with 1-(1,3-dithian-2-yl)propargylamine derivative (1.0 mmol, 1.0 equiv.) and H_2O (1.0 mmol, 1.0 equiv.) by a micropipette and KOH 80% (w/w) (0.5 mmol, 1.0 equiv.) then the vial was brought into a glovebox. The reaction vial was charged with anhydrous DMF (4.0 mL). The vial was tightly closed, wrapped with a strip of Parafilm, and taken out of the glovebox. The reaction mixture

was stirred for 4 h at 40 ºC in an oil bath. The resulting mixture was diluted with ethyl acetate (100 mL) and washed with water (2 × 25 mL). The organic phase was dried over Na_2SO_4, filtrated and the solvent was removed in a rotatory evaporator. The remaining residue was loaded onto a silica gel column and purified by using hexanes/ethyl acetate mixture as eluent to yield the product. *The purity of KOH sample was determined by alkalimetric titration following the procedure given below*: In the standardization process of KOH, 0.1 mol/L HCl was used as titrant. Since the HCl is not a primary standard, its solution was standardized by a primary standard, sodium carbonate, using acidimetric titration. The pink color produced by phenolphthalein indicator as a marker has ended the alkalimetric titration of HCl with KOH. To calculate the concentration of the examined KOH solution, the following formula was used:

$C_1 = C_2 \times V_2 / V_1$

where C_1 is the precise concentration of KOH, C_2 is the known concentration of standardized HCl solution, V_1 is the volume of the KOH solution, and V_2 is the volume of the standardized HCl solution consumed through titration. After calculating the precise concentration of commercial KOH, its purity was determined as 80% (w/w).

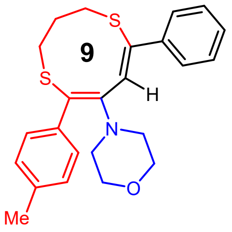

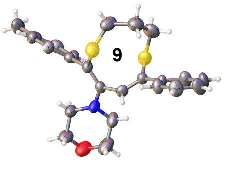

2a (X-ray)
CCDC:2095315

4-((6*E*,8*Z*)-9-Phenyl-6-(*p*-tolyl)-3,4-dihydro-2*H*-1,5-dithionin-7-yl)morpholine (2a): The reaction was performed following General Procedure E with 4-(3-phenyl-1-(2-(*p*-tolyl)-1,3-dithian-2-yl)prop-2-yn-1-yl)morpholine (**1a**) (0.25 mmol, 102 mg) and water (0.25 mmol, 4.5 µL). In glovebox, the reaction vial was charged with KO*t*Bu (0.125 mmol, 14 mg) and anhydrous DMF (1.0 mL). The reaction was stirred for 4 h at 40 ºC in an oil bath. The workup was performed following the general procedure. The crude material was purified by flash chromatography on silica gel using 20:1 hexanes/ethyl acetate as eluent to yield the product **2a** (72 mg, 71%) as an orange solid. When the reaction was performed with 0.5 mmol (205 mg) and 1.0 mmol (410 mg) **1a**, 0.5 mmol (9.0 µL) and 1.0 mmol (18 µL) water was added into the reaction vials, respectively. In glovebox, the reaction vials were charged with KO*t*Bu (0.25 mmol, 28 mg and 0.50 mmol 56 mg) and anhydrous DMF (2.0 mL

and 4.0 mL), respectively. The workup was performed following the general procedure. The crude material was purified by flash chromatography on silica gel using 20:1 hexanes/ethyl acetate as eluent to yield the product **2a** (144 mg, 70% from 0.5 mmol scale reaction and 308 mg, 75% from 1.0 mmol scale reaction) as an orange solid. The reaction also was performed following General Procedure F with 4-(3-phenyl-1-(2-(*p*-tolyl)-1,3-dithian-2-yl)prop-2-yn-1-yl)morpholine (**1a**) (1.00 mmol, 408 mg), water (1.0 mmol, 18.0 μL) and KOH (80% w/w) (0.5 mmol, 34 mg) and anhydrous DMF (4.0 mL). The reaction was stirred for 4 h at 40 °C in an oil bath. The workup was performed following the general procedure. The crude material was purified by flash chromatography on silica gel using 20:1 hexanes/ethyl acetate as eluent to yield the product **2a** (343 mg, 84%) as an orange solid. ^{1}H NMR (500 MHz, $CDCl_3$): δ 7.81–7.83 (m, 2H), 7.38–7.46 (m, 4H), 7.35–7.37 (m, 1H), 7.15 (d, J = 8.1 Hz, 2H), 6.72 (s, 1H), 3.46–3.52 (m, 4H), 2.87–2.92 (m, 2H), 2.66–2.76 (m, 3H), 2.37 (s, 3H), 2.30–2.43 (m, 3H), 1.98–2.05 (m, 1H), 1.79–1.87 (m, 1H) ppm; ^{13}C-APT NMR (125 MHz, $CDCl_3$): δ 150.6, 140.0, 139.6, 138.8, 135.9, 135.6, 130.1, 128.6, 128.5, 128.4, 127.9, 106.8, 67.2, 50.5, 33.6, 32.6, 31.1, 21.2 ppm. HRMS [TOF MS ES+]: m/z $[M + H]^+$ calcd. for $C_{24}H_{28}NOS_2$ 410.1612, found 410.1613 (0.2 ppm).

4-((6*E*,8*Z*)-9-(4-Chlorophenyl)-6-(*p*-tolyl)-3,4-dihydro-2*H*-1,5-dithionin-7-yl)morpholine (2b):

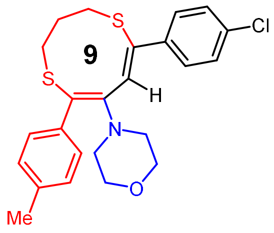

The reaction was performed following General Procedure E with 4-(3-(4-chlorophenyl)-1-(2-(*p*-tolyl)-1,3-dithian-2-yl)prop-2-yn-1-yl)morpholine (**1b**) (0.17 mmol, 75 mg) and water (0.17 mmol, 3.0 μL). In glovebox, the reaction vial was charged with KO*t*Bu (0.085 mmol, 9.5 mg) and anhydrous DMF (0.7 mL). The reaction was stirred for 4 h at 40 °C in an oil bath. The workup was performed following the general procedure. The crude material was purified by flash chromatography on silica gel using 20:1 hexanes/ethyl acetate as eluent to yield the product **2b** (46 mg, 61%) as an orange solid. ^{1}H NMR (500 MHz, $CDCl_3$): δ 7.73–7.76 (m, 2H), 7.42–7.45 (m, 2H), 7.36–7.39 (m, 2H), 7.15 (d, J = 7.6 Hz, 2H), 6.70 (s, 1H), 3.46–3.51 (m, 4H), 2.85–2.89 (m, 2H), 2.64–2.70 (m, 3H), 2.36 (s, 3H), 2.27–2.45 (m, 3H), 1.97–2.05 (m, 1H), 1.76–1.84 (m, 1H) ppm; ^{13}C-APT NMR (125 MHz, $CDCl_3$): δ 150.3, 138.8, 138.5, 138.2, 136.4, 135.7, 134.3, 130.1, 129.2, 128.6, 128.5, 107.3, 67.1, 50.5, 33.4,

32.5, 31.2, 21.2 ppm. HRMS [TOF MS ES+]: m/z $[M + H]^+$ calcd. for $C_{24}H_{27}NOS_2Cl$ 444.1223, found 444.1224 (0.2 ppm).

4-((6*E*,8*Z*)-9-(4-Methoxyphenyl)-6-(*p*-tolyl)-3,4-dihydro-2*H*-1,5-dithionin-7-yl)morpholine (2c):

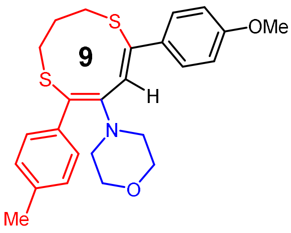

The reaction was performed following General Procedure E with 4-(1-(2-(4-methoxyphenyl)-1,3-dithian-2-yl)-3-phenylprop-2-yn-1-yl)morpholine (**1c**) (0.26 mmol, 119 mg) and water (0.26 mmol, 4.7 µL). In glovebox, the reaction vial was charged with KO*t*Bu (0.13 mmol, 15 mg) and anhydrous DMF (1.5 mL). The reaction was stirred for 4 h at 40 ºC in an oil bath. The workup was performed following the general procedure. The crude material was purified by flash chromatography on silica gel using 10:1 hexanes/ethyl acetate as eluent to yield the product **2c** (64 mg, 54%) as an orange solid. ^{1}H NMR (500 MHz, $CDCl_3$): δ7.76 (d, *J* = 8.8 Hz, 2H), 7.44 (d, *J* = 8.0 Hz, 2H), 7.14 (d, *J* = 8.6 Hz, 2H), 6.95 (d, *J* = 8.7 Hz, 2H), 6.60 (s, 1H), 3.86 (s, 3H), 3.46–3.51 (m, 4H), 2.87–2.93 (m, 2H), 2.74 (dt, *J* = 4.0, 14.3 Hz, 1H), 2.65–2.70 (m, 2H), 2.36 (s, 3H), 2.30–2.41 (m, 3H), 1.97–2.05 (m, 1H), 1.77–1.85 (m, 1H) ppm; ^{13}C-APT NMR (125 MHz, $CDCl_3$): δ 159.9, 150.9, 139.8, 138.9, 135.4, 134.0, 132.0, 130.1, 129.2, 128.5, 113.9, 106.4, 67.2, 55.3, 50.5, 33.4, 32.6, 31.1, 21.2 ppm. HRMS [TOF MS ES+]: m/z $[M + H]^+$ calcd. for $C_{25}H_{30}NO_2S_2$ 440.1718, found 440.1718 (0.0 ppm).

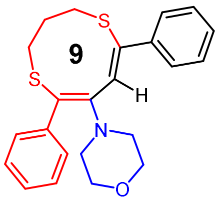

4-((6*E*,8*Z*)-6,9-Diphenyl-3,4-dihydro-2*H*-1,5-dithionin-7-yl)morpholine (2d): The reaction was performed following General Procedure E with 4-(3-phenyl-1-(2-phenyl-1,3-dithian-2-yl)prop-2-yn-1-yl)morpholine (**1d**) (0.25 mmol, 99 mg) and water (0.25 mmol, 4.5 µL). In glovebox, the reaction vial was charged with KO*t*Bu (0.125 mmol, 14 mg) and anhydrous DMF (1.0 mL). The reaction was stirred for 4 h at 40 ºC in an oil bath. The workup was performed following the general procedure. The crude material was purified by flash chromatography on silica gel using 20:1 hexanes/ethyl acetate as eluent to yield the product **2d** (67 mg, 68%) as an orange solid. ^{1}H NMR (500 MHz, $CDCl_3$): δ 7.83 (d, *J* = 7.3 Hz, 2H), 7.56 (d, *J* = 6.7 Hz, 2H), 7.41–7.44 (m, 2H), 7.33–7.39 (m, 3H), 7.18 (t, *J* = 7.4 Hz, 1H), 6.73 (s, 1H), 3.45–3.52 (m, 4H), 2.88–2.92 (m, 2H), 2.67–2.78 (m, 3H), 2.32–2.41 (m, 3H), 1.97–2.06 (m, 1H), 1.79–1.87 (m, 1H) ppm; ^{13}C-APT NMR (125 MHz, $CDCl_3$): δ 151.1, 141.9, 140.4, 139.5, 135.8,

130.2, 128.6, 128.5, 128.0, 127.8, 125.9, 106.3, 67.1, 50.5, 33.4, 32.6, 31.2 ppm. HRMS [TOF MS ES+]: m/z $[M + H]^+$ calcd. for $C_{23}H_{26}NOS_2$ 396.1456, found 396.1456 (0.0 ppm).

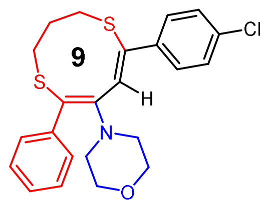

4-((6*E*,8*Z*)-9-(4-Chlorophenyl)-6-phenyl-3,4-dihydro-2*H*-1,5-dithionin-7-yl)morpholine (2e): The reaction was performed following General Procedure E with 4-(3-(4-chlorophenyl)-1-(2-phenyl-1,3-dithian-2-yl)prop-2-yn-1-yl)morpholine (**1e**) (0.41 mmol, 177 mg) and water (0.41 mmol, 7.4 µL). In glovebox, the reaction vial was charged with KO*t*Bu (0.205 mmol, 23 mg) and anhydrous DMF (1.6 mL). The reaction was stirred for 4 h at 40 °C in an oil bath. The workup was performed following the general procedure. The crude material was purified by flash chromatography on silica gel using 20:1 hexanes/ethyl acetate as eluent to yield the product **2e** (115 mg, 65%) as an orange solid. The reaction also was performed following General Procedure F with 4-(3-(4-chlorophenyl)-1-(2-phenyl-1,3-dithian-2-yl)prop-2-yn-1-yl)morpholine (**1e**) (1.00 mmol, 430 mg), water (1.0 mmol, 18.0 µL) and KOH (80% w/w) (0.5 mmol, 34 mg) and anhydrous DMF (4.0 mL). The reaction was stirred for 4 h at 40 °C. The workup was performed following the general procedure. The crude material was purified by flash chromatography on silica gel using 20:1 hexanes/ethyl acetate as eluent to yield the product **2e** (281 mg, 65%) as an orange solid. ^1H NMR (500 MHz, $CDCl_3$): δ 7.75 (d, *J*= 8.3 Hz, 2H), 7.54 (d, *J*= 7.5 Hz, 2H), 7.39 (d, *J*= 8.4 Hz, 2H), 7.34 (t, *J*= 7.6 Hz, 2H), 7.18 (t, *J*= 7.4 Hz, 1H), 6.70 (s, 1H), 3.45–3.52(m, 4H), 2.85–2.90 (m, 2H), 2.65–2.72 (m, 3H), 2.27–2.44 (m, 3H), 1.98–2.05 (m, 1H), 1.75–1.83 (m, 1H), ppm; ^{13}C-APT NMR (125 MHz, $CDCl_3$): δ 150.8, 141.6, 139.1, 138.1, 136.2, 134.4, 130.2, 129.2, 128.7, 127.9, 126.0, 106.7, 67.1, 50.5, 33.4, 32.5, 31.2 ppm. HRMS [TOF MS ES+]: m/z $[M + H]^+$ calcd. for $C_{23}H_{25}NOS_2Cl$ 430.1066, found 430.1069 (0.7 ppm).

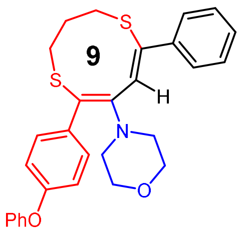

4-((6*E*,8*Z*)-6-(4-Phenoxyphenyl)-9-phenyl-3,4-dihydro-2*H*-1,5-dithionin-7-yl)morpholine (2f): The reaction was performed following General Procedure E with 4-(1-(2-(4-phenoxyphenyl)-1,3-dithian-2-yl)-3-phenylprop-2-yn-1-yl)morpholine (**1f**) (0.30 mmol, 146 mg) and water (0.30 mmol, 5.4 µL). In glovebox, the reaction vial was charged with KO*t*Bu (0.15 mmol, 17 mg) and anhydrous DMF (1.2 mL). The reaction was stirred for 4 h at 40 °C in an oil bath. The workup was performed

following the general procedure. The crude material was purified by flash chromatography on silica gel using 20:1 hexanes/ethyl acetate as eluent to yield the product **2f** (91 mg, 62%) as an orange solid. ^{1}H NMR (500 MHz, $CDCl_3$): δ 7.82 (d, *J*= 7.1 Hz, 2H), 7.52 (d, *J*= 8.5 Hz, 2H), 7.41–7.44 (m, 2H), 7.34–7.38 (m, 3H), 7.12 (t, *J*= 7.4 Hz, 1H), 7.06 (d, *J*= 8.1 Hz, 2H), 6.99 (d, *J*= 8.5 Hz, 2H), 6.70 (s, 1H), 3.51 (t, *J*= 4.8 Hz, 4H), 2.89–2.93 (m, 2H), 2.78 (dt, *J*= 4.0, 14.3 Hz, 1H), 2.68–2.73 (m, 2H), 2.35–2.42 (m, 3H), 2.01–2.08 (m, 1H) ppm; ^{13}C-APT NMR (125 MHz, $CDCl_3$): δ 157.1, 155.3, 150.9, 140.4, 139.5, 136.5, 135.7, 131.5, 129.7, 128.6, 128.5, 127.9, 123.2, 118.9, 118.1, 106.0, 67.2, 50.5, 33.4, 32.7, 31.1 ppm. HRMS [TOF MS ES+]: m/z $[M + H]^+$ calcd. for $C_{29}H_{30}NO_2S_2$ 488.1718, found 488.1713 (−1.0 ppm).

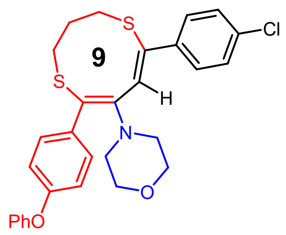

4-((6*E*,8*Z*)-9-(4-Chlorophenyl)-6-(4-phenoxyphenyl)-3,4-dihydro-*2H*-1,5-dithionin-7-yl)morpholine (2g): The reaction was performed following General Procedure E with 4-(3-(4-chlorophenyl)-1-(2-(4-phenoxyphenyl)-1,3-dithian-2-yl)prop-2-yn-1-yl)morpholine (**1g**) (0.27 mmol, 142 mg) and water (0.27 mmol, 4.9 μL). In glovebox, the reaction vial was charged with KO*t*Bu (0.135 mmol, 15.0 mg) and anhydrous DMF (1.1 mL). The reaction was stirred for 4 h at 40 °C in an oil bath. The workup was performed following the general procedure. The crude material was purified by flash chromatography on silica gel using 20:1 hexanes/ethyl acetate as eluent to yield the product **2g** (65 mg, %46) as an orange solid. ^{1}H NMR (500 MHz, $CDCl_3$): δ 7.74 (d, *J*= 8.5 Hz, 2H), 7.51 (d, *J*= 8.6 Hz, 2H), 7.34–7.39 (m, 4H), 7.12 (t, *J*= 7.4 Hz, 1H), 7.05 (d, *J*= 7.4 Hz, 2H), 6.99 (d, *J*= 8.7 Hz, 2H), 6.69 (s, 1H), 3.49–3.53 (m, 4H), 2.87–2.91 (m, 2H), 2.67–2.74 (m, 3H), 2.31–2.44 (m, 3H), 2.00–2.07 (m, 1H), 1.76–1.84 (m, 1H) ppm; ^{13}C-APT NMR (125 MHz, $CDCl_3$): δ 157.1, 155.4, 150.6, 139.2, 138.0, 136.7, 136.2, 134.4, 131.4, 129.8, 129.2, 128.7, 123.3, 118.9, 118.1, 106.5, 67.2, 50.5, 33.4, 32.6, 31.1 ppm. HRMS [TOF MS ES+]: m/z $[M + H]^+$ calcd. for $C_{29}H_{29}NO_2S_2Cl$ 522.1328, found 522.1328 (0.0 ppm).

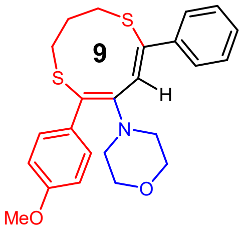

4-((6E,8Z)-6-(4-Methoxyphenyl)-9-phenyl-3,4-dihydro-2*H*-1,5-dithionin-7-yl)morpholine (2h): The reaction was performed following General Procedure E with 4-(1-(2-(4-methoxyphenyl)-1,3-dithian-2-yl)-3-phenylprop-2-yn-1-yl)morpholine (**1h**) (0.25 mmol, 106 mg) and water (0.25 mmol, 4.5 μL). In glovebox, the reaction vial was charged with KO*t*Bu (0.125 mmol, 14 mg) and anhydrous DMF (1.0 mL). The reaction was stirred for 4 h at 40 °C in an oil bath. The workup was performed following the general procedure. The crude material was purified by flash chromatography on silica gel using 20:1 hexanes/ethyl acetate as eluent to yield the product **2h** (71 mg, 67%) as an orange solid. The reaction also was performed following General Procedure F with 4-(1-(2-(4-methoxyphenyl)-1,3-dithian-2-yl)-3-phenylprop-2-yn-1-yl)morpholine (**1h**) (1.00 mmol, 424 mg), water (1.0 mmol, 18.0 μL) and KOH (80% w/w) (0.5 mmol, 34 mg) and anhydrous DMF (4.0 mL). The reaction was stirred for 4 h at 40 °C. The workup was performed following the general procedure. The crude material was purified by flash chromatography on silica gel using 20:1 hexanes/ethyl acetate as eluent to yield the product **2h** (318 mg, 75%) as an orange solid. ^{1}H NMR (500 MHz, $CDCl_3$): δ 7.81 (d, *J*= 7.0 Hz, 2H), 7.50 (d, *J*= 8.3 Hz, 2H), 7.35–7.43 (m, 3H), 6.89 (d, *J*= 8.7 Hz, 2H), 6.71 (s, 1H), 3.84 (s, 3H), 3.45–3.52 (m, 4H), 2.86–2.89 (m, 2H), 2.66–2.75 (m, 3H), 2.30–2.43 (m, 3H), 1.98–2.04 (m, 1H), 1.79–1.85 (m, 1H) ppm; ^{13}C-APT NMR (125 MHz, $CDCl_3$): δ 157.8, 150.2, 139.9, 139.7, 136.1, 134.1, 131.3, 128.5, 128.4, 127.9, 113.2, 106.9, 67.2, 55.2, 50.5, 33.4, 32.6, 31.3 ppm. HRMS [TOF MS ES+]: m/z $[M + H]^+$ calcd. for $C_{24}H_{28}NO_2S_2$ 426.1561, found 426.1562 (0.2 ppm).

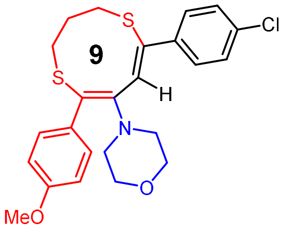

4-((6E,8Z)-9-(4-Chlorophenyl)-6-(4-methoxyphenyl)-3,4-dihydro-*2H*-1,5-dithionin-7-yl)morpholine (**2i**): The reaction was performed following General Procedure E with 4-(3-(4-chlorophenyl)-1-(2-(4-methoxyphenyl)-1,3-dithian-2-yl)prop-2-yn-1-yl)morpholine (**1i**) (0.224 mmol, 103 mg) and water (0.224 mmol, 4.0 μL). In glovebox, the reaction vial was charged with KO*t*Bu (0.112 mmol, 12.6 mg) and anhydrous DMF (0.9 mL). The reaction was stirred for 4 h at 40 °C in an oil bath. The workup was performed following the general procedure. The crude material was

purified by flash chromatography on silica gel using 20:1 hexanes/ethyl acetate as eluent to yield the product **2i** (77 mg, 75%) as an orange solid. ^{1}H NMR (500 MHz, $CDCl_3$): δ 7.74 (d, *J*= 8.5 Hz, 2H), 7.48 (d, *J*= 8.7 Hz, 2H), 7.37 (d, *J*= 8.5 Hz, 2H), 6.89 (d, *J*= 8.7 Hz, 2H), 6.69 (s, 1H), 3.84 (s, 3H), 3.46–3.52 (m, 4H), 2.84–2.88 (m, 2H), 2.64–2.70 (m, 3H), 2.35–2.45 (m, 2H), 2.26–2.32 (m, 1H), 1.97–2.05 (m, 1H), 1.76–1.84 (m, 1H) ppm; ^{13}C-APT NMR (125 MHz, $CDCl_3$): δ 157.9, 149.8, 138.7, 138.2, 136.5, 134.3, 133.8, 131.3, 129.2, 128.6, 113.3, 107.4, 67.2, 55.2, 50.4, 33.4, 32.4, 31.1 ppm. HRMS [TOF MS ES+]: m/z $[M + H]^+$ calcd. for $C_{24}H_{27}NO_2S_2Cl$ 460.1172, found 460.1173 (0.2 ppm).

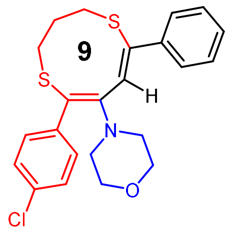

4-((6*E*,8*Z*)-6-(4-Chlorophenyl)-9-phenyl-3,4-dihydro-2*H*-1,5-dithionin-7-yl)morpholine (2j): The reaction was performed following General Procedure E with 4-(1-(2-(4-chlorophenyl)-1,3-dithian-2-yl)-3-phenylprop-2-yn-1-yl)morpholine (**1j**) (0.256 mmol, 110 mg) and water (0.256 mmol, 4.6 μL). In glovebox, the reaction vial was charged with KO*t*Bu (0.128 mmol, 14.0 mg) and anhydrous DMF (0.9 mL). The reaction was stirred for 4 h at 40 °C in an oil bath. The workup was performed following the general procedure. The crude material was purified by flash chromatography on silica gel using 20:1 hexanes/ethyl acetate as eluent to yield the product **2j** (57 mg, 52%) as an orange solid. ^{1}H NMR (500 MHz, $CDCl_3$): δ 7.81 (d, *J*= 7.0 Hz, 2H), 7.48–7.51 (m, 2H), 7.41–7.44 (m, 2H), 7.36–7.39 (m, 1H), 7.29–7.32 (m, 2H), 6.69 (s, 1H), 3.46–3.53(m, 4H), 2.89–2.93 (m, 2H), 2.76 (dt, *J* = 4.0, 18.2 Hz, 1H), 2.67–2.71 (m, 2H), 2.31–2.39 (m, 3H), 1.97–2.04 (m, 1H), 1.76–1.84 (m, 1H), ppm; ^{13}C-APT NMR (125 MHz, $CDCl_3$): δ 151.8, 140.9, 140.6, 139.2, 135.4, 131.4, 131.1, 128.7, 128.5, 127.9, 104.5, 67.0, 50.6, 33.3, 32.8, 31.1 ppm. HRMS [TOF MS ES+]: m/z $[M + H]^+$ calcd. for $C_{23}H_{25}NOS_2Cl$ 430.1066, found 430.1065 (−0.2 ppm).

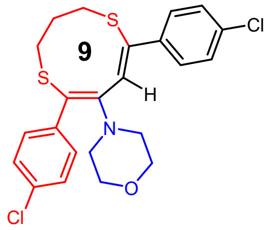

4-((6*E*,8*Z*)-6,9-Bis(4-chlorophenyl)-3,4-dihydro-2*H*-1,5-dithionin-7-yl)morpholine (2k): The reaction was performed following General Procedure E with 4-(3-(4-chlorophenyl)-1-(2-(4-chlorophenyl)-1,3-dithian-2-yl)prop-2-yn-1-yl)morpholine (**1k**) (0.19 mmol, 88 mg) and water (0.19 mmol, 3.4 μL). In glovebox, the reaction vial was charged with KO*t*Bu (0.095 mmol, 11.0 mg) and anhydrous DMF (0.75 mL). The reaction was stirred for 4 h at 40 °C in an oil bath. The workup was

performed following the general procedure. The crude material was purified by flash chromatography on silica gel using 20:1 hexanes/ethyl acetate as eluent to yield the product **2k** (69 mg, 78%) as an orange solid. ^{1}H NMR (500 MHz, $CDCl_3$): δ 7.73 (d, *J*= 8.5 Hz, 2H), 7.48 (d, *J*= 8.5 Hz, 2H), 7.39 (d, *J*= 8.5 Hz, 2H), 7.30 (d, *J*= 8.5 Hz, 2H), 6.67 (s, 1H), 3.47–3.52 (m, 4H), 2.87–2.91 (m, 2H), 2.65–2.73 (m, 3H), 2.31–2.41 (m, 3H), 1.96–2.03 (m, 1H), 1.73–1.81 (m, 1H), ppm; ^{13}C-APT NMR (125 MHz, $CDCl_3$): δ 151.4, 140.4, 139.7, 137.8, 135.9, 134.5, 131.3, 131.2, 129.3, 128.7, 128.1, 104.9, 67.0, 50.6, 33.4, 32.7, 31.2 ppm. HRMS [TOF MS ES+]: m/z $[M + H]^+$ calcd. for $C_{23}H_{24}NOS_2Cl_2$ 464.0676, found 464.0676 (0.0 ppm).

***N,N*-Dimethyl-4-((6*E*,8*Z*)-7-morpholino-9-phenyl-3,4-dihydro-*2H*-1,5-dithionin-6-yl)aniline (2l)**:

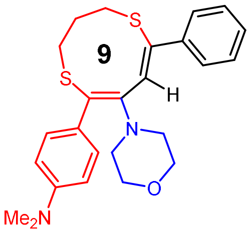

The reaction was performed following General Procedure E with *N,N*-dimethyl-4-(2-(1-morpholino-3-phenylprop-2-ynyl)-1,3-dithian-2-yl)benzenamine (**1l**) (0.25 mmol, 109 mg) and water (0.25 mmol, 4.5 µL). In glovebox, the reaction vial was charged with KO*t*Bu (0.125 mmol, 14.0 mg) and anhydrous DMF (1.0 mL). The reaction was stirred for 4 h at 40 ºC in an oil bath. The workup was performed following the general procedure. The crude material was purified by flash chromatography on silica gel using 20:1 hexanes/ethyl acetate as eluent to yield the product **2l** (73 mg, 67%) as an orange solid. ^{1}H NMR (500 MHz, $CDCl_3$): δ 7.80–7.82 (m, 2H), 7.44–7.47 (m, 2H), 7.40–7.43 (m, 2H), 7.33–7.36 (m, 1H), 6.71–6.74 (m, 3H), 3.47–3.52 (m, 4H), 2.99 (s, 6H), 2.88–2.93 (m, 2H), 2.66–2.74 (m, 3H), 2.39–2.46 (m, 2H), 2.27–2.33 (m, 1H), 1.98–2.05 (m, 1H), 1.79–1.87 (m, 1H) ppm; ^{13}C-APT NMR (125 MHz, $CDCl_3$): δ 149.0, 148.8, 139.9, 139.2, 136.5, 131.1, 129.4, 128.4, 128.3, 127.9, 111.7, 108.5, 67.3, 50.4, 40.5, 33.5, 32.4, 31.1 ppm. HRMS [TOF MS ES+]: m/z $[M + H]^+$ calcd. for $C_{25}H_{31}N_2OS_2$ 439.1878, found 439.1878 (0.0 ppm).

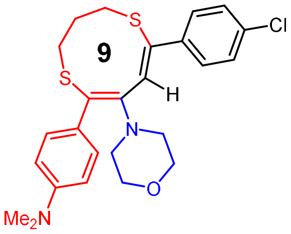

4-((6*E*,8*Z*)-9-(4-Chlorophenyl)-7-morpholino-3,4-dihydro-*2H*-1,5-dithionin-6-yl)-*N,N*-dimethylaniline (2m): The reaction was performed following General Procedure E with *N,N*-Dimethyl-4-(2-(1-morpholino-3-(4-chlorophenyl)prop-2-ynyl)-1,3-dithian-2-yl)benzenamine (**1m**) (0.17 mmol, 80 mg) and water (0.17 mmol, 3.0 µL). In glovebox, the reaction

vial was charged with KO*t*Bu (0.085 mmol, 9.5 mg) and anhydrous DMF (0.7 mL). The reaction was stirred for 4 h at 40 °C in an oil bath. The workup was performed following the general procedure. The crude material was purified by flash chromatography on silica gel using 20:1 hexanes/ethyl acetate as eluent to yield the product **2m** (57 mg, 71%) as an orange solid. ^{1}H NMR (500 MHz, $CDCl_3$): δ 7.72–7.75 (m, 2H), 7.43–7.46 (m, 2H), 7.35–7.38 (m, 2H), 6.71–6.73 (m, 2H), 6.71 (s, 1H), 3.47–3.52 (m, 4H), 2.99 (s, 6H), 2.86–2.91 (m, 2H), 2.64–2.69 (m, 3H), 2.41–2.47 (m, 2H), 2.25–2.30 (m, 1H), 1.97–2.05 (m, 1H), 1.78–1.86 (m, 1H) ppm; ^{13}C-APT NMR (125 MHz, $CDCl_3$): δ 148.9, 148.6, 138.5, 137.9, 136.9, 134.1, 131.1, 129.2, 129.0, 128.6, 111.7, 109.1, 67.3, 50.4, 40.5, 33.5, 32.3, 31.2 ppm. HRMS [TOF MS ES+]: m/z $[M + H]^+$ calcd. for $C_{25}H_{30}N_2OS_2Cl$ 473.1488, found 473.1488 (0.0 ppm).

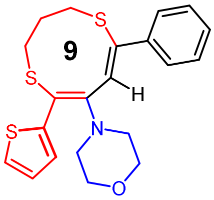

4-((6*E*,8*Z*)-9-Phenyl-6-(thiophen-2-yl)-3,4-dihydro-2H-1,5-dithionin-7-yl)morpholine (2n): The reaction was performed following General Procedure E with 4-(3-phenyl-1-(2-(thiophen-2-yl)-1,3-dithian-2-yl)prop-2-yn-1-yl)morpholine (**1n**) (0.25 mmol, 100 mg) and water (0.25 mmol, 4.5 µL). In glovebox, the reaction vial was charged with KO*t*Bu (0.125 mmol, 14.0 mg) and anhydrous DMF (1.0 mL). The reaction was stirred for 4 h at 40 °C in an oil bath. The workup was performed following the general procedure. The crude material was purified by flash chromatography on silica gel using 20:1 hexanes/ethyl acetate as eluent to yield the product **2n** (77 mg, 77%) as an orange solid. ^{1}H NMR (500 MHz, $CDCl_3$): δ 7.79 (d, *J*= 6.8 Hz, 2H), 7.41–7.44 (m, 2H), 7.35–7.39 (m, 1H), 7.23 (dd, *J*= 1.2, 5.1 Hz, 1H), 7.17 (dd, *J*= 1.2, 3.6 Hz, 1H), 7.01 (dd, *J*= 3.6, 5.2 Hz, 1H), 6.66 (s, 1H), 3.60–3.66 (m, 4H), 3.01–3.04 (m, 2H), 2.80–2.89 (m, 3H), 2.55–2.61 (m, 2H), 2.29–2.36 (m, 1H), 2.06–2.12 (m, 1H), 1.73–1.81 (m, 1H) ppm; ^{13}C-APT NMR (125 MHz, $CDCl_3$): δ 151.6, 145.9, 141.7, 139.1, 134.3, 128.7, 128.6, 127.9, 126.9, 126.4, 125.1, 103.2, 67.0, 50.5, 33.1, 33.0, 31.3 ppm. HRMS [TOF MS ES+]: m/z $[M + H]^+$ calcd. for $C_{21}H_{24}NOS_3$ 402.1020, found 402.1020 (0.0 ppm).

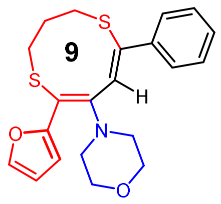

4-((6*E*,8*Z*)-6-(Furan-2-yl)-9-phenyl-3,4-dihydro-2*H*-1,5-dithionin-7-yl)morpholine (2o): The reaction was performed following General Procedure E with 4-(1-(2-(furan-2-yl)-1,3-dithian-2-yl)-3-phenylprop-2-yn-1-yl)morpholine (**1o**) (0.27 mmol, 104 mg) and water (0.27 mmol, 4.8 µL). In glovebox, the reaction vial was charged with KO*t*Bu (0.13 mmol, 15.0 mg) and anhydrous DMF (1.1 mL). The reaction was stirred for 4 h at 40 °C in an oil bath. The workup was performed following the general procedure. The crude material was purified by flash chromatography on silica gel using 20:1 hexanes/ethyl acetate as eluent to yield the product **2o** (61 mg, 59%) as an orange-red solid. The reaction also was performed following General Procedure F with 4-(1-(2-(furan-2-yl)-1,3-dithian-2-yl)-3-phenylprop-2-yn-1-yl)morpholine (**1o**) (1.00 mmol, 386 mg), KOH (80% w/w) (0.5 mmol, 34 mg) and anhydrous DMF (4.0 mL), *without using* H_2O. The reaction was stirred for 4 h at 40 °C in an oil bath. The workup was performed following the general procedure. The crude material was purified by flash chromatography on silica gel using 20:1 hexanes/ethyl acetate as eluent to yield the product **2o** (230 mg, 60%) as an orange-red solid. ^{1}H NMR (500 MHz, $CDCl_3$): δ 7.79 (d, *J*= 7.3 Hz, 2H), 7.35–7.44 (m, 4H), 6.61 (s, 1H, CH), 6.38–6.43 (m, 2H), 3.55–3.61 (m, 4H), 3.03–3.08 (m, 2H), 2.87–2.91 (m, 2H), 2.81 (dt, *J*= 4.0, 14.0 Hz, 1H), 2.46–2.54 (m, 2H), 2.32–2.38 (m, 1H), 2.06–2.12 (m, 1H), 1.73–1.81 (m, 1H) ppm; ^{13}C-APT NMR (125 MHz, $CDCl_3$): δ 154.1, 152.3, 141.8, 141.6, 139.1, 134.2, 128.7, 128.5, 127.9, 110.9, 109.4, 94.3, 67.2, 49.9, 33.1, 32.9, 31.5 ppm. HRMS [TOF MS ES+]: m/z $[M + H]^+$ calcd. for $C_{21}H_{24}NO_2S_2$ 386.1248, found 386.1241 (−1.8 ppm).

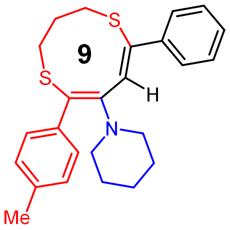

1-((6*E*,8*Z*)-9-Phenyl-6-(*p*-tolyl)-3,4-dihydro-2*H*-1,5-dithionin-7-yl)piperidine (2p): The reaction was performed following General Procedure E with 1-(3-phenyl-1-(2-(*p*-tolyl)-1,3-dithian-2-yl)prop-2-yn-1-yl)piperidine (**1p**) (0.19 mmol, 79 mg) and water (0.19 mmol, 3.4 µL). In glovebox, the reaction vial was charged with KO*t*Bu (0.095 mmol, 11.0 mg) and anhydrous DMF (0.75 mL). The reaction was stirred for 4 h at 40 °C in an oil bath. The workup was performed following the general procedure. The crude material was purified by flash chromatography on silica gel using 20:1 hexanes/ethyl acetate as eluent to yield the product **2p** (60 mg, 76%) as an orange solid. ^{1}H NMR (500

MHz, $CDCl_3$): δ 7.84 (d, *J* =7.5 Hz, 2H), 7.35–7.44 (m, 5H), 7.15 (d, *J* = 7.6 Hz, 2H), 6.71 (s, 1H), 2.82–2.84 (m, 2H), 2.74–2.77 (m, 1H), 2.64–2.66 (m, 2H), 2.31–2.41 (m, 3H), 2.37 (s, 3H), 1.99–2.04 (m, 1H), 1.79–1.84 (m, 1H), 1.36–1.44 (m, 6H) ppm; ^{13}C-APT NMR (125 MHz, $CDCl_3$): δ 151.6, 139.9, 139.6, 139.4, 136.9, 134.7, 129.9, 128.4, 128.3, 128.2, 127.9, 104.3, 51.4, 33.3, 32.7, 31.2, 26.6, 24.4, 21.2 ppm. HRMS [TOF MS ES+]: m/z $[M + H]^+$ calcd. for $C_{25}H_{30}NS_2$ 408.1820, found 408.1816 (−1.0 ppm).

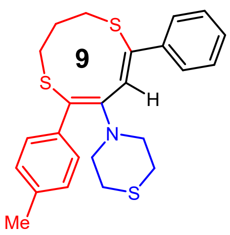

4-((6*E*,8*Z*)-9-Phenyl-6-(*p*-tolyl)-3,4-dihydro-2*H*-1,5-dithionin-7-yl)thiomorpholine (2q): The reaction was performed following General Procedure E with 4-(3-phenyl-1-(2-(*p*-tolyl)-1,3-dithian-2-yl)prop-2-yn-1-yl)thiomorpholine (**1q**) (0.25 mmol, 106 mg) and water (0.25 mmol, 4.5 µL). In glovebox, the reaction vial was charged with KOtBu (0.125 mmol, 14.0 mg) and anhydrous DMF (1.0 mL). The reaction was stirred for 4 h at 40 °C in an oil bath. The workup was performed following the general procedure. The crude material was purified by flash chromatography on silica gel using 20:1 hexanes/ethyl acetate as eluent to yield the product **2q** (82 mg, 77%) as an orange solid. ^{1}H NMR (500 MHz, $CDCl_3$): δ7.80–7.83 (m, 2H), 7.41–7.44 (m, 4H), 7.35–7.38 (m, 1H), 7.16 (d, *J* = 8.0 Hz, 2H), 6.68 (s, 1H), 2.93–2.98 (m, 2H), 3.06–3.11 (m, 2H), 2.72 (dt, *J* =4.0, 14.3 Hz, 1H), 2.38–2.45 (m, 6H), 2.37 (s, 3H), 2.28–2.34 (m, 1H), 1.97–2.04 (m, 1H), 1.79–1.87 (m, 1H) ppm; ^{13}C-APT NMR (125 MHz, $CDCl_3$): δ 151.5, 139.9, 139.7, 138.7, 136.1, 135.7, 129.9, 128.7, 128.5, 128.4, 128.0, 108.5, 52.6, 33.5, 32.4, 31.1, 28.0, 21.2 ppm. HRMS [TOF MS ES+]: m/z $[M + H]^+$ calcd. for $C_{24}H_{28}NS_3$ 426.1384, found 426.1386 (0.5 ppm).

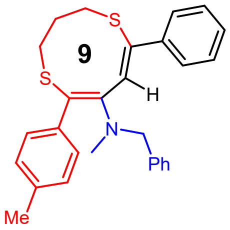

(6*E*,8*Z*)-*N*-Benzyl-*N*-methyl-9-phenyl-6-(*p*-tolyl)-3,4-dihydro-2*H*-1,5-dithionin-7-amine (2r): The reaction was performed following General Procedure E with *N*-benzyl-*N*-methyl-3-phenyl-1-(2-(*p*-tolyl)-1,3-dithian-2-yl)prop-2-yn-1-amine (**1r**) (0.29 mmol, 130 mg) and water (0.29 mmol, 5.2 µL). In glovebox, the reaction vial was charged with KO*t*Bu (0.145 mmol, 16.0 mg) and anhydrous DMF (1.2 mL). The reaction was stirred for 4 h at 40 °C in an oil bath. The workup was performed following the general procedure. The crude material was purified by flash chromatography on silica gel using 20:1 hexanes/ethyl acetate as eluent to yield the product **2r** (67 mg, 52%) as an orange solid.

^{1}H NMR (500 MHz, $CDCl_3$): δ 7.83 (d, J =7.6 Hz, 2H), 7.40–7.43 (m, 2H), 7.31–7.38 (m, 5H), 7.22–7.25 (m, 3H), 7.08 (d, J = 8.0 Hz, 2H), 6.87 (s, 1H), 3.93–4.18 (AB_{system}, $δ_A$ = 4.17, $δ_B$ = 3.95, J_{AB} = 15.0 Hz, 2H), 2.77 (dt, J =4.0, 14.2 Hz, 1H), 2.39–2.45 (m, 2H), 2.31–2.36 (m, 1H), 2.34 (s, 3H), 2.32 (s, 3H), 2.00–2.07 (m, 1H), 1.80–1.88 (m, 1H) ppm; ^{13}C-APT NMR (125 MHz, $CDCl_3$): δ 150.9, 139.9, 139.8, 139.5, 138.3, 136.6, 134.8, 130.0, 128.5, 128.4, 128.3, 128.2, 128.1, 128.0, 127.0, 103.7, 58.2, 40.2, 33.5, 32.8, 31.4, 21.2 ppm. HRMS [TOF MS ES+]: m/z $[M + H]^+$ calcd. for $C_{28}H_{30}NS_2$ 444.1820, found 444.1825 (1.1 ppm).

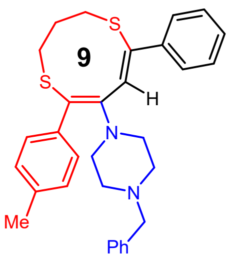

1-Benzyl-4-((6*E*,8*Z*)-9-phenyl-6-(*p*-tolyl)-3,4-dihydro-2*H*-1,5-dithionin-7-yl)piperazine (2s): The reaction was performed following General Procedure E with 1-benzyl-4-(3-phenyl-1-(2-(*p*-tolyl)-1,3-dithian-2-yl)prop-2-yn-1-yl)piperazine (**1s**) (0.25 mmol, 125 mg) and water (0.25 mmol, 4.5 μL). In glovebox, the reaction vial was charged with KOtBu (0.125 mmol, 14.0 mg) and anhydrous DMF (1.0 mL). The reaction was stirred for 4 h at 40 °C in an oil bath. The workup was performed following the general procedure. The crude material was purified by flash chromatography on silica gel using 5:1 hexanes/ethyl acetate as eluent to yield the product **2s** (94 mg, 75%) as an orange solid. ^{1}H NMR (500 MHz, $CDCl_3$): δ 7.81 (d, J =7.0 Hz, 2H), 7.38–7.42 (m, 4H), 7.34–7.37 (m, 1H), 7.19–7.28 (m, 5H), 7.11 (d, J = 8.1 Hz, 2H), 6.70 (s, 1H), 3.36–3.45 (AB_{system}, $δ_A$ = 3.43, $δ_B$ = 3.37, J_{AB} = 13.0 Hz, 2H), 2.87–2.91 (m, 2H), 2.67–2.75 (m, 3H), 2.34 (s, 3H), 2.28–2.40 (m, 3H), 2.21–2.26 (m, 4H), 1.96–2.05 (m, 1H), 1.76–1.84 (m, 1H) ppm; ^{13}C-APT NMR (125 MHz, $CDCl_3$): δ 150.6, 139.73, 139.7, 139.1, 137.7, 136.5, 130.0, 129.2, 128.5, 128.4, 128.3, 128.1, 127.9, 127.0, 105.1, 63.2, 53.5, 49.9, 33.3, 32.6, 31.2, 21.2 ppm. HRMS [TOF MS ES+]: m/z $[M + H]^+$ calcd. for $C_{31}H_{35}N_2S_2$ 499.2242, found 499.2241 (−0.2 ppm).

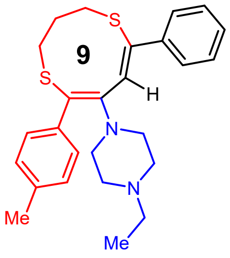

1-Ethyl-4-((6*E*,8*Z*)-9-phenyl-6-(*p*-tolyl)-3,4-dihydro-2*H*-1,5-dithionin-7-yl)piperazine (2t): The reaction was performed following General Procedure E with 1-ethyl-4-(3-phenyl-1-(2-(*p*-tolyl)-1,3-dithian-2-yl)prop-2-yn-1-yl)piperazine (**1t**) (0.21 mmol, 91.0 mg) and water (0.21 mmol, 3.8 μL). In glovebox, the reaction vial was charged with KOtBu (0.11 mmol, 12.0 mg) and anhydrous

DMF (1.0 mL). The reaction was stirred for 4 h at 40 ºC in an oil bath. The workup was performed following the general procedure. The crude material was purified by flash chromatography on silica gel using 5:1 hexanes/ethyl acetate as eluent to yield the product **2t** (68 mg, 75%) as an orange solid. ^{1}H NMR (500 MHz, $CDCl_3$): δ 7.82 (d, *J* =7.0 Hz, 2H), 7.40–7.43 (m, 4H), 7.34–7.37 (m, 1H), 7.12 (d, *J* = 8.0 Hz, 2H), 6.71 (s, 1H), 2.92–2.96 (m, 2H), 2.69–2.75 (m, 3H), 2.30–2.41 (m, 5H), 2.34 (s, 3H), 2.24 (t, *J* = 5.0 Hz, 4H), 1.97–2.05 (m, 1H), 1.77–1.85 (m, 1H), 1.00 (t, *J* = 7.2 Hz, 3H) ppm; ^{13}C-APT NMR (125 MHz, $CDCl_3$): δ 150.6, 139.8, 139.7, 139.0, 136.5, 135.2, 130.0, 128.5, 128.4, 128.3, 128.0, 105.6, 53.3, 52.4, 49.9, 33.4, 32.6, 31.2, 21.2, 11.8 ppm. HRMS [TOF MS ES+]: m/z $[M + H]^+$ calcd. for $C_{26}H_{33}N_2S_2$ 437.2085, found 437.2073 (−2.7 ppm).

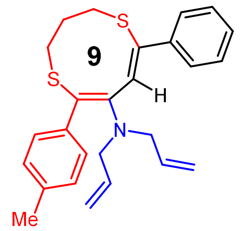

(6*E*,8*Z*)-*N,N*-Diallyl-9-phenyl-6-(*p*-tolyl)-3,4-dihydro-2*H*-1,5-dithionin-7-amine (2u): The reaction was performed following General Procedure E with *N*-allyl-*N*-(3-phenyl-1-(2-(*p*-tolyl)-1,3-dithian-2-yl)prop-2-yn-1-yl)prop-2-en-1-amine (**1u**) (0.24 mmol, 100 mg) and water (0.24 mmol, 4.3 µL). In glovebox, the reaction vial was charged with KOtBu (0.12 mmol, 13.0 mg) and anhydrous DMF (1.0 mL). The reaction was stirred for 4 h at 40 ºC in an oil bath. The workup was performed following the general procedure. The crude material was purified by flash chromatography on silica gel using 20:1 hexanes/ethyl acetate as eluent to yield the product **2u** (63 mg, 63%) as an orange solid. ^{1}H NMR (500 MHz, $CDCl_3$): δ 7.80–7.83 (m, 2H), 7.49 (d, *J* = 8.1 Hz, 2H), 7.40–7.44 (m, 2H), 7.33–7.37 (m, 1H), 7.14 (d, *J* = 8.0 Hz, 2H), 6.76 (s, 1H), 5.66–5.74 (m, 2H), 5.08 (dd, *J* = 1.7, 10.0 Hz, 2H), 4.99 (dd, *J* = 1.7, 17.0 Hz, 2H), 3.47–3.51 (m, 2H), 3.24–3.28 (m, 2H), 2.72 (dt, *J* =4.1, 14.1 Hz, 1H), 2.39–2.45 (m, 2H), 2.36 (s, 3H), 2.26–2.31 (m, 1H), 1.97–2.04 (m, 1H), 1.80–1.88 (m, 1H), ppm; ^{13}C-APT NMR (125 MHz, $CDCl_3$): δ 21.2, 31.4, 32.6, 33.5, 53.5, 105.4, 117.1, 128.0, 128.3, 128.4, 128.6, 129.9, 135.1, 135.11, 137.1, 139.1, 139.2, 139.9, 149.5 ppm. HRMS [TOF MS ES+]: m/z $[M + H]^+$ calcd. for $C_{26}H_{30}NS_2$ 420.1820, found 420.1819 (−0.2 ppm).

10. Synthesis of 8-membered *S,S*-heterocyles (2v–y) via rearrangement of 1-(1,3-dithian-2-yl)propargylamines (1v–y)

10.1. General procedure G: An oven-dried 15 mL screw-cap reaction vial equipped with a stirring bar was charged with 1-(1,3-dithian-2-yl)propargylamine derivative (1.0 equiv.) and H_2O (1.0 equiv.) by a micropipette and then the vial was brought into a glovebox. The reaction vial was charged with KO*t*Bu (1.0 equiv.) and anhydrous DMF. The vial was tightly closed, wrapped with a strip of Parafilm, and taken out of the glovebox. The reaction mixture was stirred for the given time at the stated temperature. The resulting solution was directly loaded onto a column and purified by flash chromatography on silica gel using hexanes/ethyl acetate mixture as eluent to yield the product.

10.2. Optimization of reaction conditions for product 2v:

Table S5. Optimization of reaction conditions for product **2v**

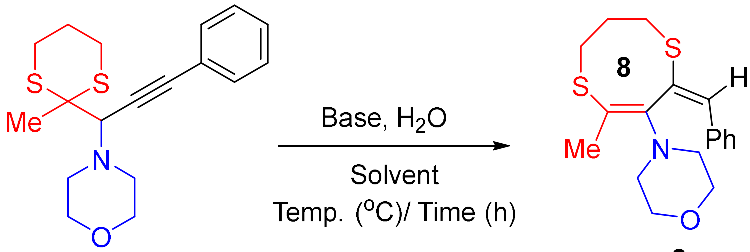

1v **2v**

Entry	A^3(mmol) **1v**	Base (KO*t*Bu mmol/M)	H_2O (equiv.)	Temp. (°C)/ Time (h)	Solvent (DMF/mL)	Yield **2v** (%)
1	0.25	0.5	1	40°C/4 h	1	trace
2	0.25	1	1	40°C/4 h	1	%38
3	0.25	1	1	0°C/18 h	1	trace
4	0.25	1	1	24 °C/18 h	1	trace
5	0.25	1	2	40°C/4 h	1	trace
6	0.25	0.5	1	70°C/3h	1	trace
7	0.23	1	1	70°C/2 h	1 (DMSO)	%44
8	0.25	1	1	40°C/18 h	1	%25
9	0.25	1	1	70°C/3 h	1	%24

10.3. Synthesis of 8-membered *S,S*-heterocyles (2v–y):

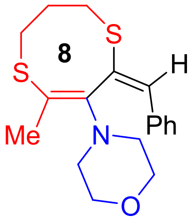

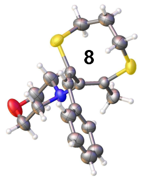

2v (X-ray)
CCDC: 1960461

4-((*E*)-2-((*E*)-Benzylidene)-4-methyl-7,8-dihydro-2*H*,6*H*-1,5-dithiocin-3-yl)morpholine (2v): The reaction was performed following General Procedure G with 4-(1-(2-methyl-1,3-dithian-2-yl)-3-phenylprop-2-yn-1-yl)morpholine (**1v**) (0.25 mmol, 83 mg) and water (0.25 mmol, 4.5 µL). In glovebox, the reaction vial was charged with KO*t*Bu (0.125 mmol, 14.0 mg) and anhydrous DMF (1.0 mL). The reaction was stirred for 4 h at 40 ºC in an oil bath. The workup was performed following the general procedure. The crude material was purified by flash chromatography on silica gel using 20:1 hexanes/ethyl acetate as eluent to yield the product **2v** (32 mg, 38%) as a white solid. ^{1}H NMR (500 MHz, $CDCl_3$): δ 7.40–7.42 (m, 2H), 7.27–7.30 (m, 2H), 7.18–7.21 (m, 1H), 6.95 (s, 1H, CH), 3.60–3.66 (m, 4H), 3.00–3.06 (m, 1H), 2.85–2.90 (m, 2H), 2.75–2.79 (m, 2H), 2.60–2.65 (m, 1H), 2.40–2.47 (m, 2H), 2.21–2.29 (m, 1H), 2.17 (s, 3H), ppm; ^{13}C-APT NMR (125 MHz, $CDCl_3$): δ 139.8, 136.5, 134.2, 130.4, 128.5, 128.1, 127.3, 121.4, 67.4, 50.2, 37.4, 31.1, 29.6, 20.4 ppm. HRMS [TOF MS ES+]: m/z $[M + H]^+$ calcd. for $C_{18}H_{24}NOS_2$ 334.1299, found 334.1299 (0.0 ppm).

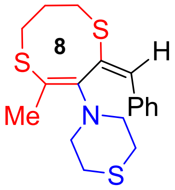

4-((*E*)-2-((*E*)-benzylidene)-4-methyl-7,8-dihydro-2*H*,6*H*-1,5-dithiocin-3-yl)thiomorpholine (2y): The reaction was performed following General Procedure G with 4-(1-(2-methyl-1,3-dithian-2-yl)-3-phenylprop-2-yn-1-yl)morpholine (**1y**) (0.30 mmol, 105 mg) and water (0.30 mmol, 5.4 µL). In glovebox, the reaction vial was charged with KOtBu (0.15 mmol, 17.0 mg) and anhydrous DMF (1.0 mL). The reaction was stirred for 4 h at 40 ºC in an oil bath. The workup was performed following the general procedure. The crude material was purified by flash chromatography on silica gel using 20:1 hexanes/ethyl acetate as eluent to yield the product **2y** (40 mg, 38%) as a white solid. ^{1}H NMR (500 MHz, $CDCl_3$): δ 7.38–7.40 (m, 2H), 7.27–7.30 (m, 2H), 7.19–7.22 (m, 1H), 6.95 (s, 1H), 3.54–3.59 (m, 1H), 3.06–3.08 (m, 4H), 2.97–3.02 (m, 1H), 2.57–2.65 (m, 5H), 2.39–2.48 (m, 2H), 2.19–2.27 (m, 1H), 2.13 (s, 3H), ppm; ^{13}C-APT NMR (125 MHz, $CDCl_3$): δ 141.3, 136.5, 134.5, 131.2, 128.5, 128.1, 127.4, 120.8, 52.5, 37.2, 31.3, 29.7,

28.4, 20.6 ppm. HRMS [TOF MS ES+]: m/z $[M + H]^+$ calcd. for $C_{18}H_{24}NS_3$ 350.1071, found 350.1074 (0.9 ppm).

Table S6. Non-reactive substrates

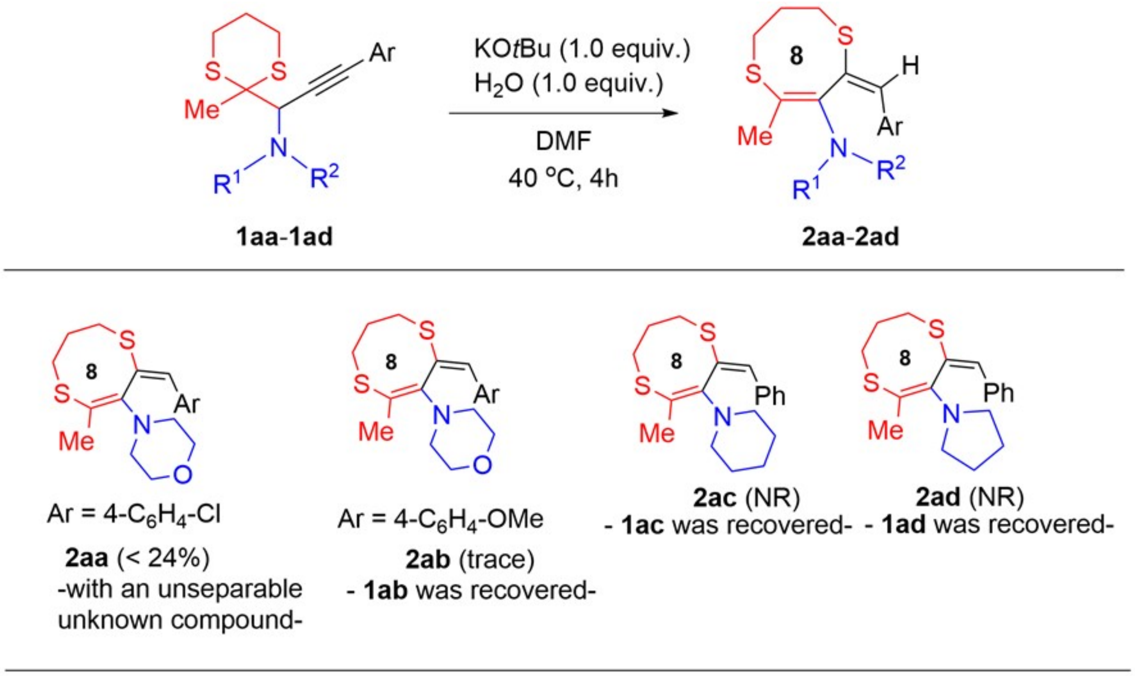

11. Synthesis of 8-membered *S,S*-heterocycles (5a–e) via rearrangement of 1-(1,3-dithiolan-2-yl)propargylamines (4a–e)

11.1. General procedure H: An oven-dried 15 mL screw-cap reaction vial equipped with a stirring bar was charged with 1-(1,3-dithiolan-2-yl)propargylamine derivative (1.0 equiv.) and H_2O (1.0 equiv.) by a micropipette and then the vial was brought into a glovebox. The reaction vial was charged with anhydrous DMF and tightly closed with a rubber septum by wrapping with a strip of Parafilm. Another oven-dried 4.0 mL vial having polypropylene cap with PTFE/Silicone Septa was charged with KO*t*Bu (2.0 equiv.) and anhydrous DMF. The vial was tightly closed, wrapped with a strip of Parafilm. Both vials were taken out of the glovebox. The KO*t*Bu solution in anhydrous DMF was added portionwise by syringe through the rubber septum in 2 h to DMF solution of 1-(1,3-dithiolan-2-yl)propargylamine and H_2O at 0 °C. The resulting reaction mixture was stirred for 18 h at 24 °C and then directly loaded onto a column and purified by flash chromatography on silica gel using hexanes/ethyl acetate mixture as eluent to yield the product.

Table S7. Optimization of reaction conditions for product **5a**

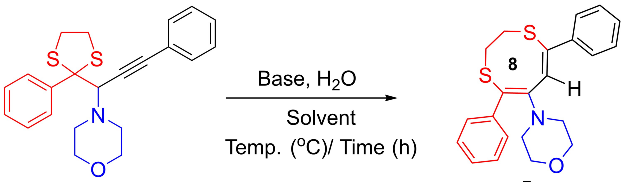

4a **5a**

Entry	A[3](mmol)	Base (KO*t*Bu mmol/M)	H_2O (equiv.)	Temp. (°C)/ Time (h)	Solvent (DMF/mL)	Yield (%)
1	0.5	0.5 mmol	1	40 °C /4 h	2	-
2	0.5	0.25 mmol	1	40 °C /4 h	2	-
3	0.5	0.5 mmol	1	40 °C /16 h	2	27%
4	0.25	0.25 mmol	1	40 °C /24 h	1	16%
5	0.25	0.25 mmol	1	40 °C /16 h	1 (DMSO)	16%
6	0.5	0.5 mmol	1	50 °C /8 h	2	26%
7	0.5	0.5 mmol	1	50 °C /16 h	2	32%
8	0.25	0.25 mmol	1	70 °C /4 h	1	14%
9	0.25	0.25 mmol	2	70 °C /6 h	1	16%
10	0.25	0.125 mmol	1	100 °C /4 h	1	24%
11	0.25	0.125 M, 1.0 mL	1	40 °C/2 h; then 40 °C, 18 h	2	22%
12	0.25	0.25 M, 1.0 mL	1	24 °C/2 h; then 40 °C, 18 h	2	36%
13	0.25	0.25 M, 1.0 mL	1	40 °C/2 h; then 40 °C, 18 h	2	45%
14	0.25	0.125 M, 1.0 mL	-	40 °C/2 h; then 40 °C, 18 h	2	-
15	0.25	0.25 M, 1.0 mL	2	40 °C/2 h; then 40 °C, 18 h	2	25%
16	0.25	0.125 M, 1.0 mL	1	0 °C/2 h; then 24 °C, 18 h	2	20%
17	0.25	0.25 M, 1.0 mL	1	0 °C/2 h; then 24 °C, 18 h	2	49%
18	0.25	0.38 M, 1.0 mL	1	0 °C/2 h; then 24 °C, 18 h	2	52%
**19**	**0.25**	**0.33 M, 1.5 mL**	**1**	**0 °C/2 h; then 24 °C, 18 h**	**2.5**	**58%**
20	0.25	0.5 M, 1.5 mL	1	0 °C/2 h; then 24 °C, 18 h	2	48%
21	0.25	0.4 M, 1.25 mL	1	0 °C/2 h; then 40 °C, 18 h	2	54%
22	0.25	0.5 M, 1.0 mL (KHMDS)	1	0 °C/2 h; then 40 °C, 18 h	2	41%
23	0.25	0.25 M, 1.0 mL (KHMDS)	1	0 °C/2 h; then 40 °C, 18 h	2	46%

11.2. Synthesis of 8-membered *S,S*-heterocyles (5a–e):

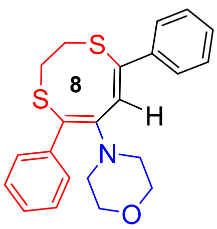

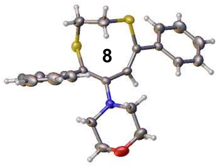

5a (X-ray)
CCDC: 2172067

4-((5*E*,7*Z*)-5,8-diphenyl-2,3-dihydro-1,4-dithiocin-6-yl)morpholine (5a): The reaction was performed following General Procedure H with 4-(3-phenyl-1-(2-phenyl-1,3-dithiolan-2-yl)prop-2-yn-1-yl)morpholine (**4a**) (0.25 mmol, 95 mg) and water (0.25 mmol, 4.5 µL). In glovebox, the reaction vial was charged with anhydrous DMF (1.0 mL). The solution of KO*t*Bu (0.50 mmol, 56 mg) in DMF (1.5 mL) was prepared in a 4.0 mL vial. The KO*t*Bu solution was added in 2 h to the solution of **4a** and H_2O at 0 °C as described in the general procedure. The resulting reaction mixture was stirred for 18 h at 24 °C and then loaded onto a column and purified by flash chromatography on silica gel using 20:1 hexanes/ethyl acetate as eluent to yield the product **5a** (55mg, 58%) as a yellow solid. ^{1}H NMR (500 MHz, $CDCl_3$): δ 7.79 (d, J = 7.3 Hz, 2H), 7.50 (d, J = 6.7 Hz, 2H), 7.40–7.42 (m, 2H), 7.33–7.37 (m, 2H), 7.19 (t, J = 7.3 Hz, 2H), 6.83 (s, 1H), 3.48–3.53 (m, 4H), 2.88– 3.01 (m, 3H), 2.71–2.75 (m, 2H), 2.46– 2.51 (m, 1H), 2.34–2.42 (m, 2H) ppm; ^{13}C-APT NMR (125 MHz, $CDCl_3$): δ 153.1, 142.1, 141.0, 138.5, 135.1, 130.6, 128.5, 128.3, 128.2, 127.9, 126.2, 105.4, 67.1, 51.0, 28.1, 26.9 ppm. HRMS [TOF MS ES+]: m/z $[M + H]^+$ calcd. for $C_{22}H_{24}NOS_2$ 382.1299, found 382.1308 (2.4 ppm).

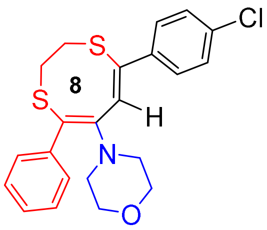

4-((5*E*,7*Z*)-8-(4-chlorophenyl)-5-phenyl-2,3-dihydro-1,4-dithiocin-6-yl)morpholine (5b): The reaction was performed following General Procedure H with 4-(3-(4-chlorophenyl)-1-(2-phenyl-1,3-dithiolan-2-yl)prop-2-yn-1-yl)morpholine (**4b**) (0.21 mmol, 89 mg) and water (0.21 mmol, 3.9 µL). In glovebox, the reaction vial was charged with anhydrous DMF (1.0 mL). The solution of KO*t*Bu (0.42 mmol, 47 mg) in DMF (1.25 mL) was prepared in a 4.0 mL vial. The KO*t*Bu solution was added in 2 h to the solution of **4b** and H_2O at 0 °C as described in the general procedure. The resulting reaction mixture was stirred for 18 h at 24 °C and then loaded onto a column and purified by flash chromatography on silica gel using 20:1 hexanes/ethyl acetate as eluent to yield the product **5b** (67 mg, 75%) as a yellow solid. ^{1}H NMR (500 MHz, $CDCl_3$): δ 7.72 (d, J = 8.5 Hz, 2H), 7.48 (d, J = 6.8 Hz, 2H), 7.33–7.38 (m, 4H), 7.19 (t, J = 7.4 Hz, 1H), 6.82 (s, 1H), 3.48–3.53 (m, 4H), 2.85–2.96

(m, 3H), 2.69–2.74 (m, 2H), 2.45–2.51 (m, 1H), 2.30–2.40 (m, 2H) ppm; ^{13}C-APT NMR (125 MHz, $CDCl_3$): δ 152.9, 141.9, 139.4, 137.2, 135.6, 134.4, 130.6, 129.5, 128.5, 127.9, 126.3, 105.8, 67.1, 51.0, 28.0, 26.7 ppm. HRMS [TOF MS ES+]: m/z $[M + H]^+$ calcd. for $C_{22}H_{23}NOS_2Cl$ 416.0910, found 416.0920 (2.4 ppm).

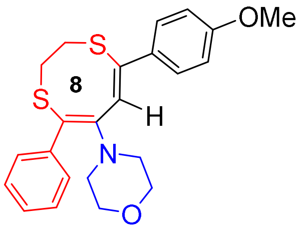

4-((5*E*,7*Z*)-8-(4-methoxyphenyl)-5-phenyl-2,3-dihydro-1,4-dithiocin-6-yl)morpholine (5c): The reaction was performed following General Procedure H with 4-(3-(4-methoxyphenyl)-1-(2-phenyl-1,3-dithiolan-2-yl)prop-2-yn-1-yl)morpholine (**4c**) (0.165 mmol, 68 mg) and water (0.165 mmol, 3.0 µL). In glovebox, the reaction vial was charged with anhydrous DMF (0.5 mL). The solution of KO*t*Bu (0.33 mmol, 37 mg) in DMF (1.0 mL) was prepared in a 4.0 mL vial. The KO*t*Bu solution was added in 2 h to the solution of **4c** and H_2O at 0 °C as described in the general procedure. The resulting reaction mixture was stirred for 18 h at 24 °C and then loaded onto a column and purified by flash chromatography on silica gel using 20:1 hexanes/ethyl acetate as eluent to yield the product **5c** (38 mg, 56%) as a yellow solid. ^{1}H NMR (500 MHz, $CDCl_3$): δ 7.73–7.76 (m, 2H), 7.48–7.50 (m, 2H), 7.32–7.34 (m, 2H), 7.16–7.20 (m, 1H), 6.92–6.95 (m, 2H), 6.76 (s, 1H), 3.85 (s, 3H), 3.49–3.51 (m, 4H), 2.88–2.94 (m, 3H), 2.70–2.74 (m, 2H), 2.45–2.49 (m, 1H), 2.32–2.40 (m, 2H) ppm; ^{13}C-APT NMR (125 MHz, $CDCl_3$): δ 160.0, 153.5, 142.2, 138.0, 133.6, 133.3, 130.6, 129.6, 127.9, 126.1, 113.7, 104.9, 67.2, 55.4, 51.1, 28.1, 26.8 ppm. HRMS [TOF MS ES+]: m/z $[M + H]^+$ calcd. for $C_{23}H_{26}NO_2S_2$ 412.1405, found 412.1416 (2.7 ppm).

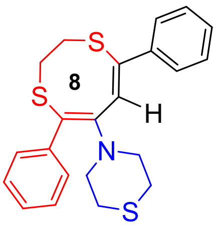

4-((5*E*,7*Z*)-5,8-diphenyl-2,3-dihydro-1,4-dithiocin-6-yl)thiomorpholine (5d): The reaction was performed following General Procedure H with 4-(3-phenyl-1-(2-phenyl-1,3-dithiolan-2-yl)prop-2-yn-1-yl)thiomorpholine (**4d**) (0.25 mmol, 99 mg) and water (0.25 mmol, 4.5 µL). In glovebox, the reaction vial was charged with anhydrous DMF (1.0 mL). The solution of KO*t*Bu (0.50 mmol, 56 mg) in DMF (1.25 mL) was prepared in a 4.0 mL vial. The KO*t*Bu solution was added in 2 h to the solution of **4d** and H_2O at 0 °C as described in the general procedure. The resulting reaction mixture was stirred for 18 h at 24 °C and then loaded onto a column and purified by flash chromatography on silica gel using 20:1

hexanes/ethyl acetate as eluent to yield the product **5d** (65mg, 65%) as a yellow solid. ^{1}H NMR (500 MHz, $CDCl_3$): δ 7.78 (d, *J* = 7.9 Hz, 2H), 7.48 (d, *J* = 7.8 Hz, 2H), 7.34–7.43 (m, 5H), 7.20 (t, *J* = 7.4 Hz, 1H), 6.79 (s, 1H), 2.97–3.12 (m, 5H), 2.33–2.51 (m, 7H) ppm; ^{13}C-APT NMR (125 MHz, $CDCl_3$): δ 154.2, 142.1, 141.0, 138.4, 135.1, 130.5, 128.5, 128.3, 128.2, 128.0, 126.2, 106.8, 53.2, 27.9, 27.8, 26.8 ppm. HRMS [TOF MS ES+]: m/z $[M + H]^+$ calcd. for $C_{22}H_{24}NS_3$ 398.1071, found 398.1071 (0.0 ppm).

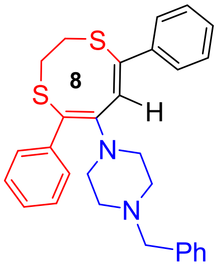

1-Benzyl-4-((5*E*,7*Z*)-5,8-diphenyl-2,3-dihydro-1,4-dithiocin-6-yl)piperazine (5e): The reaction was performed following General Procedure H with 1-benzyl-4-(3-phenyl-1-(2-phenyl-1,3-dithiolan-2-yl)prop-2-yn-1-yl)piperazine (**4e**) (0.37 mmol, 175 mg) and water (0.37 mmol, 6.6 µL). In glovebox, the reaction vial was charged with anhydrous DMF (1.2 mL). The solution of KO*t*Bu (0.74 mmol, 83 mg) in DMF (1.5 mL) was prepared in a 4.0 mL vial. The KO*t*Bu solution was added in 2 h to the solution of **4e** and H_2O at 0 °C as described in the general procedure. The resulting reaction mixture was stirred for 18 h at 24 °C and then loaded onto a column and purified by flash chromatography on silica gel using 10:1 hexanes/ethyl acetate as eluent to yield the product **5e** (80 mg, 46%) as a yellow solid. ^{1}H NMR (500 MHz, $CDCl_3$): δ7.81 (d, *J* = 7.0 Hz, 2H), 7.50 (d, *J* = 6.7 Hz, 2H), 7.43–7.45 (m, 2H), 7.24–7.41 (m, 8H), 7.18 (t, *J* = 7.4 Hz, 1H), 6.84 (s, 1H), 3.42–3.51 (AB_{system}, δ_A = 3.50, δ_B = 3.44, J_{AB} = 12.9 Hz, 2H), 2.95–3.02 (m, 3H), 2.77– 2.82 (m, 2H), 2.30–2.50 (m, 7H) ppm; ^{13}C-APT NMR (125 MHz, $CDCl_3$): δ 152.9, 142.3, 141.2, 138.1, 137.5, 135.5, 130.5, 129.2, 128.34, 128.3, 128.2, 128.1, 127.8, 127.1, 125.8, 103.9, 63.2, 53.5, 50.5, 28.1, 26.9 ppm. HRMS [TOF MS ES+]: m/z $[M + H]^+$ calcd. for $C_{29}H_{31}N_2S_2$ 471.1929, found 471.1927 (−0.4 ppm).

12. Synthesis of 10-membered *S,S*-heterocyles (7a–d) via rearrangement of 1-(1,3-dithiepan-2-yl)propargylamines (6a-d)

12.1. General procedure I: An oven-dried 15 mL screw-cap reaction vial equipped with a stirring bar was charged with 1-(1,3-dithiepan-2-yl)propargylamine derivative (1.0 equiv.) and H_2O (1.0 equiv.) by a micropipette and then the vial was brought into a glovebox. The reaction vial was charged with KO*t*Bu (0.5 equiv.) and anhydrous DMF. The vial was tightly closed, wrapped with a strip of

Parafilm, and taken out of the glovebox. The reaction mixture was stirred for the given time at the stated temperature. The resulting solution was directly loaded onto a column and purified by flash chromatography on silica gel using hexanes/ethyl acetate mixture as eluent to yield the product.

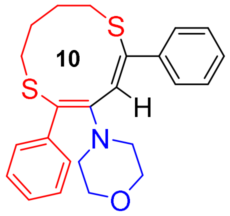

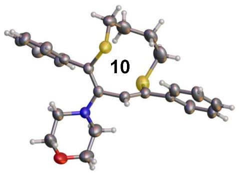

7a (X-ray)
CCDC:2183868

4-((2*E*,4*Z*)-2,5-Diphenyl-7,8,9,10-tetrahydro-1,6-dithiecin-3-yl)morpholine (7a): The reaction was performed following General Procedure I with 4-(3-phenyl-1-(2-phenyl-1,3-dithiepan-2-yl)prop-2-yn-1-yl)morpholine (**6a**) (0.25 mmol, 102 mg) and water (0.25 mmol, 4.5 µL). In glovebox, the reaction vial was charged with KO*t*Bu (0.125 mmol, 14.0 mg) and anhydrous DMF (1.5 mL). The reaction was stirred for 18 h at 40 °C in an oil bath. The workup was performed following the general procedure. The crude material was purified by flash chromatography on silica gel using 20:1 hexanes/ethyl acetate as eluent to yield the product **7a** (93 mg, 91%) as a yellow solid. ^{1}H NMR (500 MHz, $CDCl_3$): δ 7.84 (d, *J* = 7.4 Hz, 2H), 7.60 (d, *J* = 6.8 Hz, 2H), 7.43–7.46 (m, 2H), 7.35–7.40 (m, 3H), 7.20 (t, *J* = 7.3 Hz, 1H), 6.66 (s, 1H), 3.42–3.50 (m, 4H), 2.87–2.92 (m, 2H), 2.66–2.74 (m, 3H), 2.48 (d, *J* = 13 Hz, 1H), 2.03–2.09 (m, 1H), 1.80–1.94 (m, 2H), 1.38–1.57 (m, 3H) ppm; ^{13}C-APT NMR (125 MHz, $CDCl_3$): δ 148.5, 140.0, 138.9, 137.4, 135.1, 129.9, 128.5, 128.4, 128.1, 127.9, 126.0, 106.1, 67.3, 50.4, 31.7, 31.6, 24.9, 24.0 ppm. HRMS [TOF MS ES+]: m/z $[M + H]^+$ Calcd. for $C_{24}H_{28}NOS_2$ 410.1612, found 410.1612 (0.0 ppm).

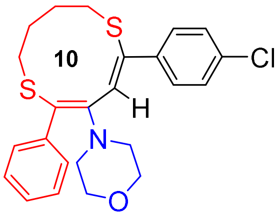

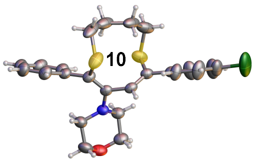

7b (X-ray)
CCDC:2214579

4-((2*E*,4*Z*)-5-(4-Chlorophenyl)-2-phenyl-7,8,9,10-tetrahydro-1,6-dithiecin-3-yl)morpholine (7b): The reaction was performed following General Procedure I with 4-(3-(4-chlorophenyl)-1-(2-phenyl-1,3-dithiepan-2-yl)prop-2-yn-1-yl)morpholine (**6b**) (0.25 mmol, 111 mg) and water (0.25 mmol, 4.5 µL). In glovebox, the reaction vial was charged with KO*t*Bu (0.125 mmol, 14.0 mg) and anhydrous DMF (1.5 mL). The reaction was stirred for 18 h at 60 °C in an oil bath. The workup was performed following the general procedure. The crude material was purified by flash chromatography on silica gel using 20:1 hexanes/ethyl acetate as eluent to

yield the product **7b** (100 mg, 90%) as a yellow solid. ^{1}H NMR (500 MHz, $CDCl_3$): δ 7.74–7.77 (m, 2H), 7.56–7.98 (m, 2H), 7.39–7.41 (m, 2H), 7.34–7.37 (m, 2H), 7.17–7.21 (m, 1H), 6.62 (s, 1H), 3.41–3.39 (m, 4H), 2.84–2.88 (m, 2H), 2.63–2.70 (m, 3H), 2.44–2.47 (m, 1H), 2.03–2.09 (m, 1H), 1.79–1.90 (m, 2H), 1.37–1.50 (m, 3H) ppm; ^{13}C-APT NMR (125 MHz, $CDCl_3$): δ 148.2, 139.8, 137.4, 136.3, 135.5, 134.2, 129.9, 129.4, 128.7, 128.0, 126.1, 106.5, 67.2, 50.3, 31.8, 31.6, 24.9, 23.9 ppm. HRMS [TOF MS ES+]: m/z $[M + H]^+$ Calcd. for $C_{24}H_{27}NOS_2Cl$ 444.1223, found 444.1222 (−0.2 ppm).

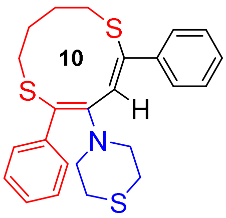

4-((2*E*,4*Z*)-2,5-diphenyl-7,8,9,10-tetrahydro-1,6-dithiecin-3-yl)thiomorpholine (7c): The reaction was performed following General Procedure I with 4-(3-phenyl-1-(2-phenyl-1,3-dithiepan-2-yl)prop-2-yn-1-yl)thio-morpholine **(6c)** (0.267 mmol, 114 mg) and water (0.267 mmol, 4.8 µL). In glovebox, the reaction vial was charged with KO*t*Bu (0.134 mmol, 15.0 mg) and anhydrous DMF (1.0 mL). The reaction was stirred for 18 h at 40 °C in an oil bath. The workup was performed following the general procedure. The crude material was purified by flash chromatography on silica gel using 20:1 hexanes/ethyl acetate as eluent to yield the product **7c** (87 mg, 76%) as a yellow solid. ^{1}H NMR (500 MHz, $CDCl_3$): δ 7.82 (d, J = 7.2 Hz, 2H), 7.57 (d, J = 6.7 Hz, 2H), 7.42–7.45 (m, 2H), 7.35–7.39 (m, 3H), 7.19 (t, J = 7.4 Hz, 1H), 6.60 (s, 1H), 2.94–3.10 (m, 4H), 2.70–2.73 (m, 1H), 2.47–2.50 (m, 1H), 2.33–2.42 (m, 4H), 2.05 (dt, J = 4.0, 13 Hz, 1H), 1.79–1.90 (m, 2H), 1.38–1.52 (m, 3H) ppm; ^{13}C-APT NMR (125 MHz, $CDCl_3$): δ 149.4, 140.1, 138.9, 137.5, 135.1, 129.8, 128.5, 128.4, 128.2, 126.1, 107.9, 52.5, 31.9, 31.6, 28.1, 25.1, 24.1 ppm. HRMS [TOF MS ES+]: m/z $[M + H]^+$ Calcd. for $C_{24}H_{28}NS_3$ 426.1384, found 426.1384 (0.0 ppm).

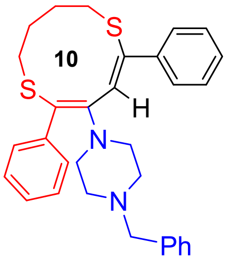

1-Benzyl-4-((2*E*,4*Z*)-2,5-diphenyl-7,8,9,10-tetrahydro-1,6-dithiecin-3-yl)piperazine (7d): The reaction was performed following General Procedure I with 1-benzyl-4-(3-phenyl-1-(2-phenyl-1,3-dithiepan-2-yl)prop-2-yn-1-yl)piperazine **(6d)** (0.34 mmol, 170 mg) and water (0.34 mmol, 6.1 µL). In glovebox, the reaction vial was charged with KO*t*Bu (0.17 mmol, 19.0 mg) and anhydrous DMF (1.5 mL). The reaction was stirred for 18 h at 40 °C in an oil bath. The workup was performed

following the general procedure. The crude material was purified by flash chromatography on silica gel using 20:1 hexanes/ethyl acetate as eluent to yield the product **7d** (99 mg, 58%) as an orange solid. ^{1}H NMR (500 MHz, $CDCl_3$): δ 7.83–7.86 (m, 2H), 7.56–7.58 (m, 2H), 7.43–7.47 (m, 2H), 7.33–7.41 (m, 3H), 7.21–7.30 (m, 5H), 7.15–7.18 (m, 1H), 6.65 (s, 1H), 3.37–3.45 (AB_{system}, δ_A = 3.43, δ_B = 3.39, J_{AB} = 13.0 Hz, 2H), 2.91–2.95 (m, 2H), 2.70–2.74 (m, 3H), 2.49 (dt, J = 3.0, 13 Hz, 1H), 2.19–2.26 (m, 4H), 2.05 (dt, J = 3.0, 13 Hz, 1H), 1.79–1.93 (m, 2H), 1.36–1.57 (m, 3H) ppm; ^{13}C-APT NMR (125 MHz, $CDCl_3$): δ 148.5, 140.3, 139.0, 137.7, 136.9, 135.5, 129.9, 129.2, 128.4, 128.3, 128.1, 127.9, 127.0, 125.6, 104.3, 63.2, 53.6, 49.8, 31.8, 31.6, 24.9, 23.9 ppm, [one CH signal belonging to a phenyl ring was not observed or co-incident]. HRMS [TOF MS ES+]: m/z $[M + H]^+$ Calcd. for $C_{31}H_{35}N_2S_2$ 499.2242, found 499.2240 (−0.4 ppm).

13. Oxidation of 9-membered ring 2a, synthesis of sulfoxide 3a.

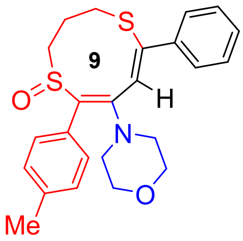

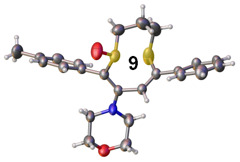

3a (X-ray)
CCDC:2206029

(6*Z*, 8*E*)-8-morpholino-6-phenyl-9-(*p*-tolyl)-3,4-dihydro-2*H*-1,5-dithionine 1-oxide (3a): A solution (DCM, 2.5 mL) of *m*-CPBA (0.50 mmol, 86 mg) was dropwise added to a solution of **2a** (0.25 mmol, 102 mg) in DCM (5 mL) at 0 °C, and then the mixture was stirred at the same temperature until the starting material was consumed (monitored by TLC, 30 min). The reaction mixture was taken up in DCM (20 mL). The solution was washed with water (2×20 mL), the aqueous phase was extracted with DCM (10 mL), and the combined organic phases were dried with anhydrous Na_2SO_4. After removal of the solvent in a rotatory evaporator, the residue was subjected to purification by chromatography on silica gel using 20:1 DCM/MeOH as eluent to yield **3a** (80 mg, 75%) as a yellow solid. ^{1}H NMR (500 MHz, $CDCl_3$): δ7.73–7.77 (m, 2H), 7.55 (d, J = 8.0 Hz, 2H), 7.40–7.45 (m, 3H), 7.21 (d, J = 8.1 Hz, 2H), 6.69 (s, 1H), 3.44–3.51 (m, 4H), 3.05 (ddd, J = 2.8, 7.1, 13.9 Hz, 1H), 2.88–2.94 (m, 4H), 2.69 (dt, J = 4.2, 18.5 Hz, 1H), 2.38 (s, 3H), 1.97–2.04 (m, 1H), 1.74–1.82 (m, 1H) ppm; ^{13}C-APT NMR (125 MHz, $CDCl_3$): δ 151.9, 146.6, 137.8, 137.5, 132.1, 130.9, 130.4, 129.7, 128.8, 128.7, 128.4, 111.8, 66.9, 52.8, 50.0, 30.8, 25.2, 21.3 ppm. HRMS [TOF MS ES+]: m/z $[M + H]^+$ calcd. for $C_{24}H_{28}NO_2S_2$ 426.1561, found 426.1563 (0.5 ppm).

14. Mechanistic Studies

14.1 Deuterium labeling experiments

a) Synthesis of D-2a:

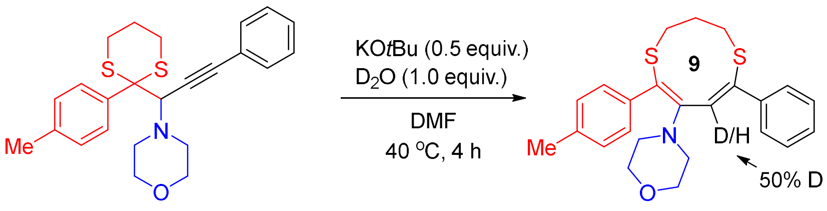

The reaction was performed following General Procedure E with 4-(3-phenyl-1-(2-(*p*-tolyl)-1,3-dithian-2-yl)prop-2-yn-1-yl)morpholine (**1a**) (0.25 mmol, 102 mg) and D_2O (0.25 mmol, 4.5 µL). In glovebox, the reaction vial was charged with KO*t*Bu (0.125 mmol, 14 mg) and anhydrous DMF (1.0 mL). The reaction was stirred for 4 h at 40 °C in an oil bath. The workup was performed following the general procedure. The crude material was purified by flash chromatography on silica gel using 20:1 hexanes/ethyl acetate as eluent to yield the product **D-2a** (56 mg, 55%) as an orange solid. ^{1}H NMR (500 MHz, $CDCl_3$): δ 7.82 (d, *J* = 7.4 Hz, 2H), 7.41–7.46 (m, 4H), 7.35–7.38 (m, 1H), 7.15 (d, *J* = 7.9 Hz, 2H), 6.72 (s, 1H, CH, integral value; 0.5, 50% less), 3.46–3.52 (m, 4H), 2.87–2.92 (m, 2H), 2.66–2.76 (m, 3H), 2.37 (s, 3H), 2.27–2.43 (m, 3H), 1.98–2.05 (m, 1H), 1.79–1.87 (m, 1H) ppm.

Figure S1. ^{1}H-NMR ($CDCl_3$, 500 MHz) spectrum of **2a**

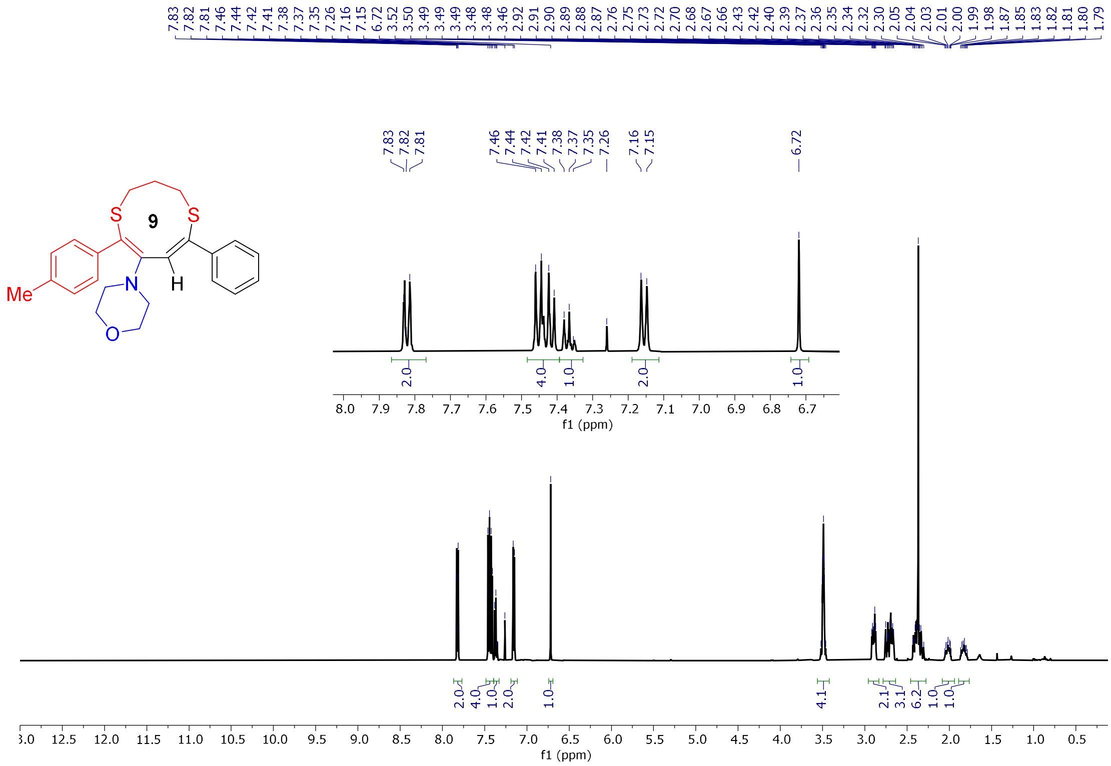

Figure S2. ^{1}H-NMR ($CDCl_3$, 500 MHz) spectrum of **D-2a**

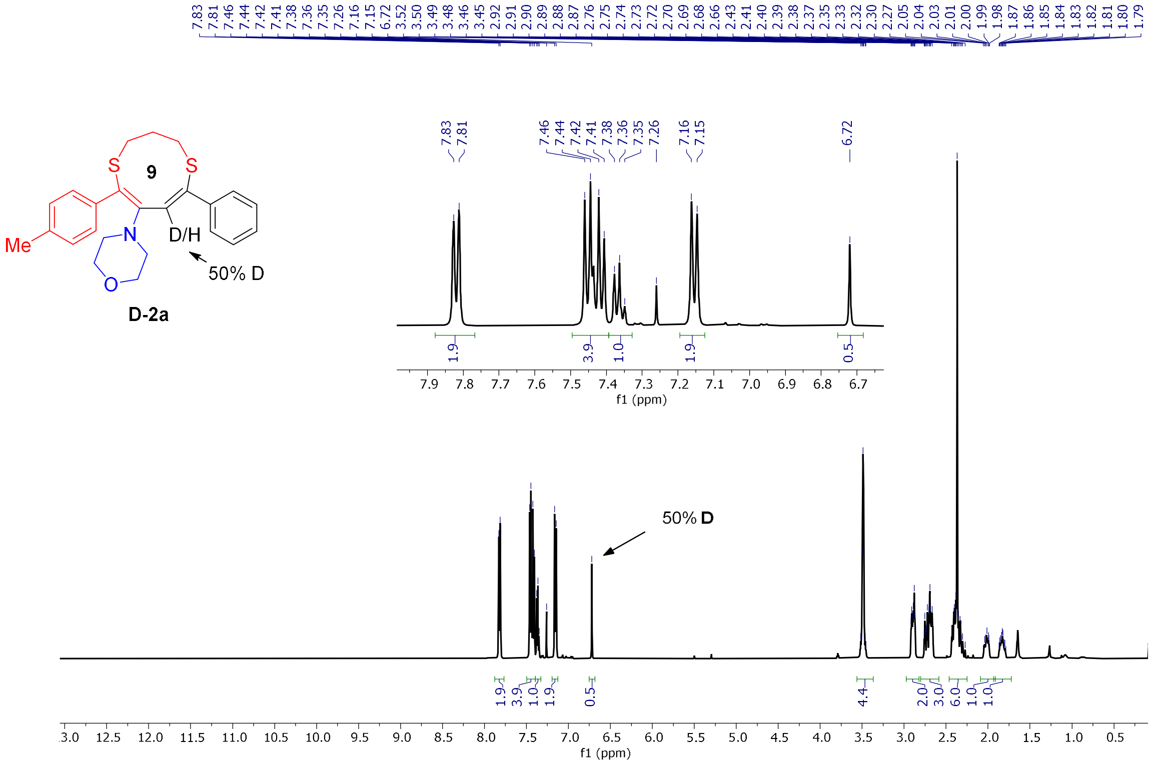

b) **Synthesis of D-2v:**

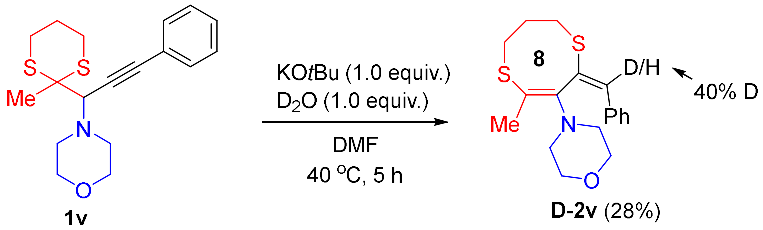

The reaction was performed following General Procedure E with 4-(1-(2-methyl-1,3-dithian-2-yl)-3-phenylprop-2-yn-1-yl)morpholine (**1v**) (0.44 mmol, 146 mg) and D_2O (0.44 mmol, 7.9 µL). In glovebox, the reaction vial was charged with KO*t*Bu (0.44 mmol, 50.0 mg) and anhydrous DMF (1.8 mL). The reaction was stirred for 5 h at 40 °C in an oil bath. The workup was performed following the general procedure. The crude material was purified by flash chromatography on silica gel using 20:1 hexanes/ethyl acetate as eluent to yield the product **D-2v** (42 mg, 28%) as a white solid. ^{1}H NMR (500 MHz, $CDCl_3$): δ 7.40–7.42 (m, 2H), 7.27–7.30 (m, 2H), 7.18– 7.21 (m, 1H), 6.95 (s, 1H, CH integral value; 0.6, 40% less), 3.60–3.65 (m, 4H), 3.00–3.06 (m, 1H), 2.85–2.90 (m, 2H), 2.74–2.79 (m, 2H), 2.60–2.65 (m, 1H), 2.40–2.47 (m, 2H), 2.21–2.29 (m, 1H), 2.16 (s, 3H), ppm.

Figure S3. ^{1}H-NMR ($CDCl_3$, 500 MHz) spectrum of **2v**

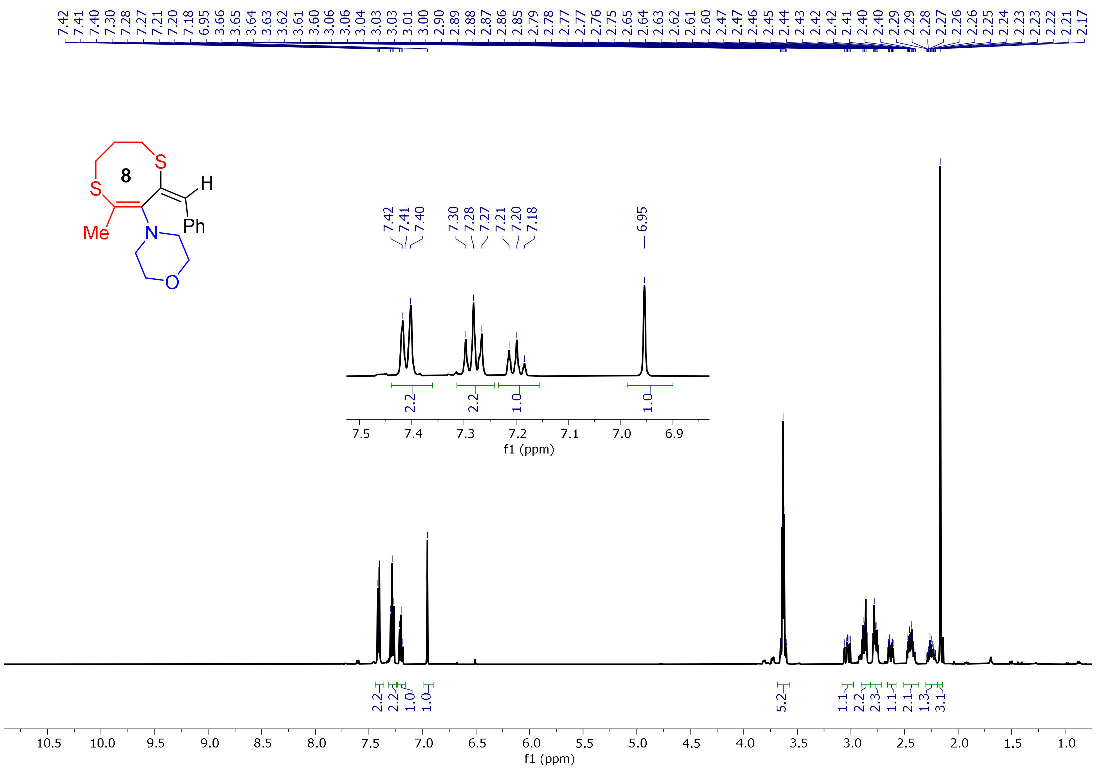

Figure S4. ^{1}H-NMR ($CDCl_3$, 500 MHz) spectrum of **D-2v**

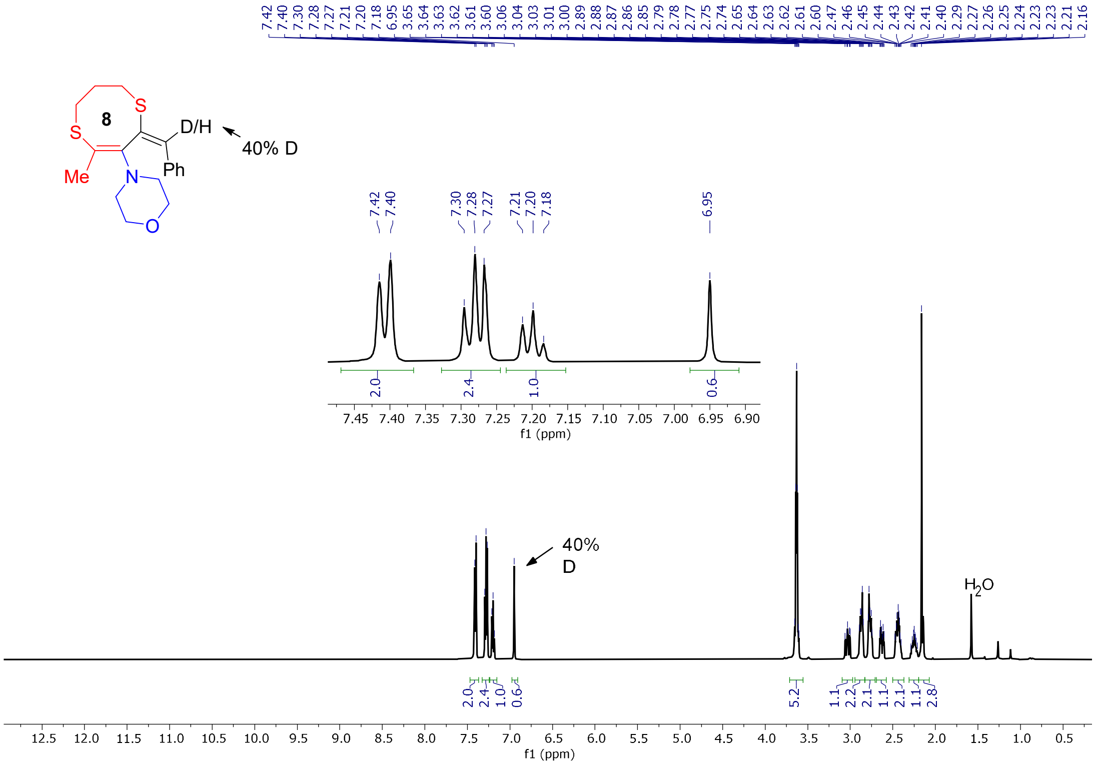

14.2 Radical Trapping Experiments

Table S8. Radical Trapping Experiments with TEMPO, DPPH, BHT and *p*-BQ

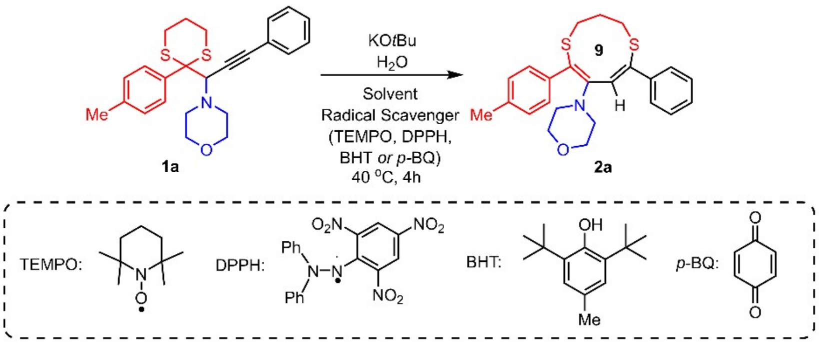

entry	solvent	KO*t*Bu (equiv.)	Radical Scavenger (equiv.)	H_2O (equiv.)	Yield **2a** (%) (isolated)	Recovery **1a** (%) (isolated)
1	DMF	0.5	-	1.0	75	-
2	DMF	0.5	TEMPO (1.5)	1.0	46	-
3	DMF	0.5	TEMPO (2.0)	1.0	36	-
4	DMF	0.5	BHT (2.0)	1.0	-	77
5	DMF	1.0	BHT (0.5)	1.0	64	-
6	DMF	0.5	DPPH (2.0)	1.0	-	46
7	DMF	1.0	DPPH (0.5)	1.0	-	76
8	DMF	0.5	DPPH (0.5)	1.0	-	80
9	DMF	0.5	DPPH (0.25)	1.0	-	59
10	DMF	0.5	DPPH (0.2)	1.0	-	83
11	DMF	0.5	DPPH (0.2)	-	-	80
12	DMF	0.5	DPPH (0.1)	1.0	27	23
13	DMA	0.5	DPPH (0.25)	1.0	-	74
14	DMF	1.0	*p*-BQ (0.5)	1.0	trace	67
15	DMF	1.0	*p*-BQ (0.5)	-	13	32
16	DMF	0.5	*p*-BQ (0.25)	1.0	-	76

a) Trapping experiment with TEMPO (2,2,6,6-Tetramethyl-1-piperidinyloxy) [Table S8, entries 2, 3]:

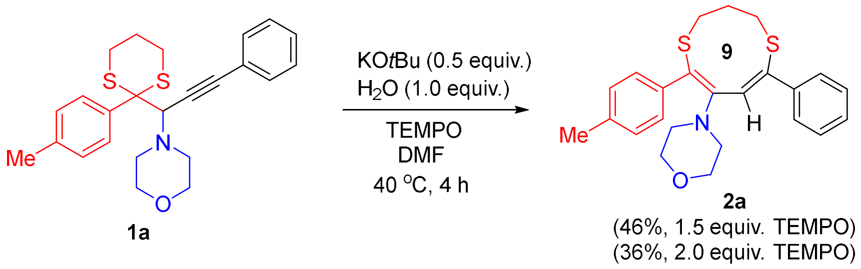

The reaction was performed following General Procedure E with 4-(3-phenyl-1-(2-(*p*-tolyl)-1,3-dithian-2-yl)prop-2-yn-1-yl)morpholine (**1a**) (0.25 mmol, 102 mg) and H_2O (0.25 mmol, 4.5 µL). In glovebox, the reaction vial was charged with KO*t*Bu (0.125 mmol, 14 mg), TEMPO (0.375 mmol, 58.6 mg *or* 0.5 mmol, 78 mg) and anhydrous DMF (1.0 mL). The reaction was stirred for 4 h at 40 °C in an oil bath. The workup was performed following the general procedure. The crude material was purified by flash chromatography on silica gel using 20:1 hexanes/ethyl acetate as eluent to yield the product **2a** (47 mg, 46% with 0.375 mmol TEMPO; 37 mg 36% with 0.5 mmol TEMPO) as an orange solid.

b) Trapping experiment with BHT (Butylhydroxytoluene) [Table S8, entry 4]:

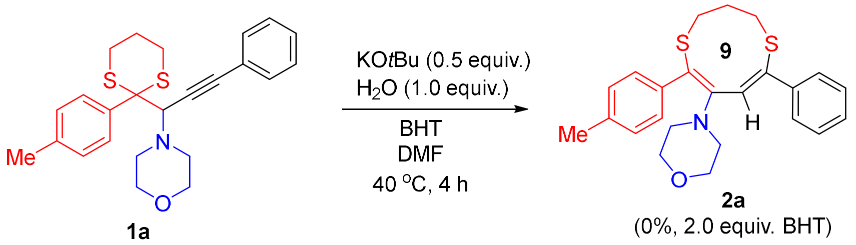

The reaction was performed following General Procedure E with 4-(3-phenyl-1-(2-(*p*-tolyl)-1,3-dithian-2-yl)prop-2-yn-1-yl)morpholine (**1a**) (0.25 mmol, 102 mg) and H_2O (0.25 mmol, 4.5 µL). In glovebox, the reaction vial was charged with KO*t*Bu (0.125 mmol, 14 mg), BHT (0.5 mmol, 110 mg) and anhydrous DMF (1.0 mL). The reaction was stirred for 4 h at 40 °C in an oil bath. In the resulting mixture, 9-membered product (**2a**) was not observed (TLC). The workup was performed following the general procedure. From the crude material **1a** was

recovered (78 mg) by flash chromatography on silica gel using 20:1 hexanes/ethyl acetate as eluent.

c) Trapping experiment with DPPH (2,2-Diphenyl-1-picrylhydrazyl) [Table S8, entry 10]:

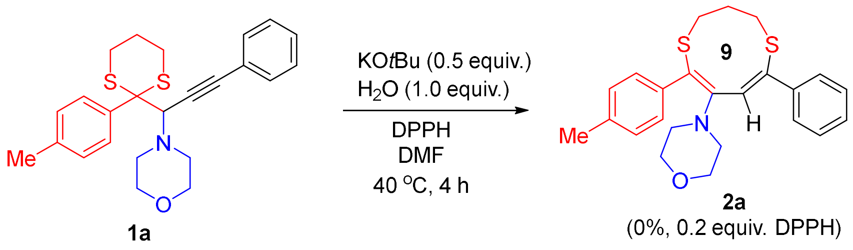

The reaction was performed following General Procedure E with 4-(3-phenyl-1-(2-(*p*-tolyl)-1,3-dithian-2-yl)prop-2-yn-1-yl)morpholine (**1a**) (0.25 mmol, 102 mg) and H_2O (0.25 mmol, 4.5 µL). In glovebox, the reaction vial was charged with KO*t*Bu (0.125 mmol, 14 mg), DPPH (0.05 mmol, 20 mg) and anhydrous DMF (1.0 mL). The reaction was stirred for 4 h at 40 °C in an oil bath. In the resulting mixture, 9-membered product (**2a**) was not observed (TLC). The workup was performed following the general procedure. From the crude material **1a** was recovered (85 mg) by flash chromatography on silica gel using 20:1 hexanes/ethyl acetate as eluent.

d) Trapping experiment with *p*-BQ (*p*-Benzoquinone) [Table S8, entry 16]:

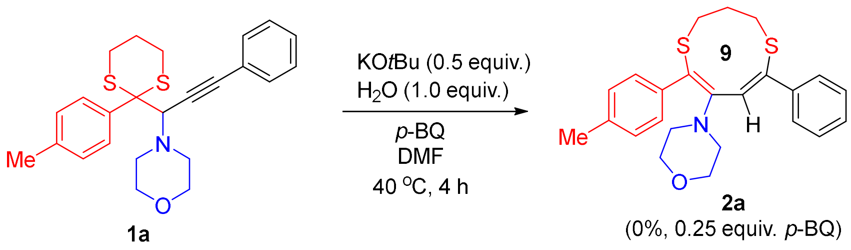

The reaction was performed following General Procedure E with 4-(3-phenyl-1-(2-(*p*-tolyl)-1,3-dithian-2-yl)prop-2-yn-1-yl)morpholine (**1a**) (0.25 mmol, 102 mg) and H_2O (0.25 mmol, 4.5 µL). In glovebox, the reaction vial was charged with KO*t*Bu (0.125 mmol, 14 mg), *p*-BQ (0.063 mmol, 7 mg) and anhydrous DMF (1.0 mL). The reaction was stirred for 4 h at 40 °C in an oil bath. In the resulting mixture, 9-membered product (**2a**) was not observed (TLC).

The workup was performed following the general procedure. From the crude material **1a** was recovered (84 mg) by flash chromatography on silica gel using 20:1 hexanes/ethyl acetate as eluent.

15. The proposed anionic mechanism

Scheme S4. The proposed anionic mechanism for the formation of 9- and 8-membered *S,S*-heterocycles

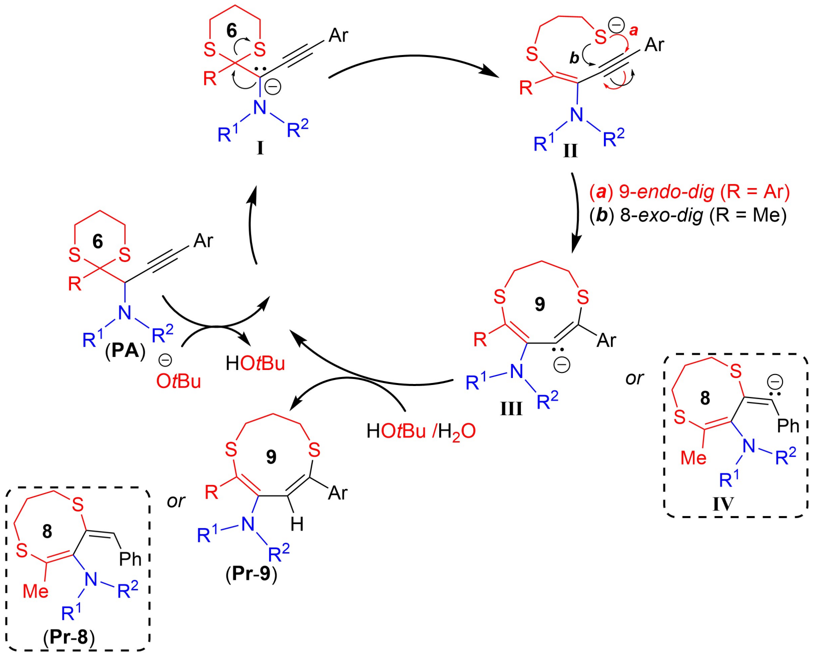

16. X-ray Crystallography Data for compound 2a–7a, 2v and 7b

Crystals of **2a**–**7a**, **2v** and **7b** were mounted on a micromount and attached to a goniometer head on a Bruker D8 VENTURE diffractometer equipped with PHOTON100 detector and was measured with graphite monochromated Mo-Kα radiation (λ = 0.71073 Å) using 1.0° of Ω and ϕ rotation frames at room temperature (297 K). The structure has been solved by intrinsic method SHELXS-1997[S3] and refined using SHELXL2014/7.[S4] Molecular drawings are generated using OLEX2. Ver. 1.2-dev.[S5]

Crystallization: Crystals of compound **2a**, **4a–7a** and **7b** were grown from the solvent chloroform, crystals of **2v** and **3a** were grown from the mixture of solvents methanol and chloroform (3:1, v/v).

Table S9. Crystal data and structure refinement for **2a** and ORTEP diagram of **2a** with ellipsoid contour at 50% probability level

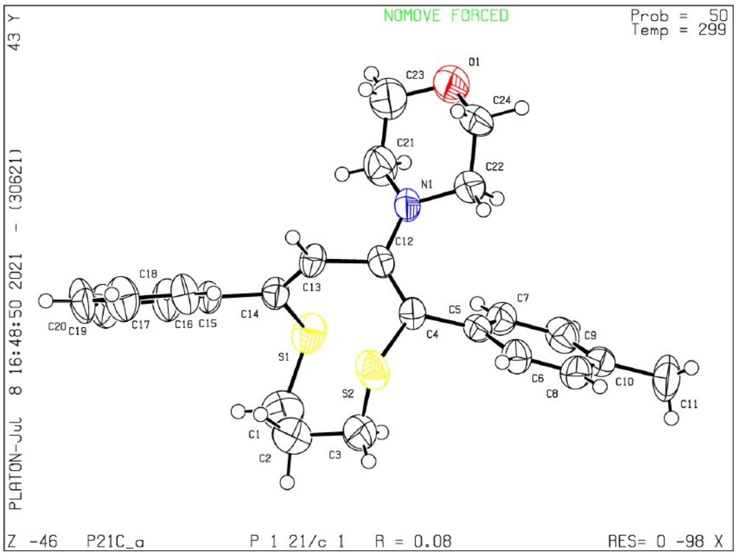

Chemical formula	$C_{24}H_{27}NOS_2$
Formula weight	409.62
Temperature/K	299.03
Crystal system	monoclinic
Space group	$P2_1/c$
a/Å	10.6570(11)
b/Å	14.5158(14)
c/Å	14.5013(14)
α/°	90
β/°	103.060(3)
γ/°	90
Volume/Å^3	2185.3(4)
Z	4
ρ_{calc}g/cm^3	1.2450
μ/mm^{-1}	0.258
F(000)	873.3
Crystal size/mm^3	0.45 × 0.13 × 0.07
Radiation	Mo Kα (λ = 0.71073)
2Θ range for data collection/°	4.82 to 50.88
Index ranges	$-12 \le h \le 12$, $-17 \le k \le 17$, $-17 \le l \le 17$
Reflections collected	53608
Independent reflections	4017 [R_{int} = 0.3147, R_{sigma} = 0.1838]
Data/restraints/parameters	4017/0/354
Goodness-of-fit on F^2	1.094
Final R indexes [I>=2σ (I)]	R_1 = 0.0844, wR_2 = 0.1038
Final R indexes [all data]	R_1 = 0.2356, wR_2 = 0.1384
Largest diff. peak/hole / e Å^{-3}	0.84/-0.93

Table S10. Crystal data and structure refinement for **2v** and ORTEP diagram of **2v** with ellipsoid contour at 50% probability level

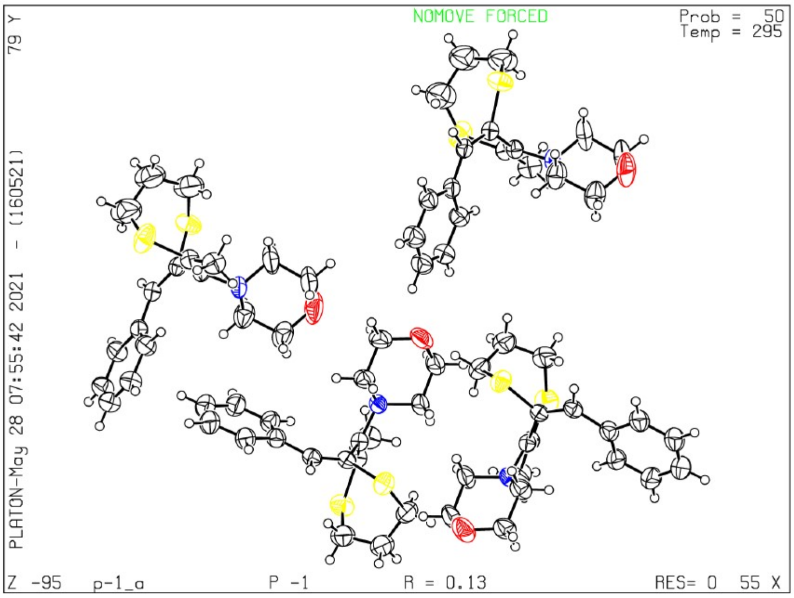

Chemical formula	$C_{18}H_{23}NOS_2$
Formula weight	333.49 g/mol
Temperature/K	295 (0)
Crystal system	triclinic
Space group	P-1
a/Å	7.528 (18)
b/Å	20.72 (5)
c/Å	23.60 (6)
α/°	73.65 (10)
β/°	89.88 (7)
γ/°	89.67(10)°
Volume/Å^3	3532.(15) Å3
Z	8
ρ_{calc} g/cm^3	1.254
μ/mm^{-1}	0.303
F(000)	1424
Crystal size/mm^3	0.020 x 0.300 x 1.000
2Θ range for data collection/°	1.99 to 25.00°
Index ranges	-8<=h<=8, -24<=k<=24, -28<=l<=28
Reflections collected	110470
Independent reflections	12400 [R(int) = 0.1164]
Data/restraints/parameters	12400 / 904 / 797
Goodness-of-fit on F^2	1.119
Final R indexes [I>=2σ (I)]	R_1 = 0.1310, wR_2 = 0.3496
Final R indexes [all data]	R_1 = 0.2101, wR_2 = 0.3948
Largest diff. peak/hole / e Å^{-3}	2.023 and -0.855

Table S11. Crystal data and structure refinement for **3a** and ORTEP diagram of **3a** with ellipsoid contour at 50% probability level

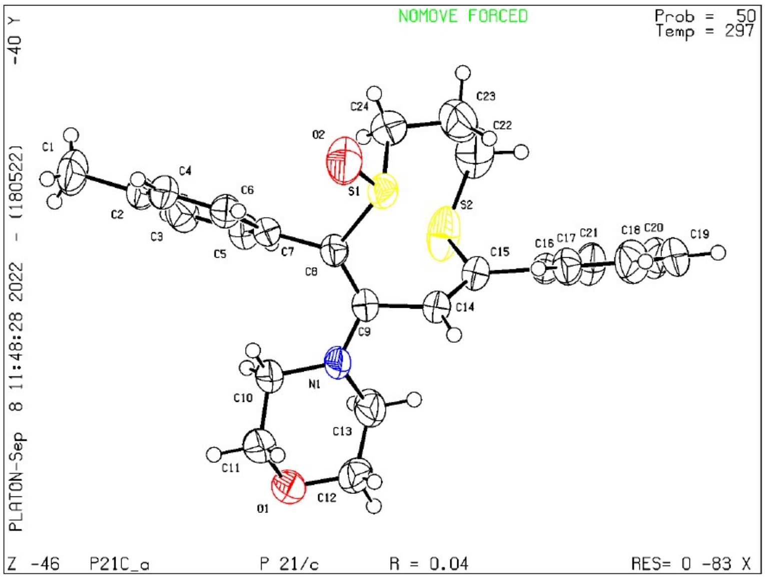

Chemical formula	$C_{24}H_{27}NO_2S_2$
Formula weight	425.58
Temperature/K	297 (0)
Crystal system	monoclinic
Space group	P 1 21/c 1
a/Å	10.9249(16)
b/Å	14.156(2)
c/Å	14.720(2)
α/°	90
β/°	102.808 (4)°
γ/°	90
Volume/Å^3	2219.8(6)
Z	4
ρ_{calc}g/cm^3	1.273
μ/mm^{-1}	0.260
F(000)	904
Crystal size/mm^3	0.050 x 0.100 x 0.400
2Θ range for data collection/°	2.39 to 25.00
Index ranges	-12<=h<=12, -16<=k<=16, -17<=l<=17
Reflections collected	87733
Independent reflections	3903 [R(int) = 0.0584]
Data/restraints/parameters	3903 / 0 / 371
Goodness-of-fit on F^2	1.040
Final R indexes [I>=2σ (I)]	R_1 = 0.0406, wR_2 = 0.0843
Final R indexes [all data]	R_1 = 0.0649, wR_2 = 0.0967
Largest diff. peak/hole / e Å^{-3}	0.264 and -0.276

Table S12. Crystal data and structure refinement for **4a** and ORTEP diagram of **4a** with ellipsoid contour at 50% probability level

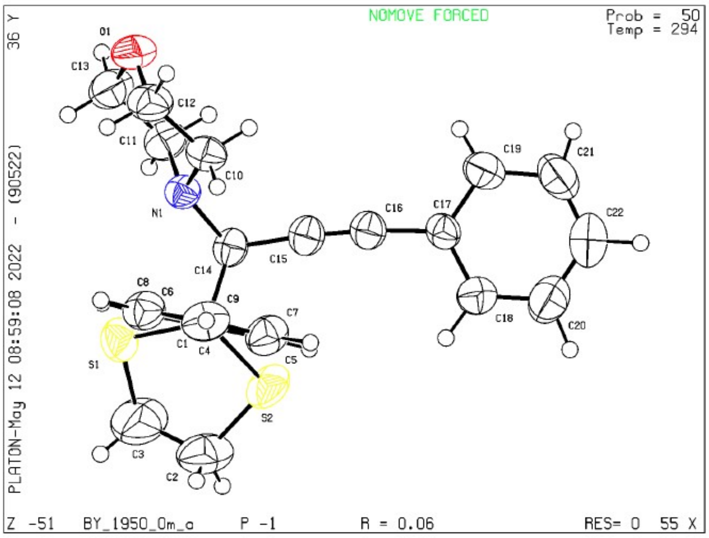

Chemical formula	$C_{22}H_{23}NOS_2$
Formula weight	381.53
Temperature/K	294 (2)
Crystal system	triclinic
Space group	P-1
a/Å	8.4448 (6)
b/Å	10.6091 (7)
c/Å	12.2623 (9)
α/°	112.112 (2)
β/°	101.696 (2)
γ/°	90.254 (2)
Volume/Å^3	992.71 (12)
Z	2
ρ_{calc}g/cm^3	1.276
μ/mm^{-1}	0.279
F(000)	404.0
Crystal size/mm^3	0.350 × 0.090 × 0.030
Radiation	MoKα (λ = 0.71073)
2Θ range for data collection/°	4.364 to 50.184
Index ranges	$-10 \leq h \leq 10$, $-12 \leq k \leq 12$, $-14 \leq l \leq 14$
Reflections collected	31684
Independent reflections	3503 [R_{int} = 0.0811, R_{sigma} = 0.0588]
Data/restraints/parameters	3503/292/311
Goodness-of-fit on F^2	1.035
Final R indexes [I>=2σ (I)]	R_1 = 0.0593, wR_2 = 0.1070
Final R indexes [all data]	R_1 = 0.1241, wR_2 = 0.1280
Largest diff. peak/hole / e Å^{-3}	0.45/-0.32

Table S13. Crystal data and structure refinement for **5a** and ORTEP diagram of **5a** with ellipsoid contour at 50% probability level

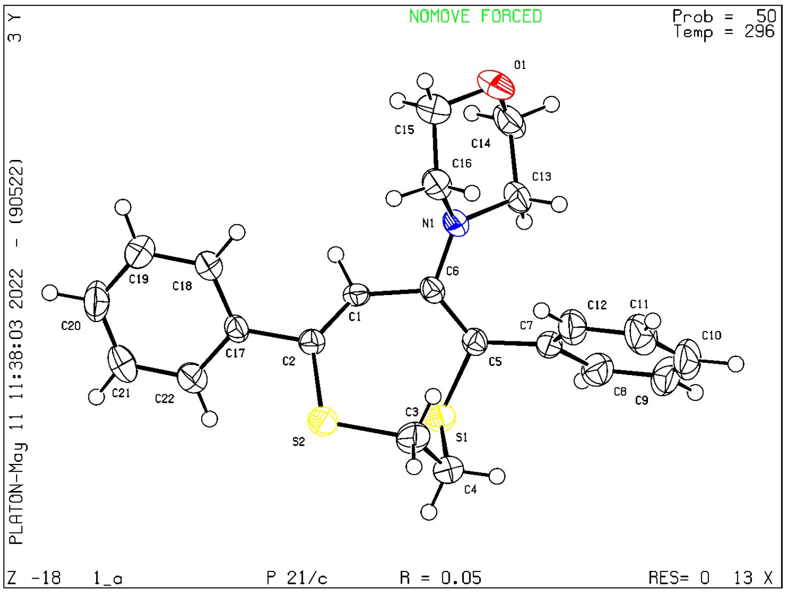

Chemical formula	$C_{22}H_{23}NOS_2$
Formula weight	381.53
Temperature/K	296 (2)
Crystal system	monoclinic
Space group	P 1 21/c 1
a/Å	12.2761(8)
b/Å	16.8325(11)
c/Å	9.9607(6)
α/°	90
β/°	109.179 (2)
γ/°	90
Volume/Å^3	1944.0 (2)
Z	4
ρ_{calc}g/cm^3	1.304
μ/mm^{-1}	0.285
F(000)	808
2Θ range for data collection/°	2.42 to 25.00°
Index ranges	-14<=h<=14, -20<=k<=20, -11<=l<=11
Reflections collected	54233
Independent reflections	3422 [R(int) = 0.1290]
Data/restraints/parameters	3422 / 0 / 328
Goodness-of-fit on F^2	1.087
Final R indexes [I>=2σ (I)]	R_1 = 0.0499, wR_2 = 0.0596
Final R indexes [all data]	R_1 = 0.1025, wR_2 = 0.0702
Largest diff. peak/hole / e Å^{-3}	0.230 and -0.283

Table S14. Crystal data and structure refinement for **6a** and ORTEP diagram of **6a** with ellipsoid contour at 50% probability level

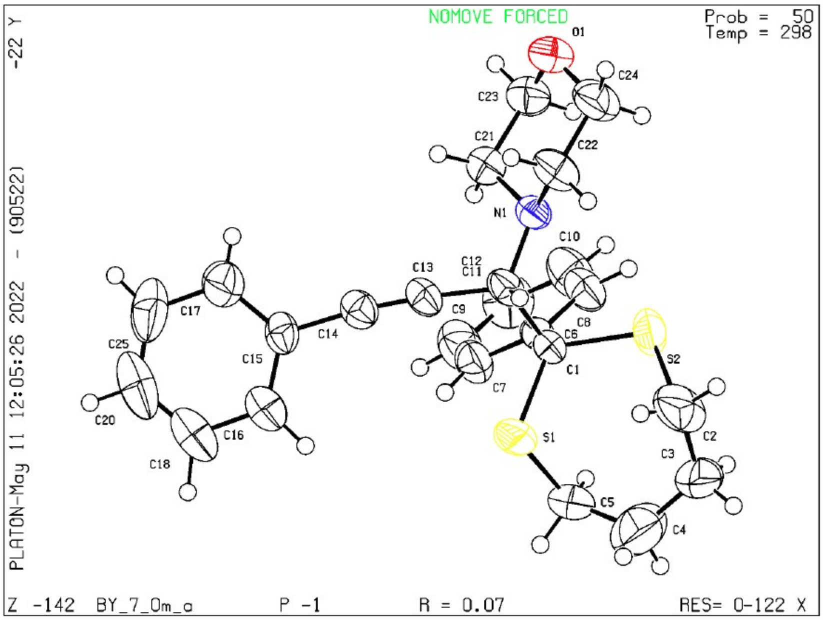

Chemical formula	$C_{24}H_{27}NOS_2$
Formula weight	409.58 g/mol
Temperature/K	298 (0)
Crystal system	triclinic
Space group	P-1
a/Å	9.314 (3)
b/Å	10.866 (4)
c/Å	11.726 (4)
α/°	108.708 (9)
β/°	99.560 (9)
γ/°	93.757 (10)
Volume/Å^3	1099.4 (7)
Z	2
ρ_{calc} g/cm^3	1.237
μ/mm^{-1}	0.256
F(000)	436
Crystal size/mm^3	0.080 x 0.100 x 0.240
2Θ range for data collection/°	2.23 to 25.00°
Index ranges	-11<=h<=11, -12<=k<=12, -13<=l<=13
Reflections collected	43351
Independent reflections	3858 [R(int) = 0.1609]
Data/restraints/parameters	3858 / 0 / 338
Goodness-of-fit on F^2	1.053
Final R indexes [I>=2σ (I)]	R_1 = 0.0689, wR_2 = 0.0780
Final R indexes [all data]	R_1 = 0.1781, wR_2 = 0.1007
Largest diff. peak/hole / e Å^{-3}	0.298 and -0.211

Table S15. Crystal data and structure refinement for **7a** and ORTEP diagram of **7a** with ellipsoid contour at 50% probability level

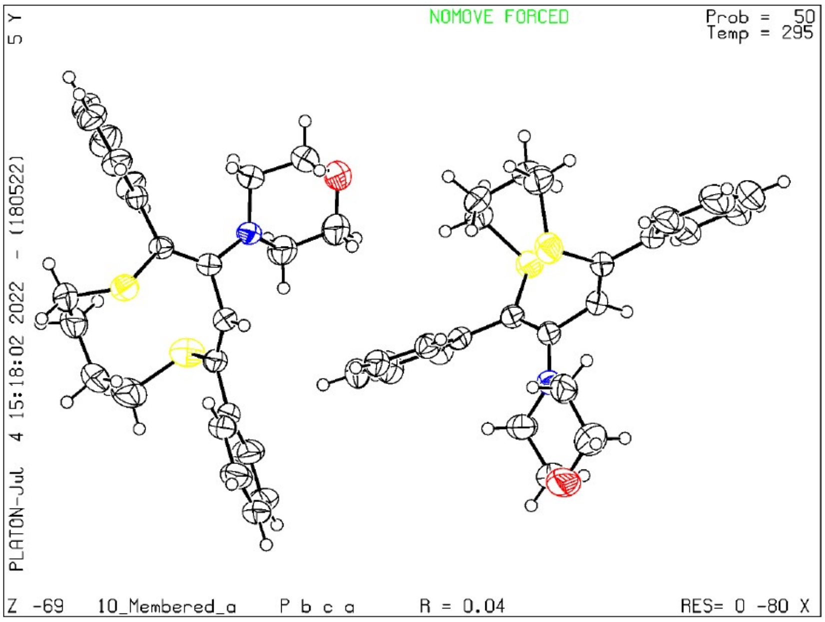

Chemical formula	$C_{24}H_{27}NOS_2$
Formula weight	409.58
Temperature/K	295 (0)
Crystal system	orthorhombic
Space group	P b c a
a/Å	21.4122 (18)
b/Å	12.0250 (11)
c/Å	34.111 (3)
α/°	90
β/°	90
γ/°	90
Volume/Å^3	8783.0 (14)
Z	16
ρ_{calc}g/cm^3	1.239
μ/mm^{-1}	0.257
F(000)	3488
2Θ range for data collection/°	2.25 to 25.00
Index ranges	-25<=h<=25, -14<=k<=14, -40<=l<=40
Reflections collected	328831
Independent reflections	7733 [R(int) = 0.0770]
Data/restraints/parameters	7733 / 0 / 506
Goodness-of-fit on F^2	1.035
Final R indexes [I>=2σ (I)]	R_1 = 0.0441, wR_2 = 0.0895
Final R indexes [all data]	R_1 = 0.0684, wR_2 = 0.1012
Largest diff. peak/hole / e Å^{-3}	0.331 and -0.217

Table S16. Crystal data and structure refinement for **7b** and ORTEP diagram of **7b** with ellipsoid contour at 50% probability level

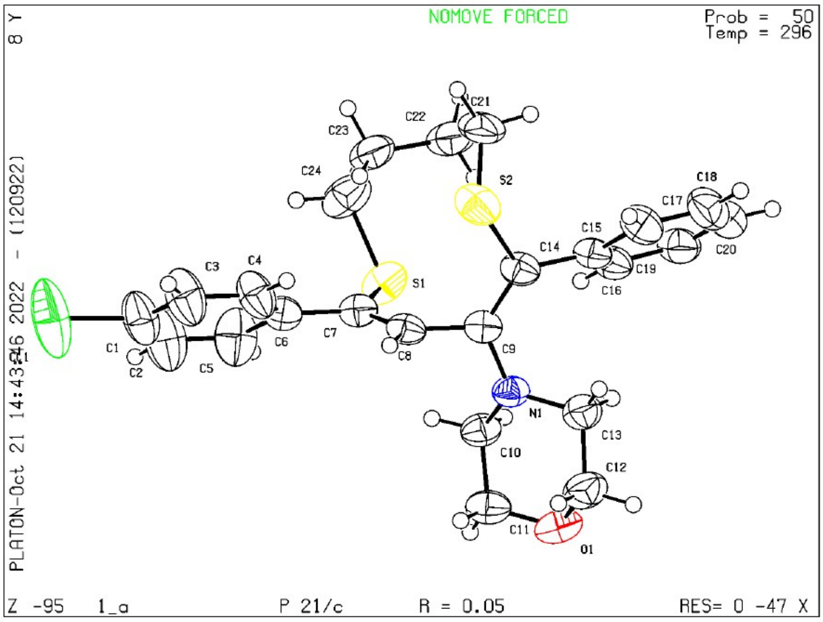

Chemical formula	$C_{24}H_{26}ClNOS_2$
Formula weight	444.03
Temperature/K	296 (0)
Crystal system	monoclinic
Space group	P 1 21/c 1
a/Å	14.0961(12)
b/Å	8.8816 (7)
c/Å	18.8140 (16)
α/°	90
β/°	106.035(2)°
γ/°	90
Volume/Å^3	2263.8(3)
Z	4
ρ_{calc}g/cm^3	1.303
μ/mm^{-1}	0.369
F(000)	936
2Θ range for data collection/°	2.25 to 25.00
Index ranges	-16<=h<=16, -10<=k<=10, -22<=l<=22
Reflections collected	69667
Independent reflections	3988 [R(int) = 0.0474]
Data/restraints/parameters	3988 / 0 / 263
Goodness-of-fit on F^2	1.012
Final R indexes [I>=2σ (I)]	R_1 = 0.0464, wR_2 = 0.0813
Final R indexes [all data]	R_1 = 0.0679, wR_2 = 0.0921
Largest diff. peak/hole / e Å^{-3}	0.580 and -0.414

15. References

(S1) Ismailoglu, E.; Mert, Z.; Dinc, M.; Kaya, K.; Yucel, B. Synthesis of 3-Amino-4-iodothiophenes through Iodocyclization of 1-(1,3-Dithian-2-yl)propargylamines. *Eur. J. Org. Chem.* **2021**, 4107–4124.

(S2) 2-Aryl-1,3-dithiane derivatives were prepared according to literatüre procedure, see; Besra, R. C.; Rudrawar, S.; Chakraborti, A. K.; Copper(II) tetrafluoroborate as an extremely efficient catalyst for 1,3-dithiolane/dithiane formation from carbonyl compounds under solvent-free conditions at room temperature. *Tetrahedron Lett*. **2005**, *46*, 6213–6217.

(S3) Sheldrick, G. M. SHELXS-97, Program for Crystal Structure Solution, University of Göttingen, Göttingen, 1997.

(S4) Sheldrick, G. M. Foundations of Crystallography. Acta Crystallogr. Sect. A **2008**, *64*, 112–122.

(S5) Dolomanov, O. V.; Bourhis, L. J.; Gildea, R. J.; Howard, J. A. K.; Puschmann, H. OLEX2: a complete structure solution, refinement and analysis program. *J. Appl. Crystallogr.* **2009**, 42, 339–341.

Figure S5. ^{1}H- NMR ($CDCl_3$, 500 MHz) spectrum **1'c**

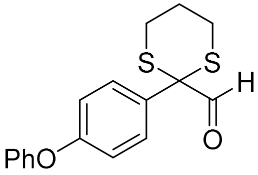

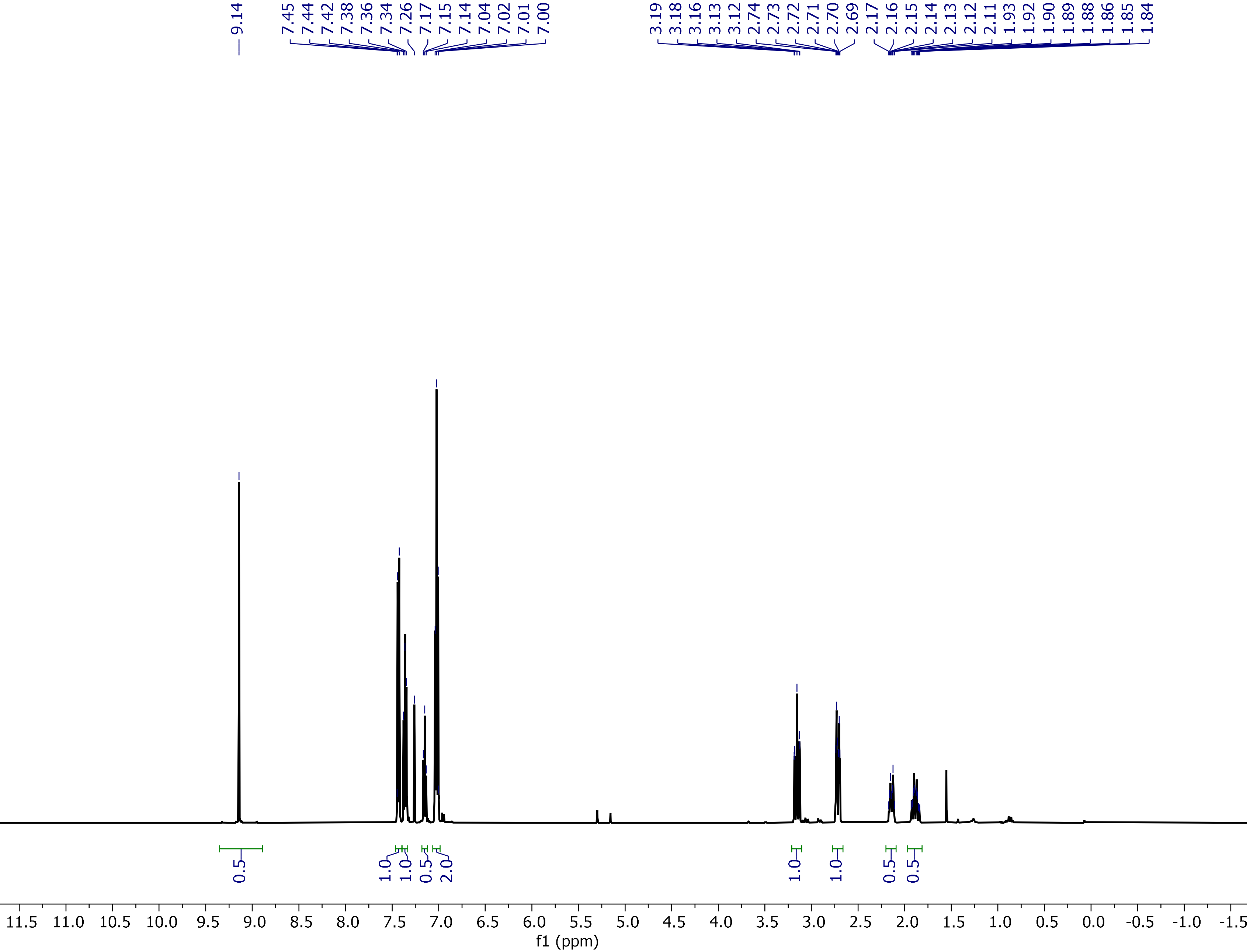

Figure S6. ^{13}C NMR ($CDCl_3$, 125 MHz) spectrum of **1'c**

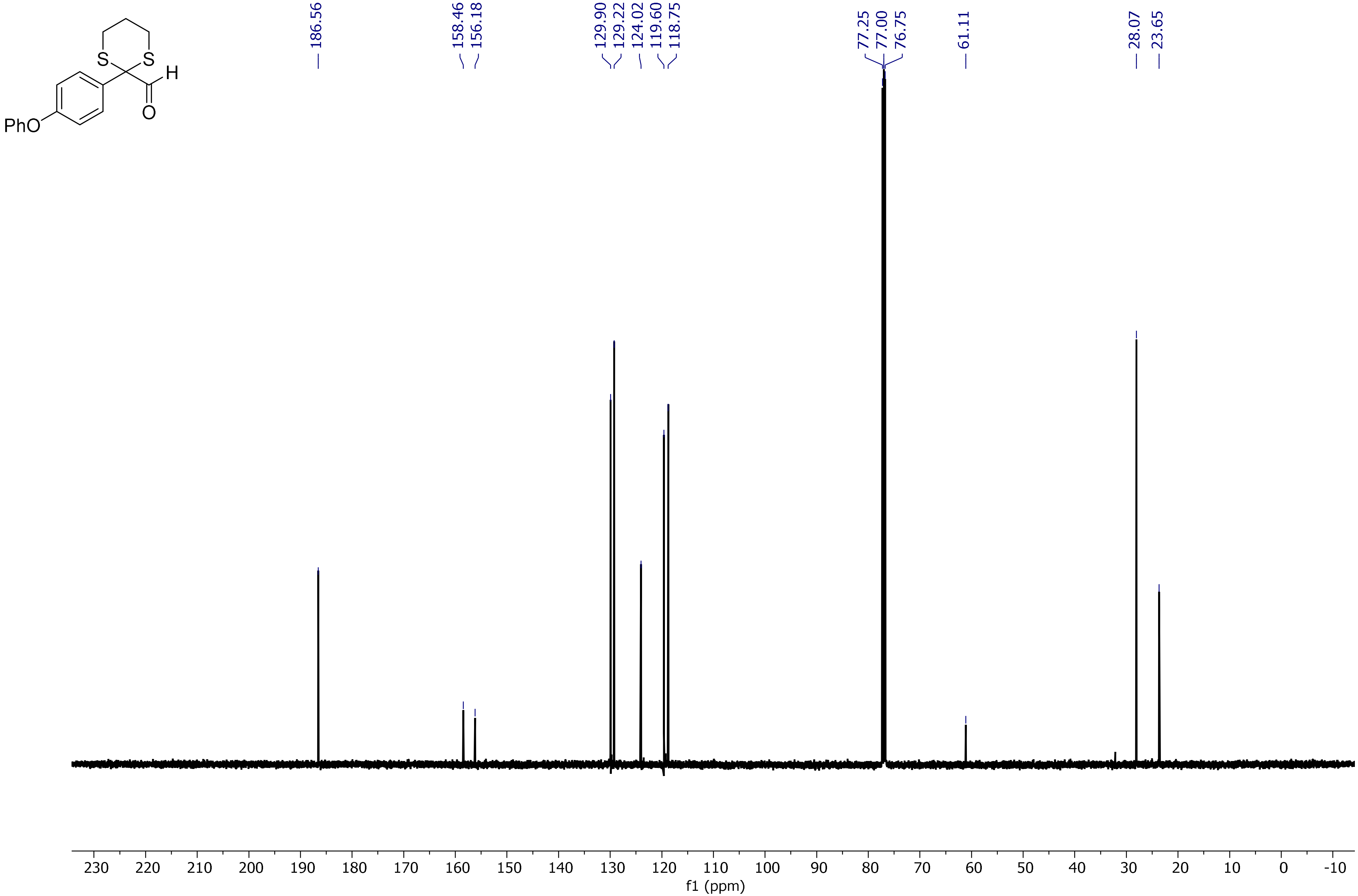

Figure S7. ^{1}H- NMR ($CDCl_3$, 500 MHz) spectrum **4'a**

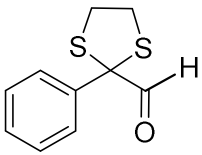

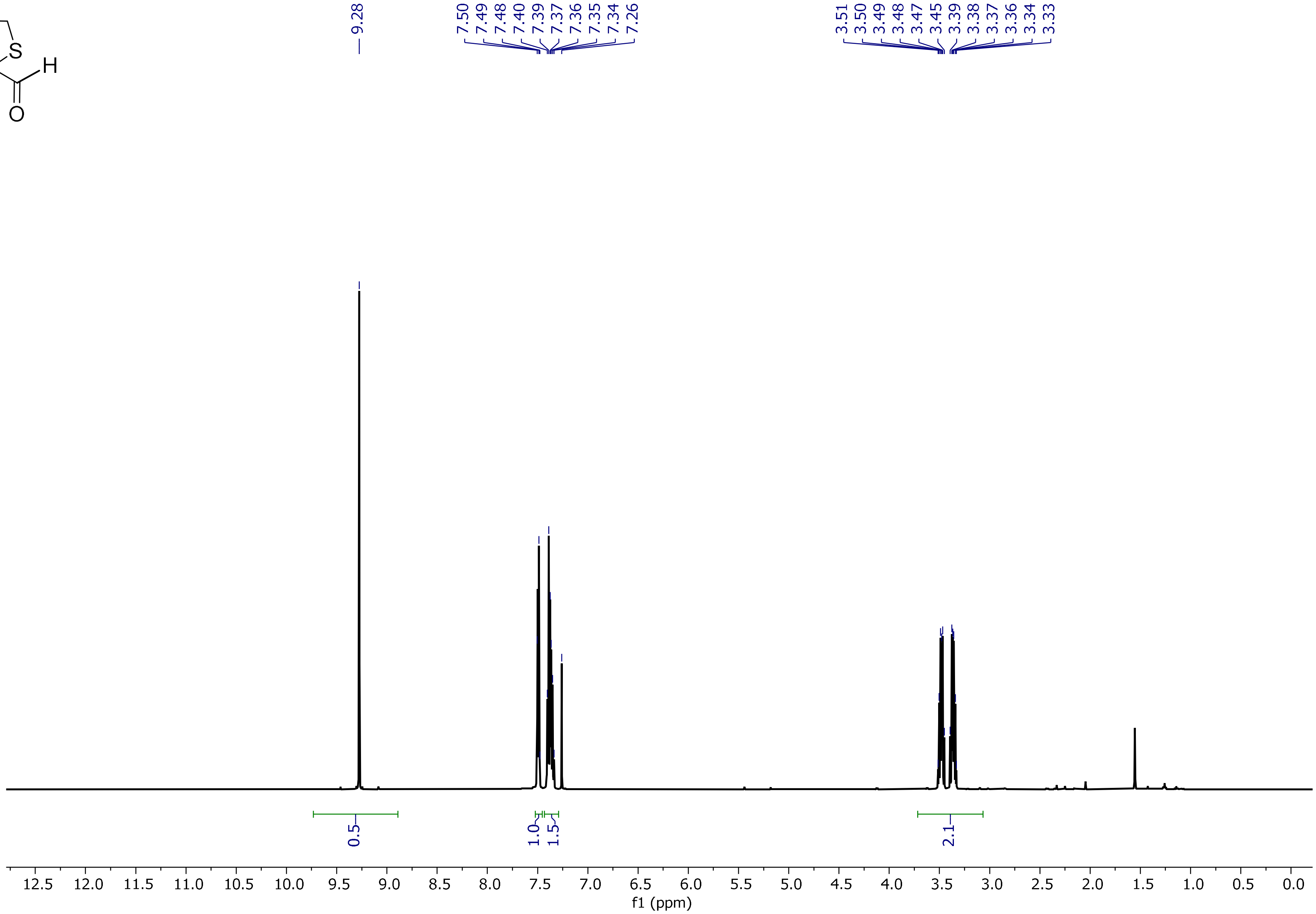

Figure S8. ^{13}C NMR ($CDCl_3$, 125 MHz) spectrum of **4'a**

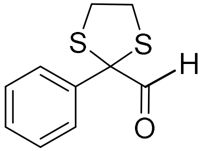

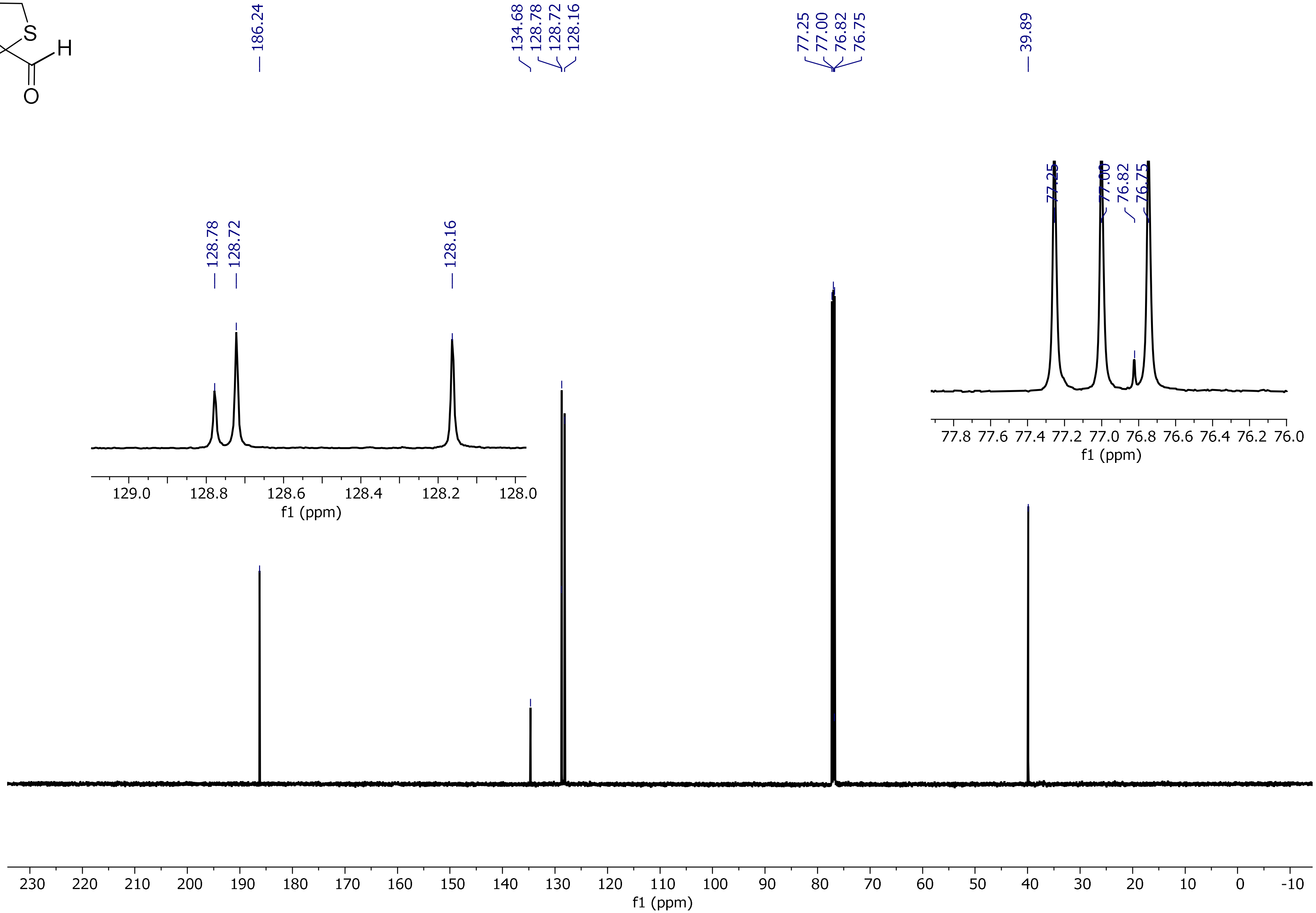

Figure S9. ^{1}H- NMR ($CDCl_3$, 500 MHz) spectrum **5'a**

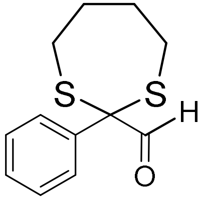

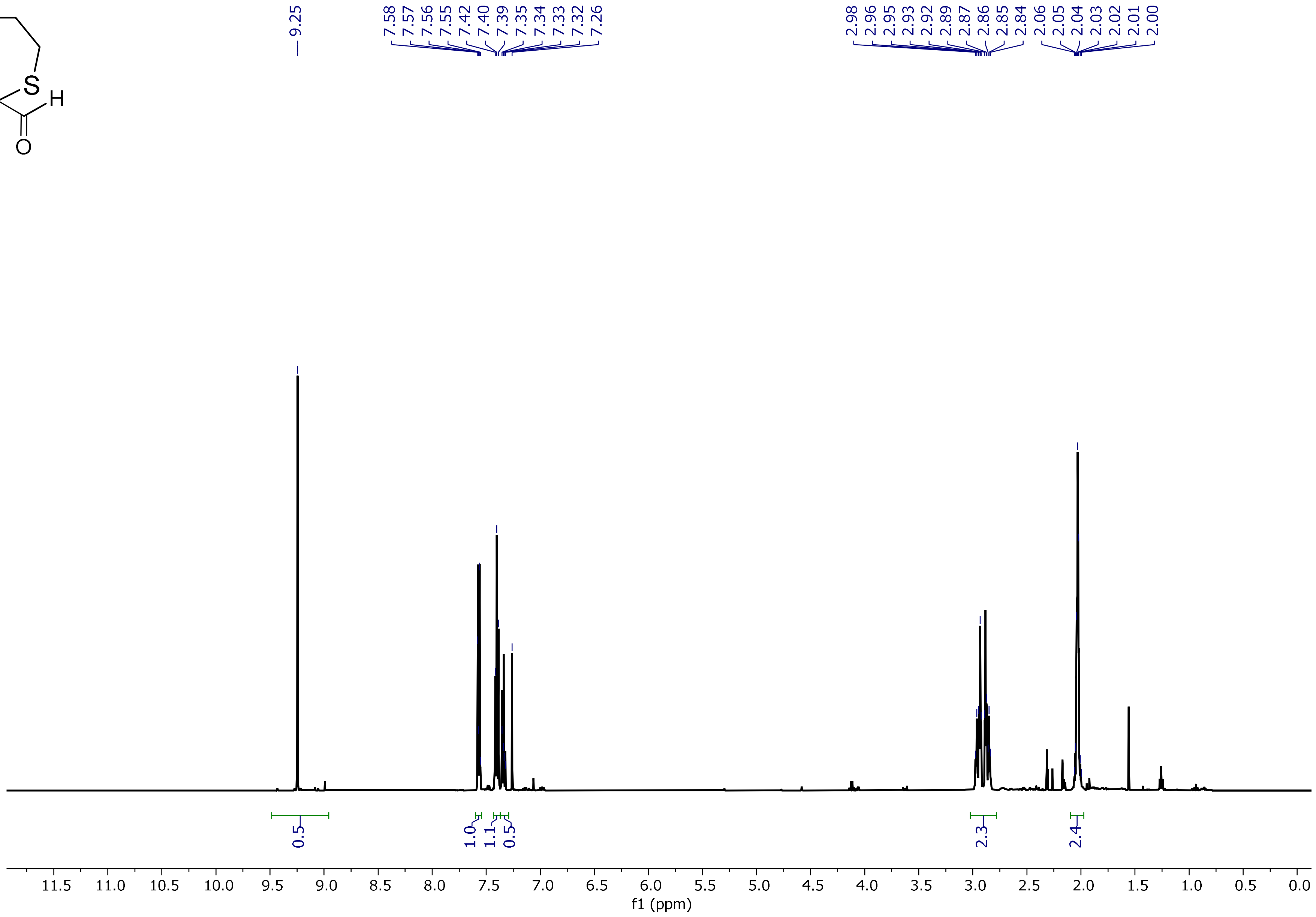

Figure S10. ^{13}C NMR ($CDCl_3$, 125 MHz) spectrum of **5'a**

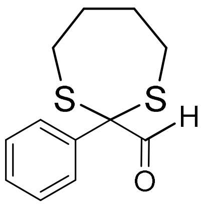

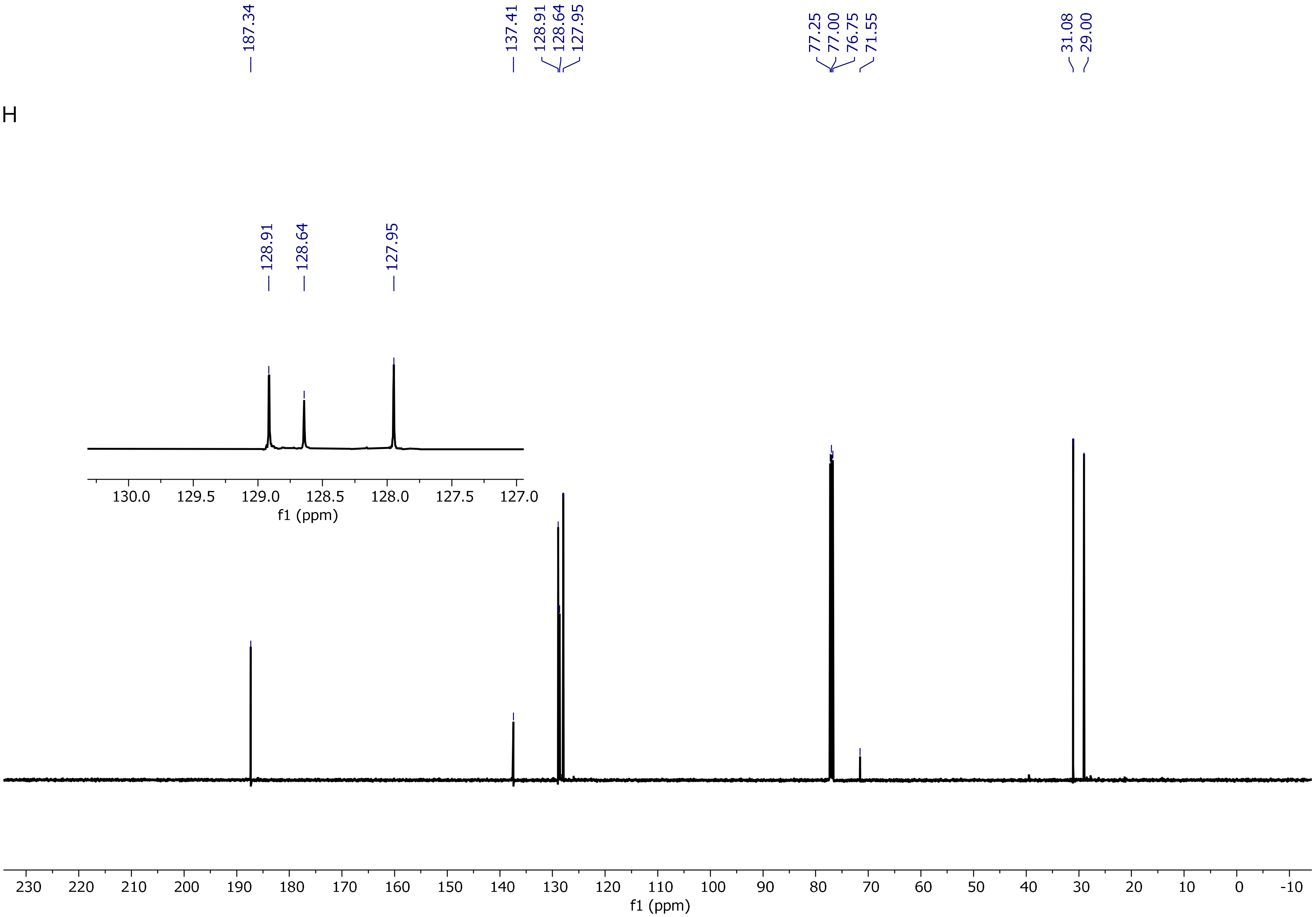

Figure S11. ^{1}H- NMR ($CDCl_3$, 500 MHz) spectrum **1e**

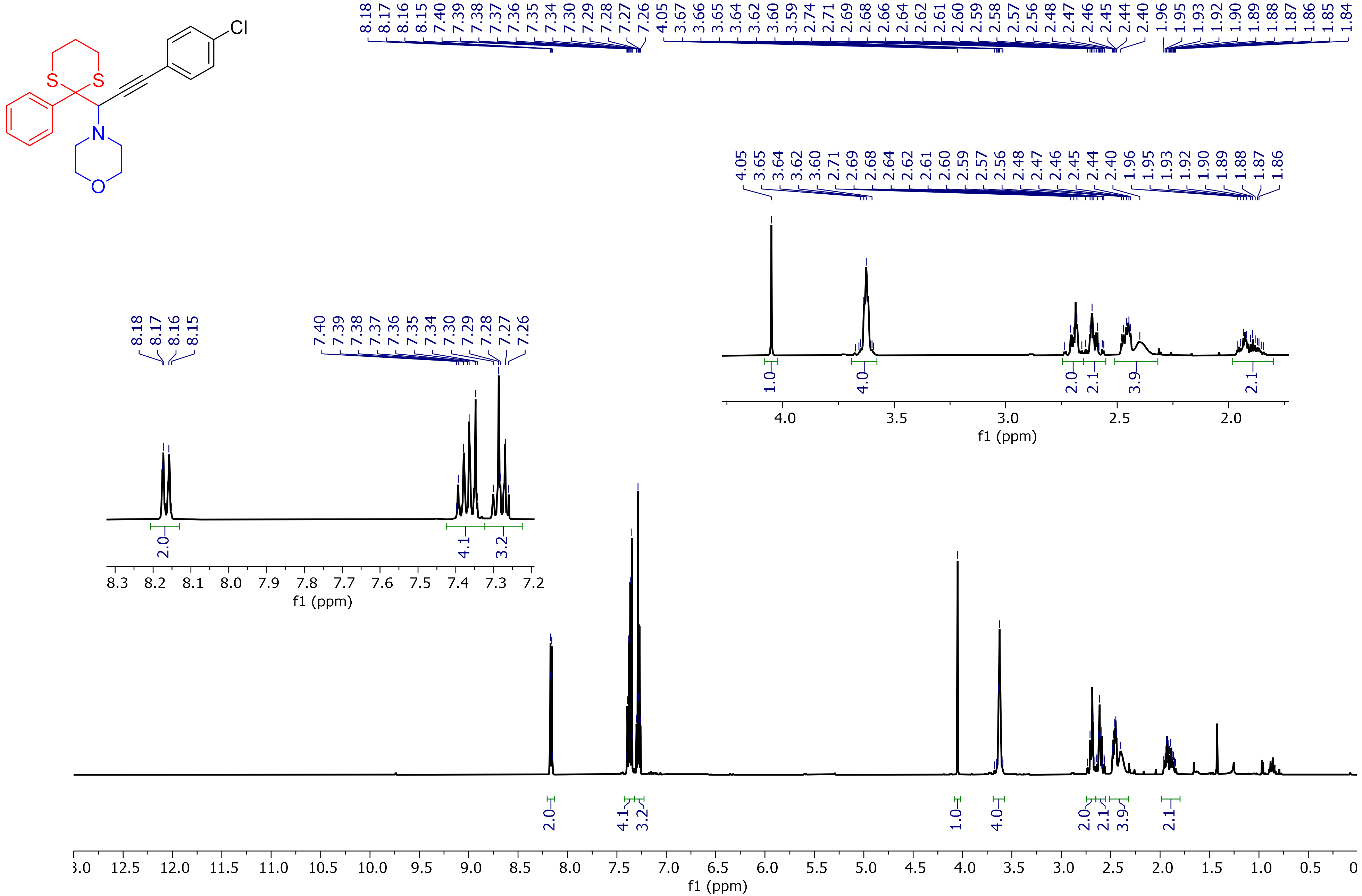

Figure S12. ^{13}C-APT NMR ($CDCl_3$, 125 MHz) spectrum **1e**

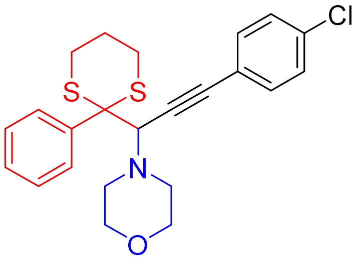

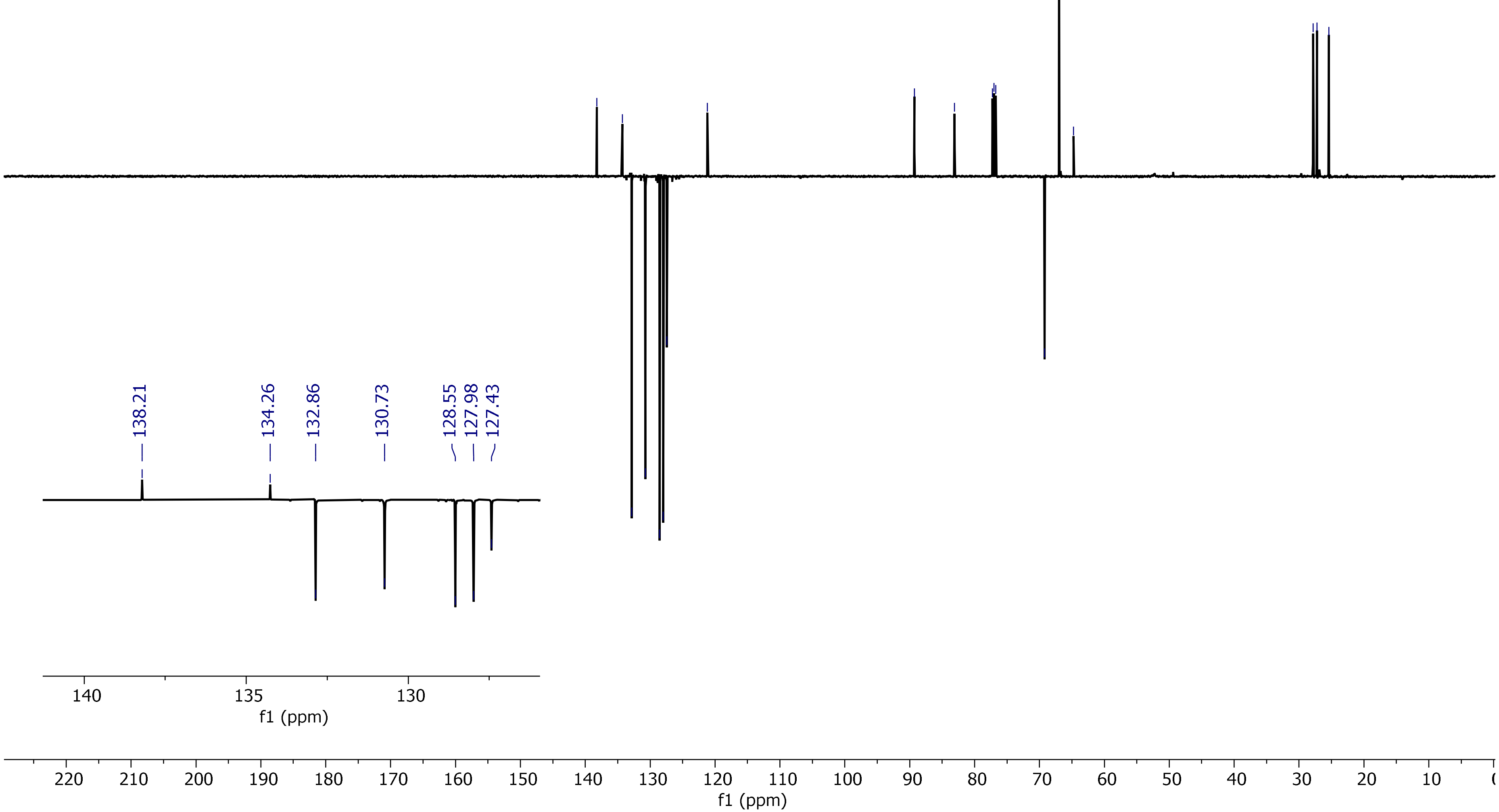

Figure S13. ^{1}H- NMR ($CDCl_3$, 500 MHz) spectrum **1f**

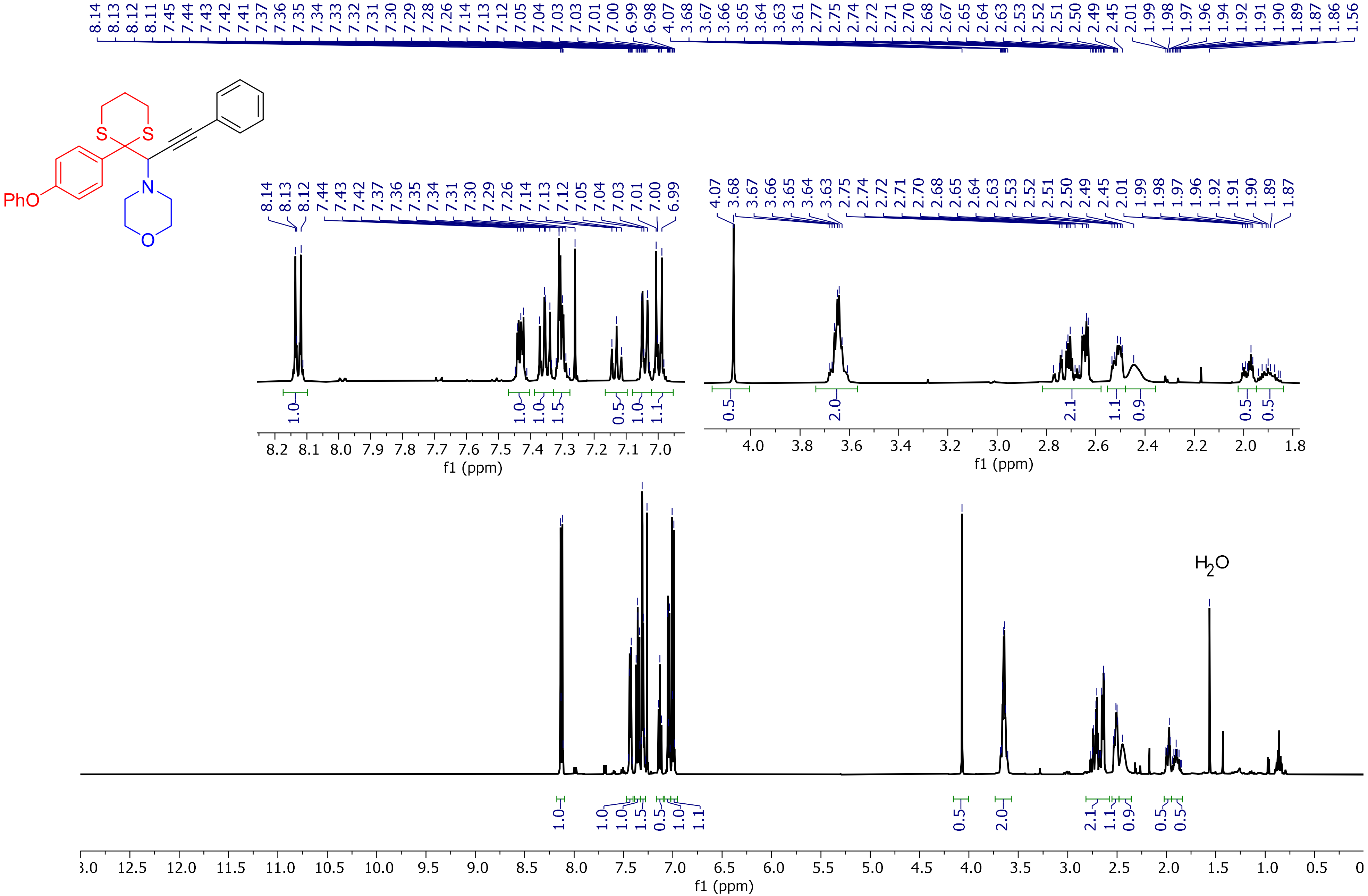

Figure S14. ^{13}C-APT NMR ($CDCl_3$, 125 MHz) spectrum **1e**

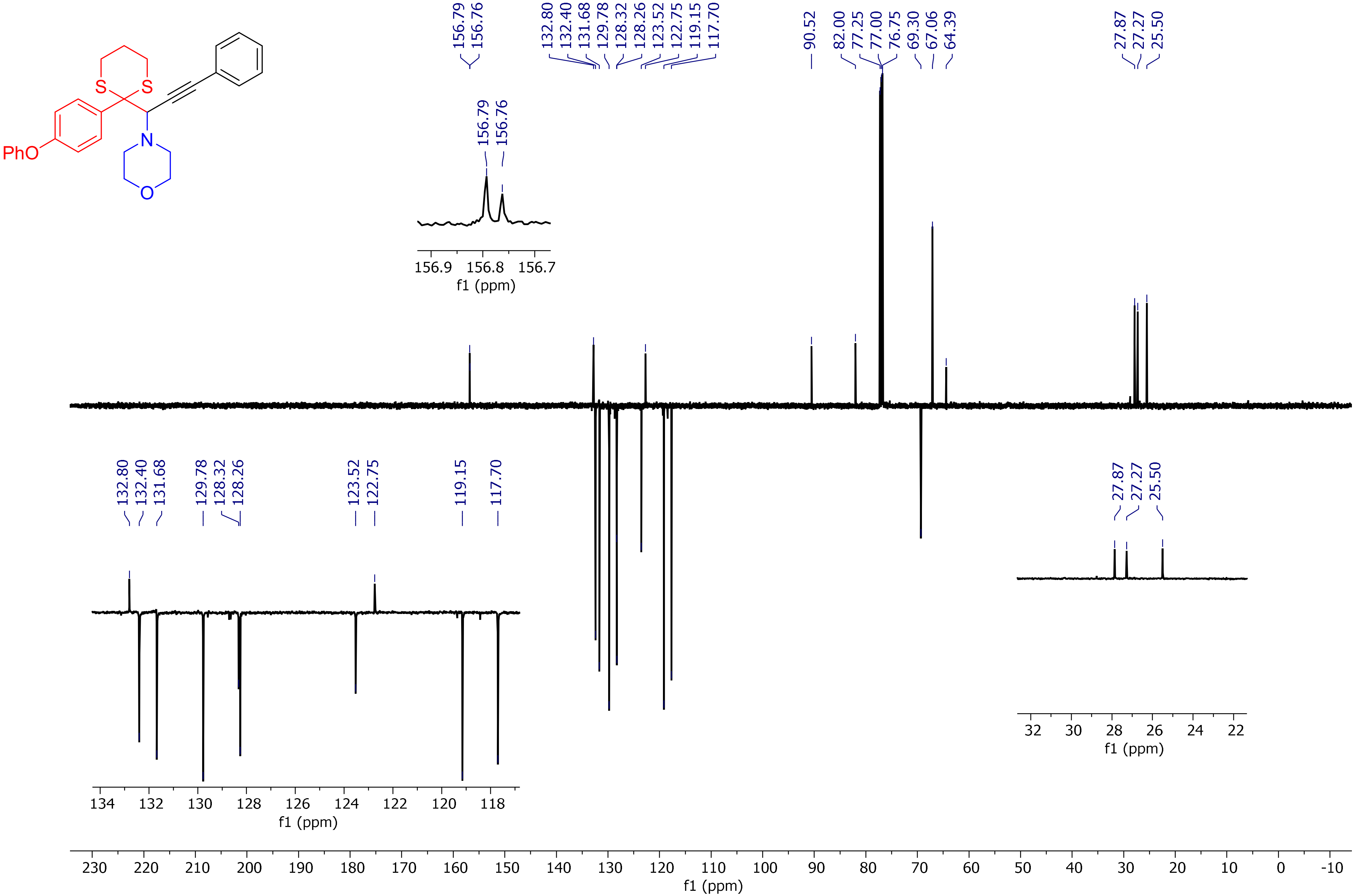

Figure S15. ^{1}H- NMR ($CDCl_3$, 500 MHz) spectrum **1g**

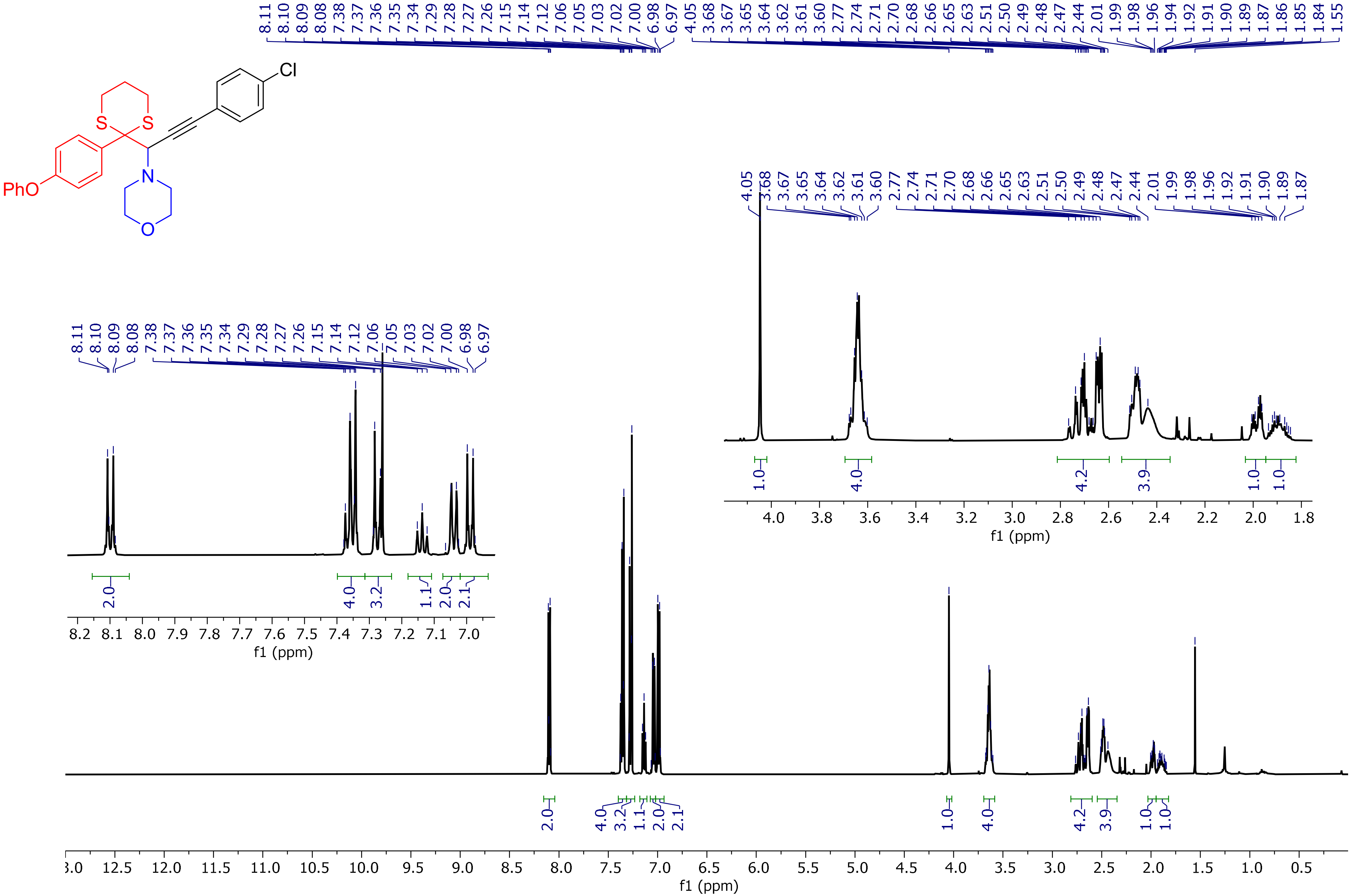

Figure S16. ^{13}C-APT NMR ($CDCl_3$, 125 MHz) spectrum **1g**

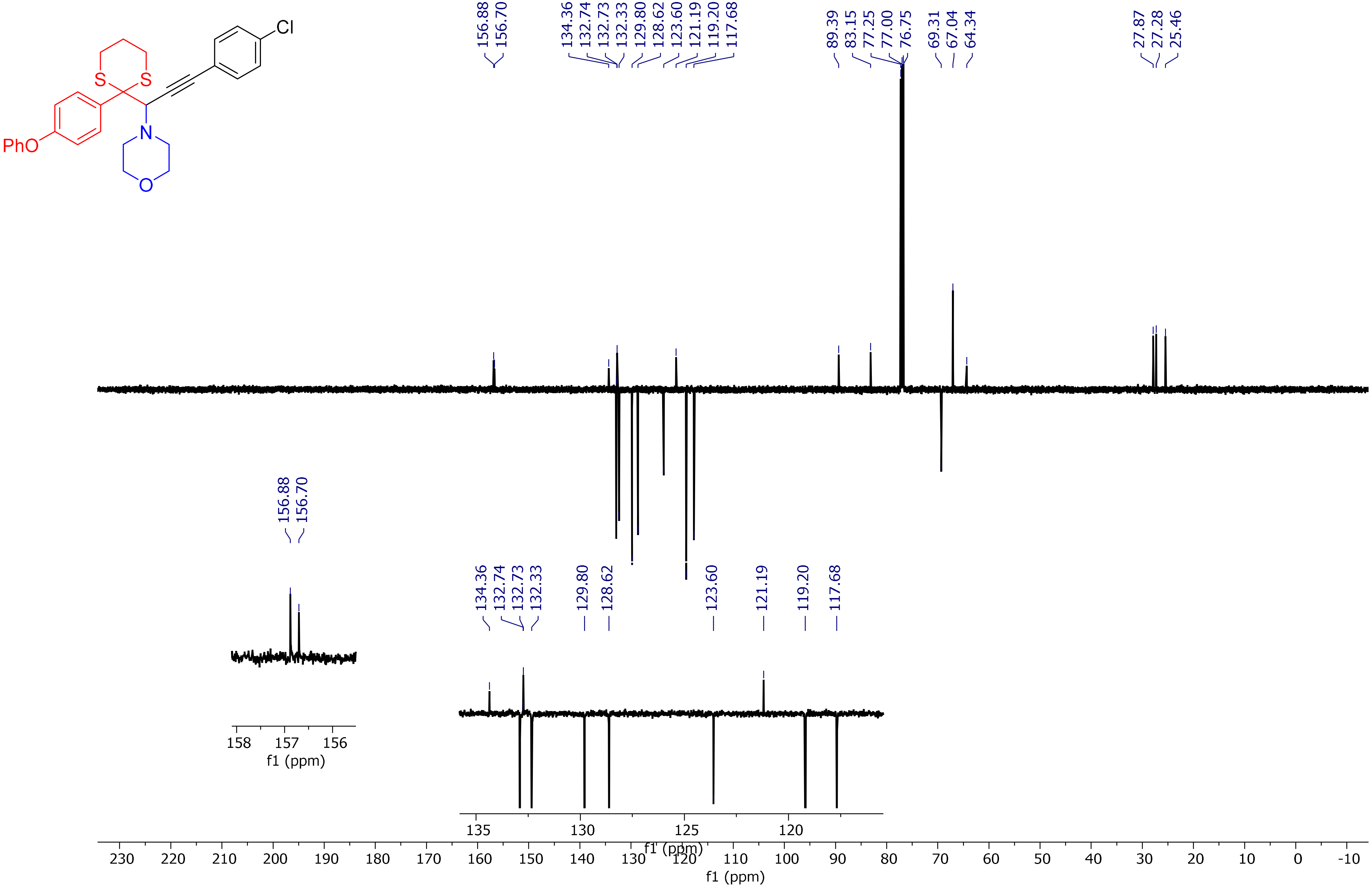

Figure S17. ^{1}H- NMR ($CDCl_3$, 500 MHz) spectrum **1u**

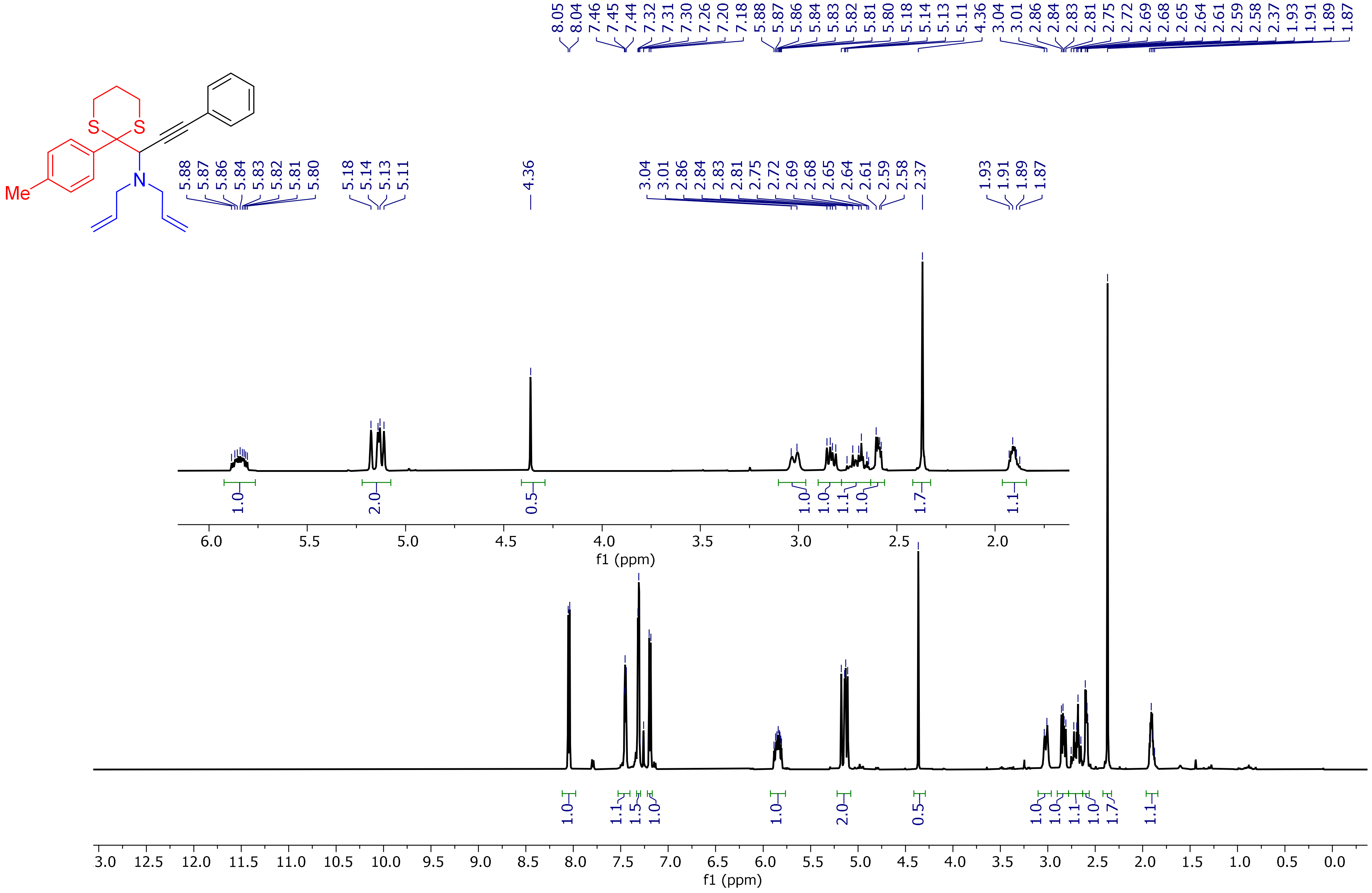

Figure S18. ^{13}C-NMR ($CDCl_3$, 125 MHz) spectrum **1u**

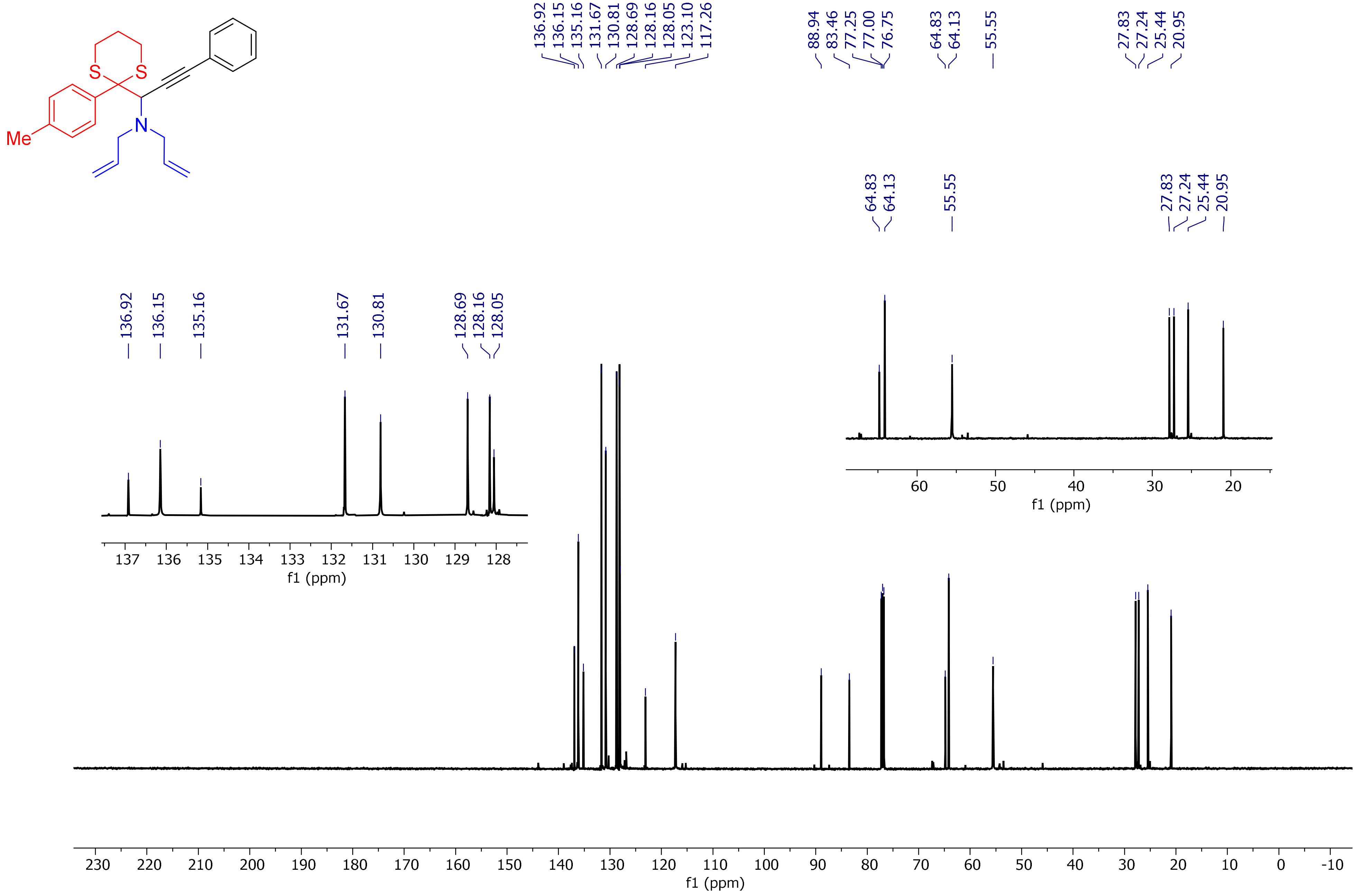

Figure S19. ^{13}C-APT NMR ($CDCl_3$, 125 MHz) spectrum **1u**

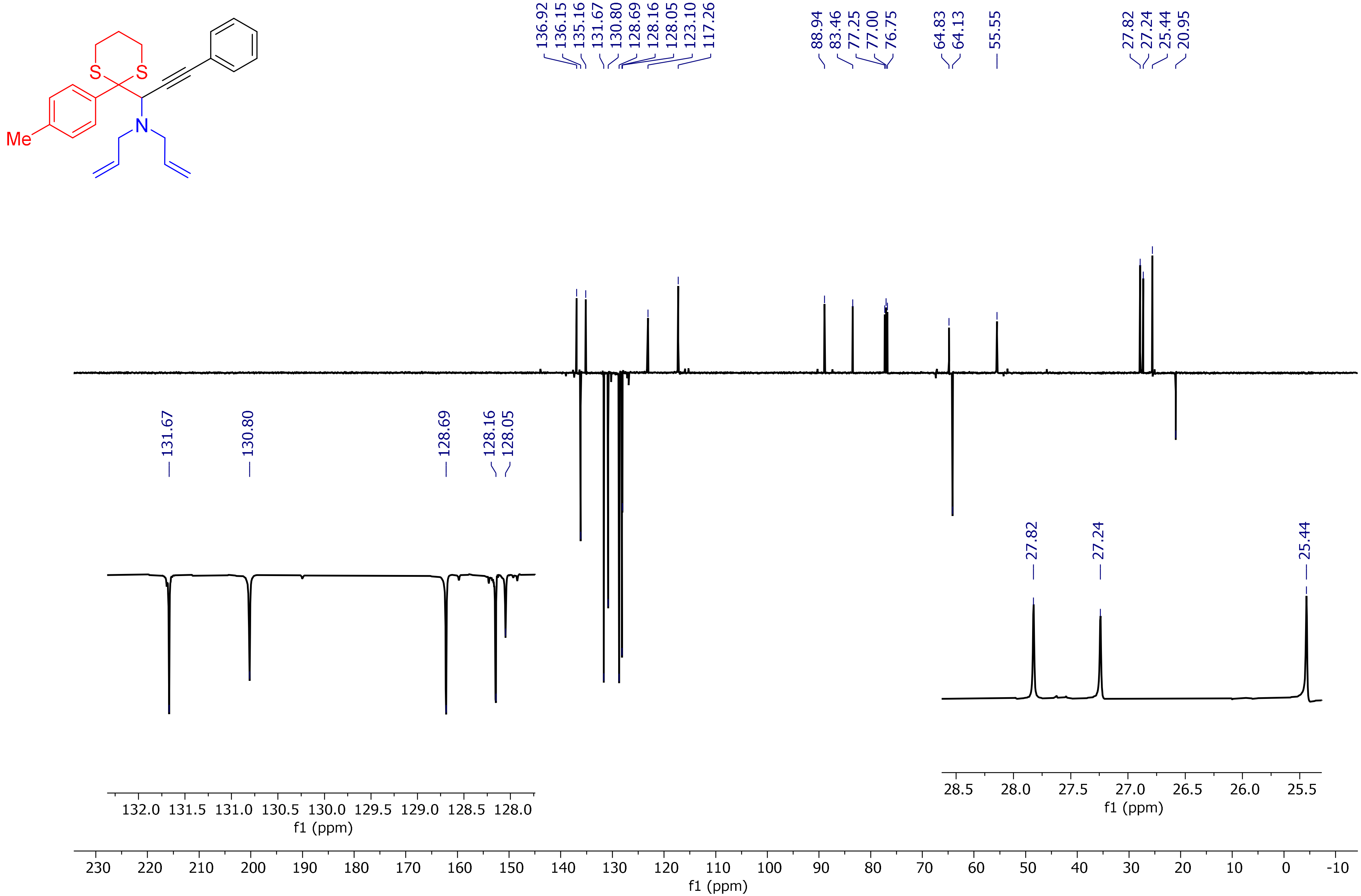

Figure S20. ^{1}H- NMR ($CDCl_3$, 500 MHz) spectrum **1y**

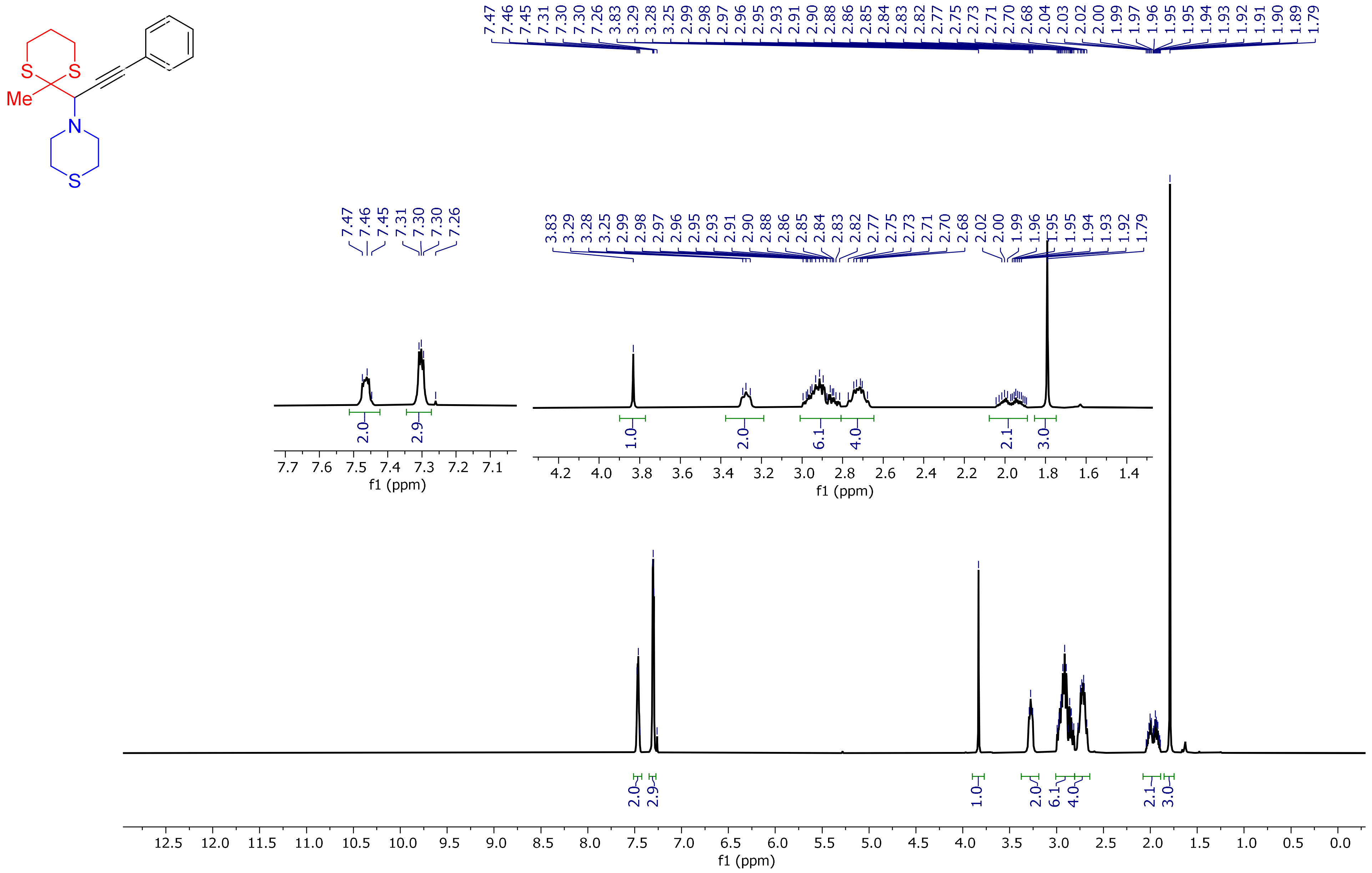

Figure S21. ^{13}C-NMR ($CDCl_3$, 125 MHz) spectrum **1y**

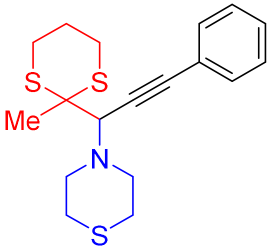

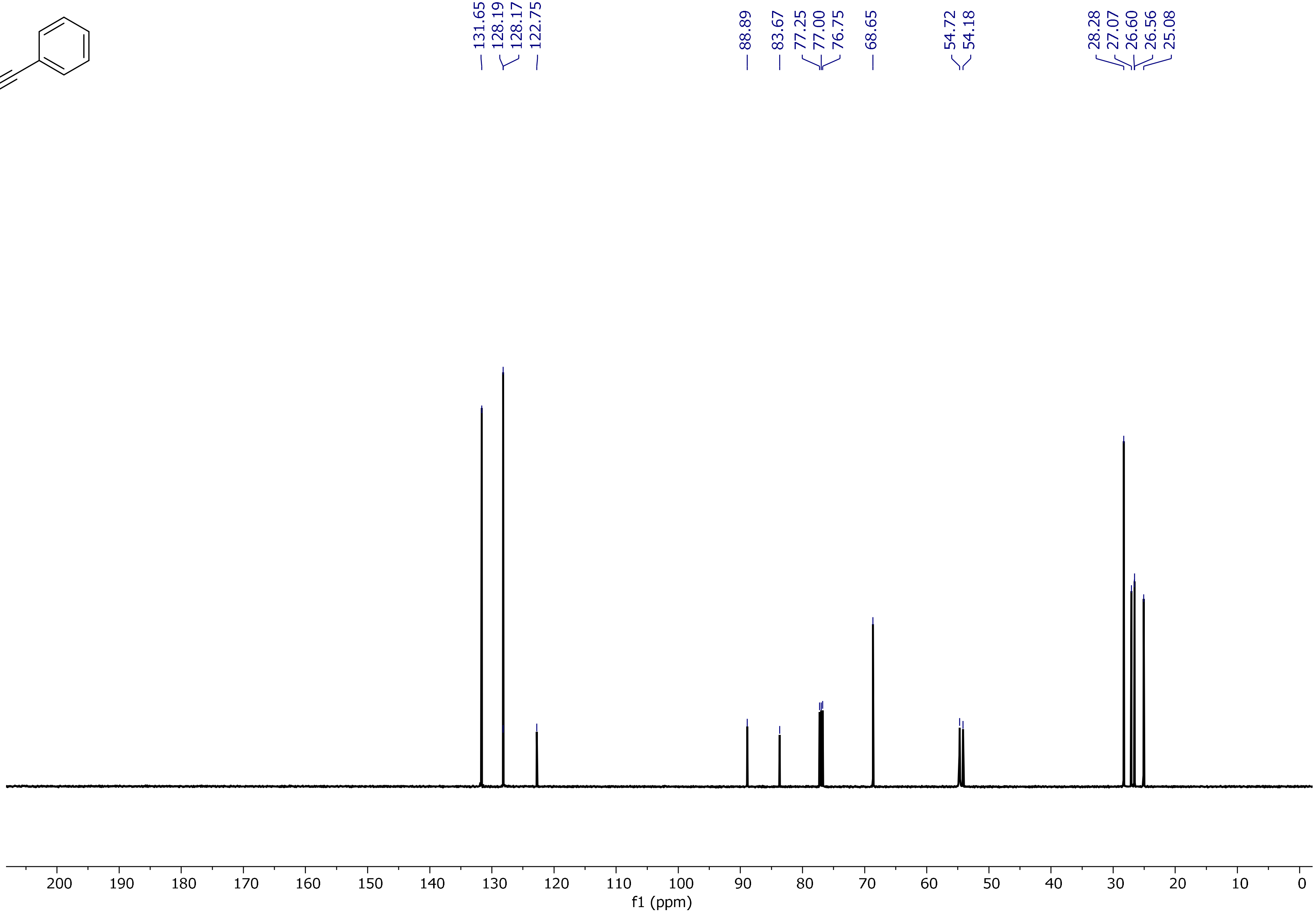

Figure S22. ^{13}C-APT NMR ($CDCl_3$, 125 MHz) spectrum **1y**

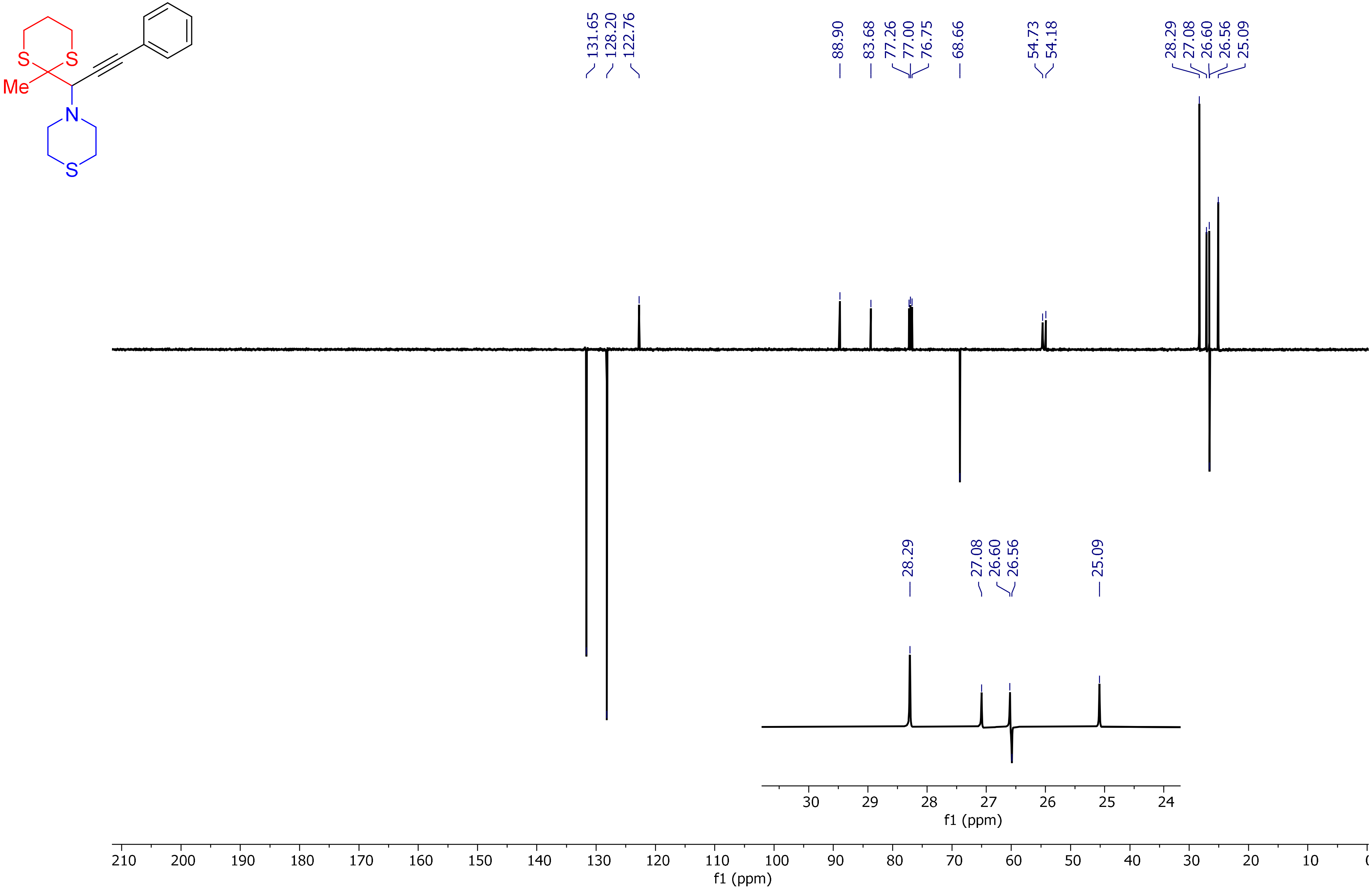

Figure S23. ^{1}H- NMR ($CDCl_3$, 500 MHz) spectrum **1ad**

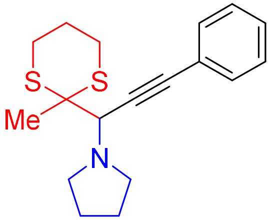

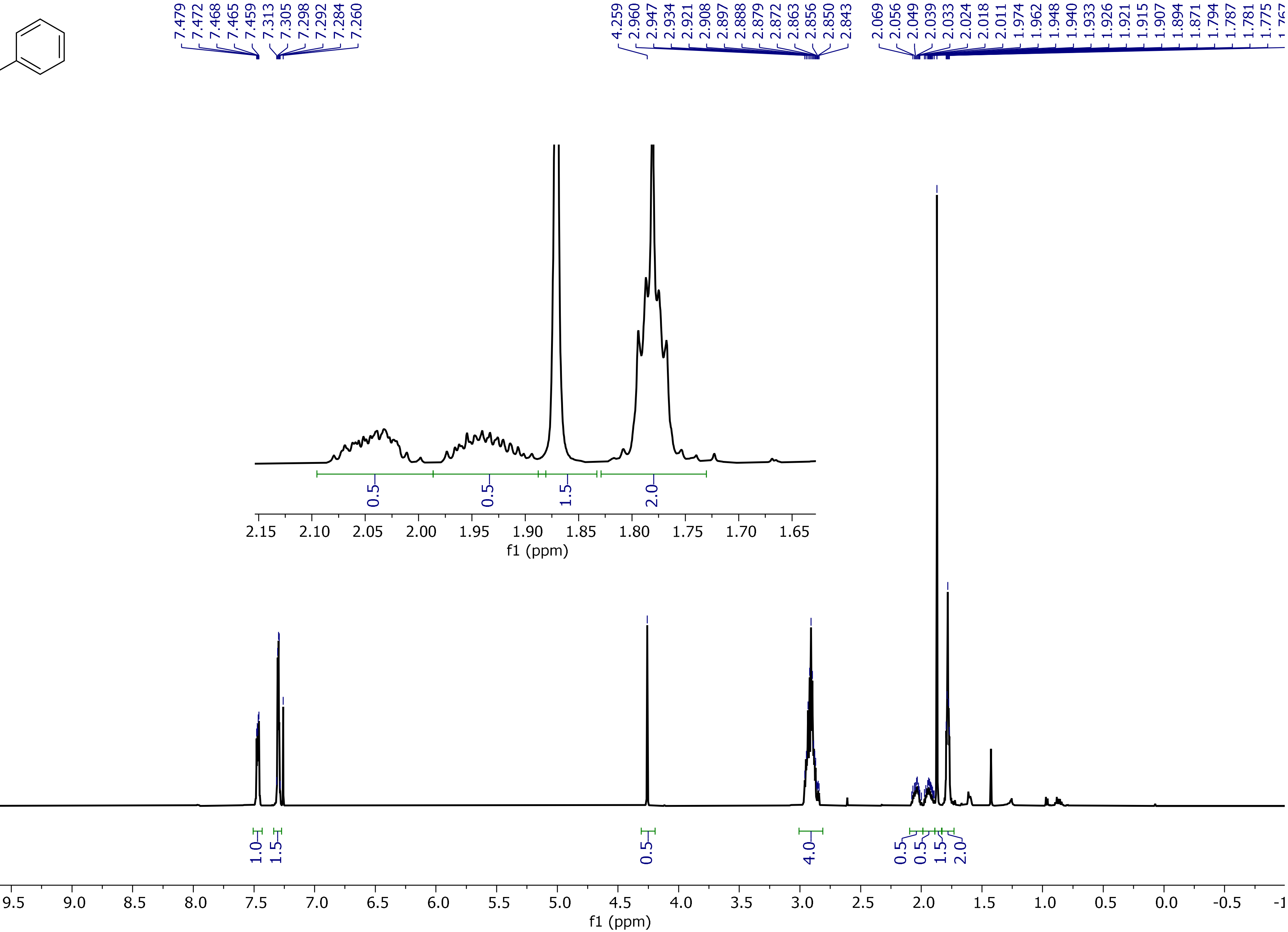

Figure S24. ^{13}C-NMR ($CDCl_3$, 125 MHz) spectrum **1ad**

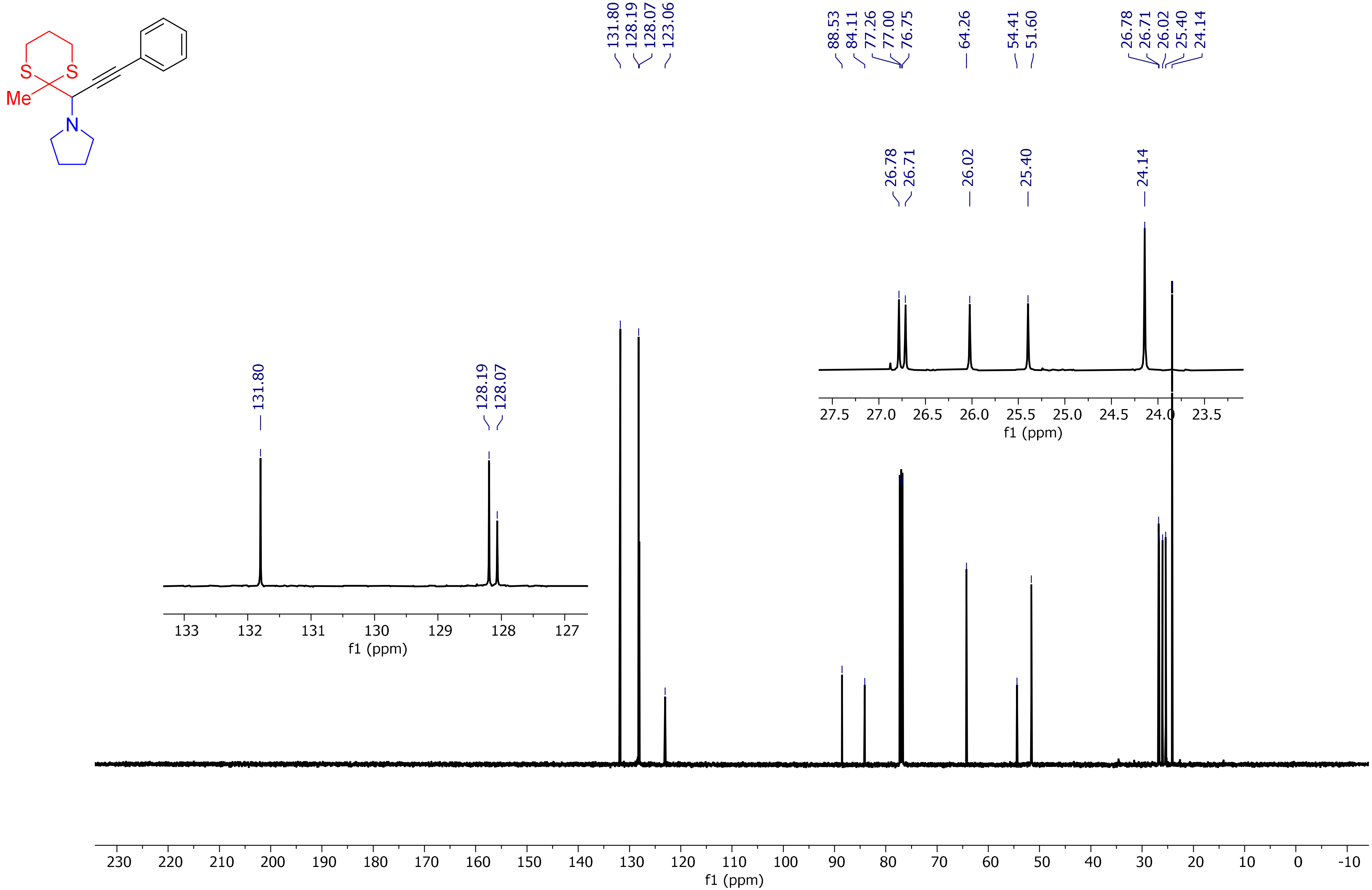

Figure S25. ^{13}C-APT NMR ($CDCl_3$, 125 MHz) spectrum **1ad**

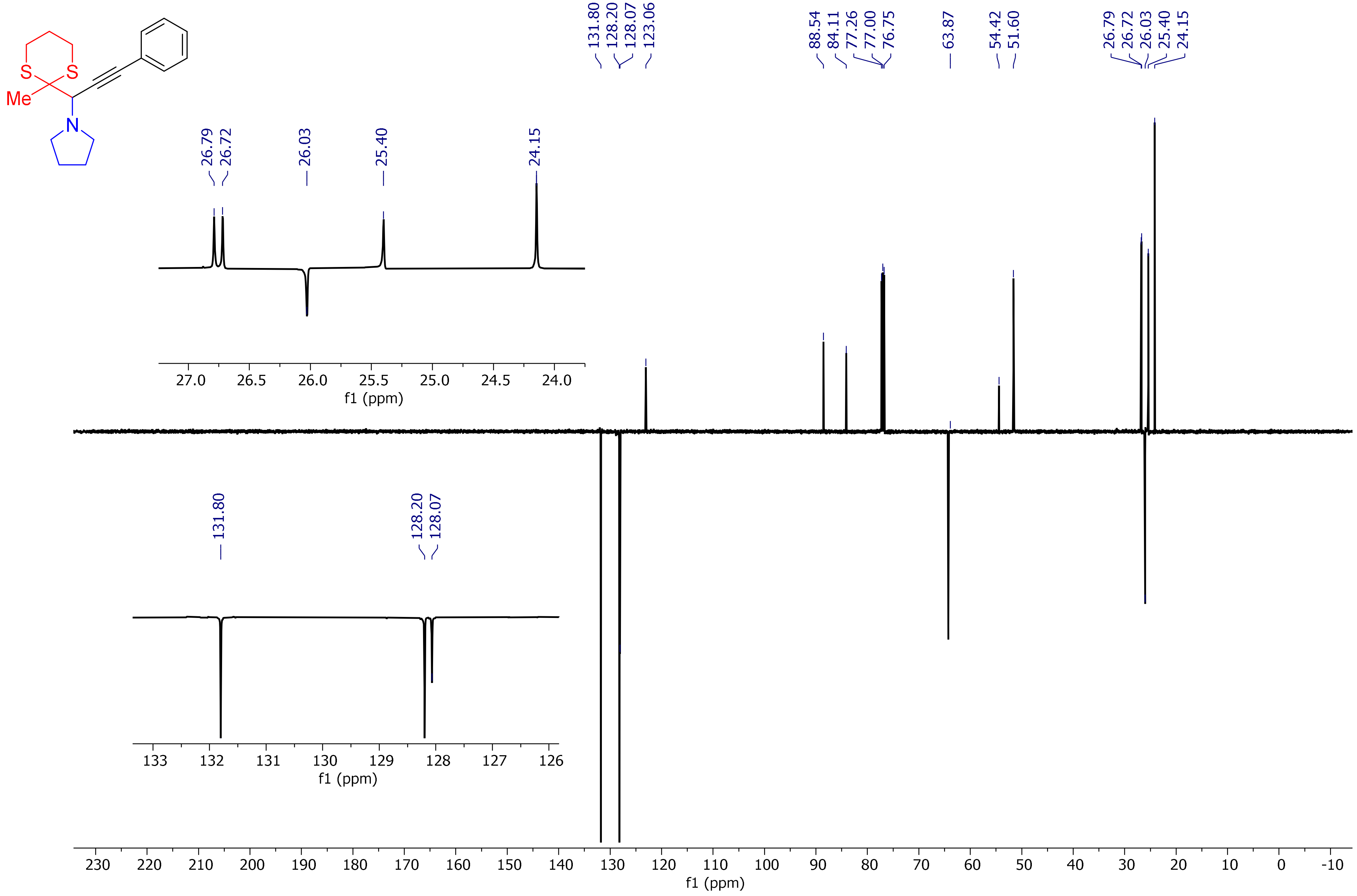

Figure S26. ^{1}H-NMR ($CDCl_3$, 500 MHz) spectrum **1a$_1$**

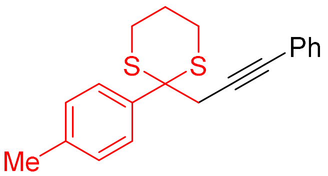

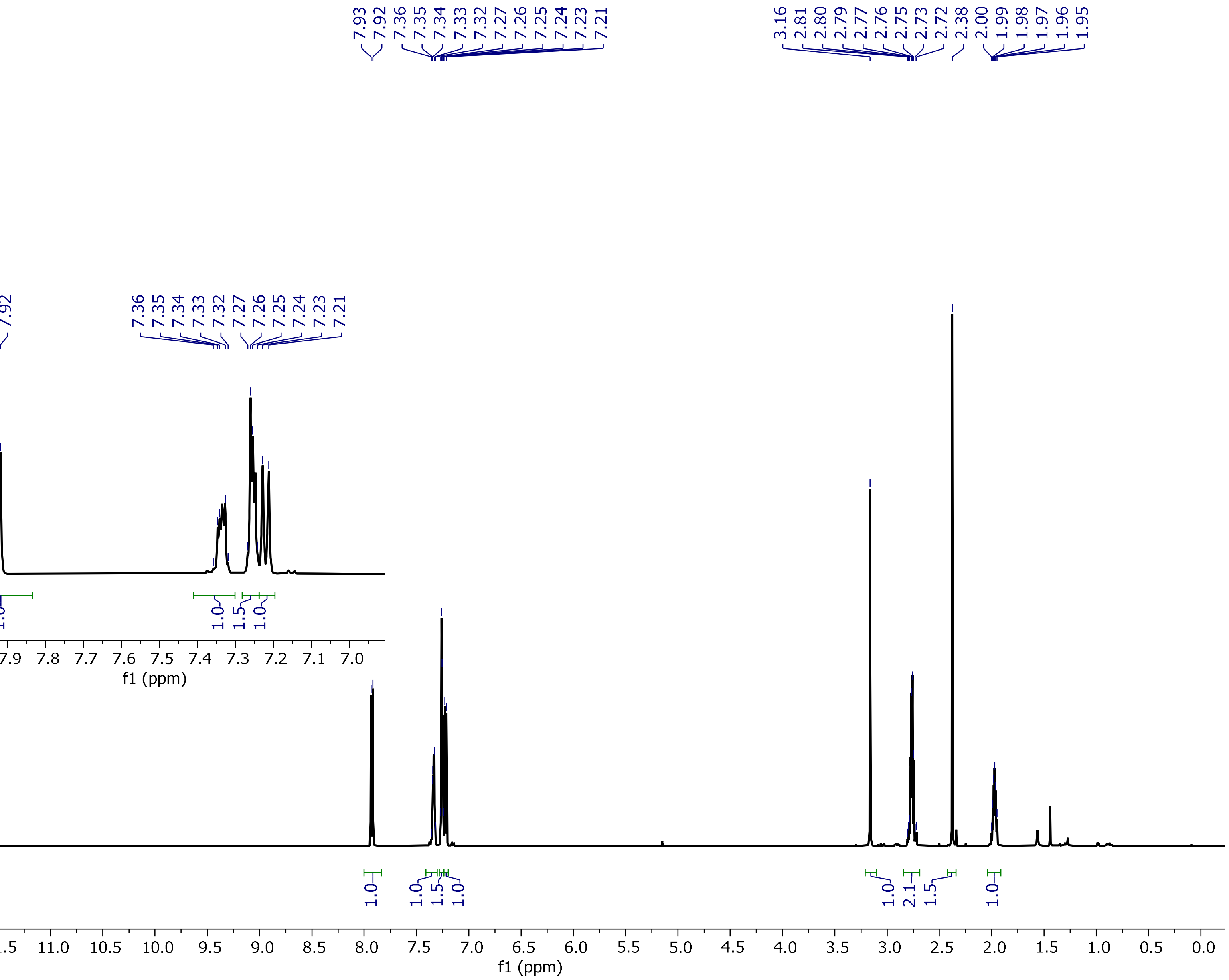

Figure S27. ^{13}C-NMR ($CDCl_3$, 125 MHz) spectrum **1a$_1$**

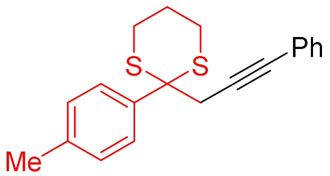

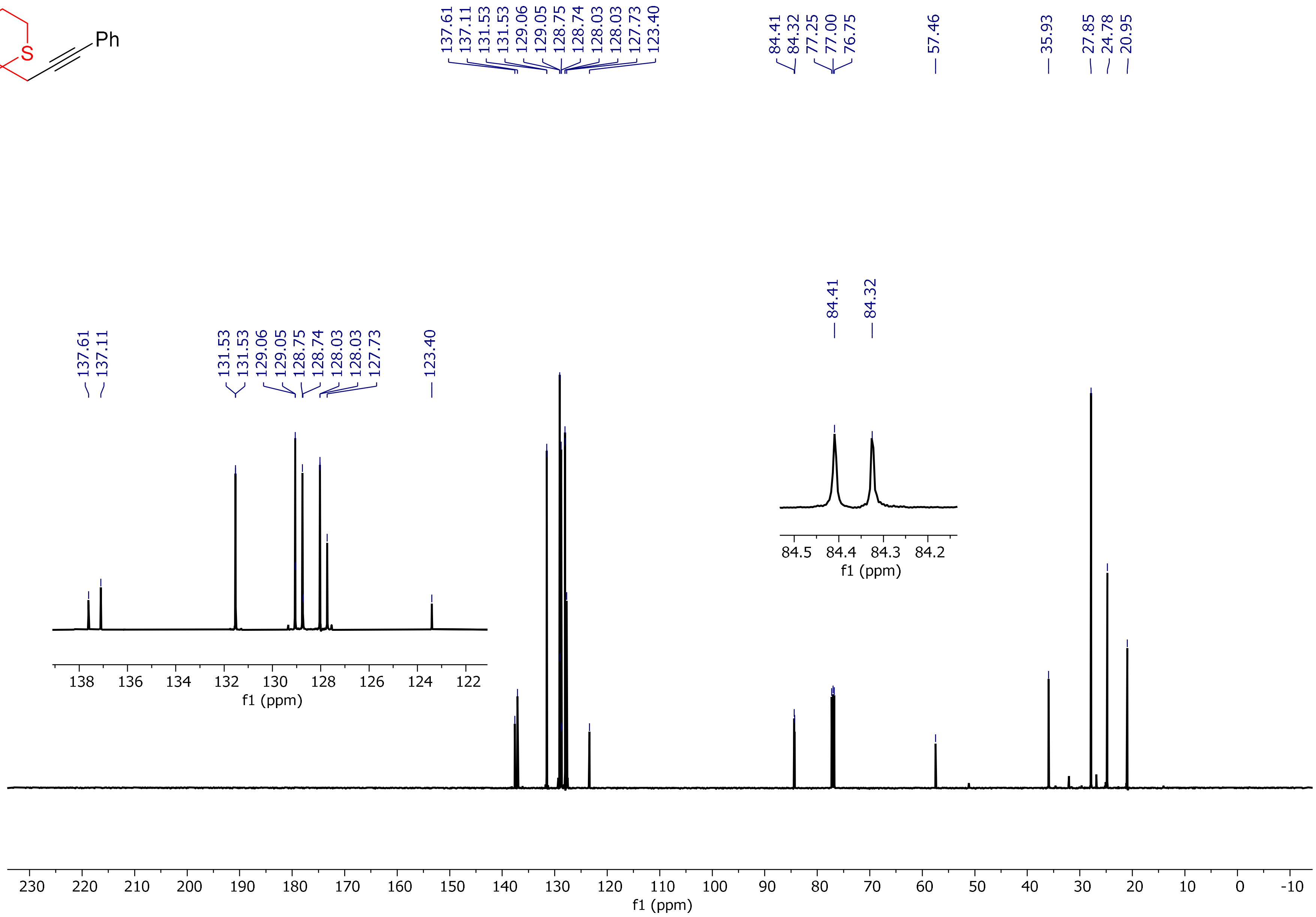

Figure S28. ^{13}C-APT NMR ($CDCl_3$, 125 MHz) spectrum **1a$_1$**

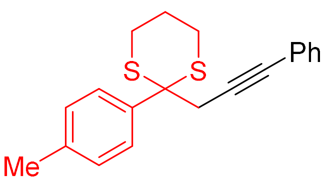

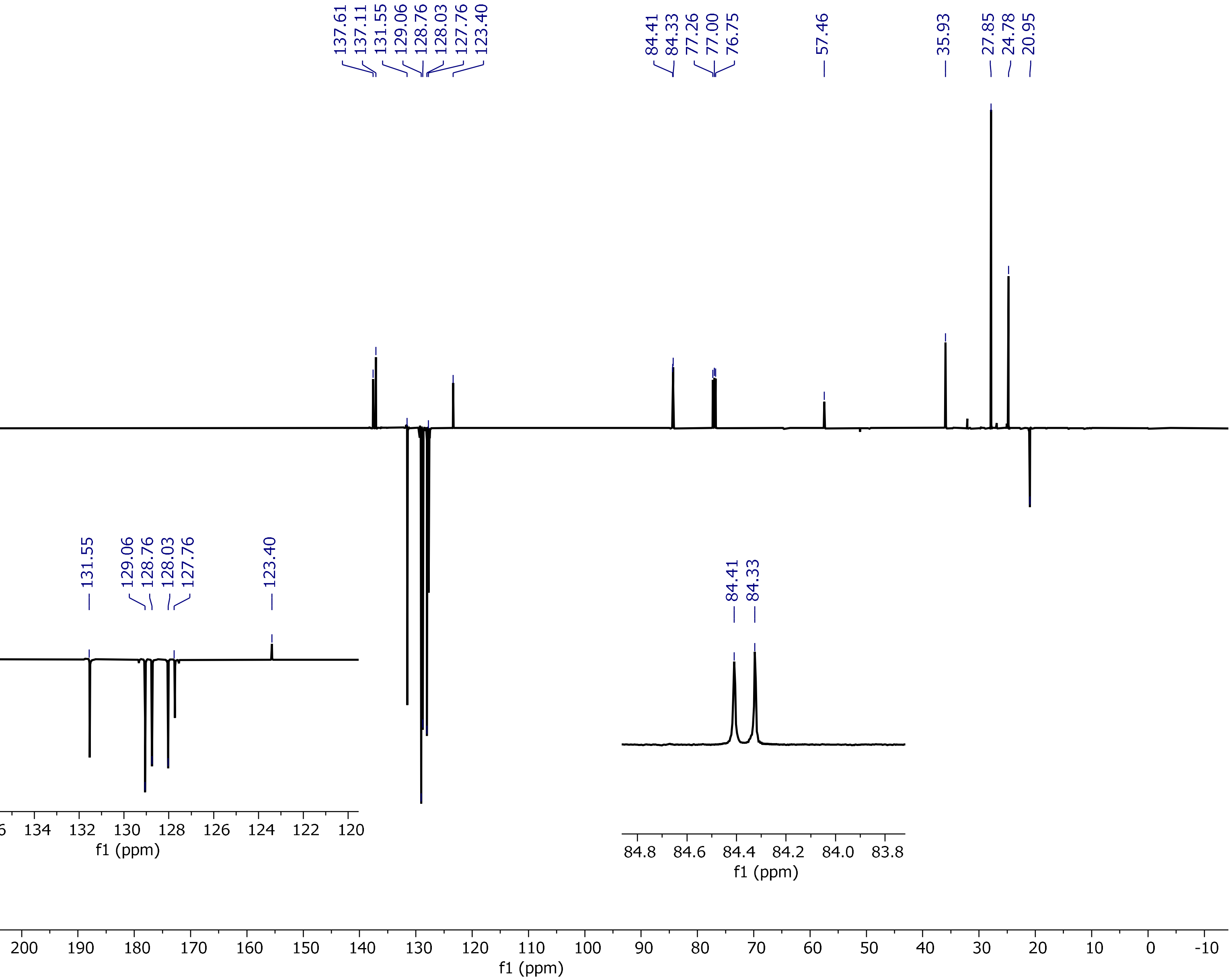

Figure S29. ^{1}H- NMR ($CDCl_3$, 500 MHz) spectrum **4a**

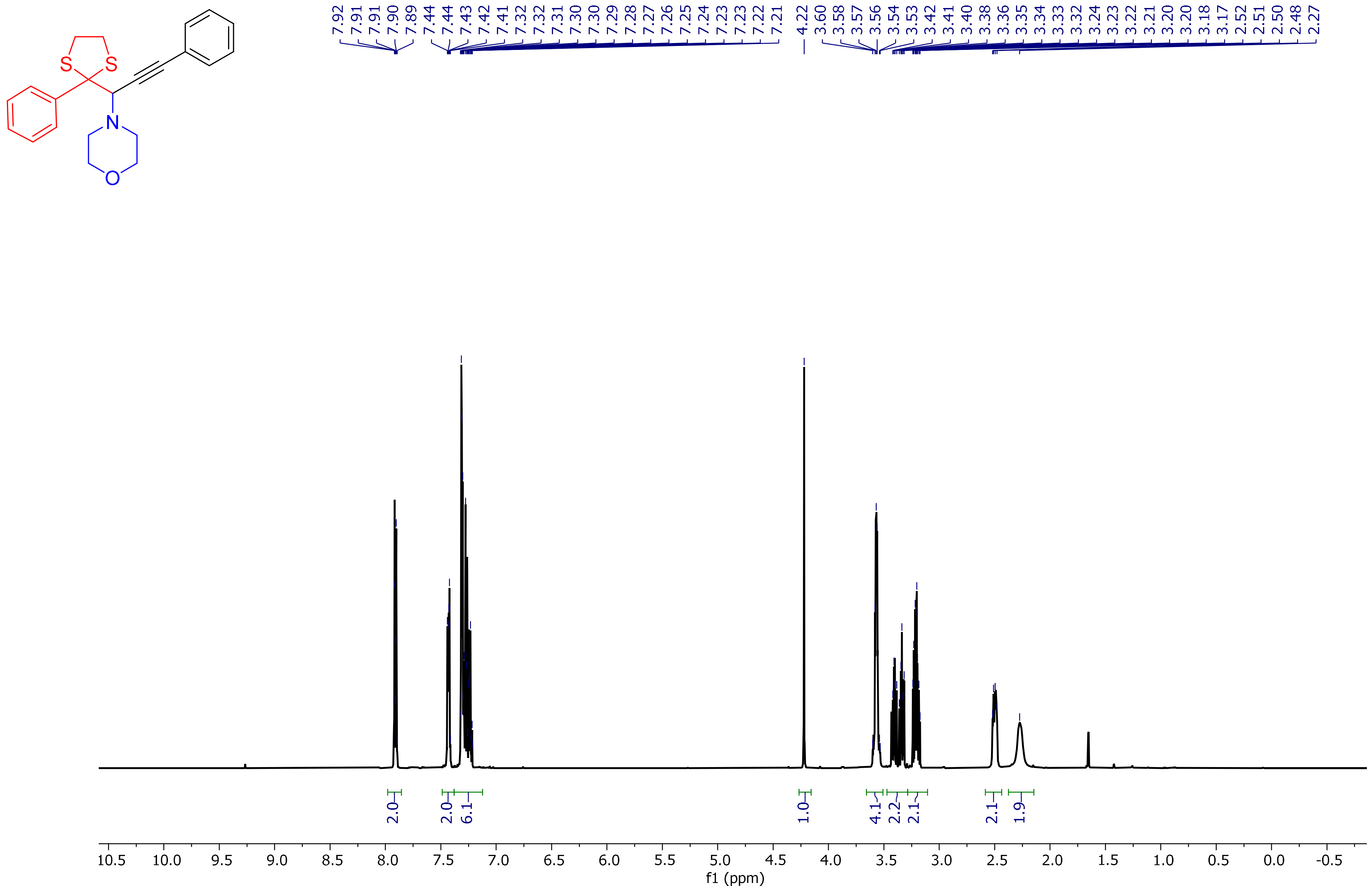

Figure S30. ^{13}C-NMR ($CDCl_3$, 125 MHz) spectrum **4a**

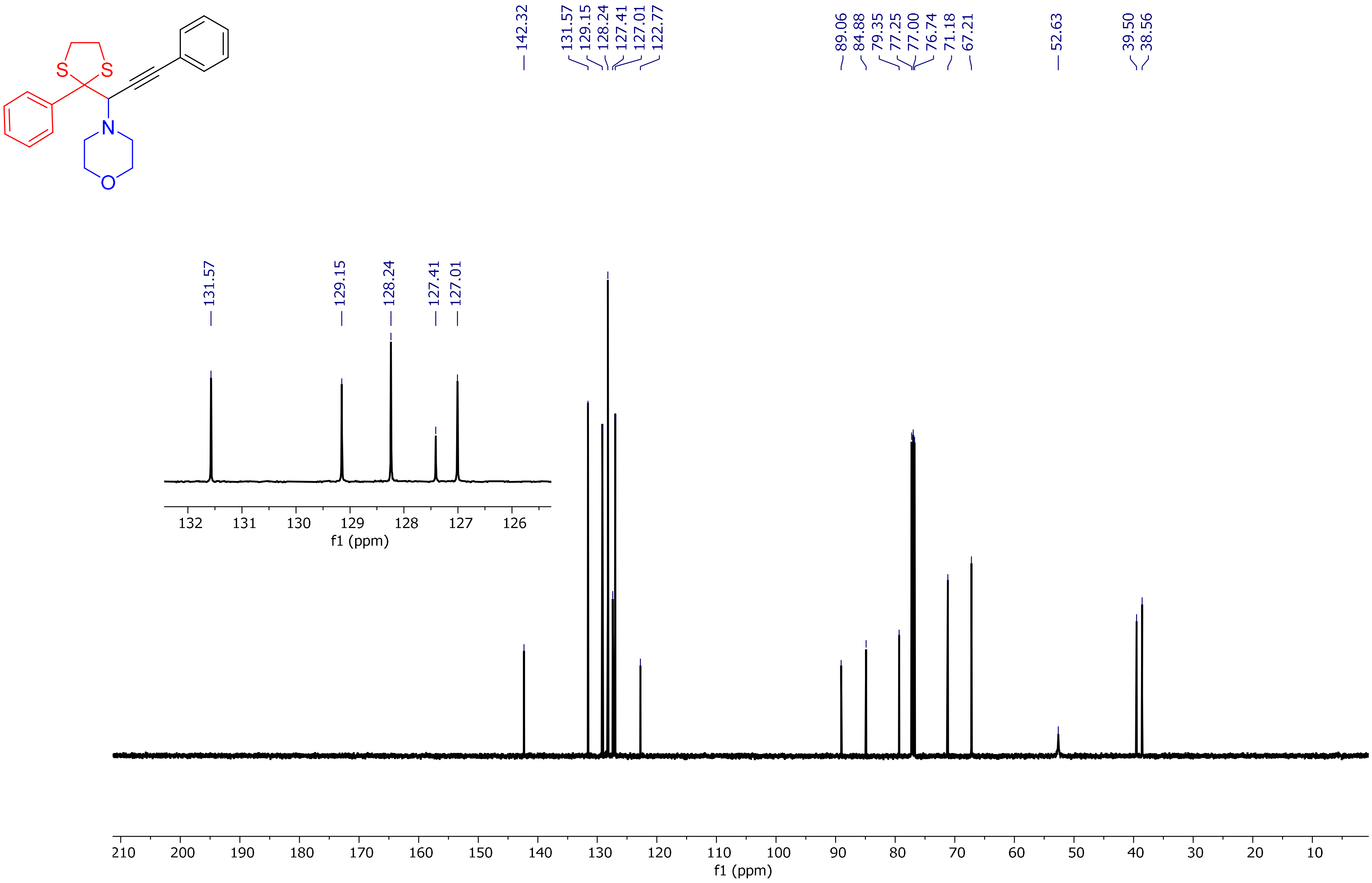

Figure S31. ^{1}H- NMR ($CDCl_3$, 500 MHz) spectrum **4b**

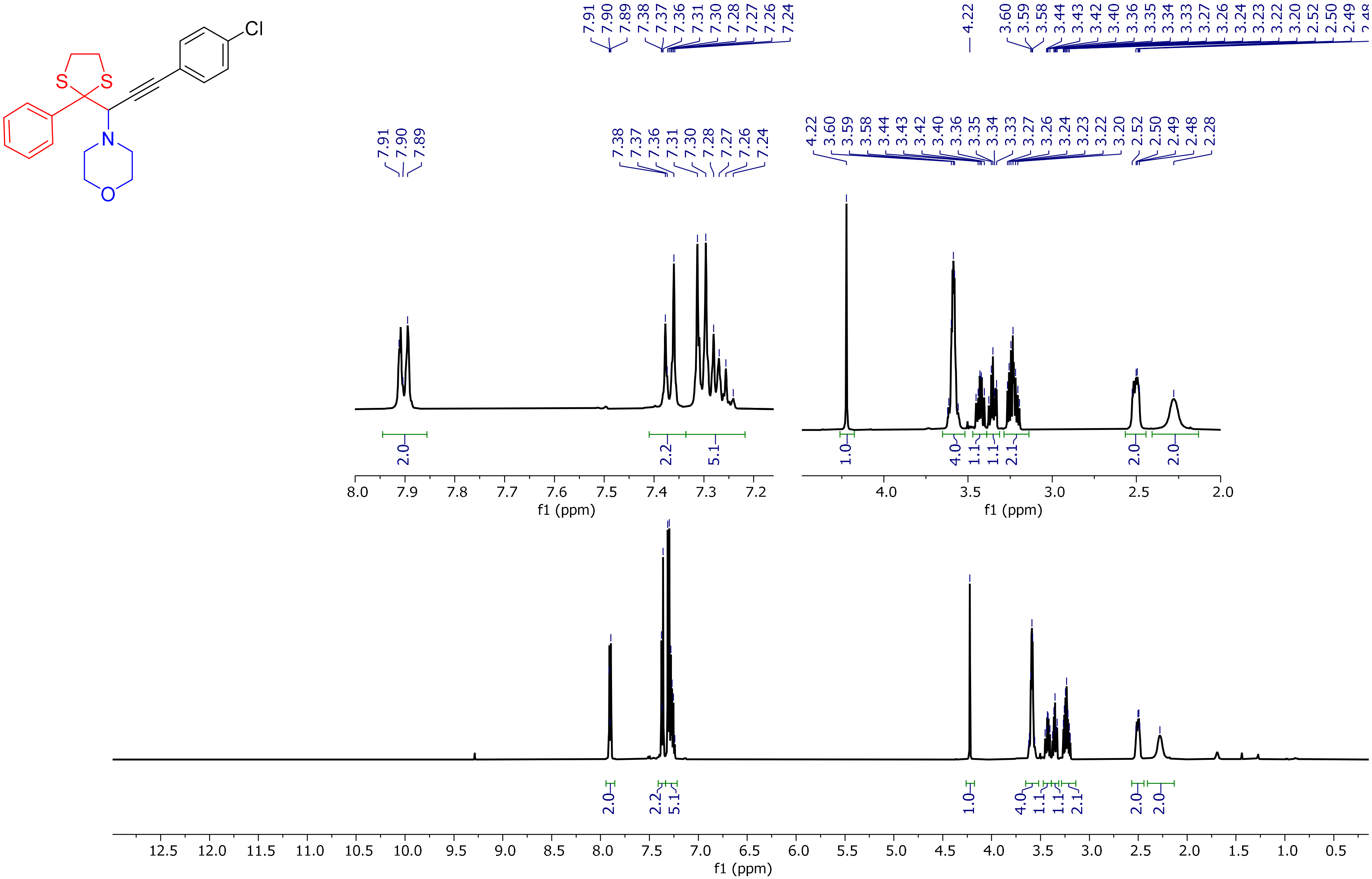

Figure S32. ^{13}C-NMR ($CDCl_3$, 125 MHz) spectrum **4b**

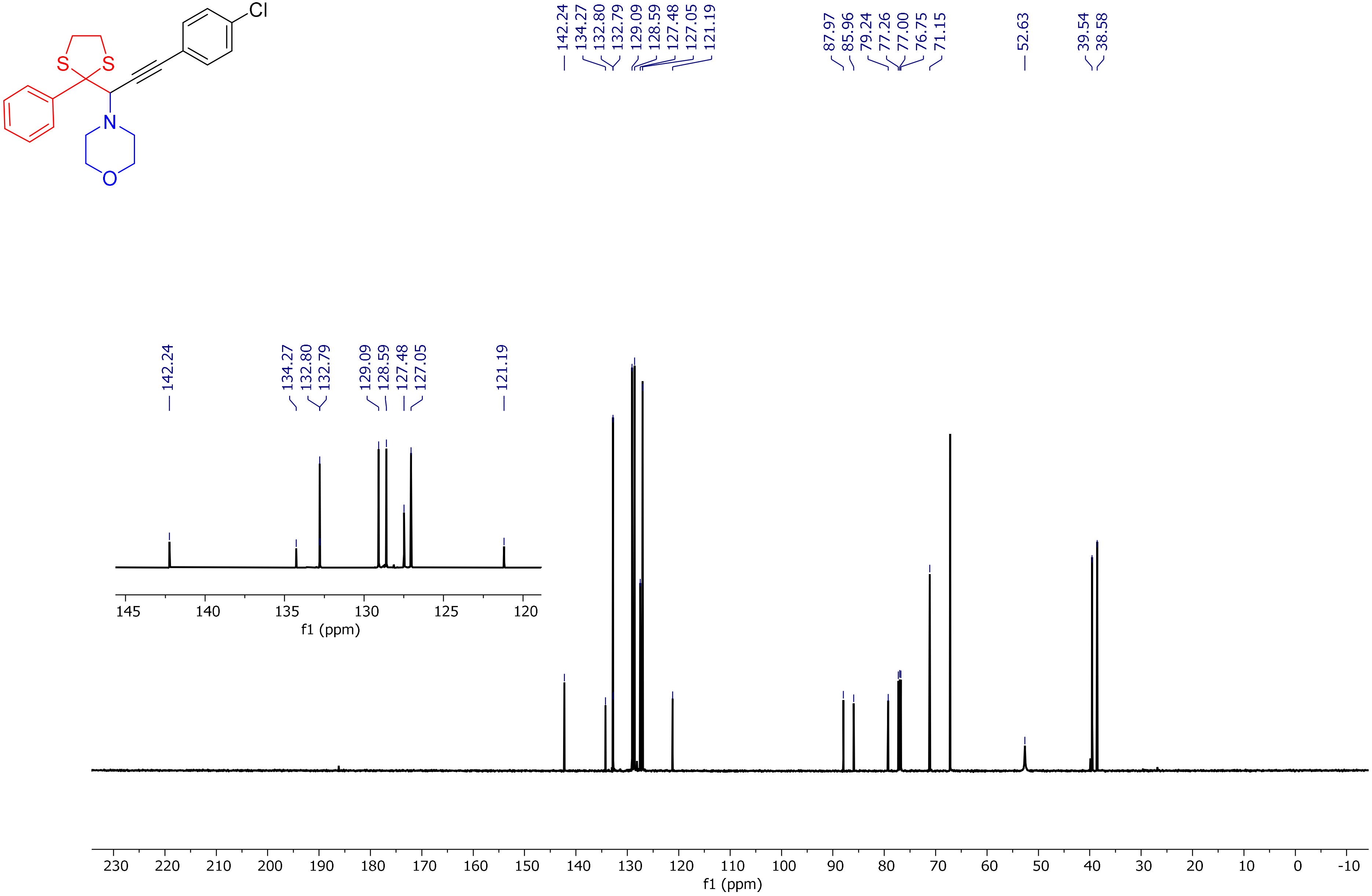

Figure S33. ^{13}C-APT NMR ($CDCl_3$, 125 MHz) spectrum **4b**

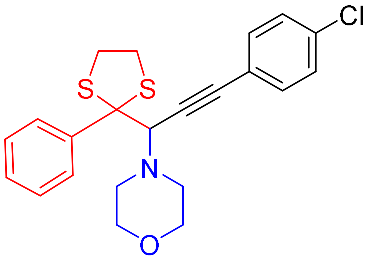

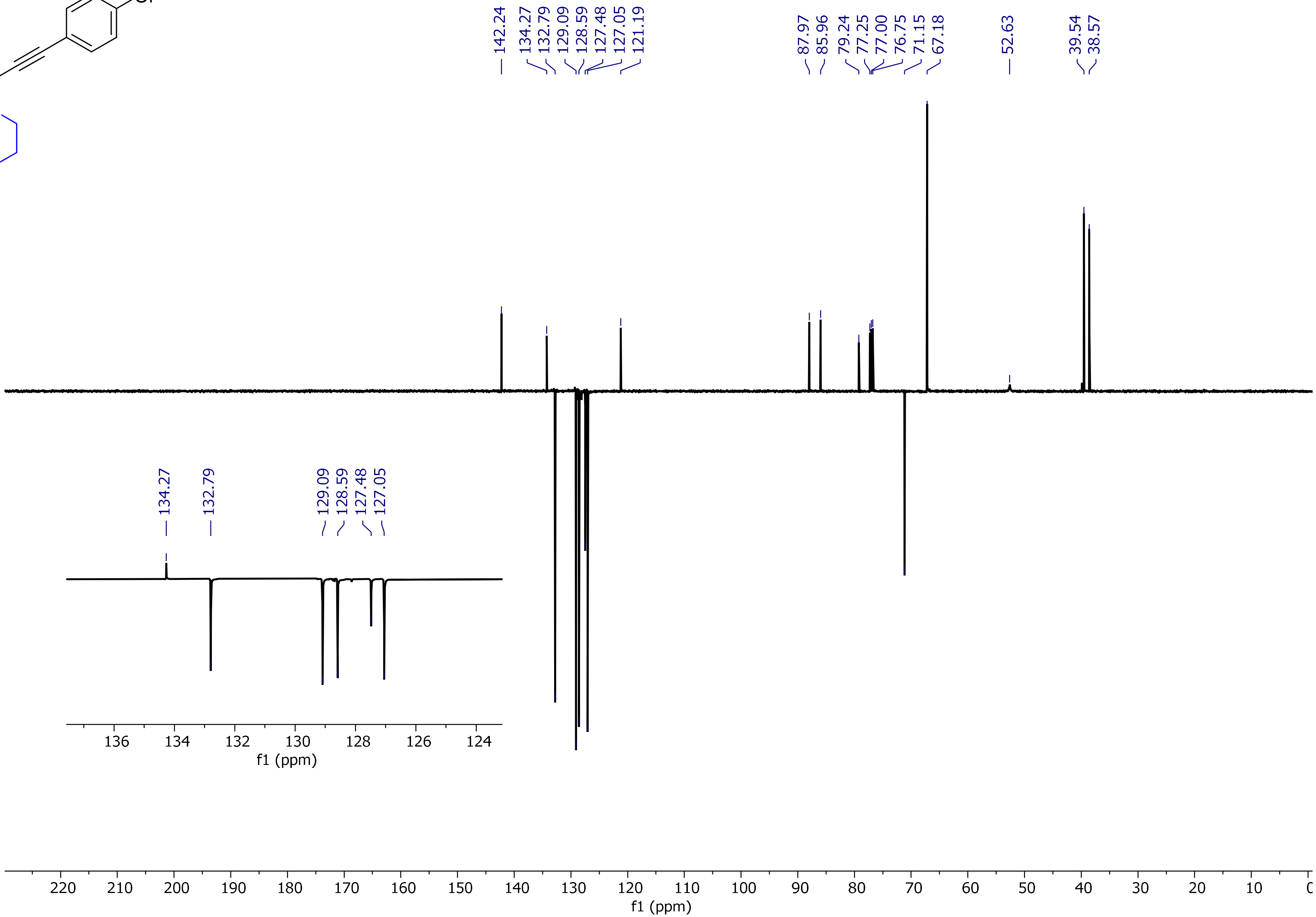

Figure S34. ^{1}H- NMR ($CDCl_3$, 500 MHz) spectrum **4c**

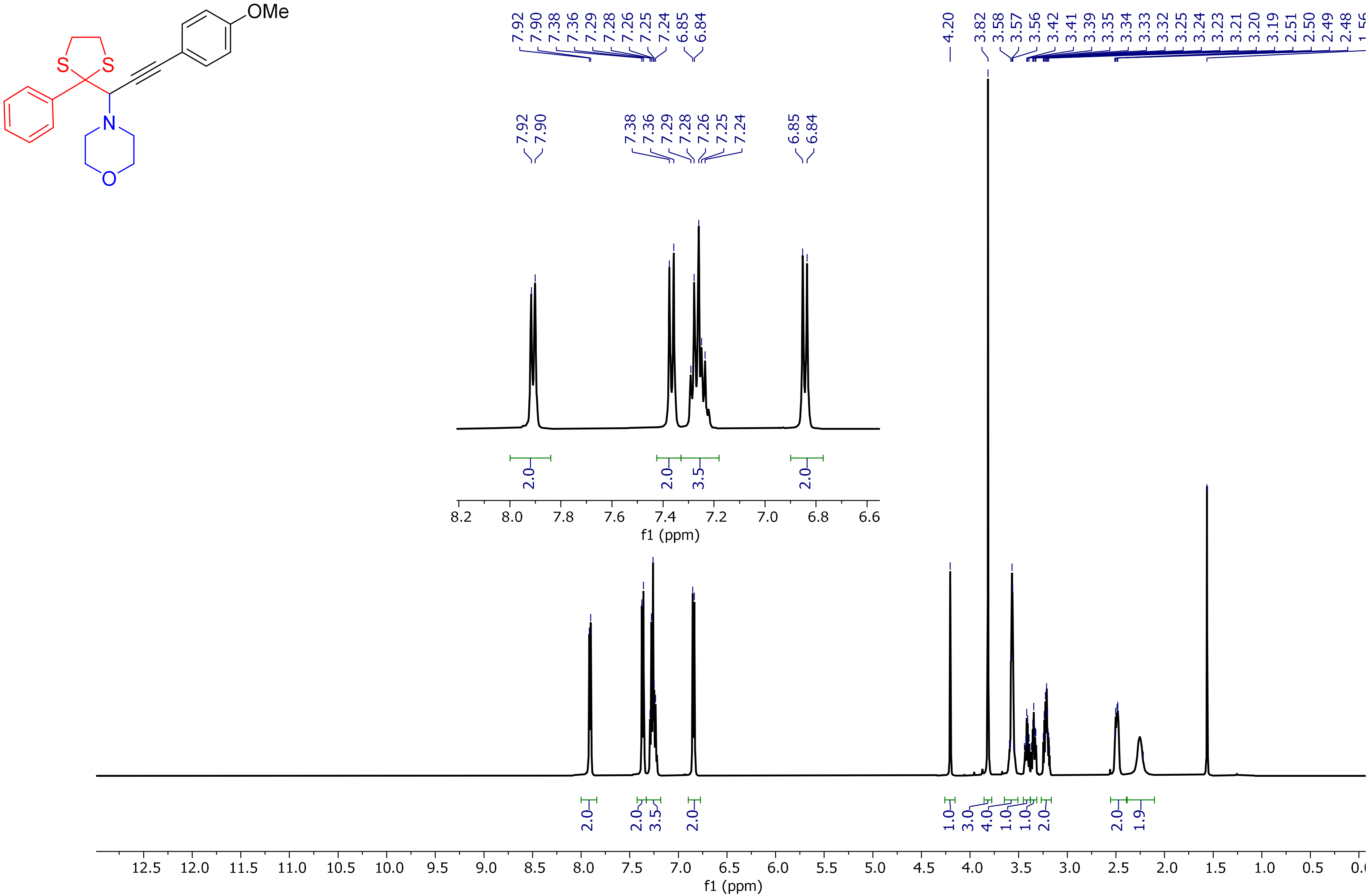

Figure S35. ^{13}C- NMR ($CDCl_3$, 125 MHz) spectrum **4c**

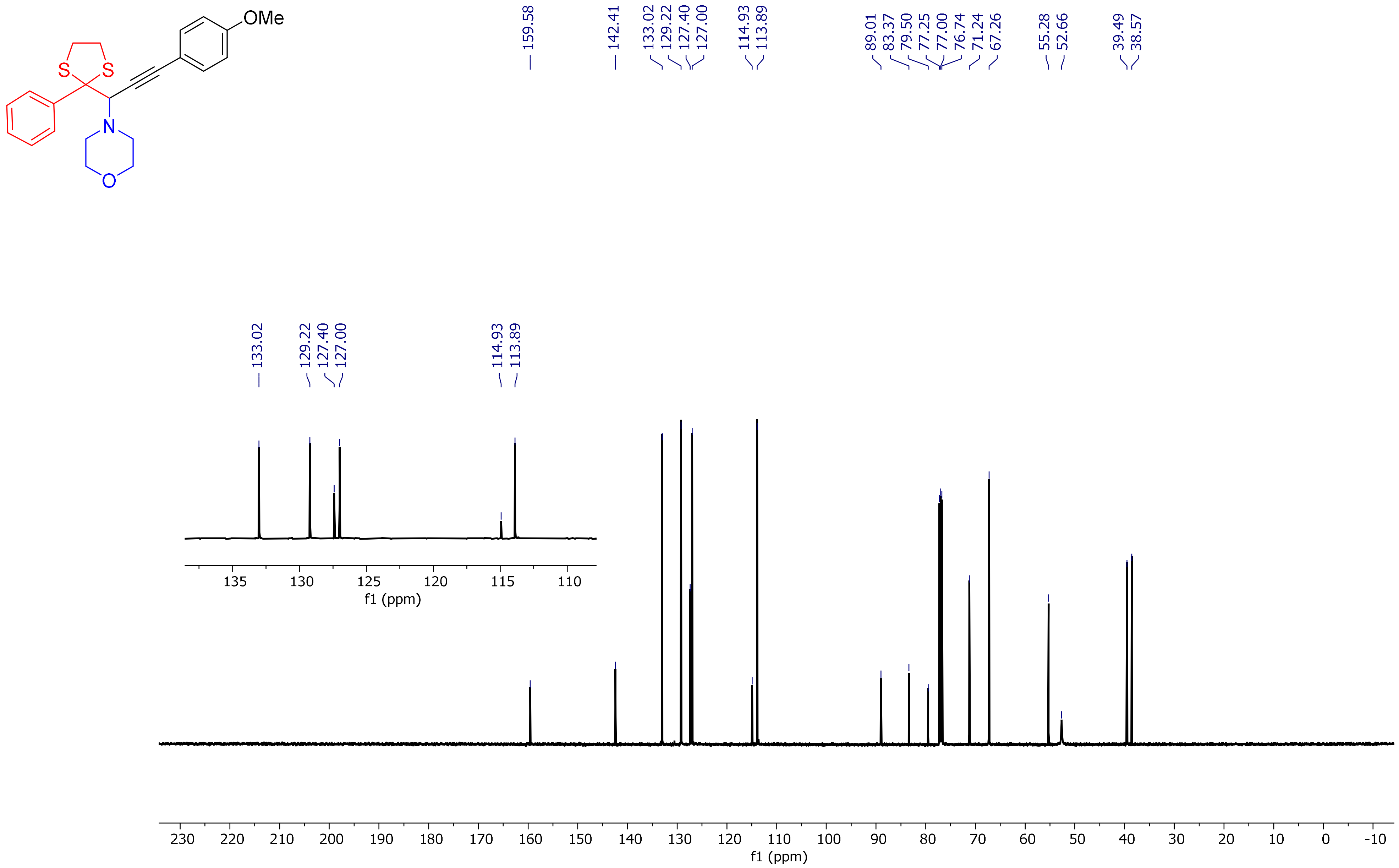

Figure S36. ^{13}C-APT NMR ($CDCl_3$, 125 MHz) spectrum **4c**

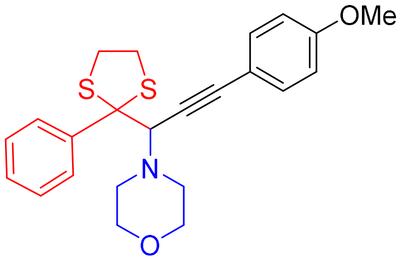

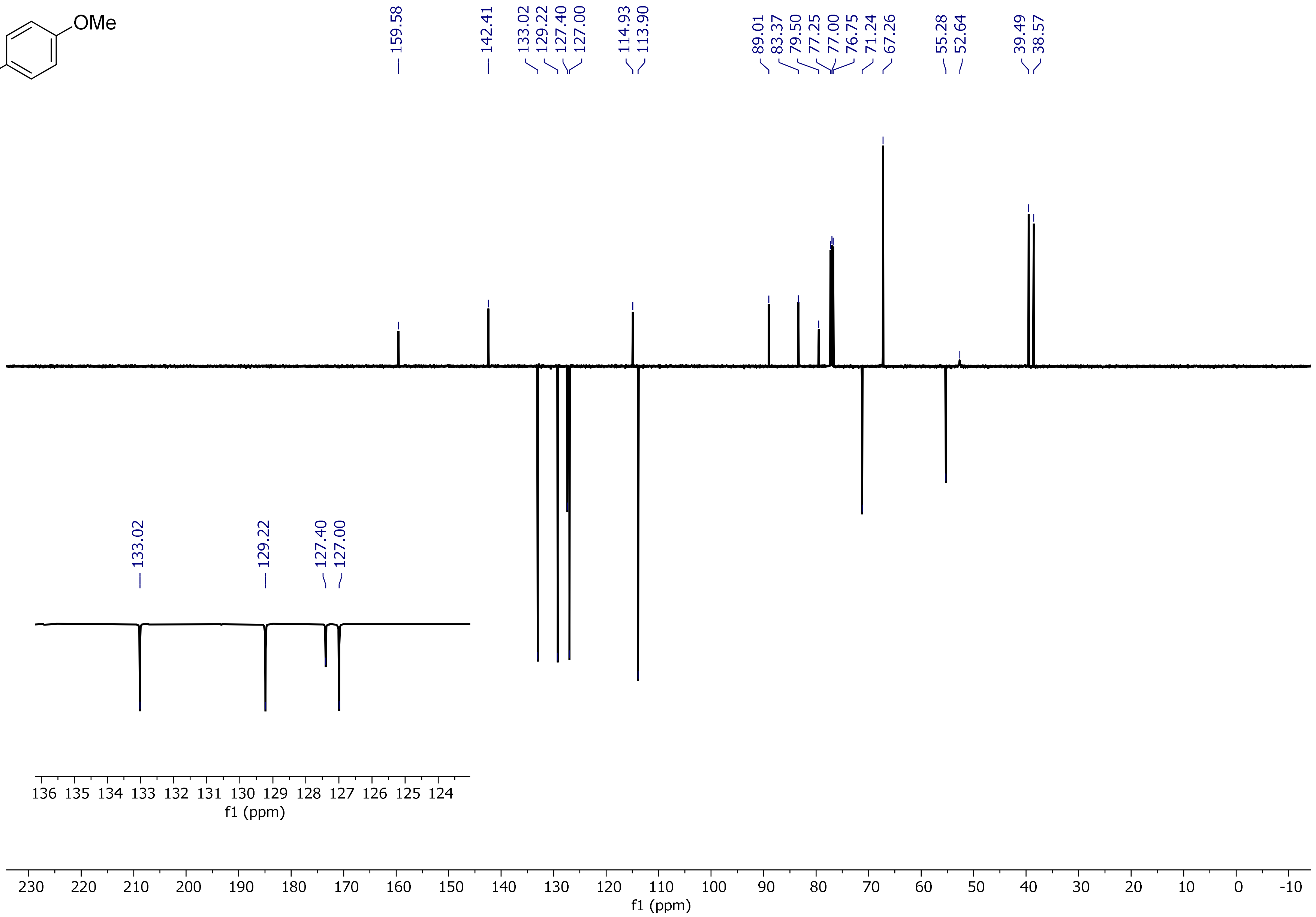

Figure S37. ^{1}H- NMR ($CDCl_3$, 500 MHz) spectrum **4d**

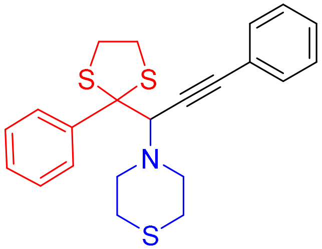

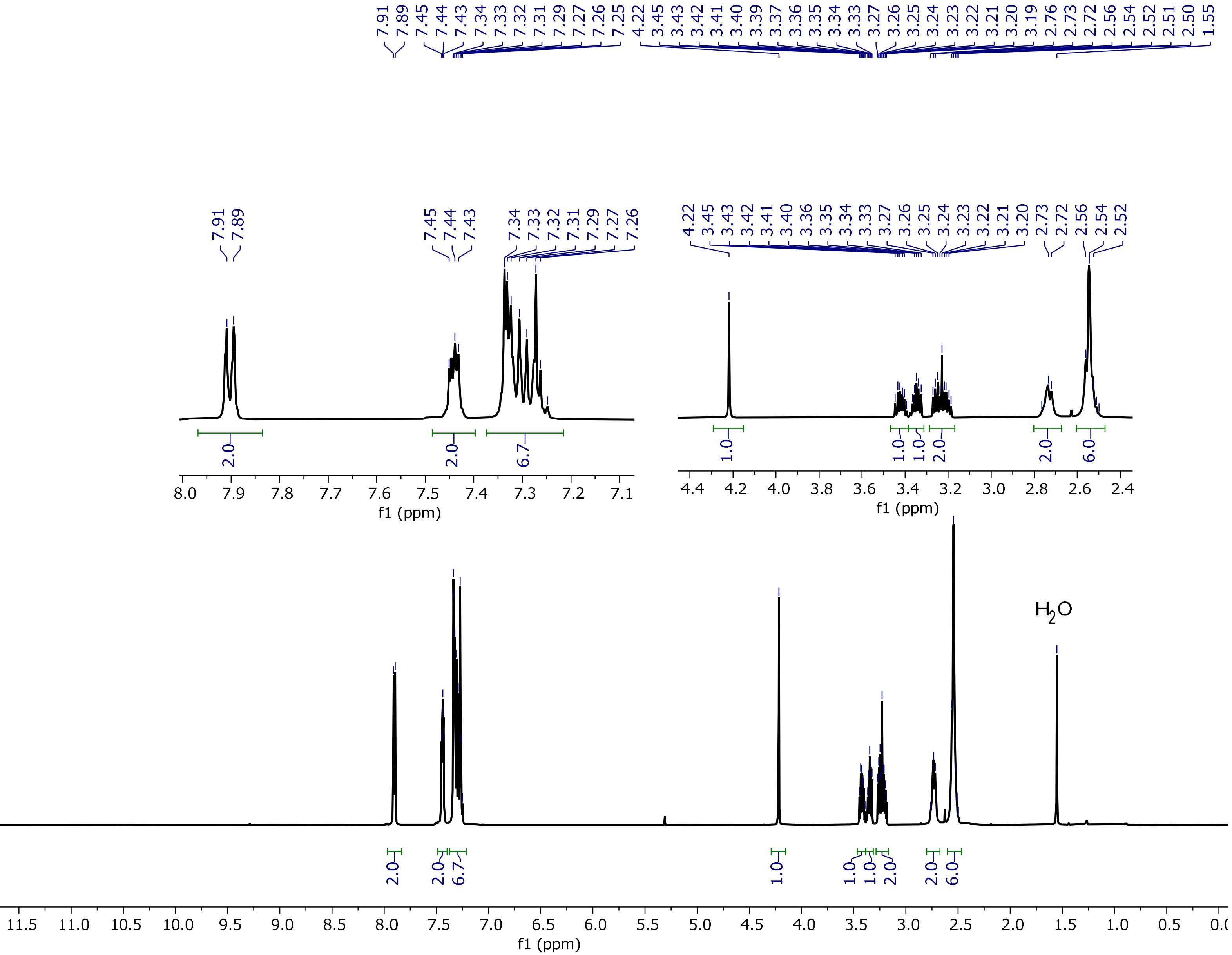

Figure S38. ^{13}C- NMR ($CDCl_3$, 125 MHz) spectrum **4d**

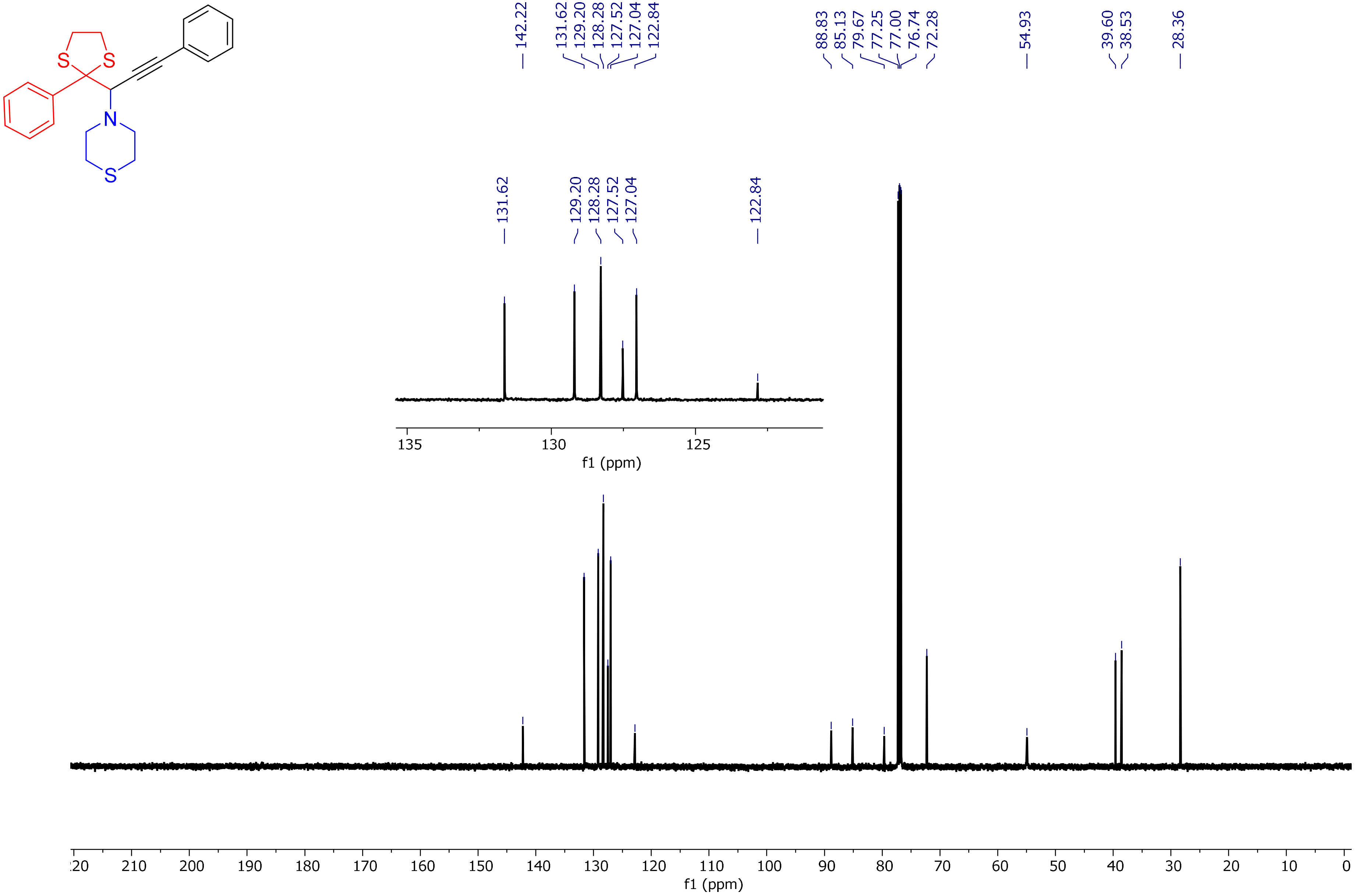

Figure S39. ^{13}C-APT NMR ($CDCl_3$, 125 MHz) spectrum **4d**

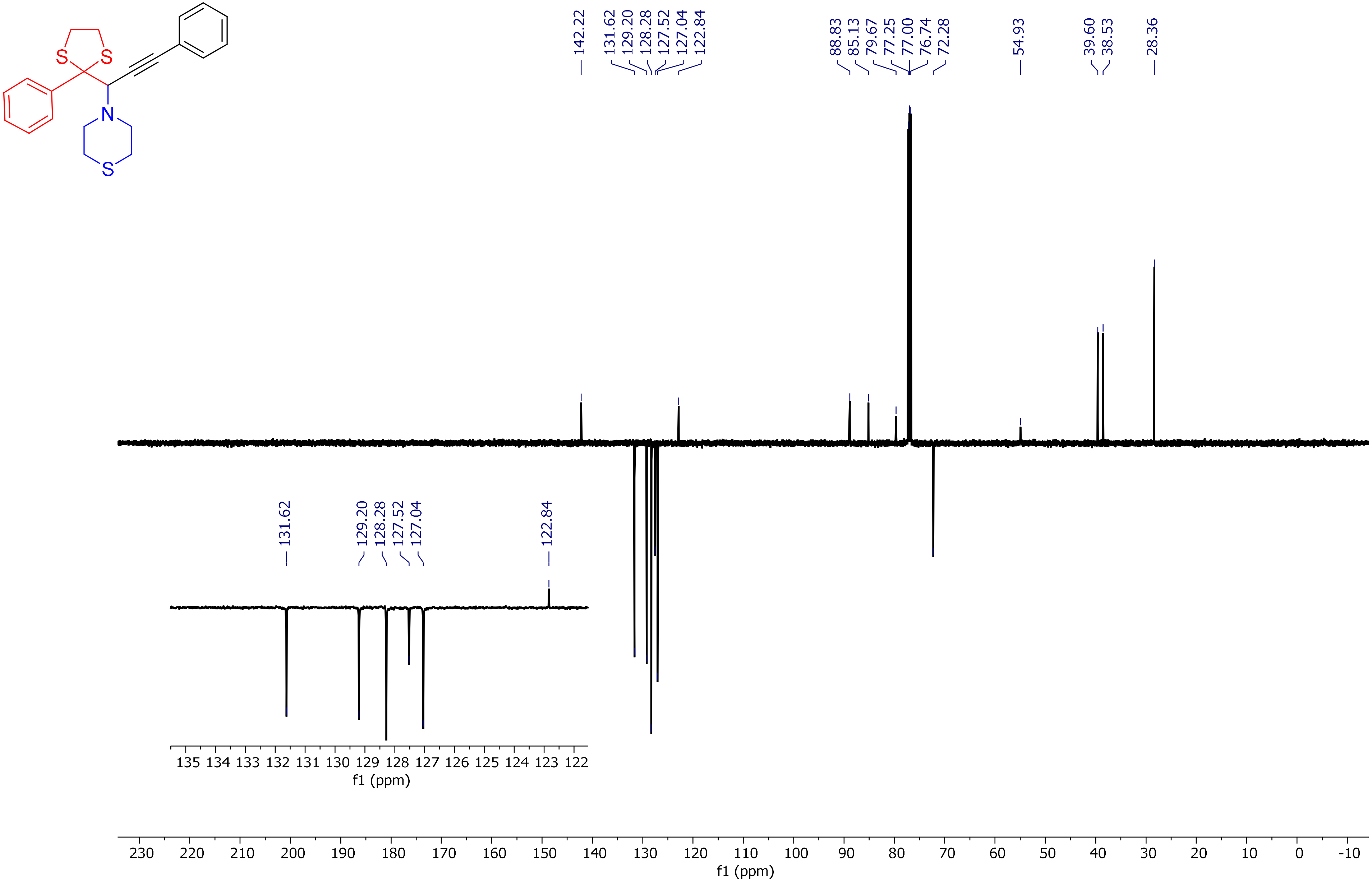

Figure S40. ^{1}H- NMR (CDCl$_3$, 500 MHz) spectrum **4e**

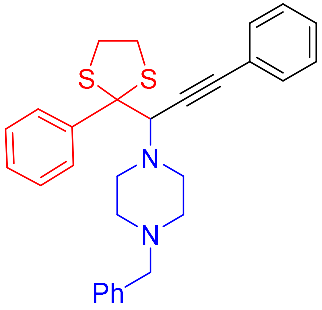

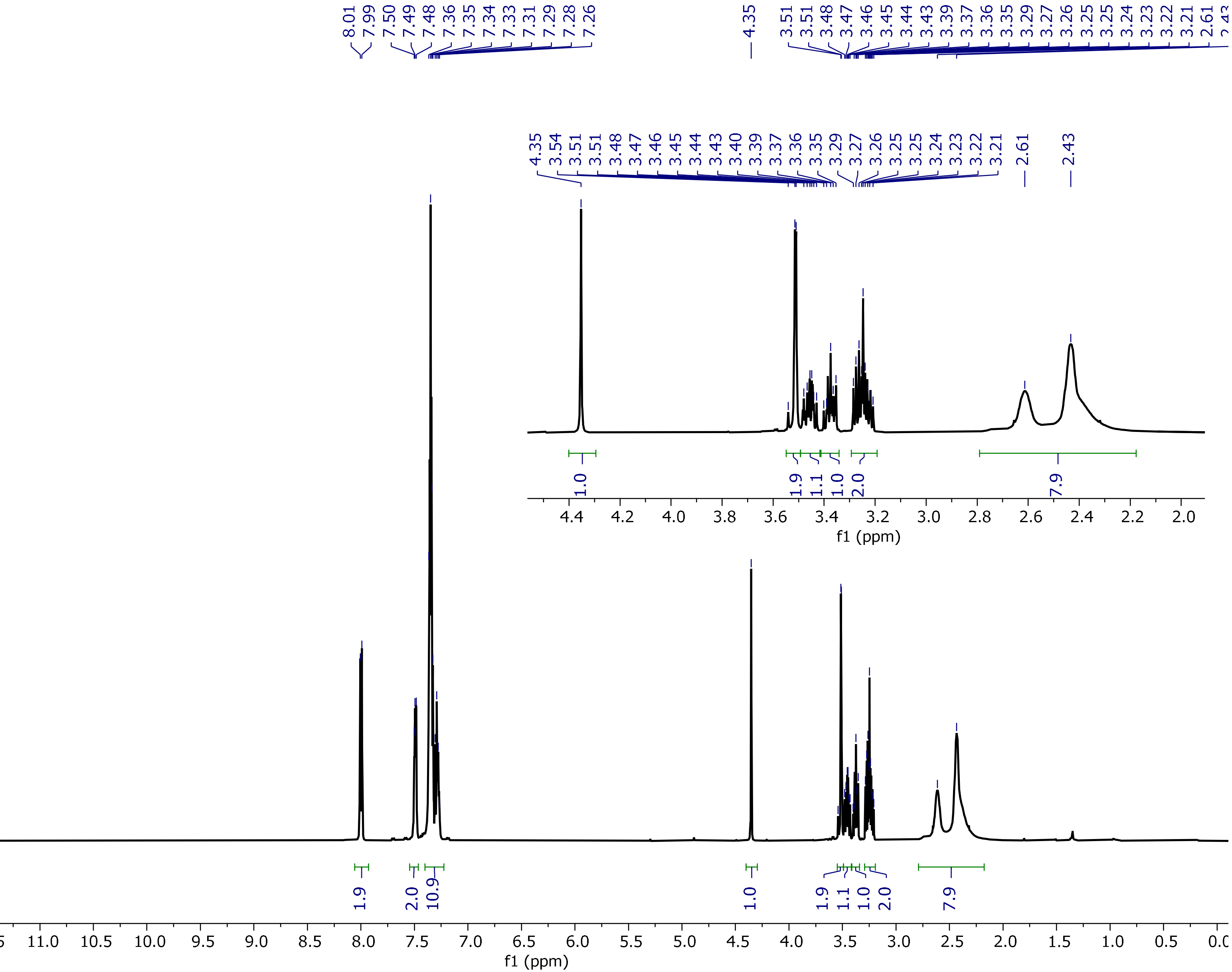

Figure S41. ^{13}C- NMR ($CDCl_3$, 125 MHz) spectrum **4e**

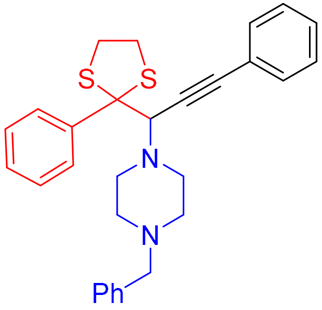

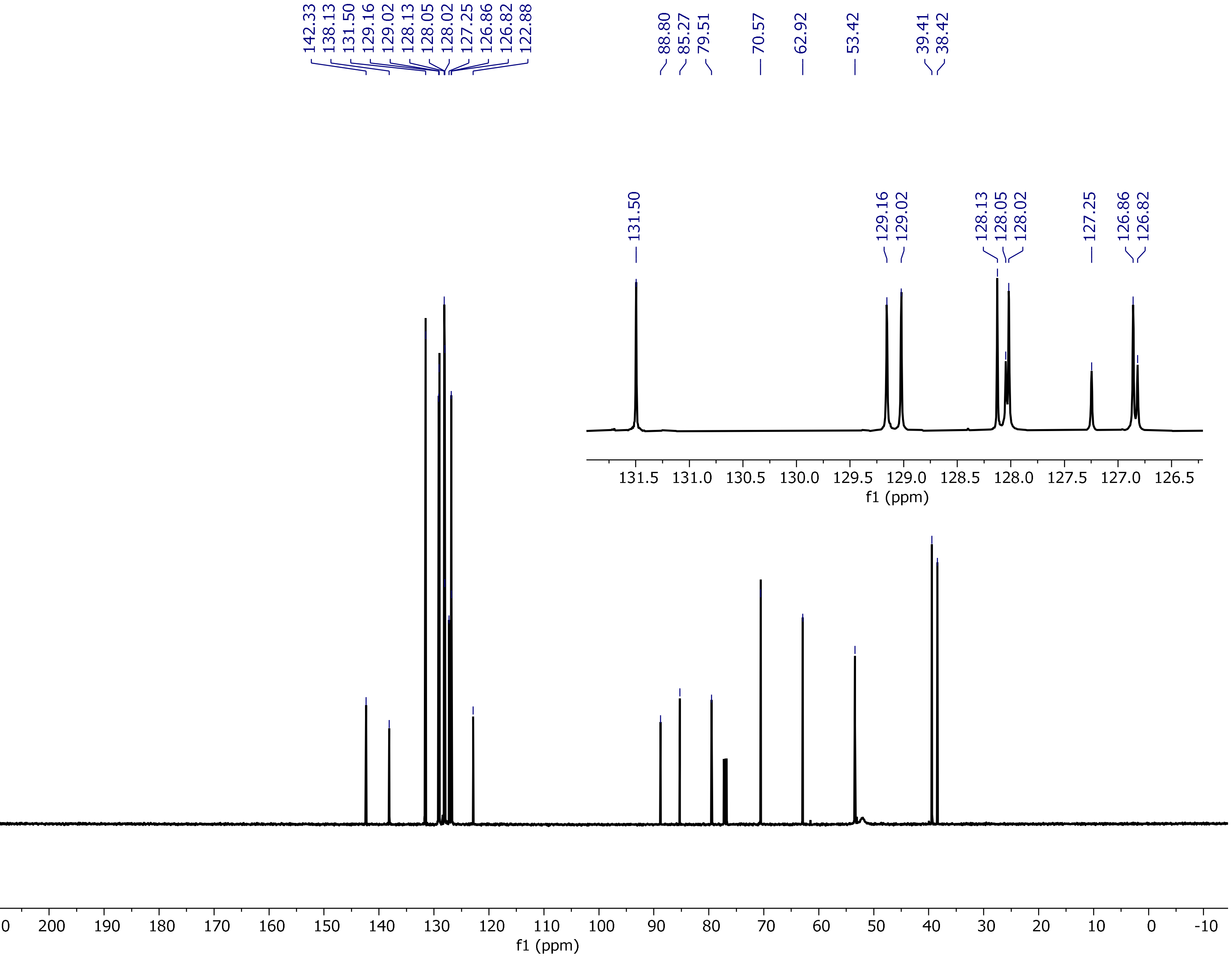

Figure S42. ^{13}C-APT NMR ($CDCl_3$, 125 MHz) spectrum **4e**

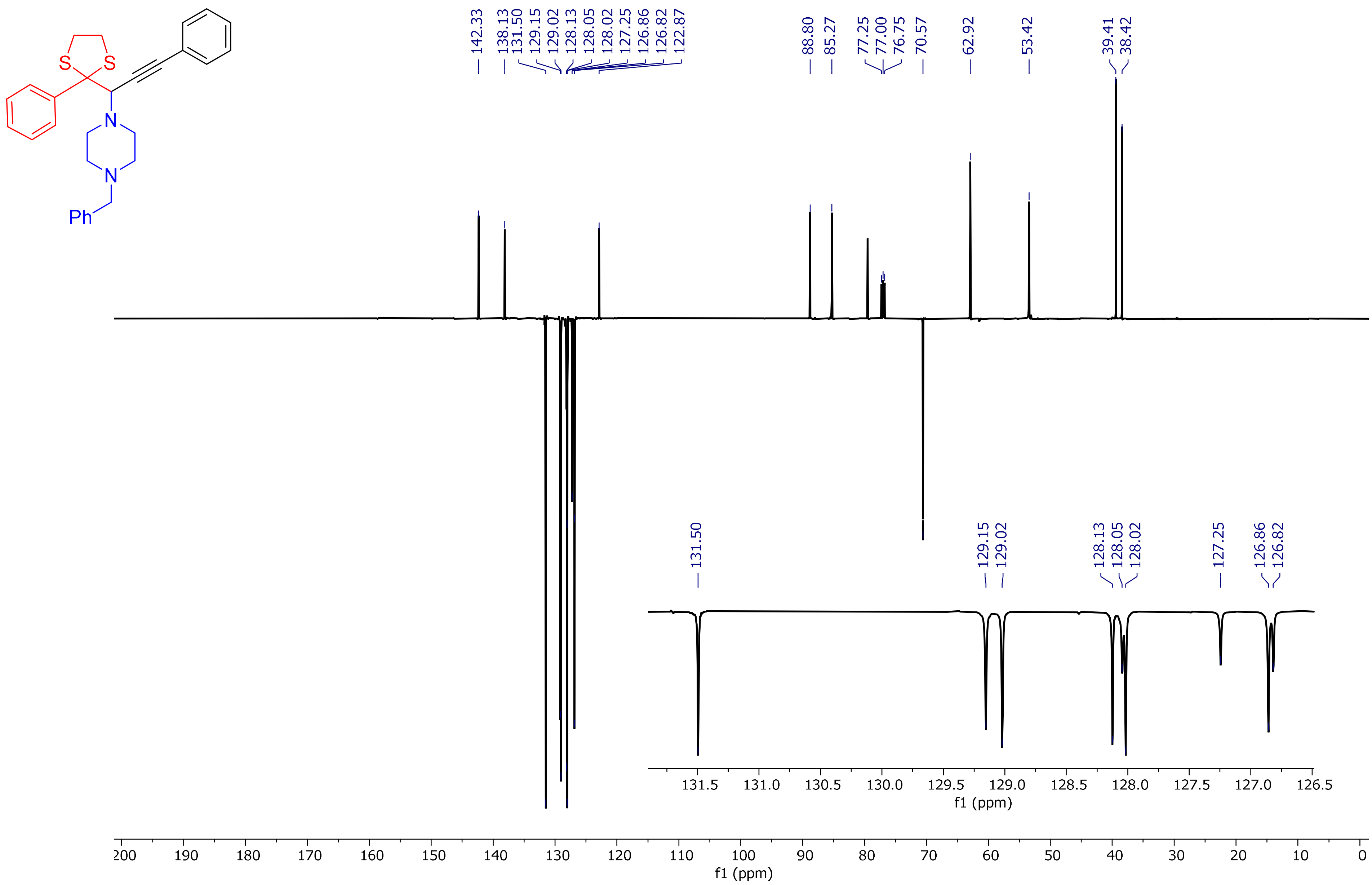

Figure S43. ¹H- NMR ($CDCl_3$, 500 MHz) spectrum **6a**

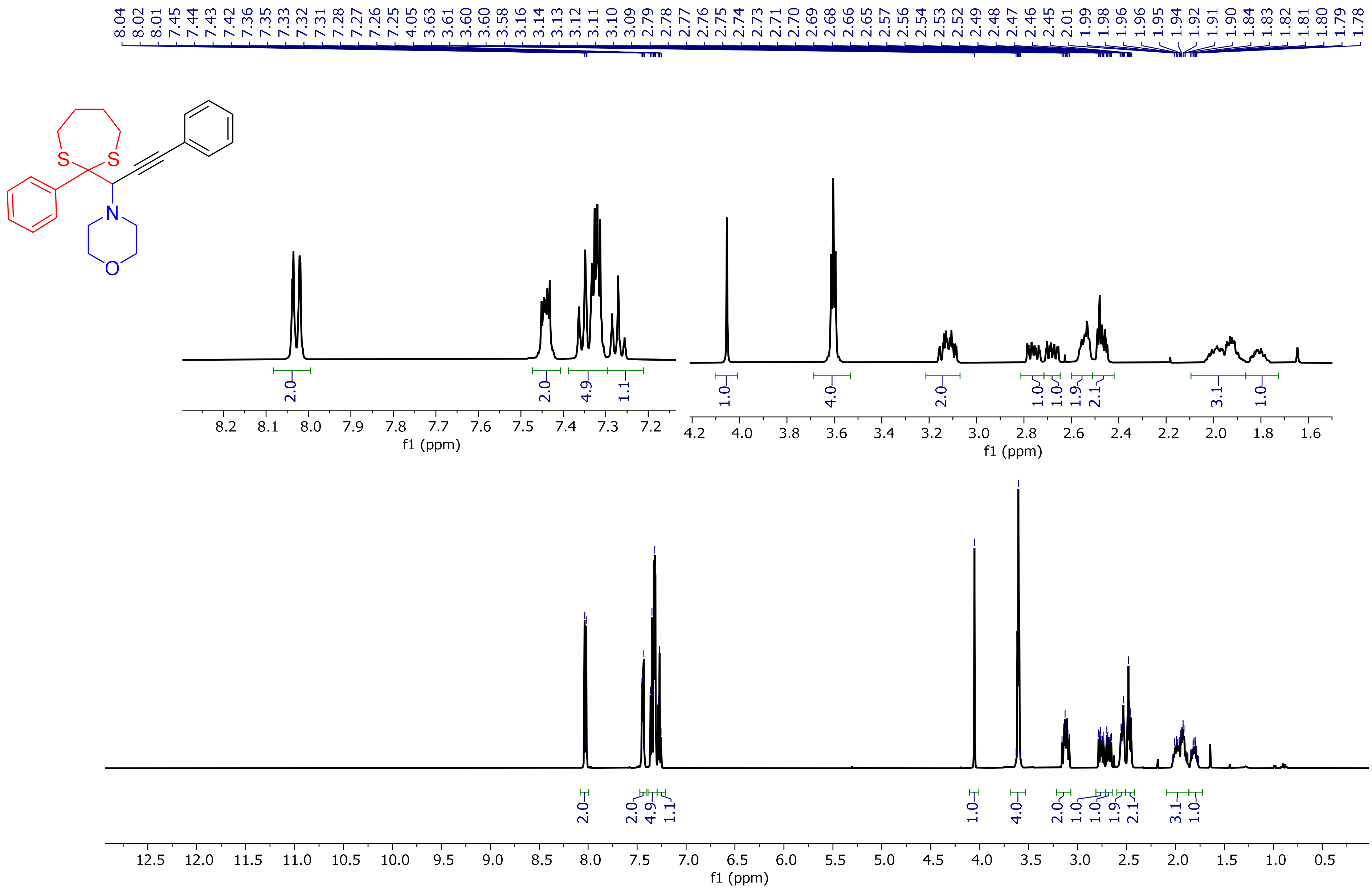

Figure S44. ^{13}C-APT NMR ($CDCl_3$, 125 MHz) spectrum **6a**

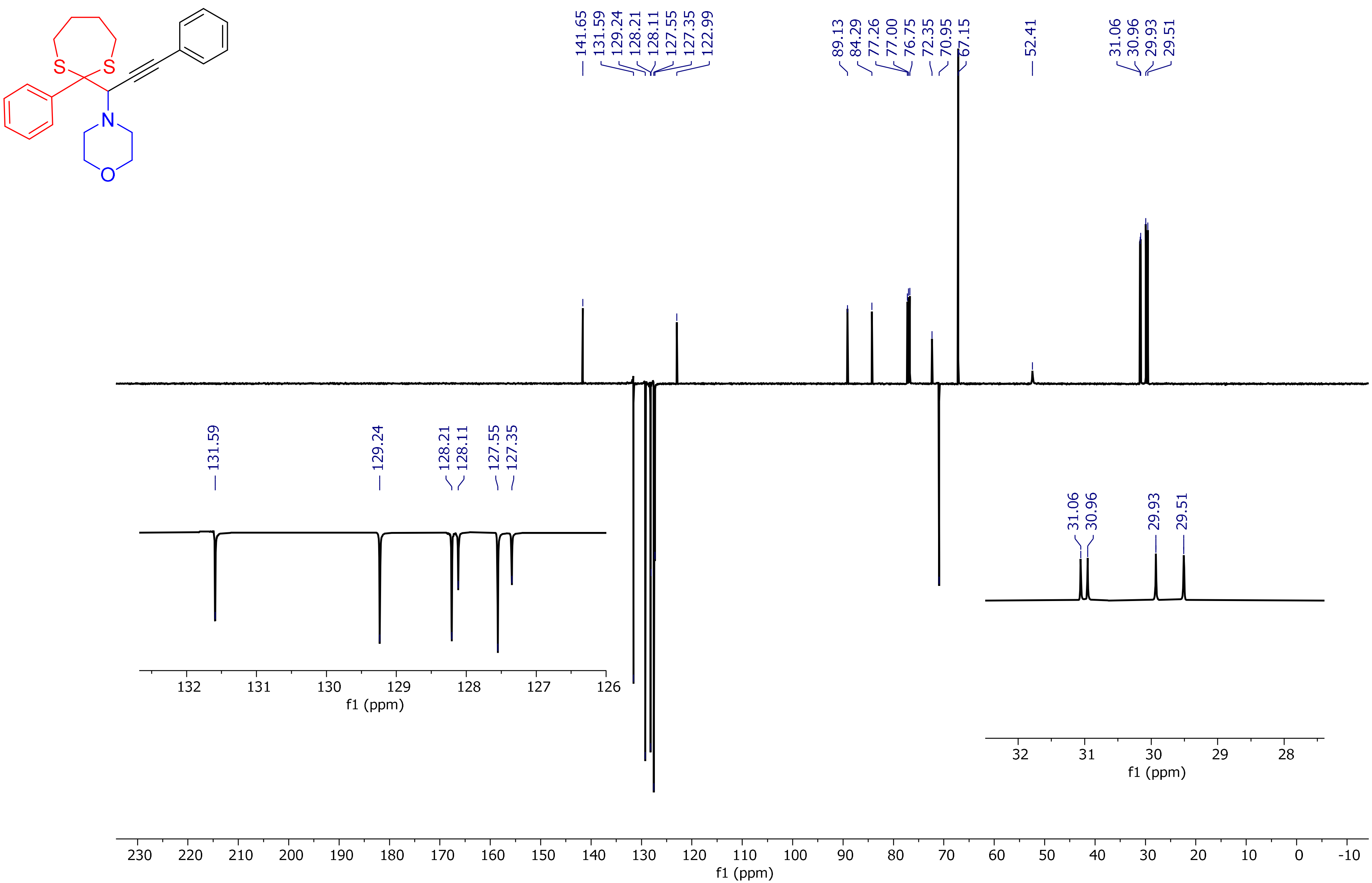

Figure S45. ^{1}H- NMR ($CDCl_3$, 500 MHz) spectrum **6b**

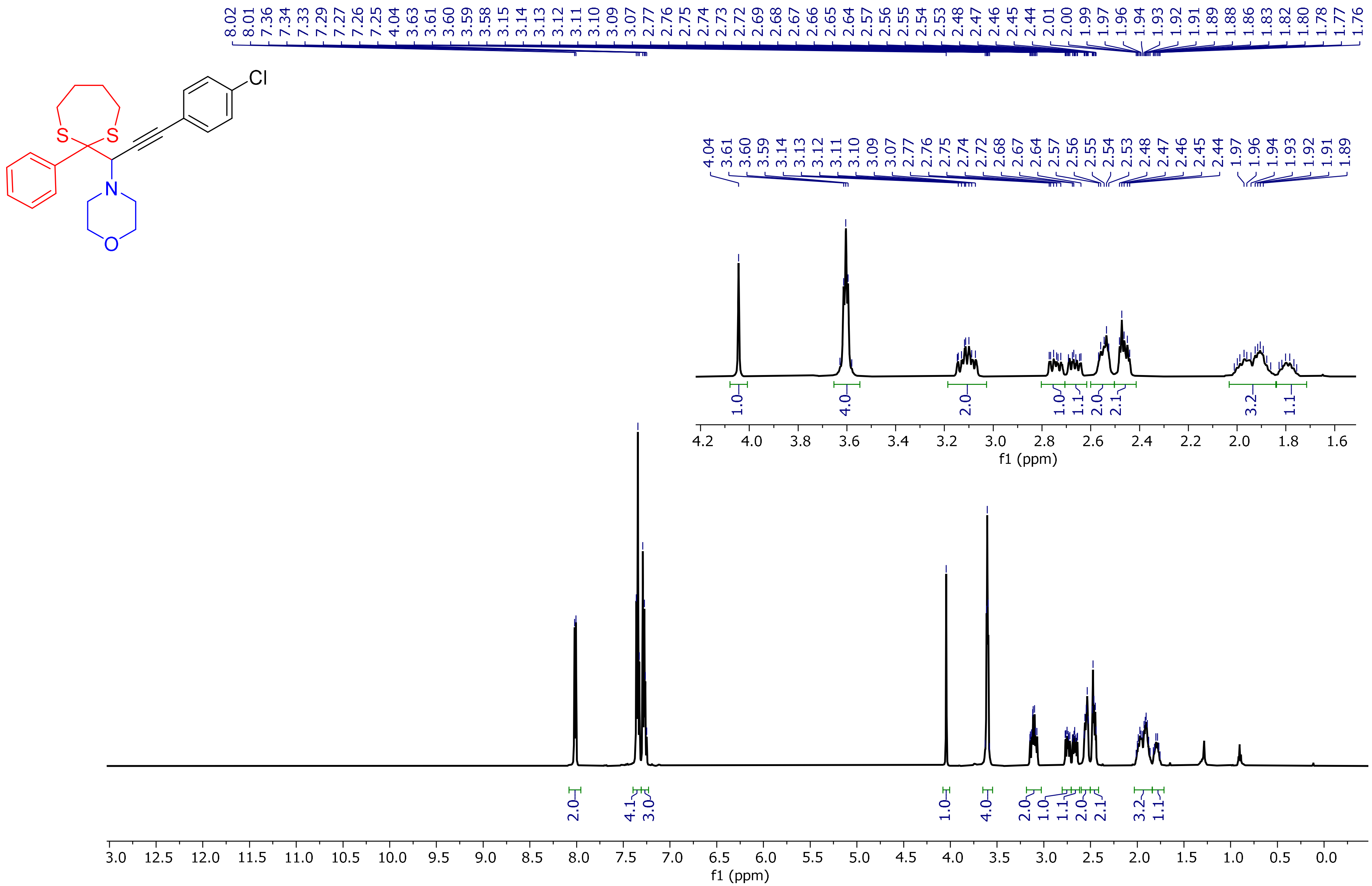

Figure S46. ^{13}C- NMR (CDCl$_3$, 125 MHz) spectrum **6b**

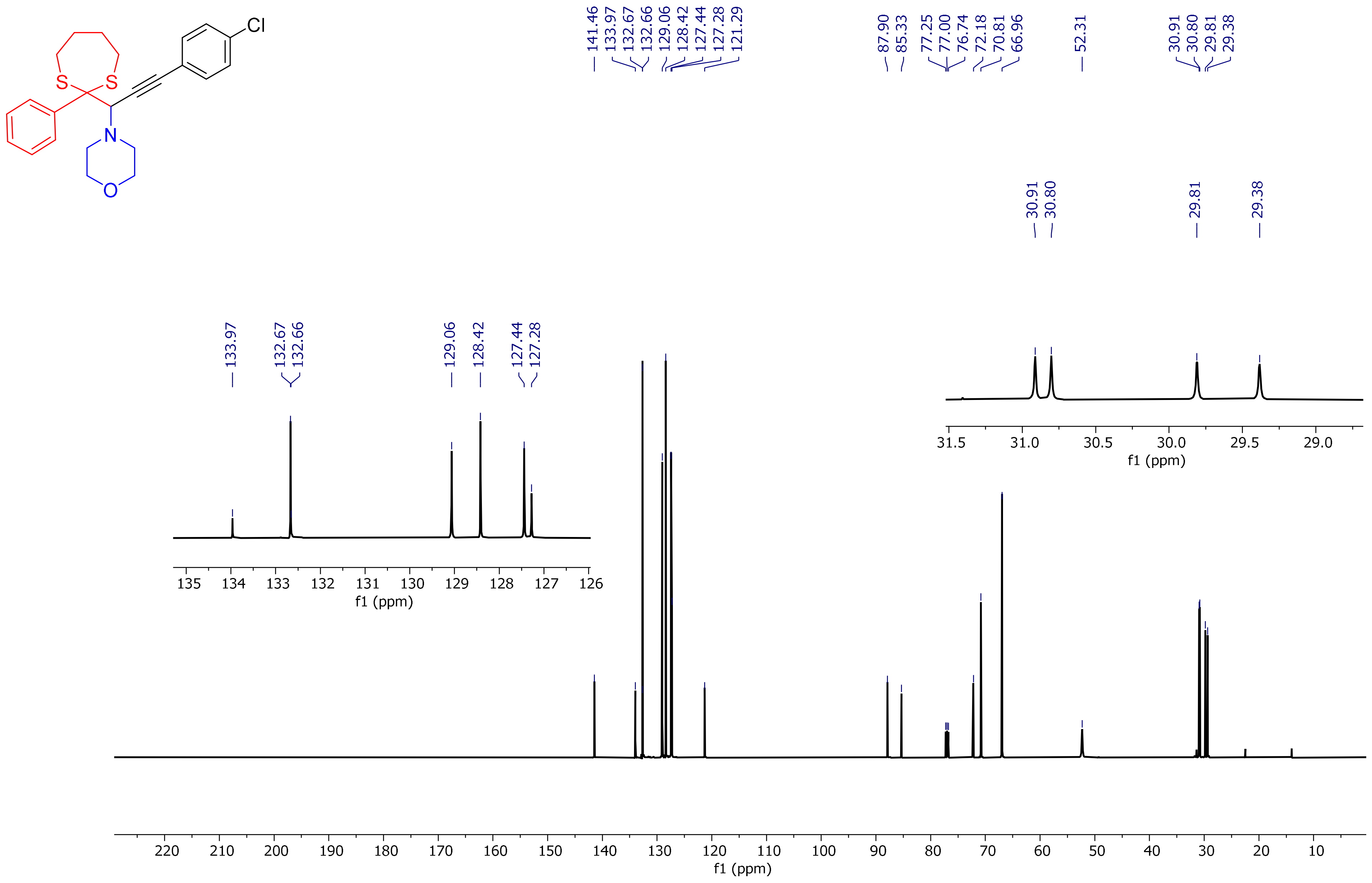

Figure S47. ^{13}C-APT NMR ($CDCl_3$, 125 MHz) spectrum **6b**

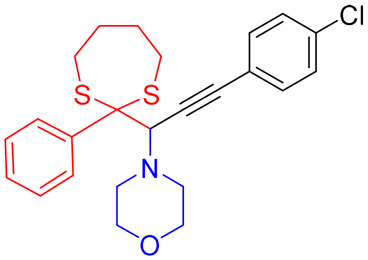

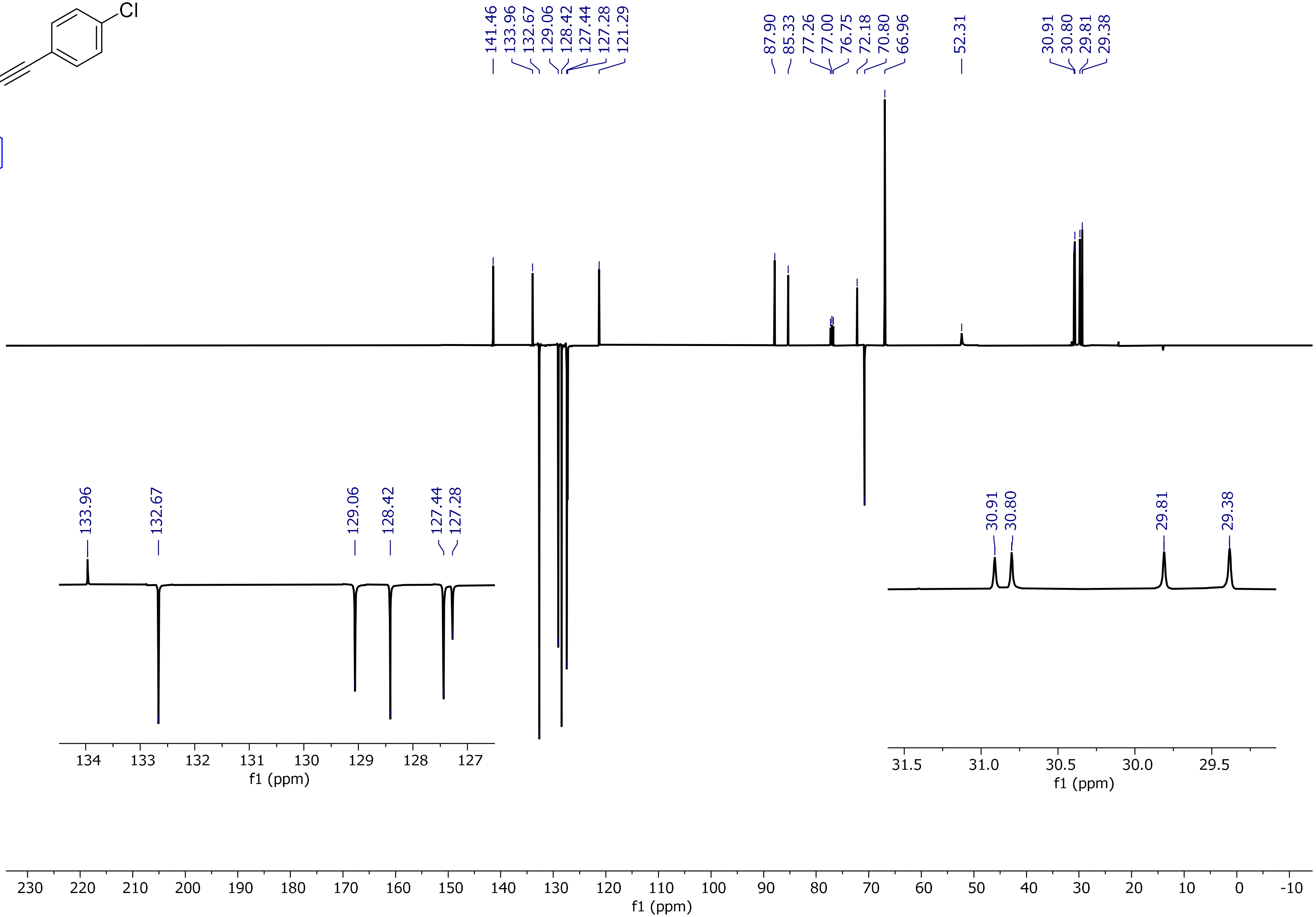

Figure S48. ^{1}H- NMR ($CDCl_3$, 500 MHz) spectrum **6c**

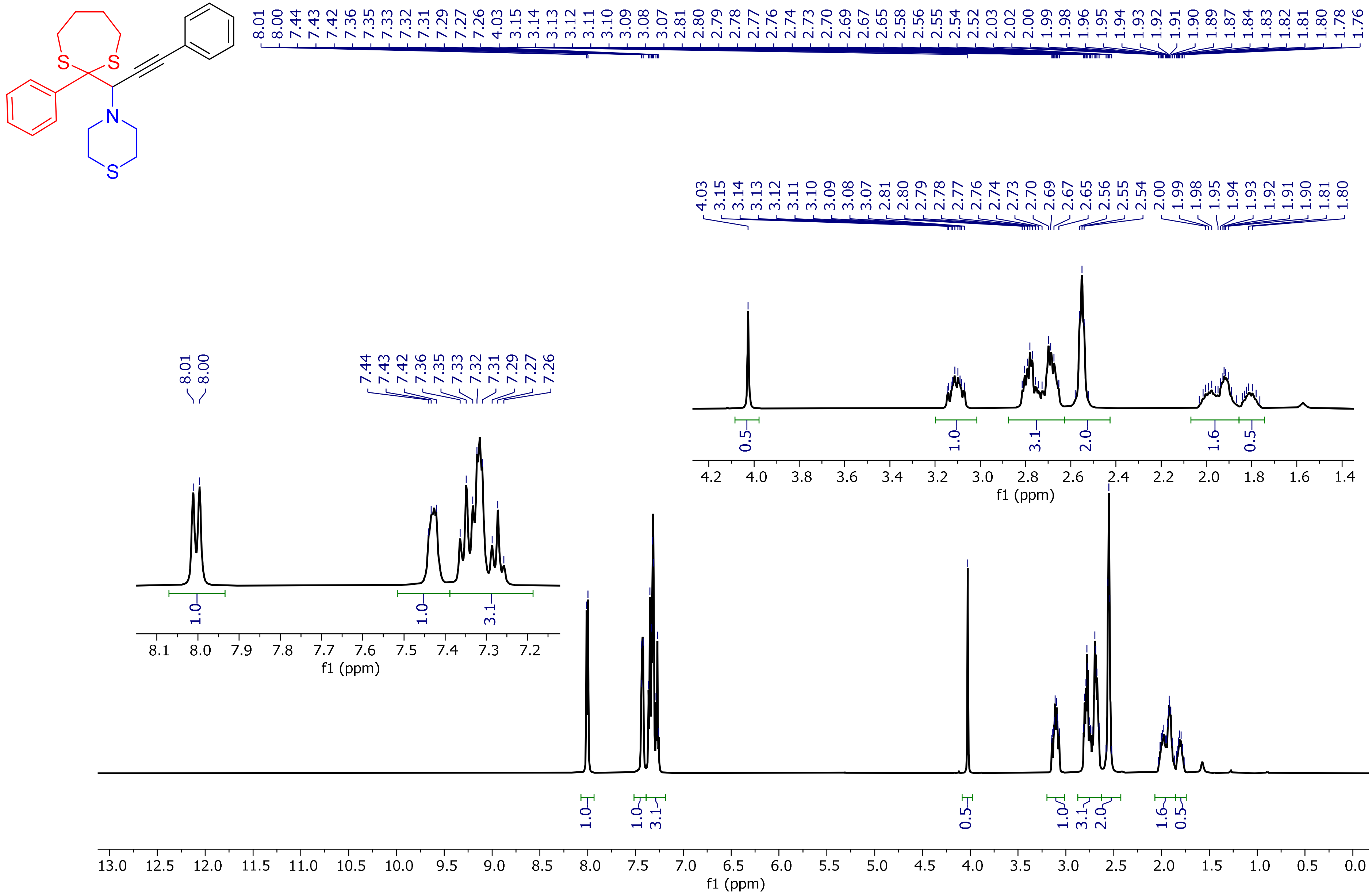

Figure S49. ^{13}C- NMR (CDCl$_3$, 125 MHz) spectrum **6c**

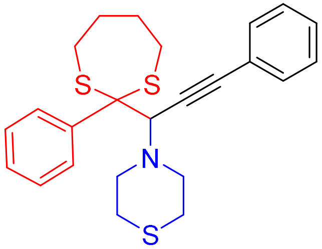

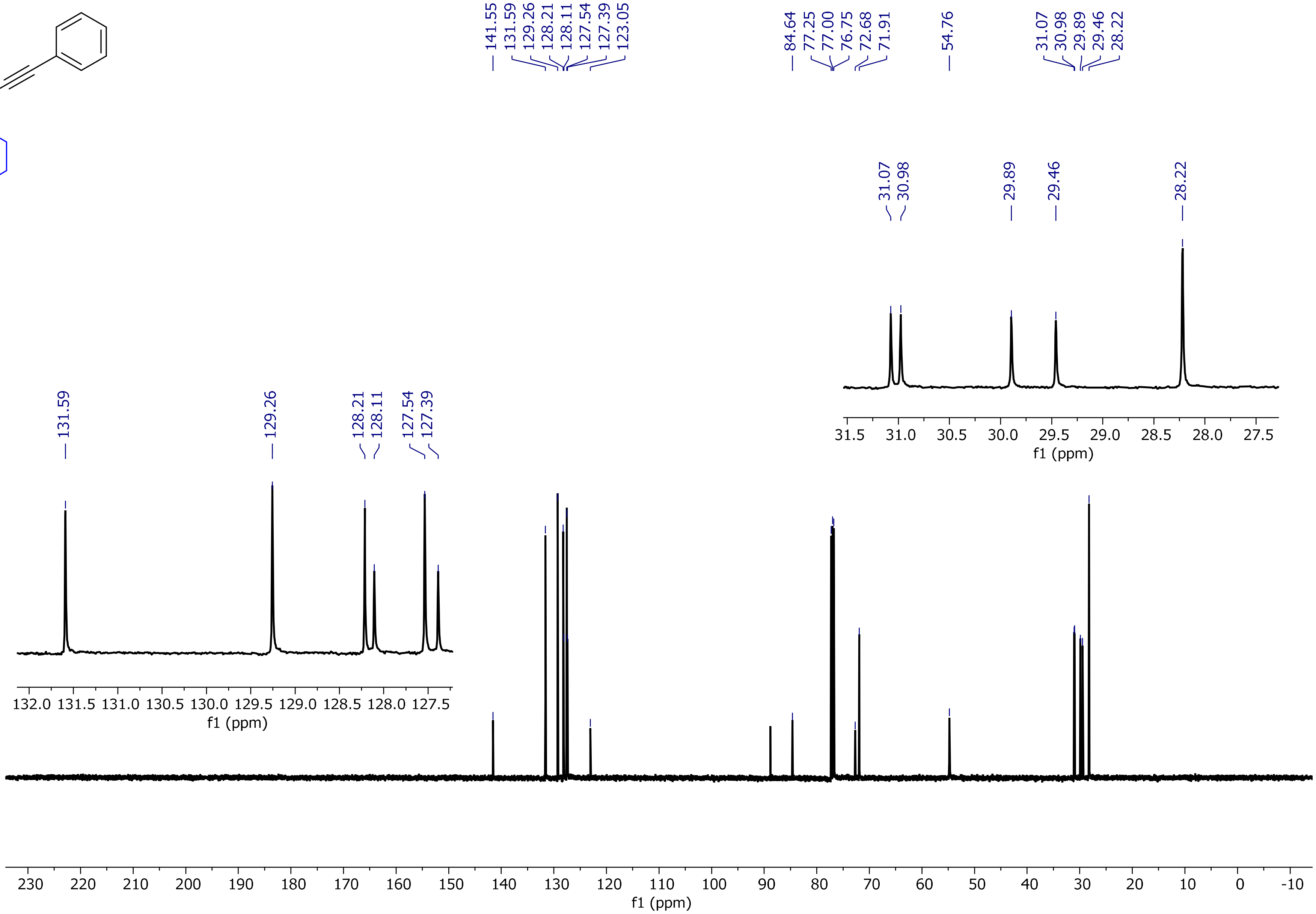

Figure S50. ^{13}C-APT NMR ($CDCl_3$, 125 MHz) spectrum **6c**

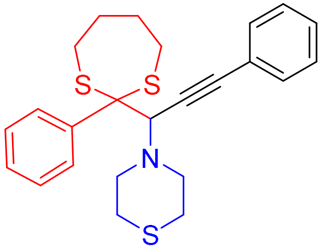

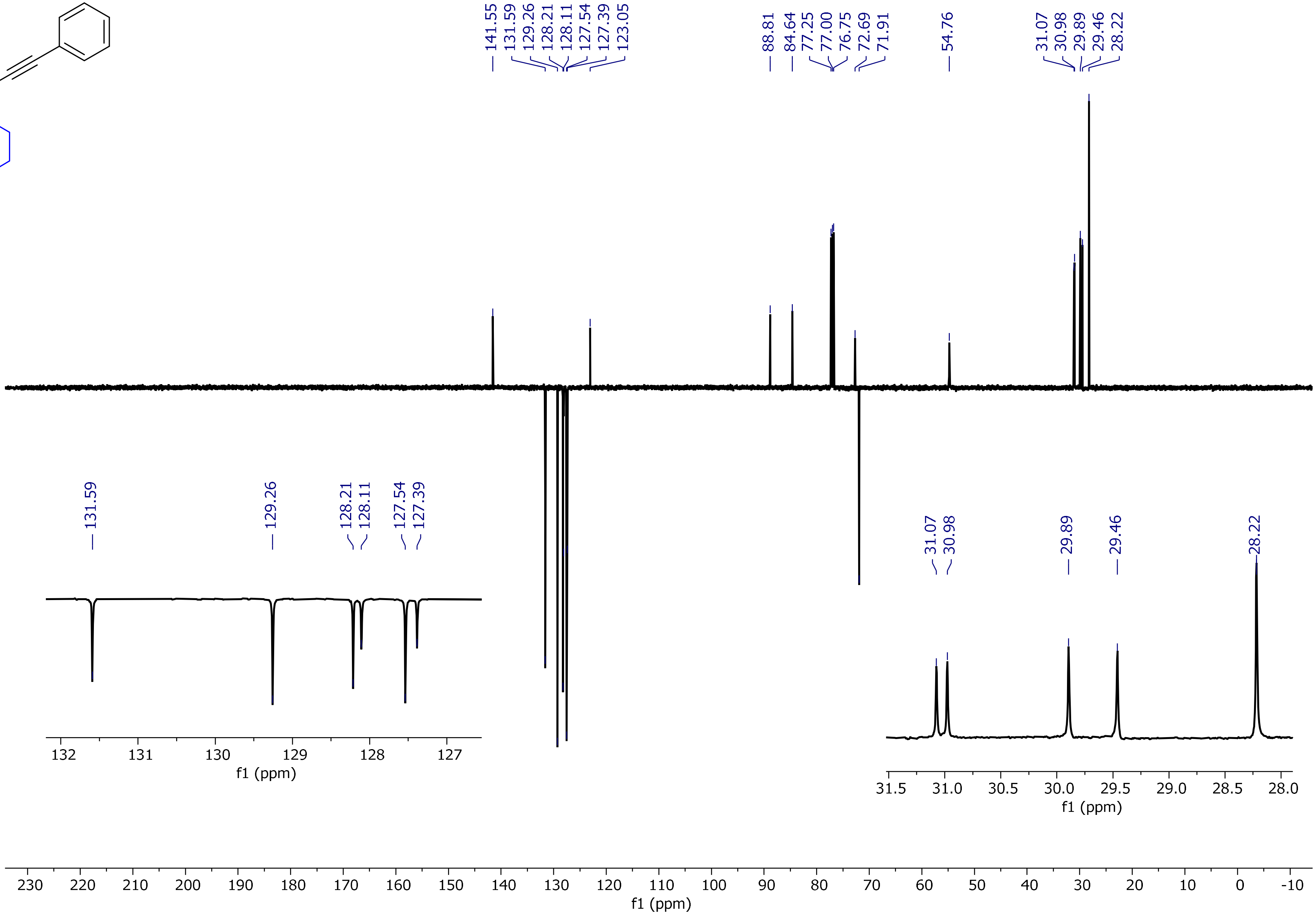

Figure S51. ^{1}H- NMR ($CDCl_3$, 500 MHz) spectrum **6d**

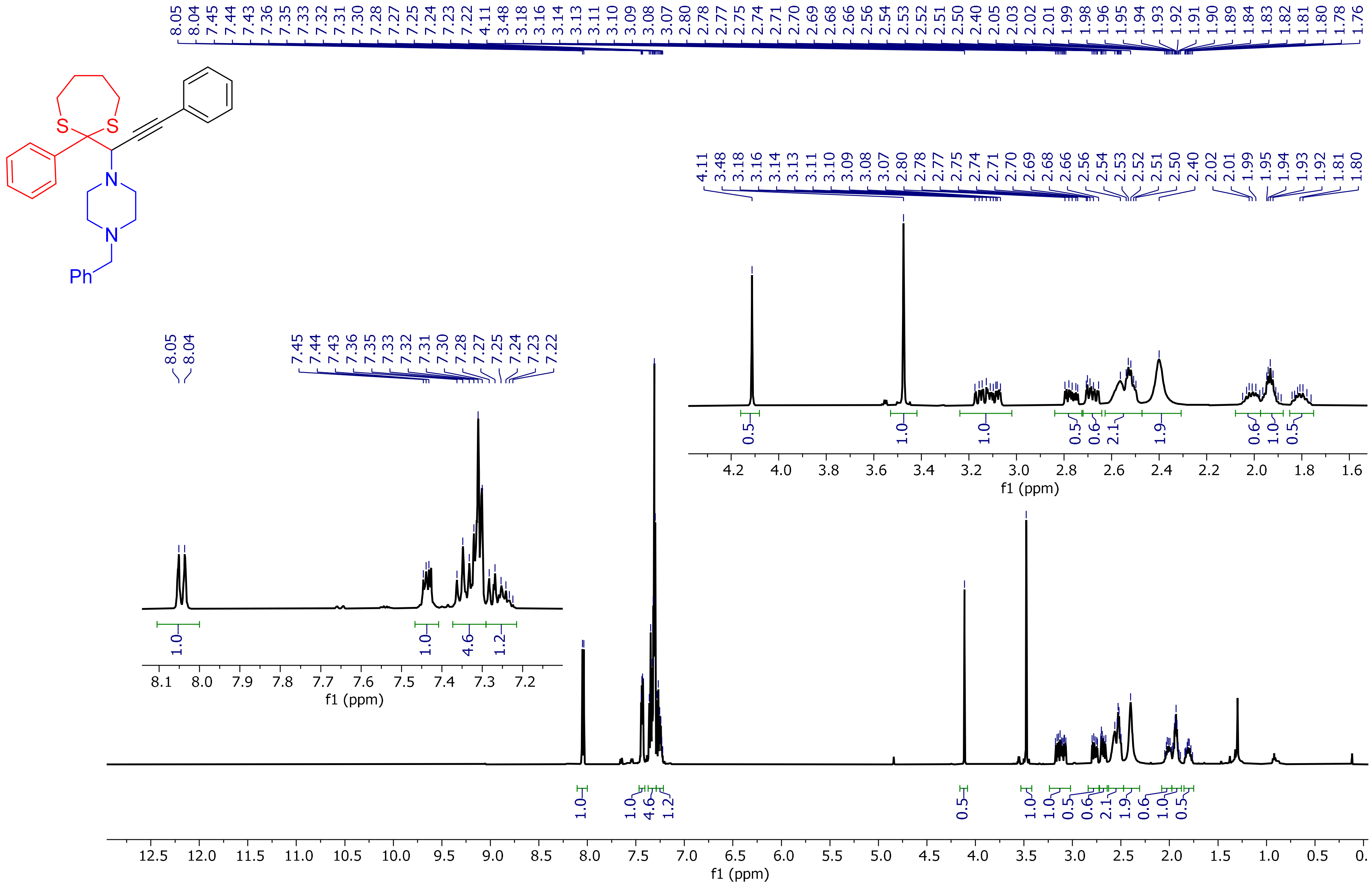

Figure S52. ^{13}C- NMR ($CDCl_3$, 125 MHz) spectrum **6d**

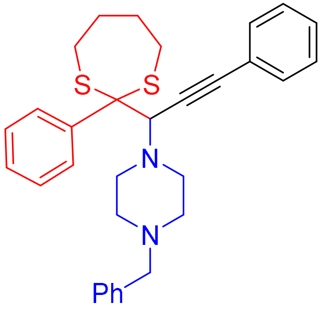

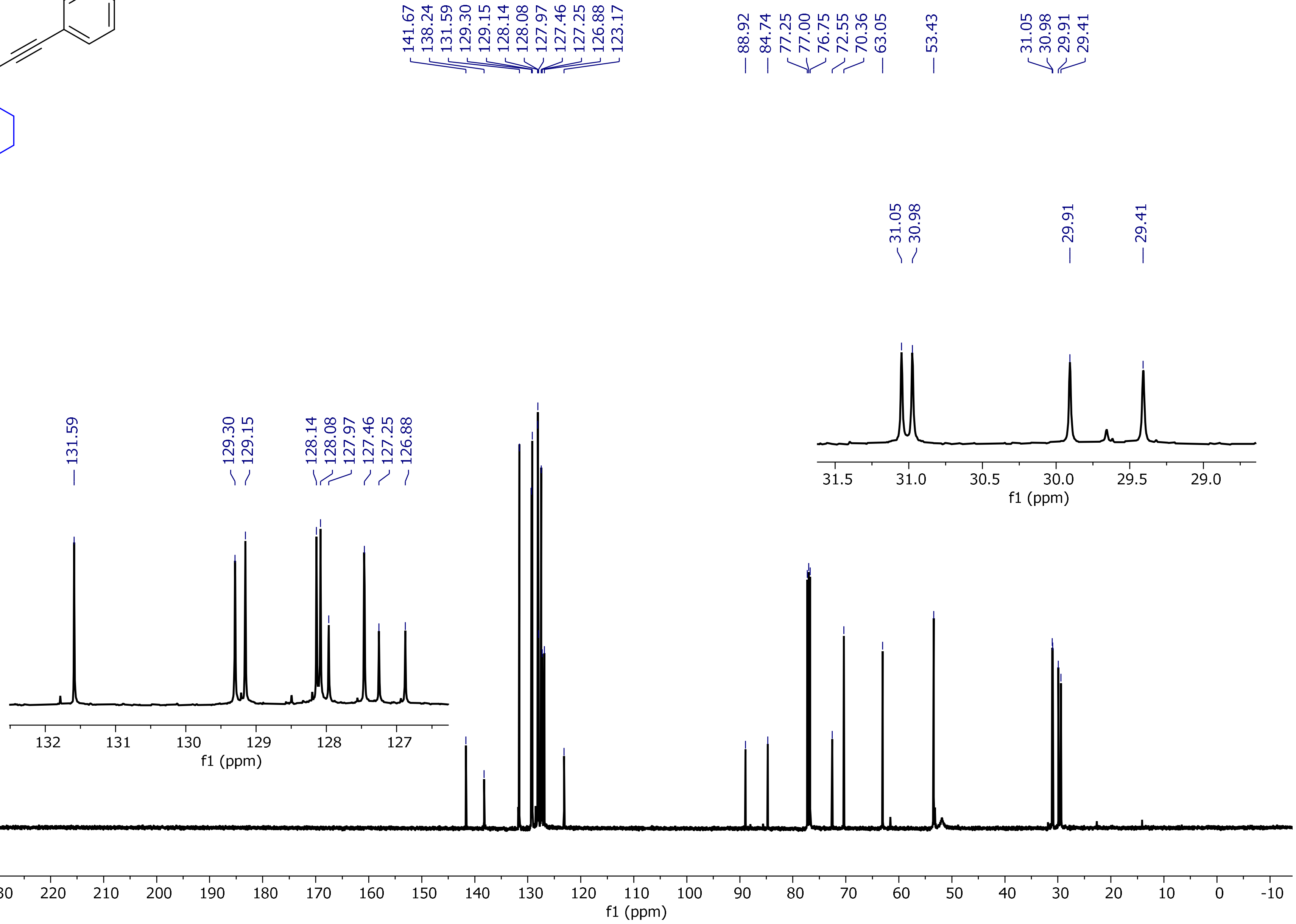

Figure S53. ^{13}C-APT NMR ($CDCl_3$, 125 MHz) spectrum **6d**

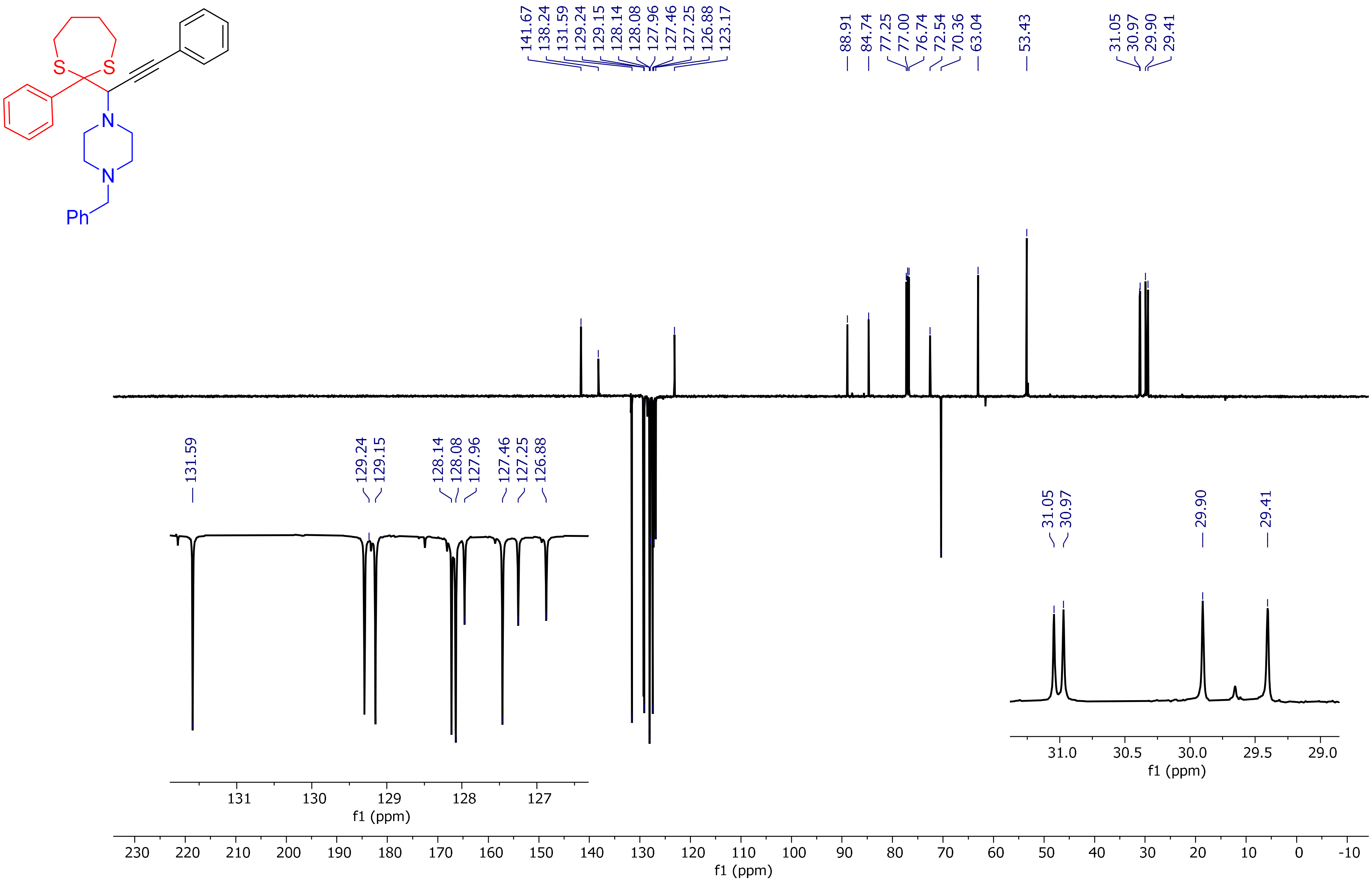

Figure S54. ^{1}H- NMR ($CDCl_3$, 500 MHz) spectrum **2a**

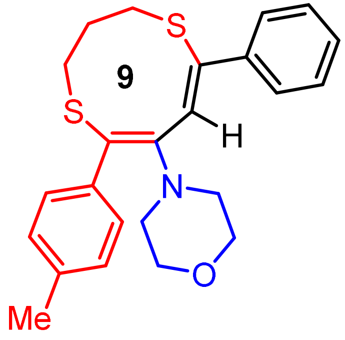

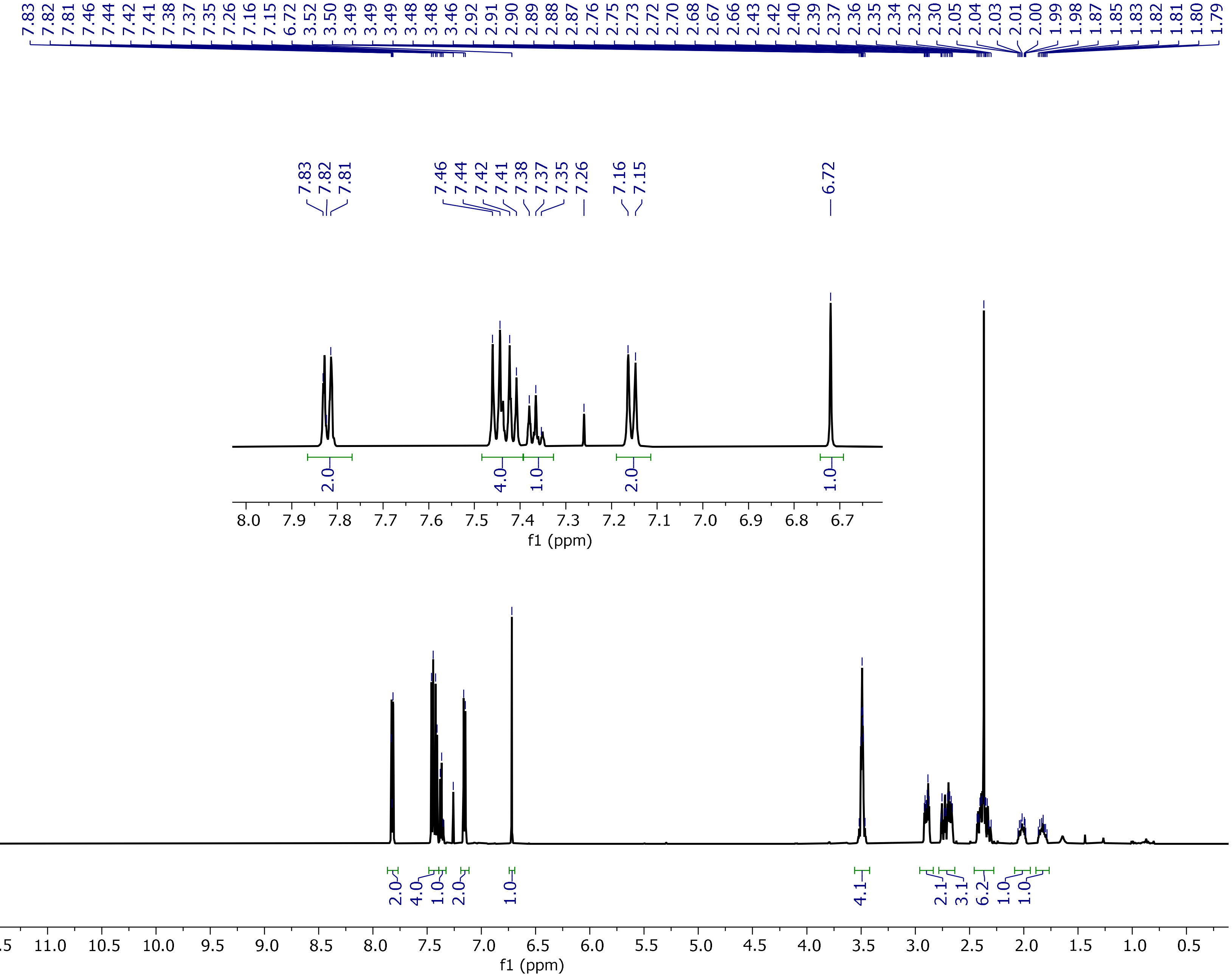

Figure S55. ^{1}H- NMR ($CDCl_3$, 500 MHz) spectrum **D-2a**

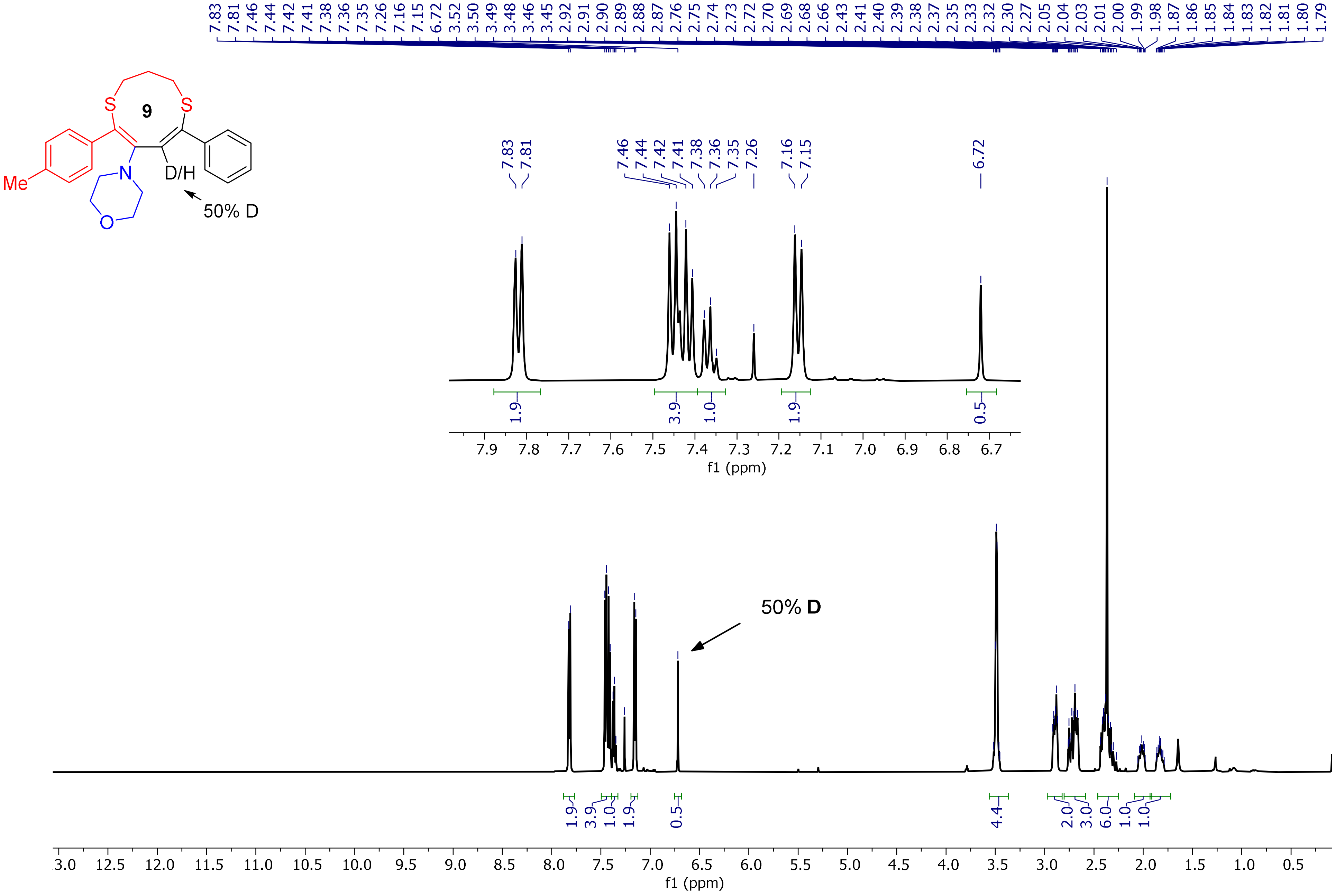

Figure S56. ^{13}C-APT NMR (CDCl$_3$, 125 MHz) spectrum **2a**

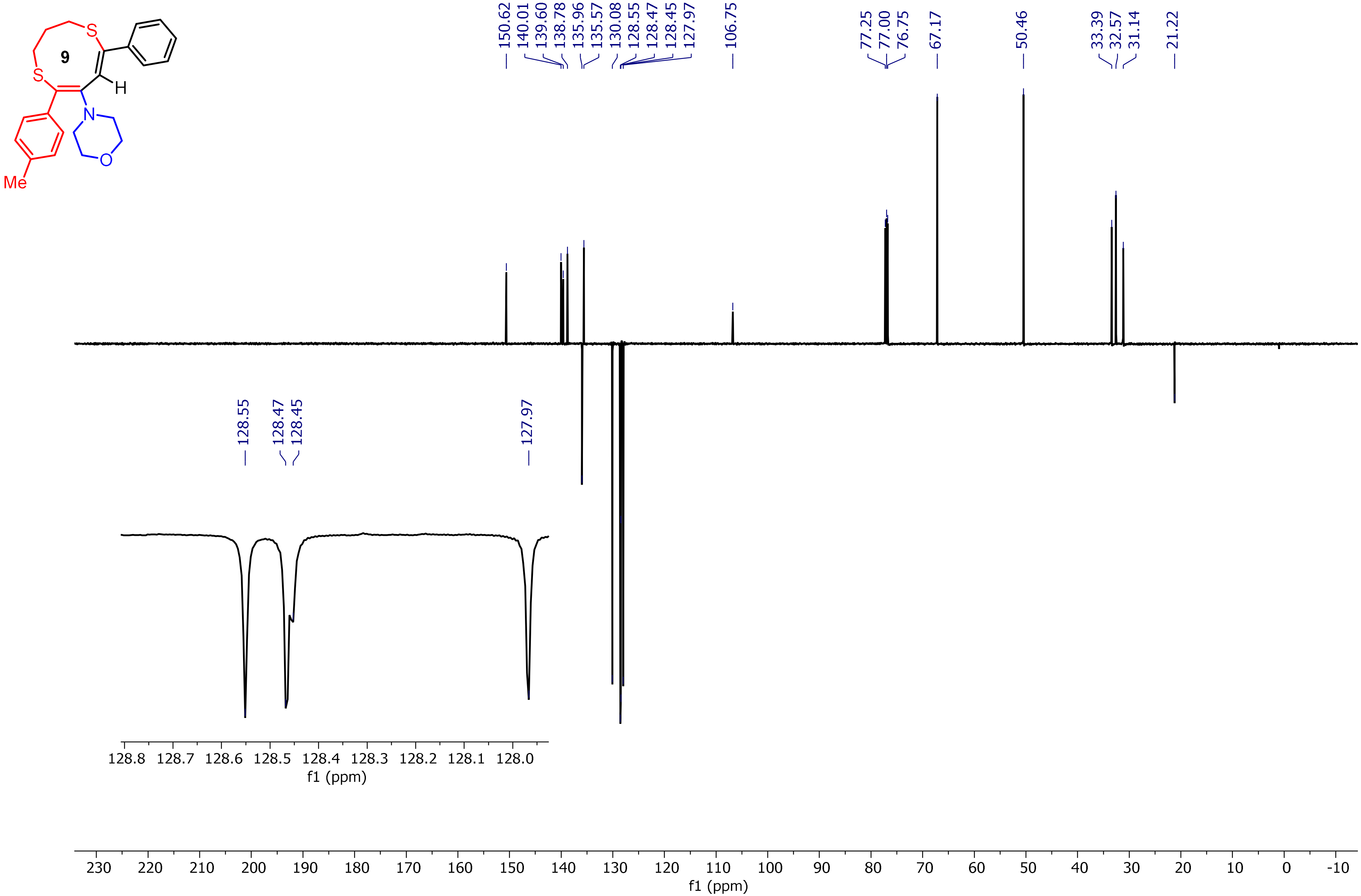

Figure S57. ^{13}C-APT NMR ($CDCl_3$, 125 MHz) spectrum **D-2a**

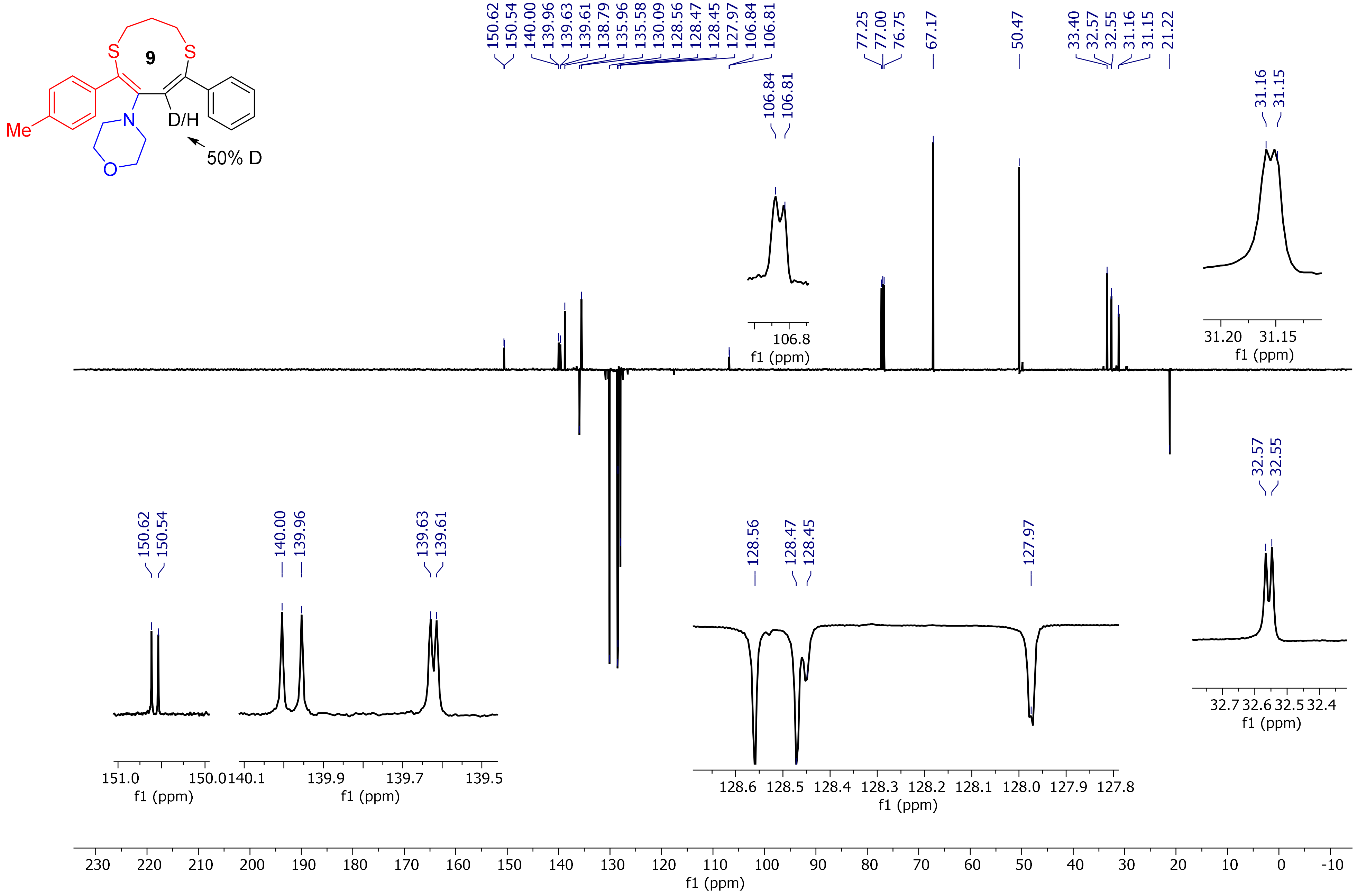

Figure S58. ^{1}H- NMR ($CDCl_3$, 500 MHz) spectrum **2b**

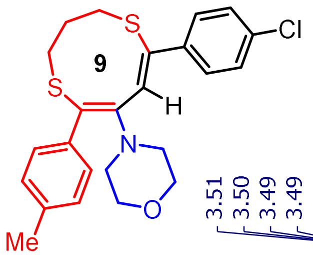

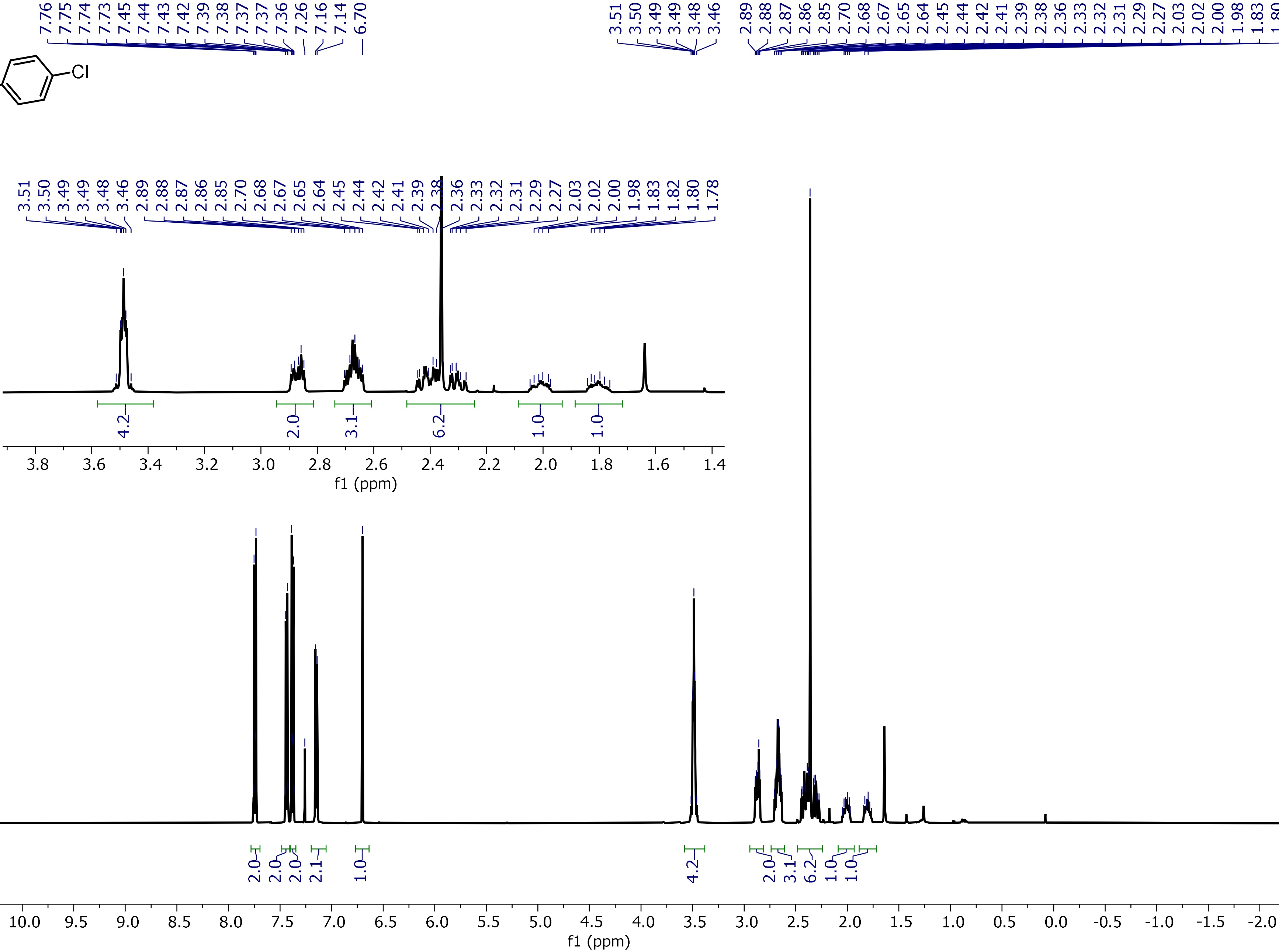

Figure S59. ^{13}C-APT NMR ($CDCl_3$, 125 MHz) spectrum **2b**

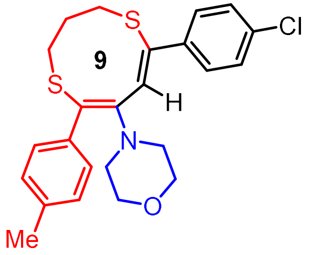

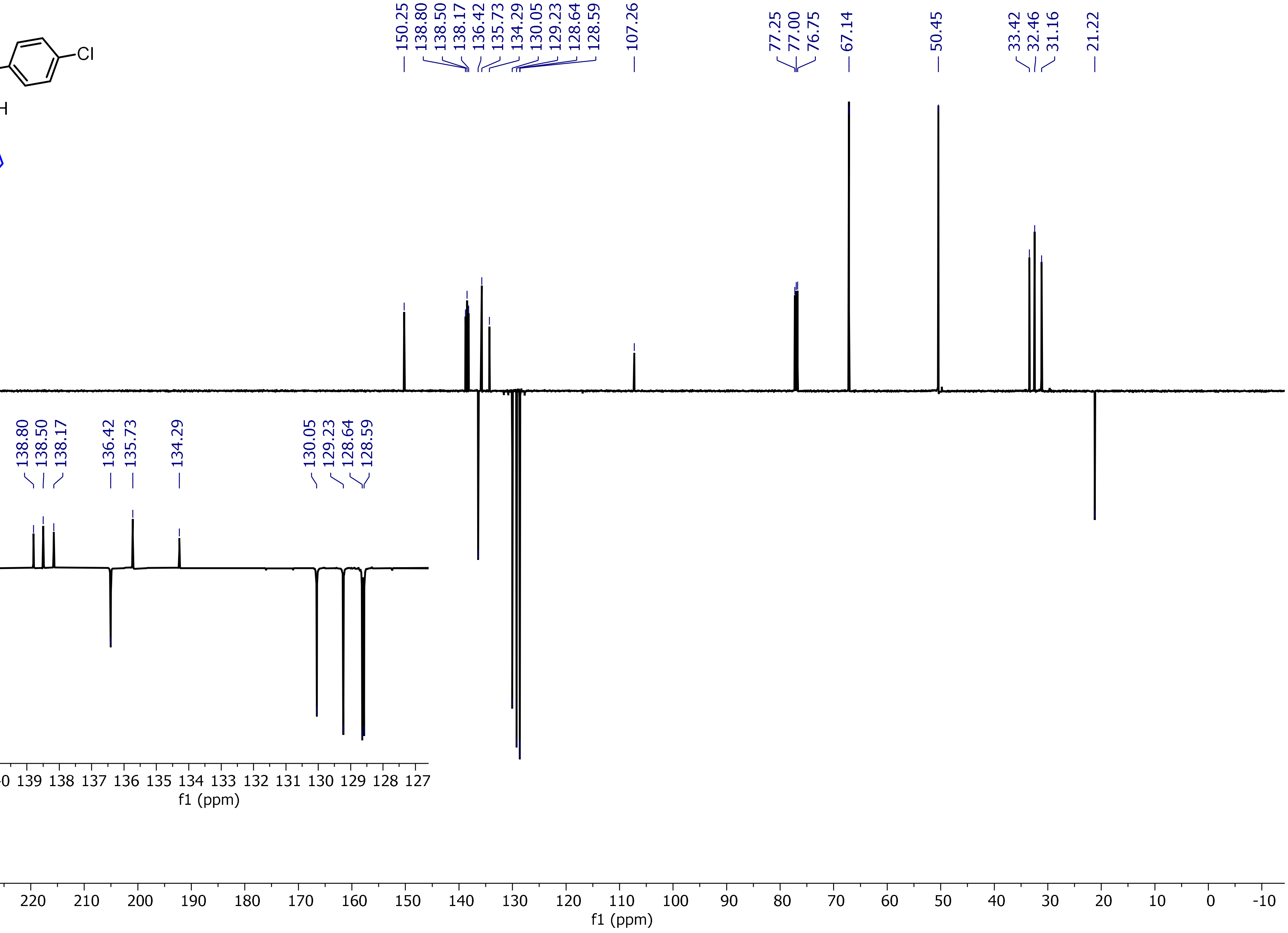

Figure S60. ^{1}H-NMR ($CDCl_3$, 500 MHz) spectrum **2c**

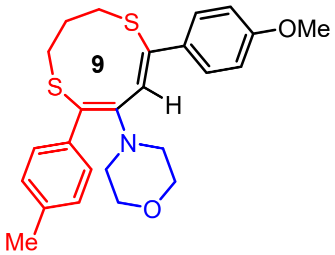

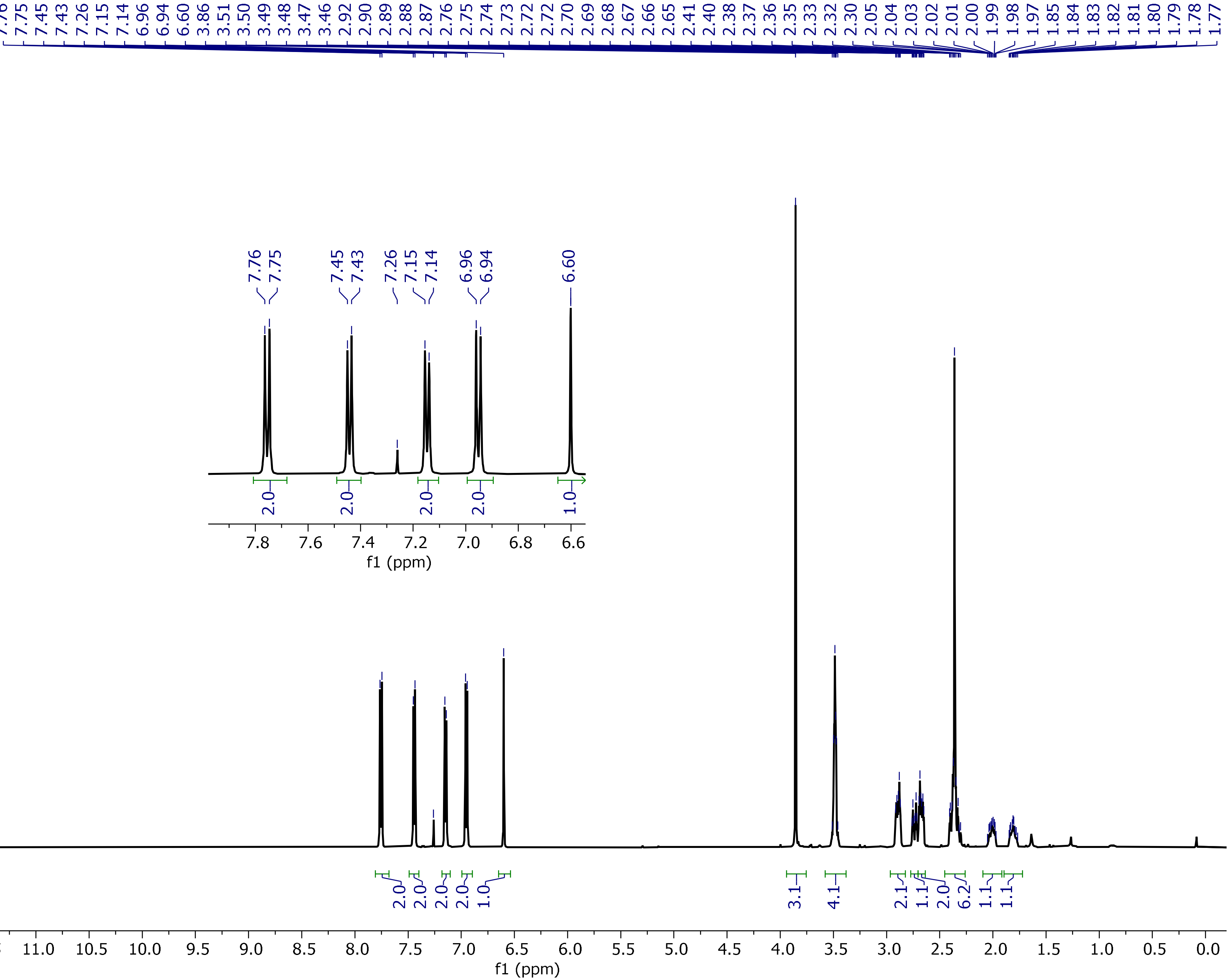

Figure S61. ^{13}C-APT NMR ($CDCl_3$, 125 MHz) spectrum **2c**

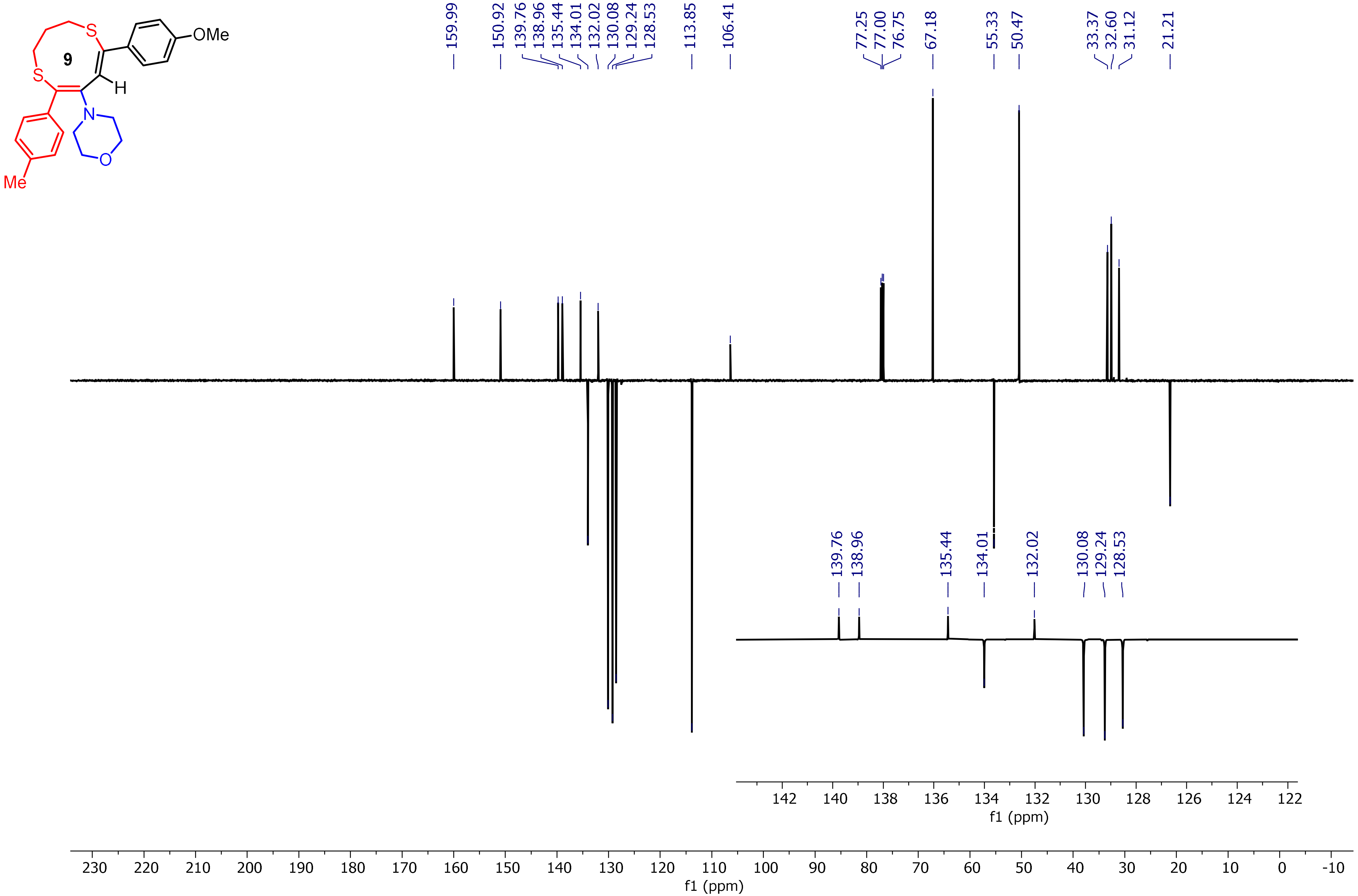

Figure S62. 1H-NMR ($CDCl_3$, 500 MHz) spectrum **2d**

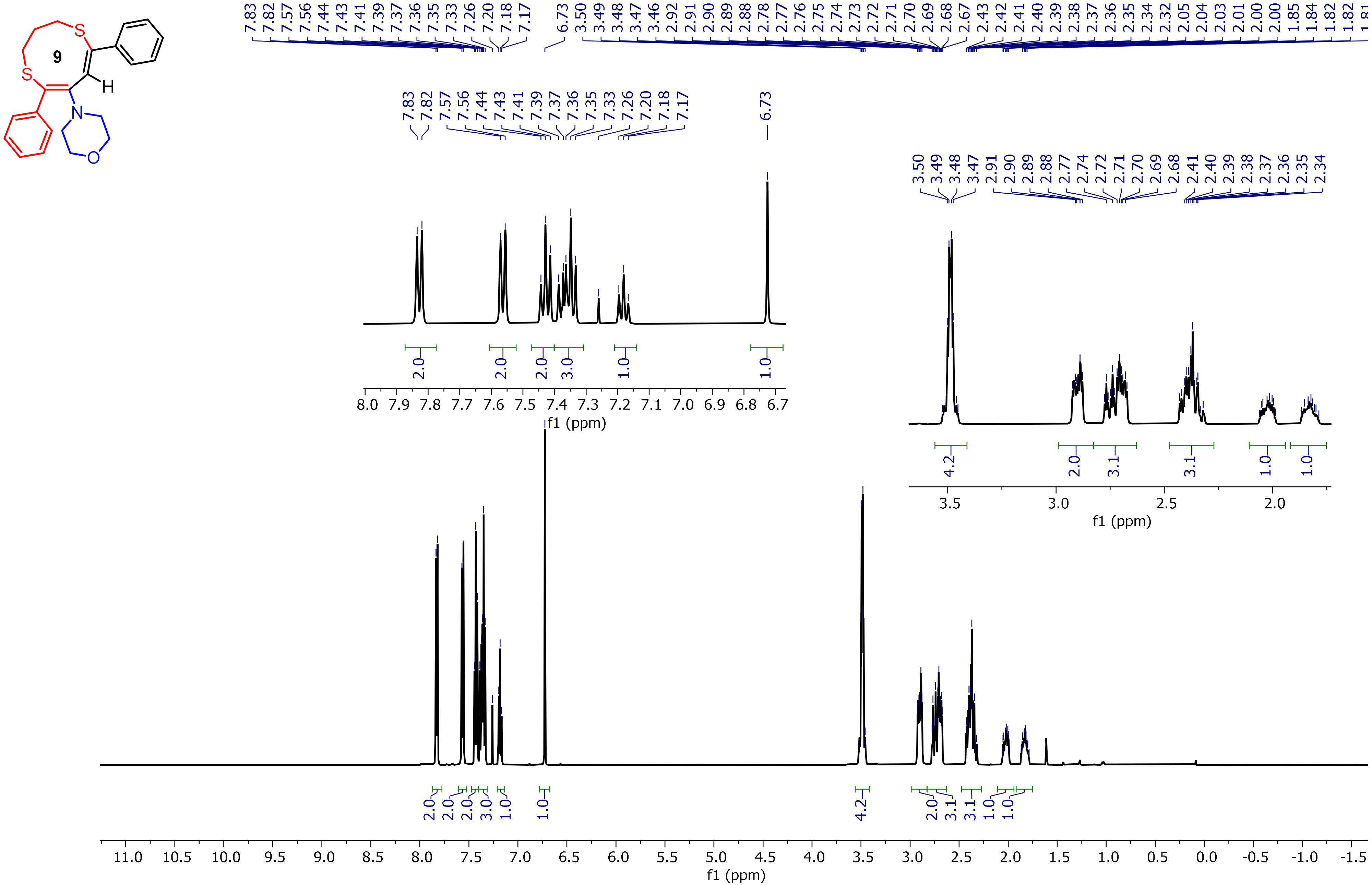

Figure S63. ^{13}C-APT NMR ($CDCl_3$, 125 MHz) spectrum **2d**

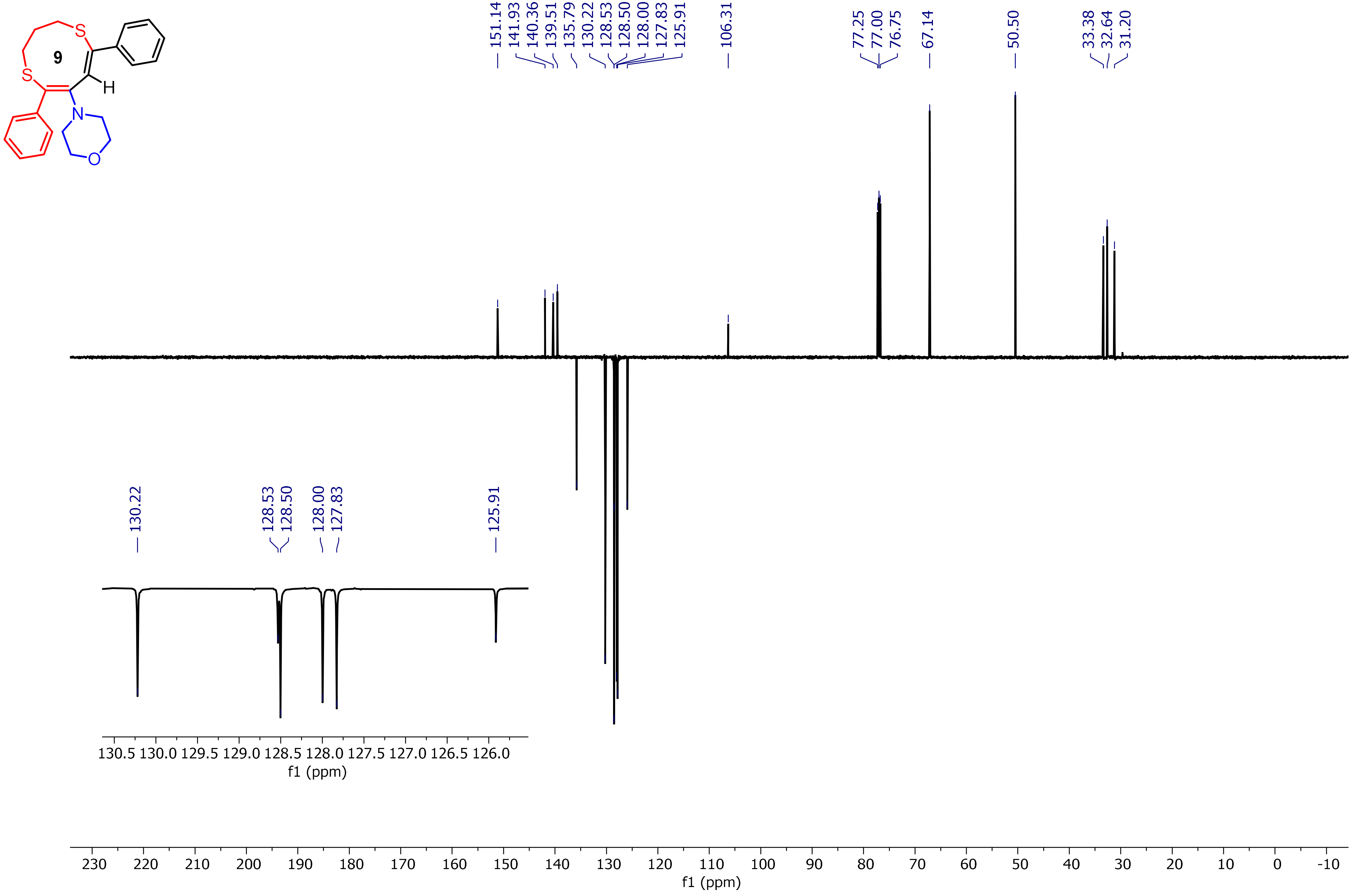

Figure S64. ^{1}H- NMR ($CDCl_3$, 500 MHz) spectrum **2e**

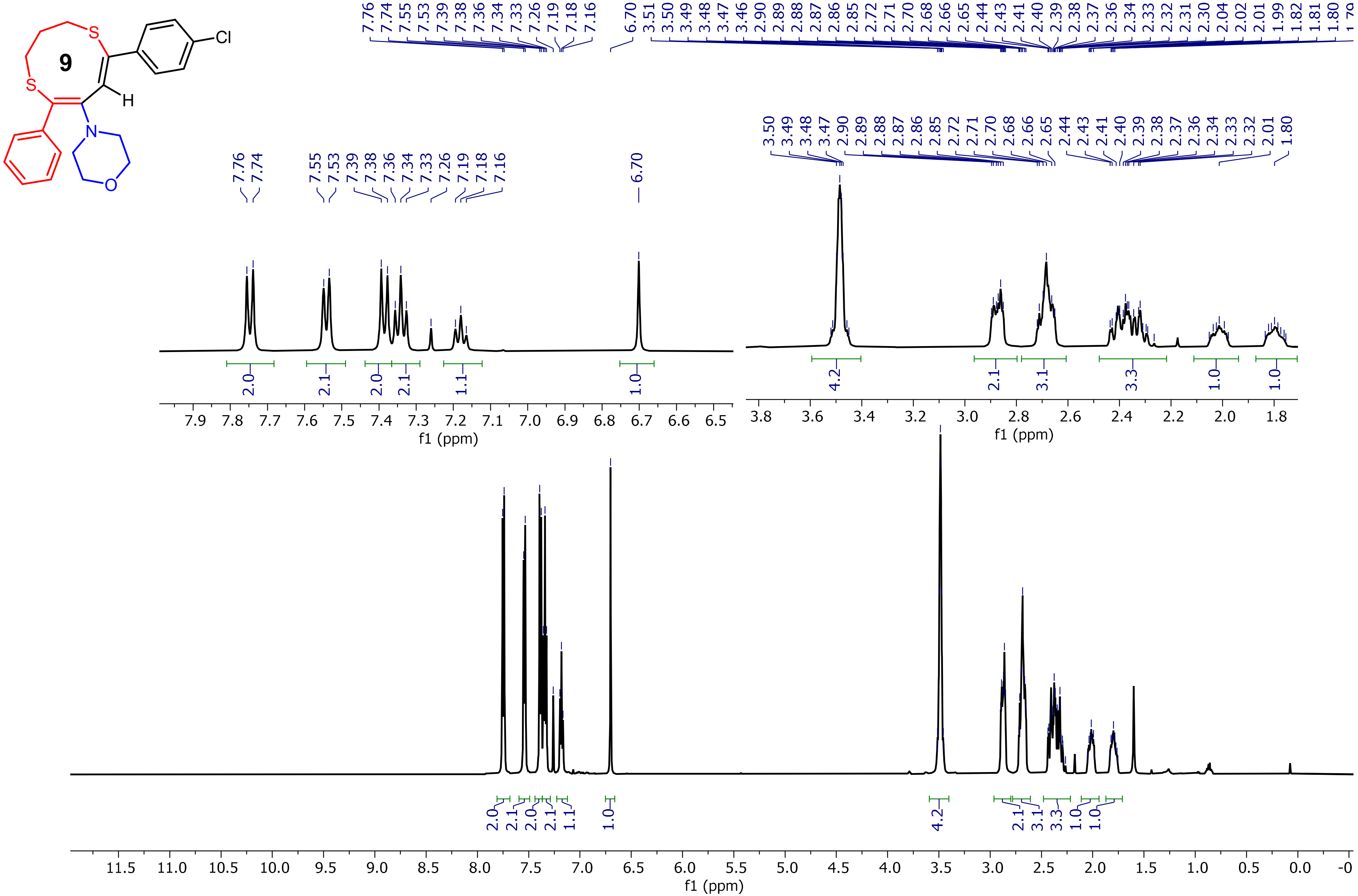

Figure S65. ^{13}C-APT NMR ($CDCl_3$, 125 MHz) spectrum **2e**

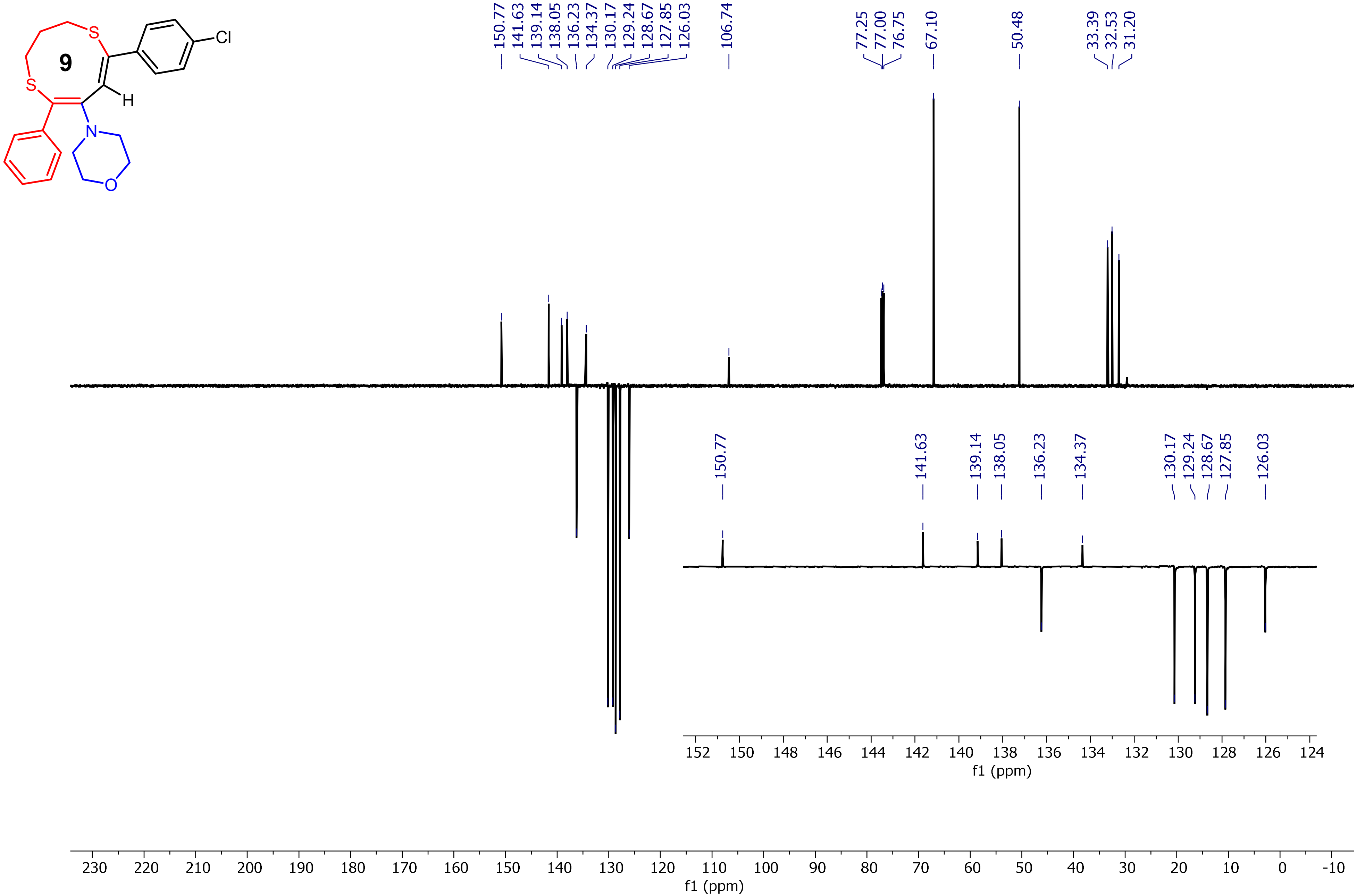

Figure S66. ^{1}H- NMR ($CDCl_3$, 500 MHz) spectrum **2f**

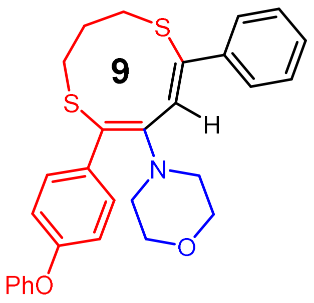

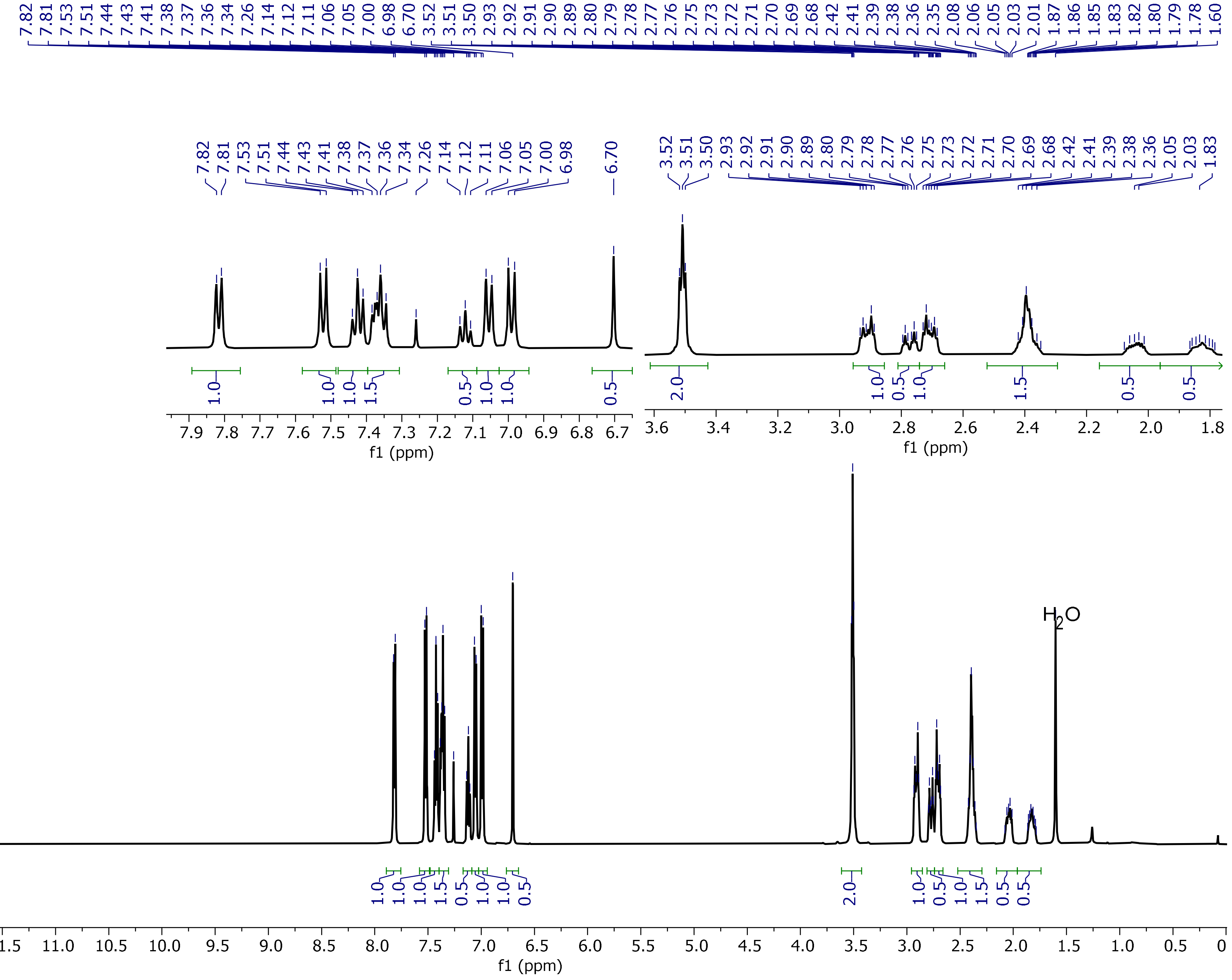

Figure S67. ^{13}C-APT NMR ($CDCl_3$, 125 MHz) spectrum **2f**

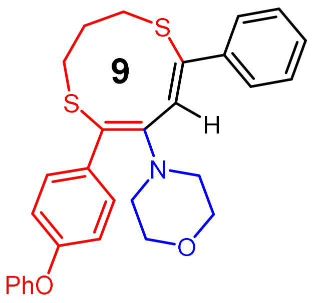

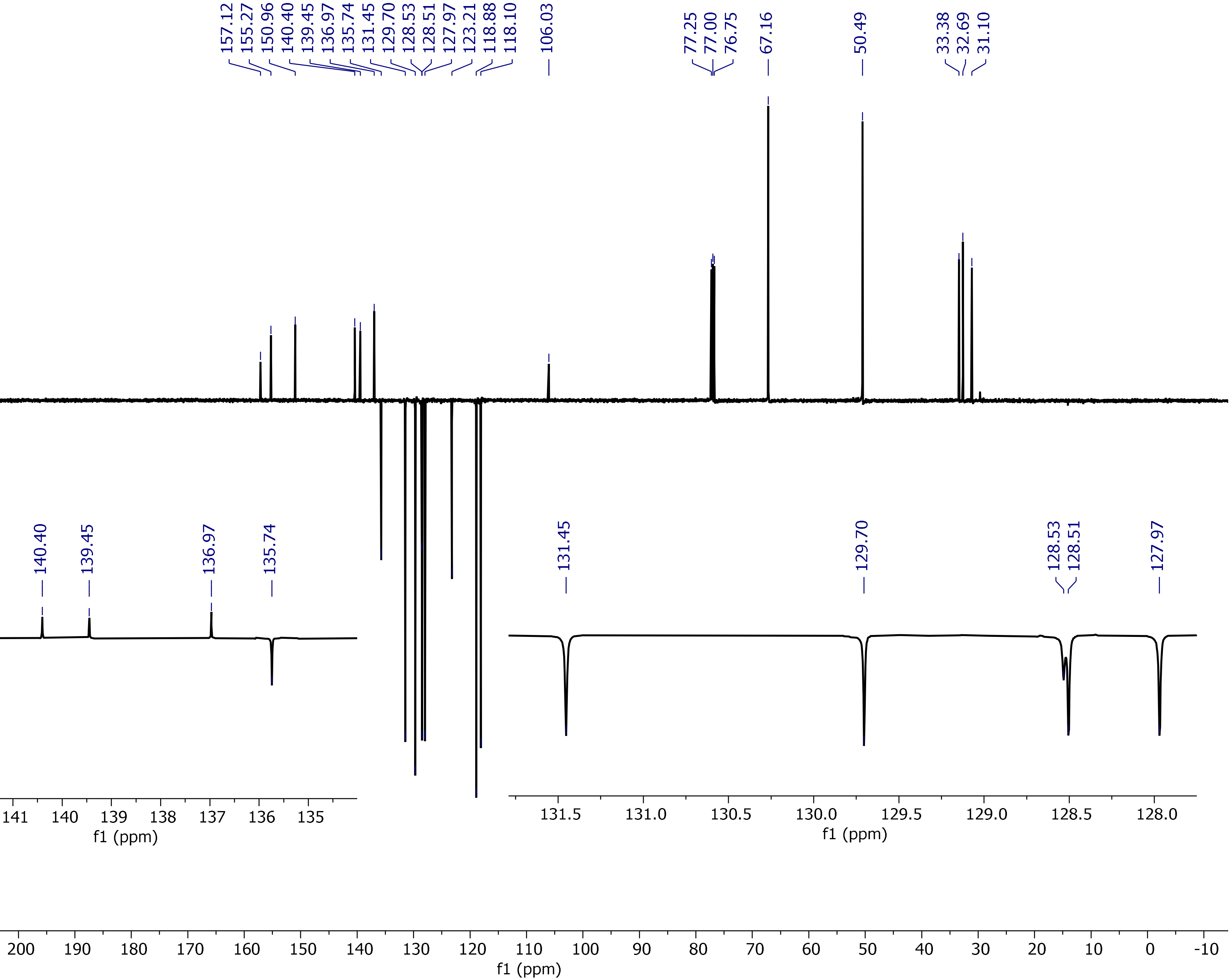

Figure S68. ^{1}H- NMR ($CDCl_3$, 500 MHz) spectrum **2g**

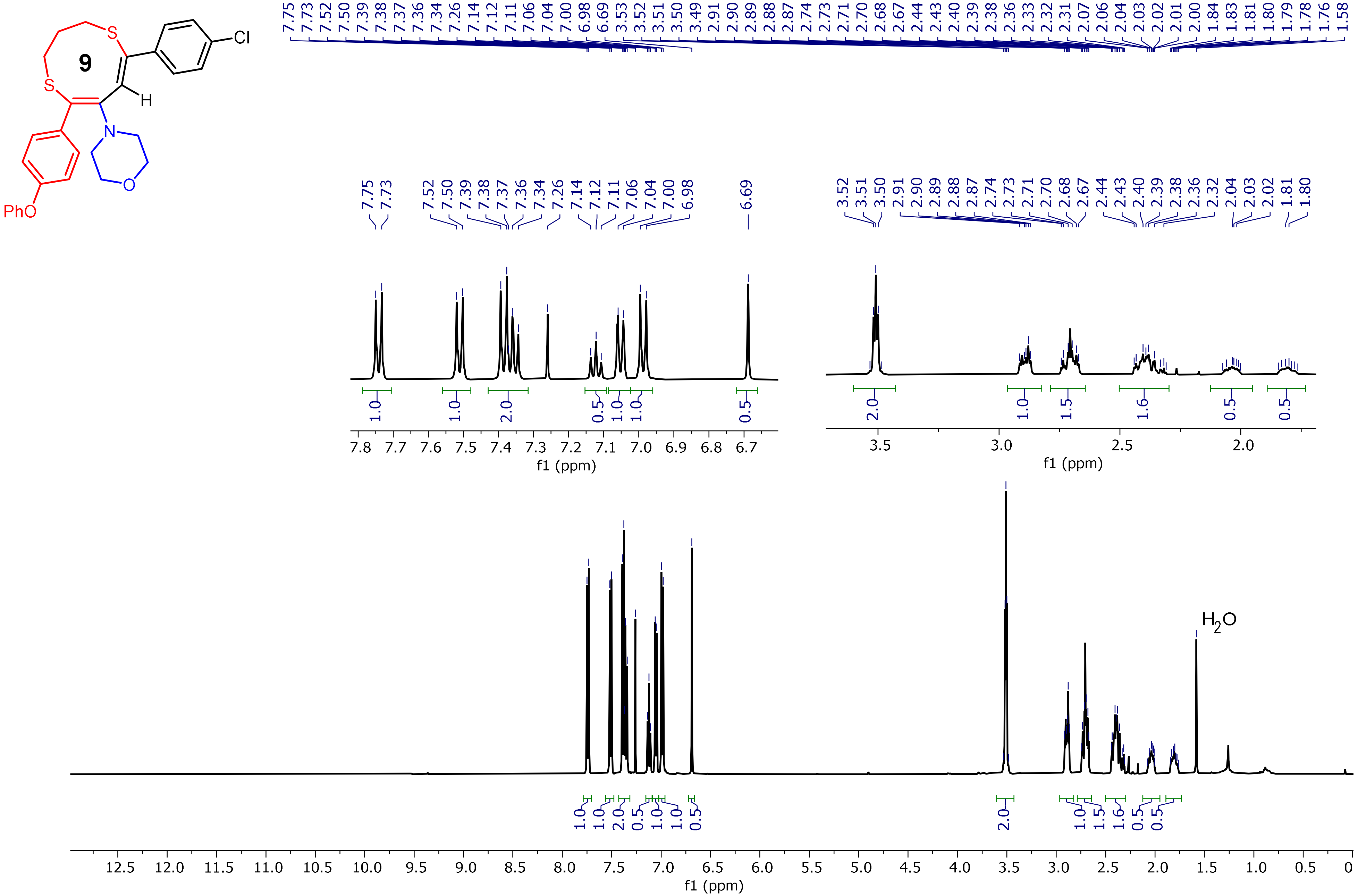

Figure S69. ^{13}C-APT NMR ($CDCl_3$, 125 MHz) spectrum **2g**

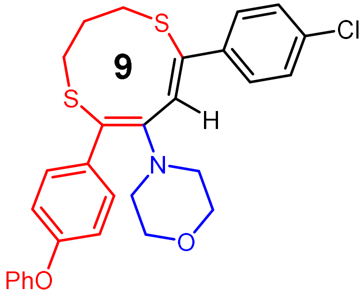

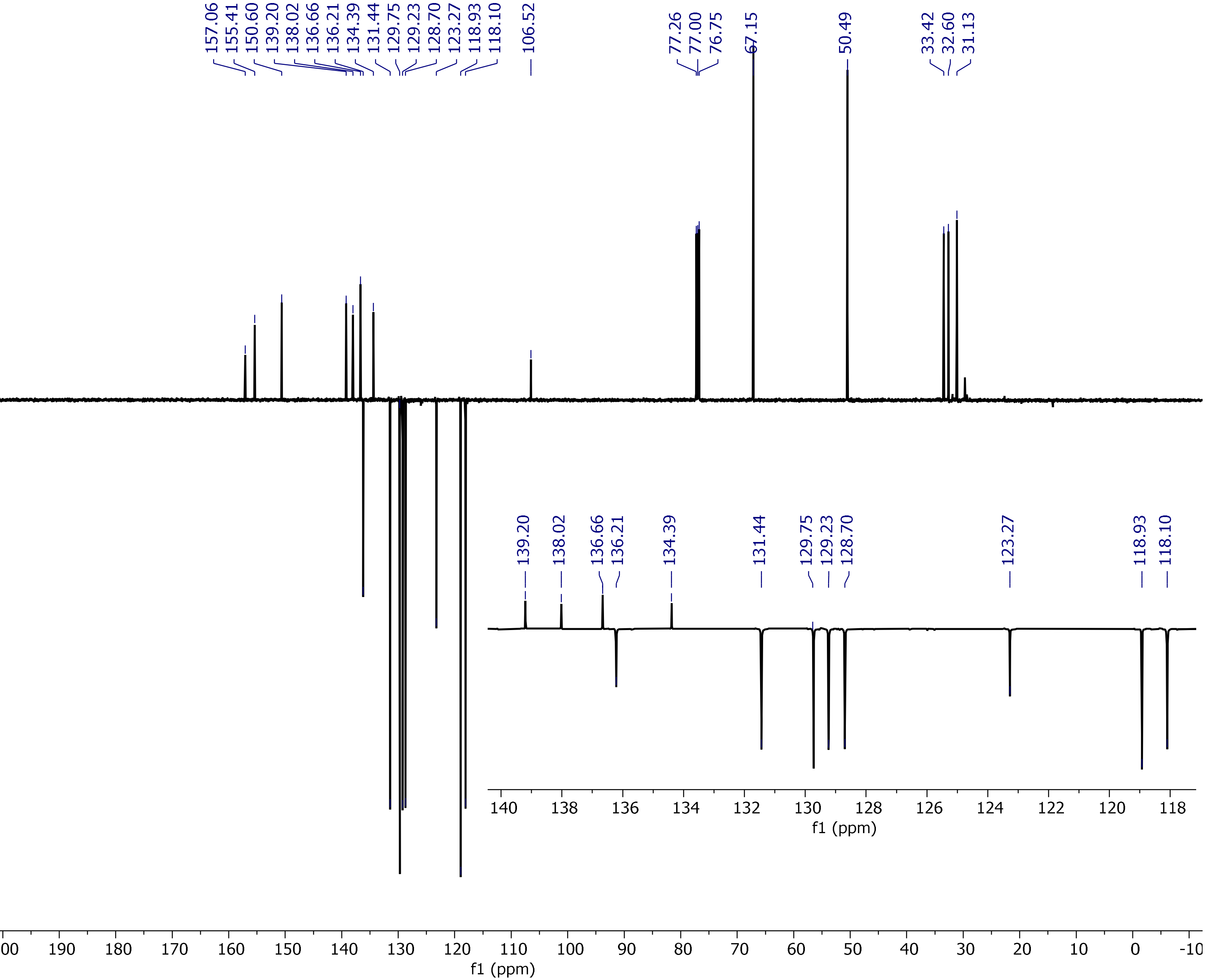

Figure S70. ^{1}H- NMR ($CDCl_3$, 500 MHz) spectrum **2h**

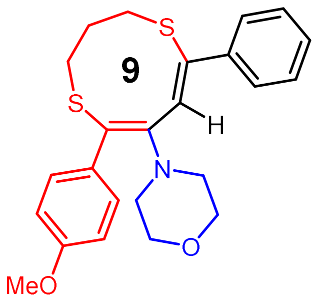

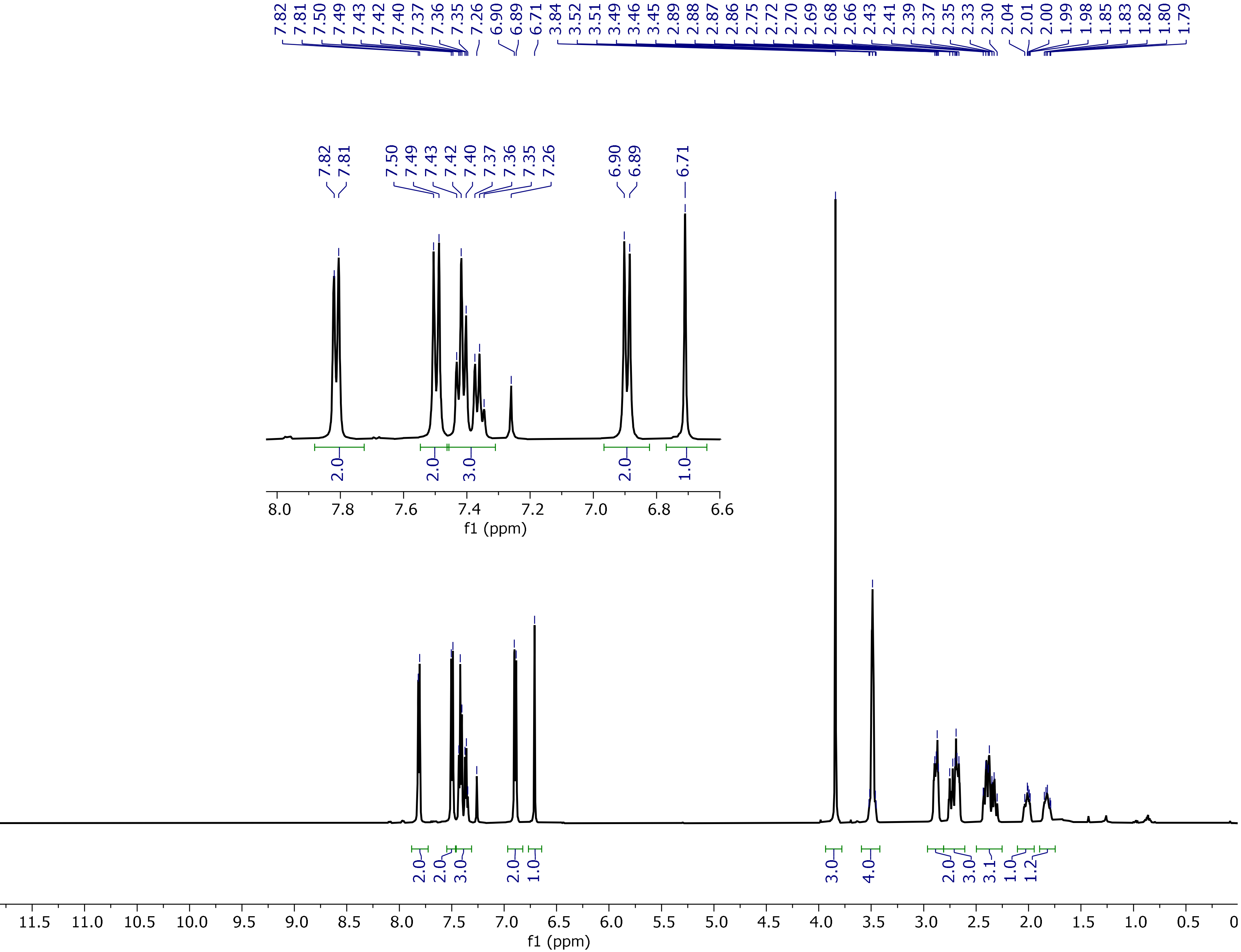

Figure S71. ^{13}C-APT NMR ($CDCl_3$, 125 MHz) spectrum **2h**

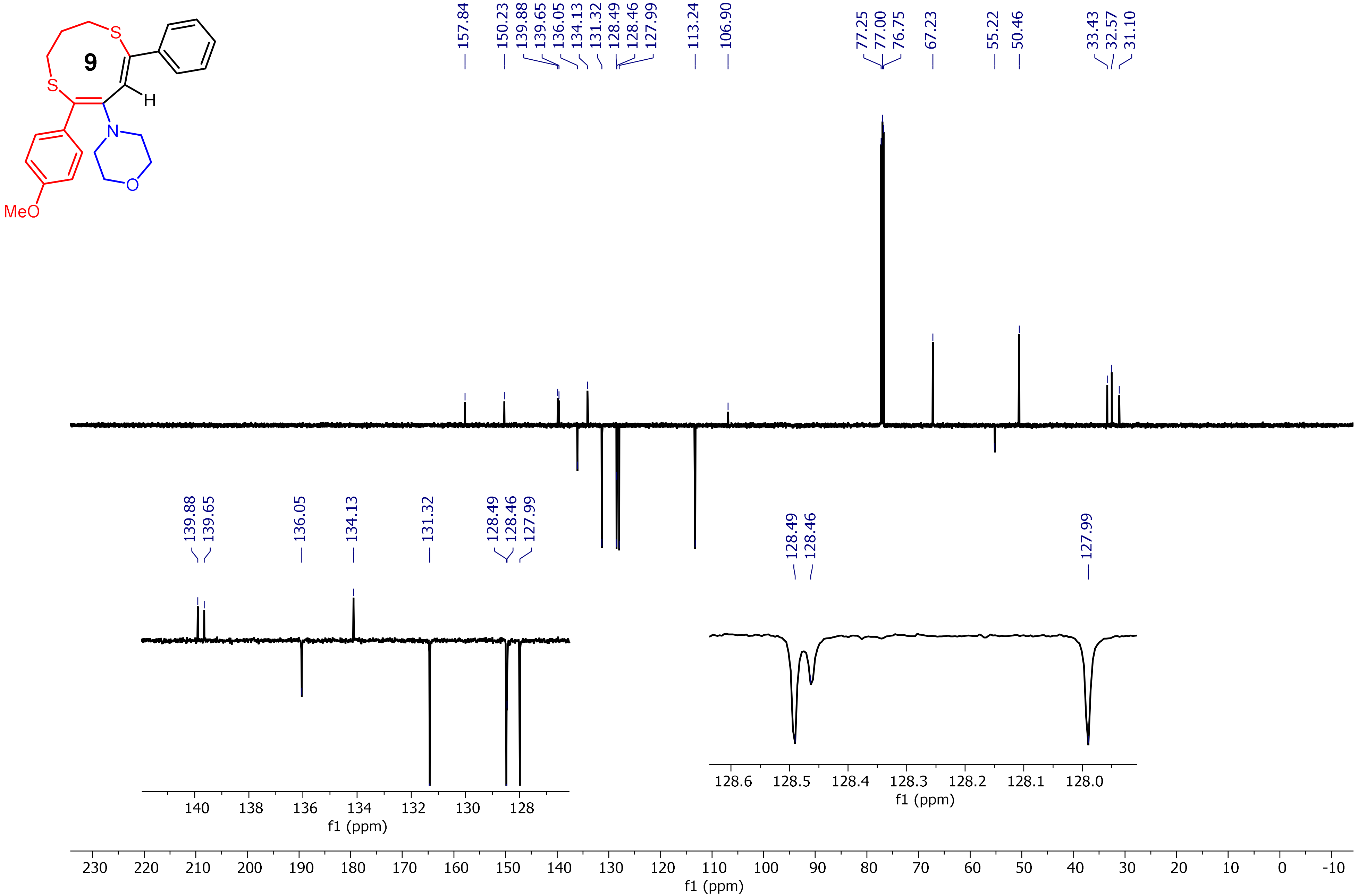

Figure S72. ^{1}H- NMR ($CDCl_3$, 500 MHz) spectrum **2i**

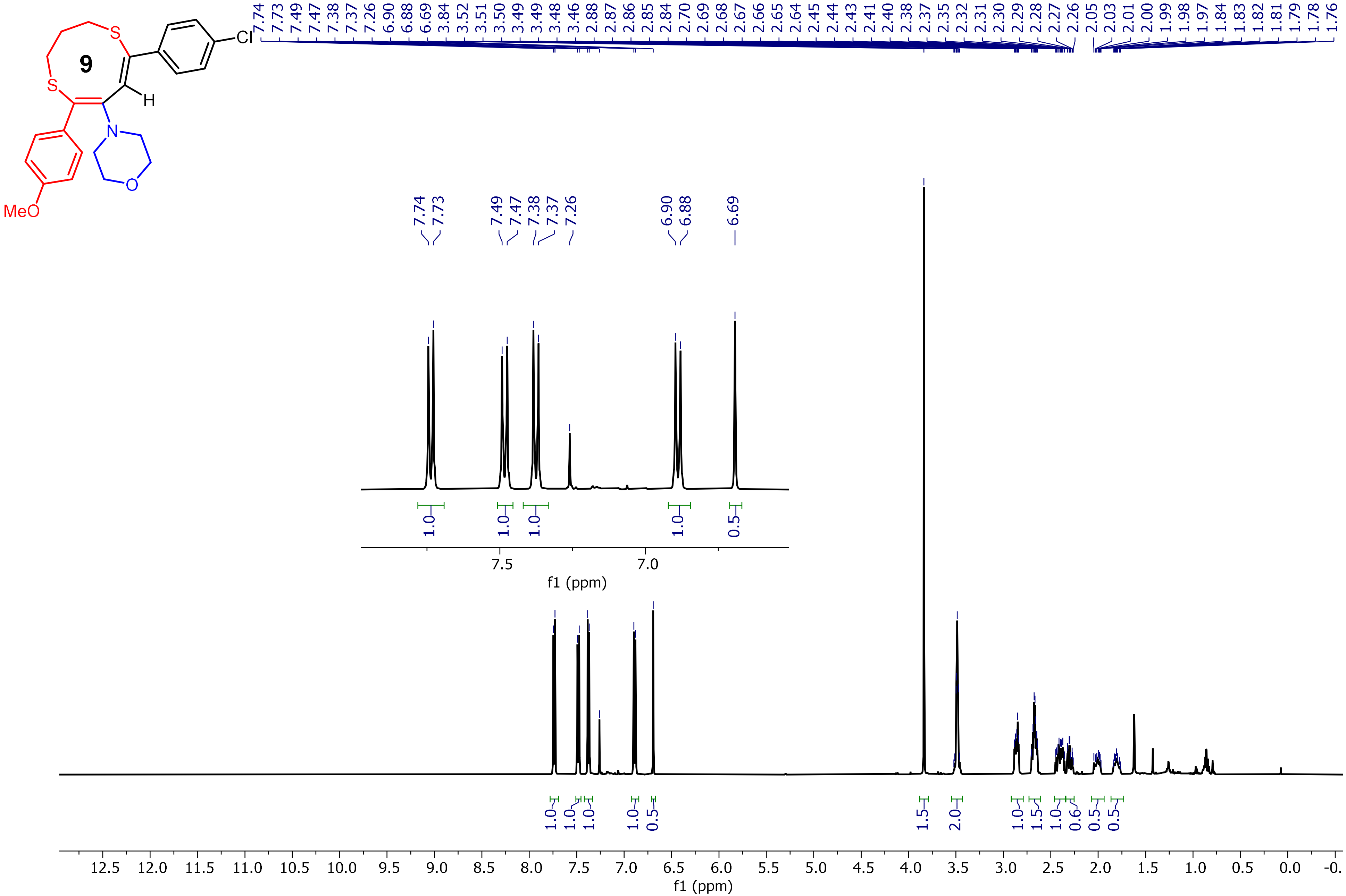

Figure S73. ^{13}C-APT NMR ($CDCl_3$, 125 MHz) spectrum **2i**

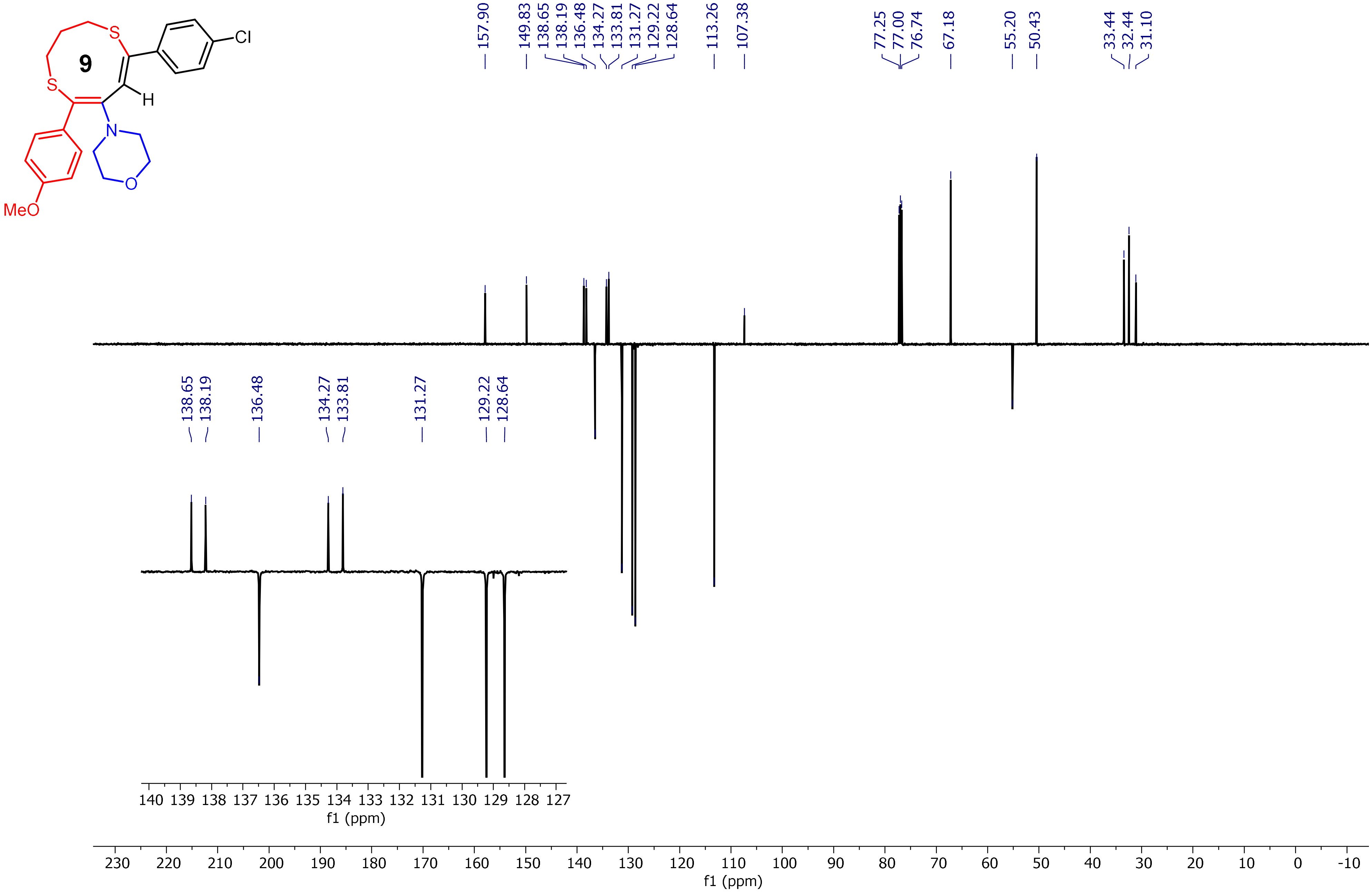

Figure S74. ^{1}H-NMR ($CDCl_3$, 500 MHz) spectrum **2j**

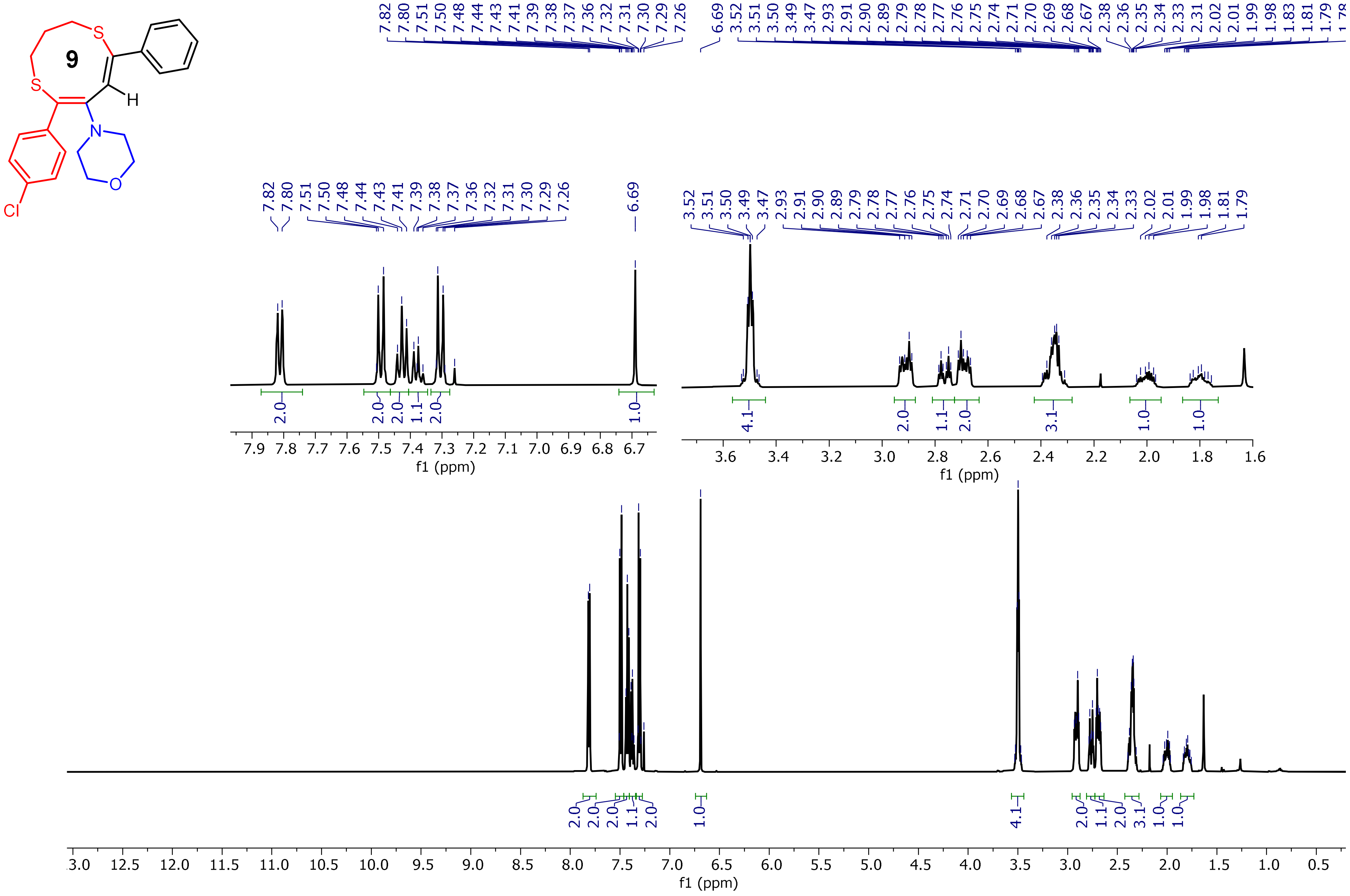

Figure S75. ^{13}C-APT ($CDCl_3$, 500 MHz) spectrum **2j**

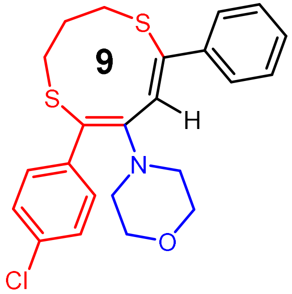

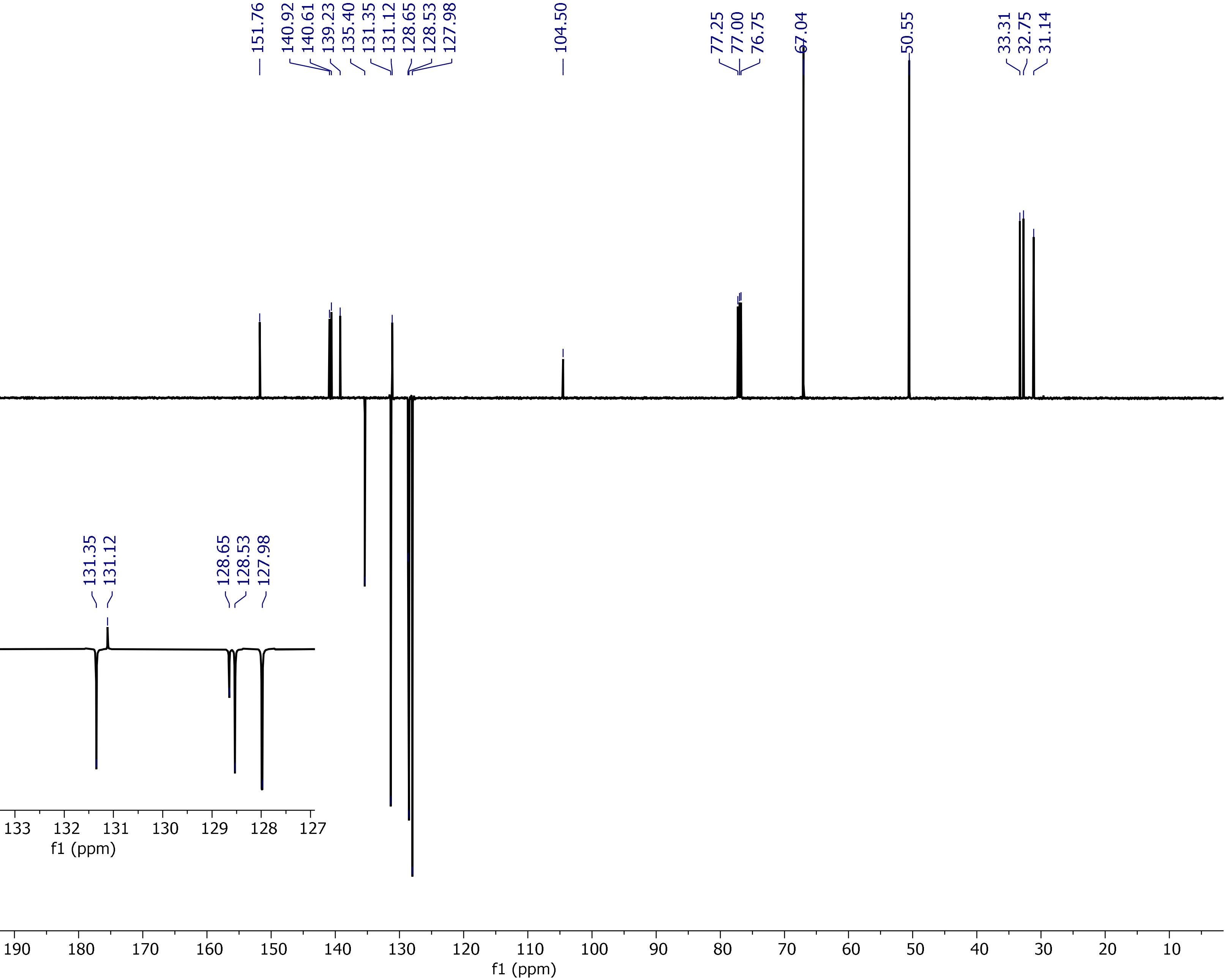

Figure S76. ^{1}H- NMR ($CDCl_3$, 500 MHz) spectrum **2k**

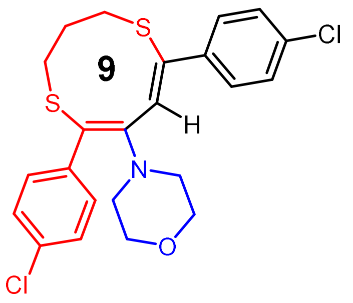

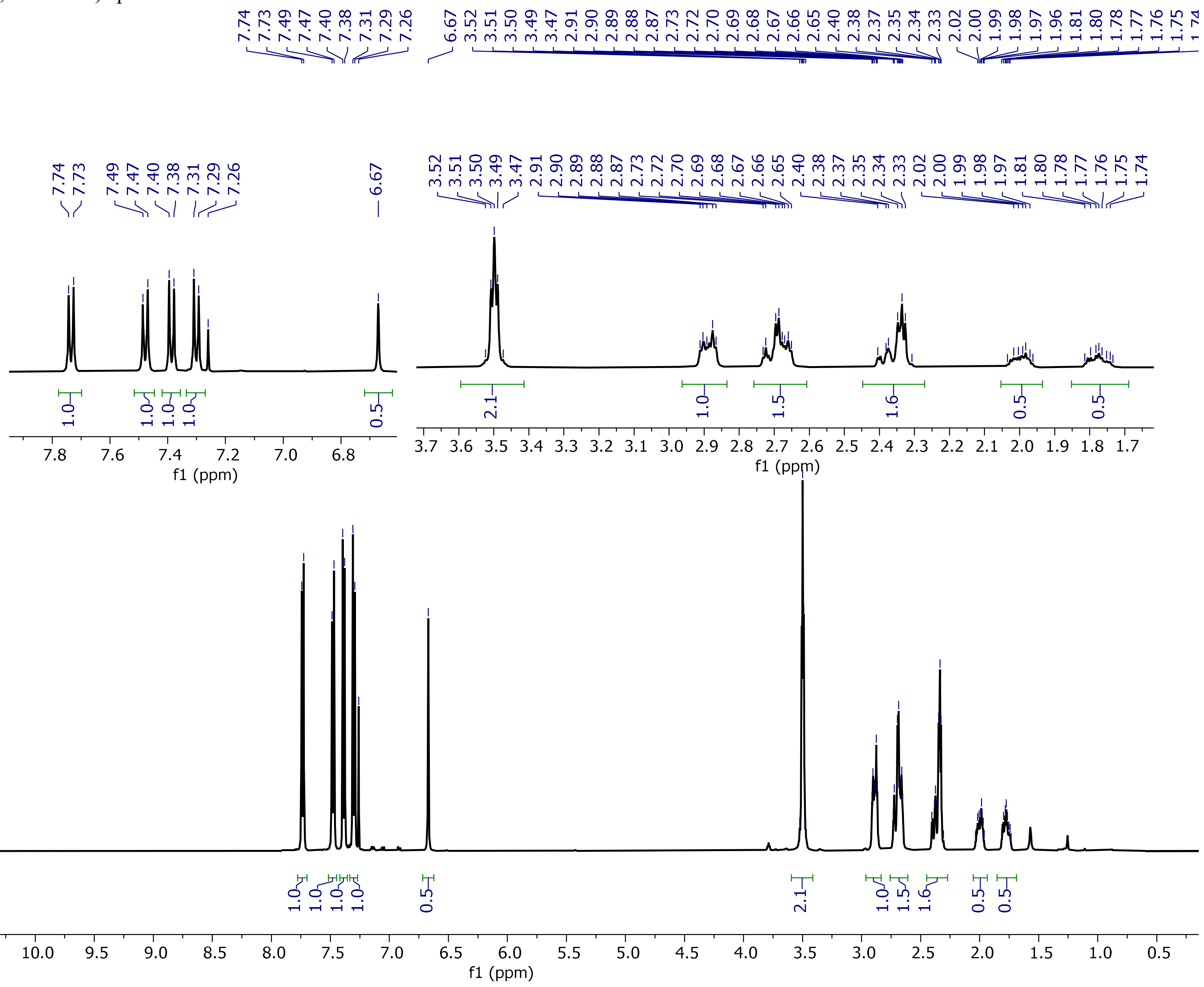

Figure S77. ^{13}C-APT ($CDCl_3$, 500 MHz) spectrum **2k**

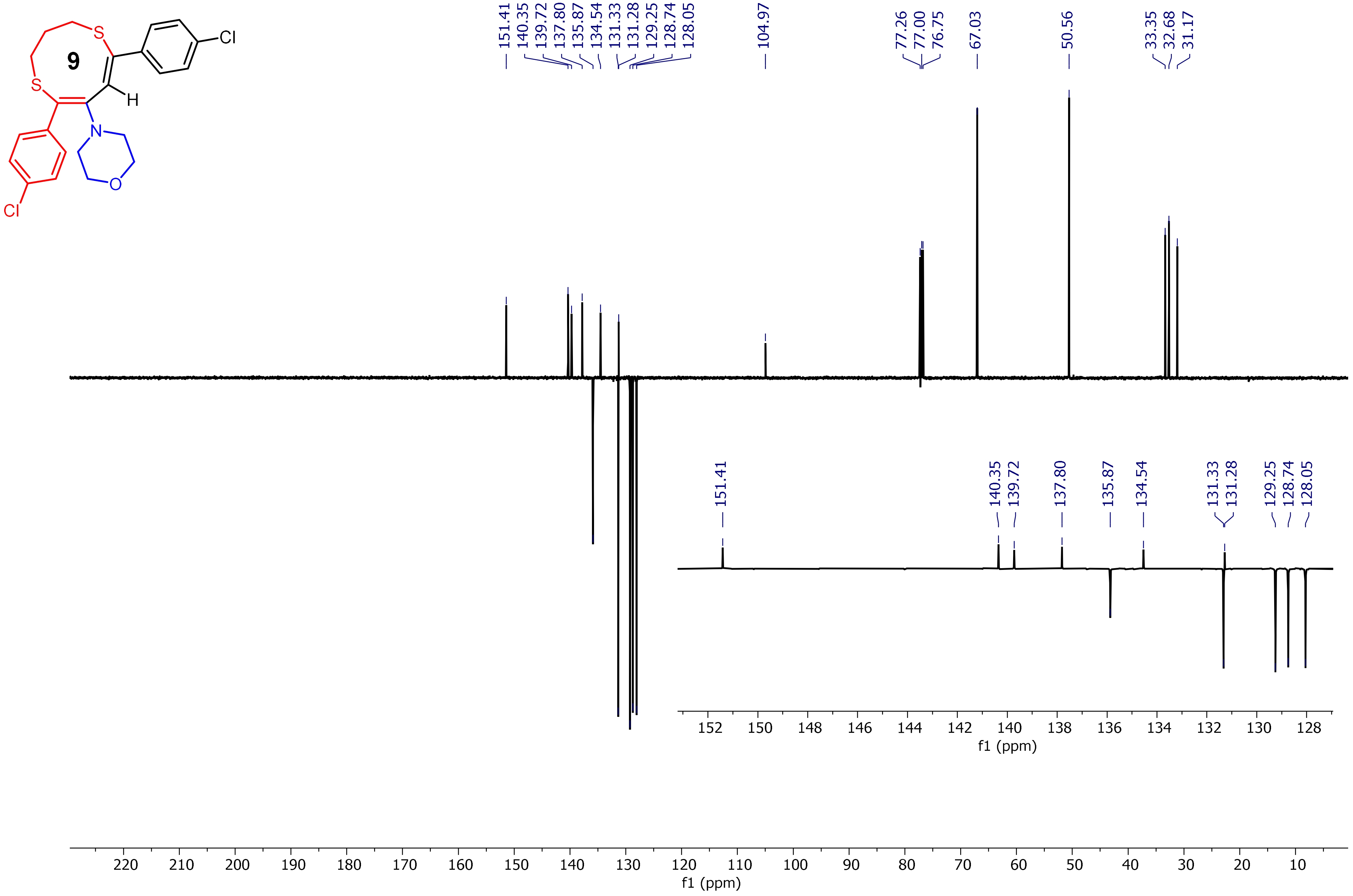

Figure S78. ^{1}H- NMR ($CDCl_3$, 500 MHz) spectrum **2l**

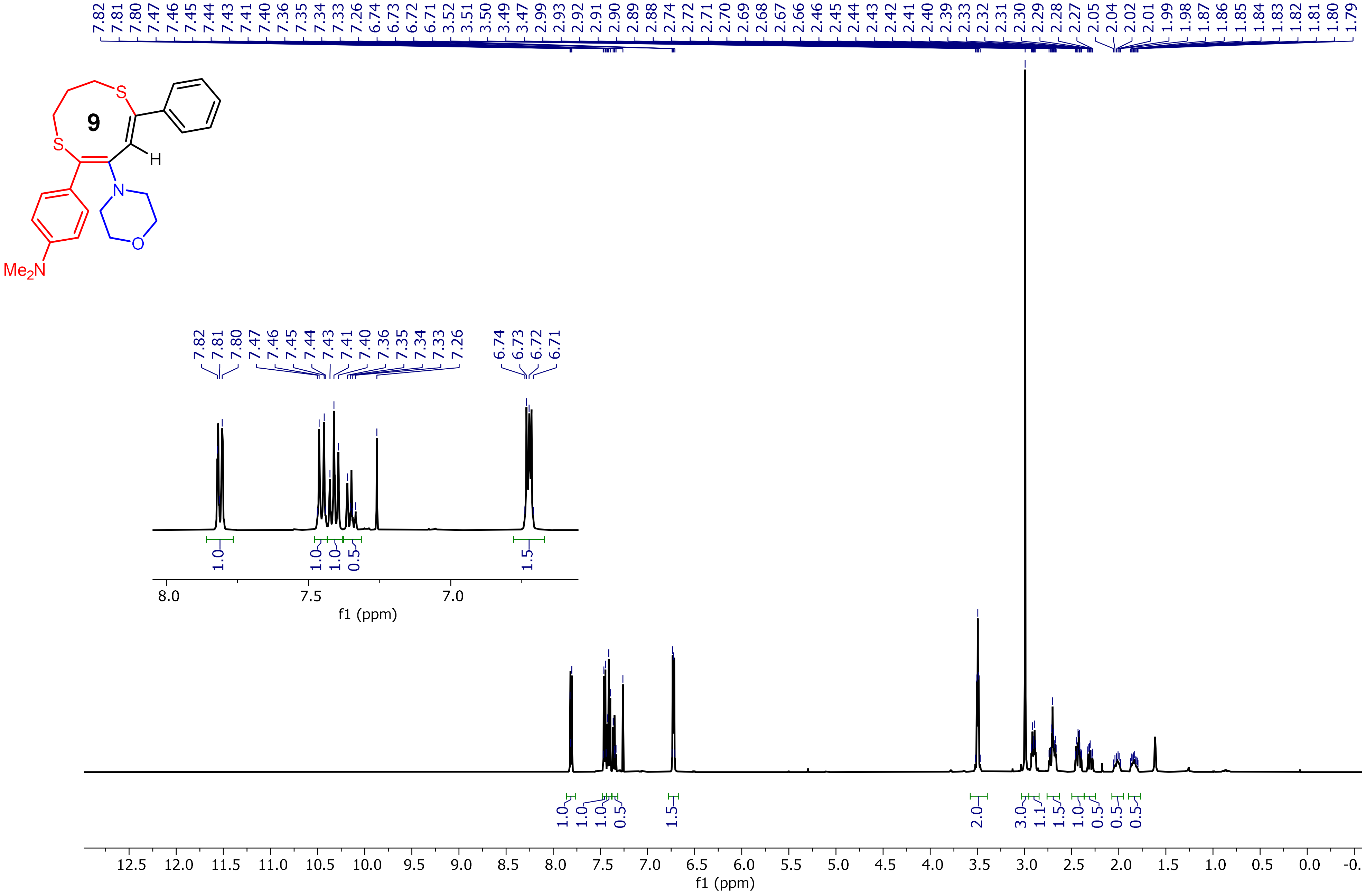

Figure S79. ^{13}C-APT NMR ($CDCl_3$, 125 MHz) spectrum **2l**

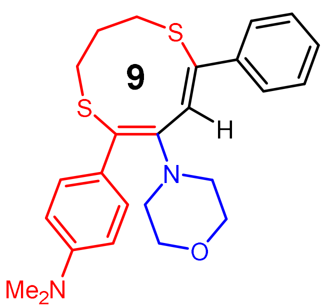

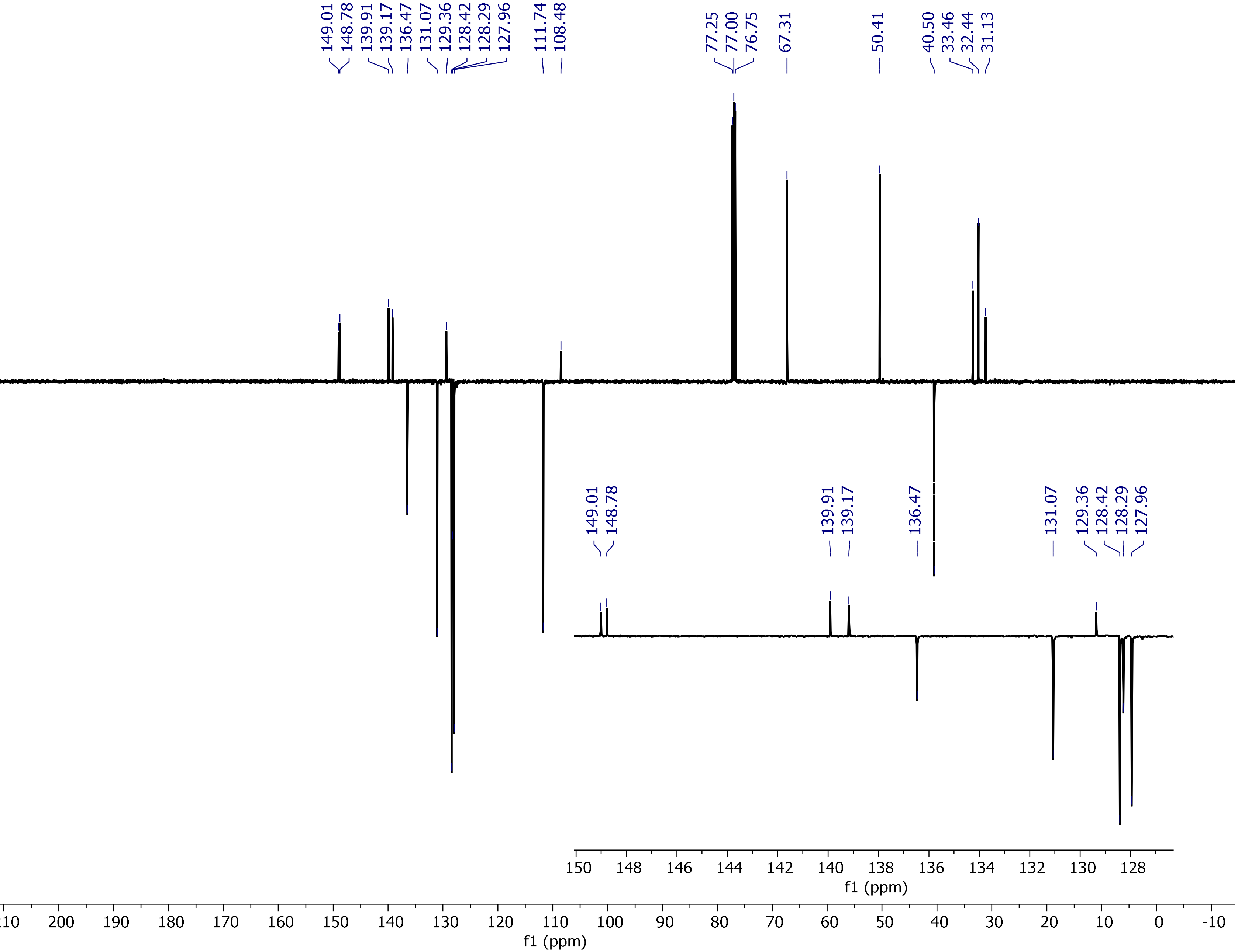

Figure S80. ^{1}H-NMR (CDCl₃, 500 MHz) spectrum **2m**

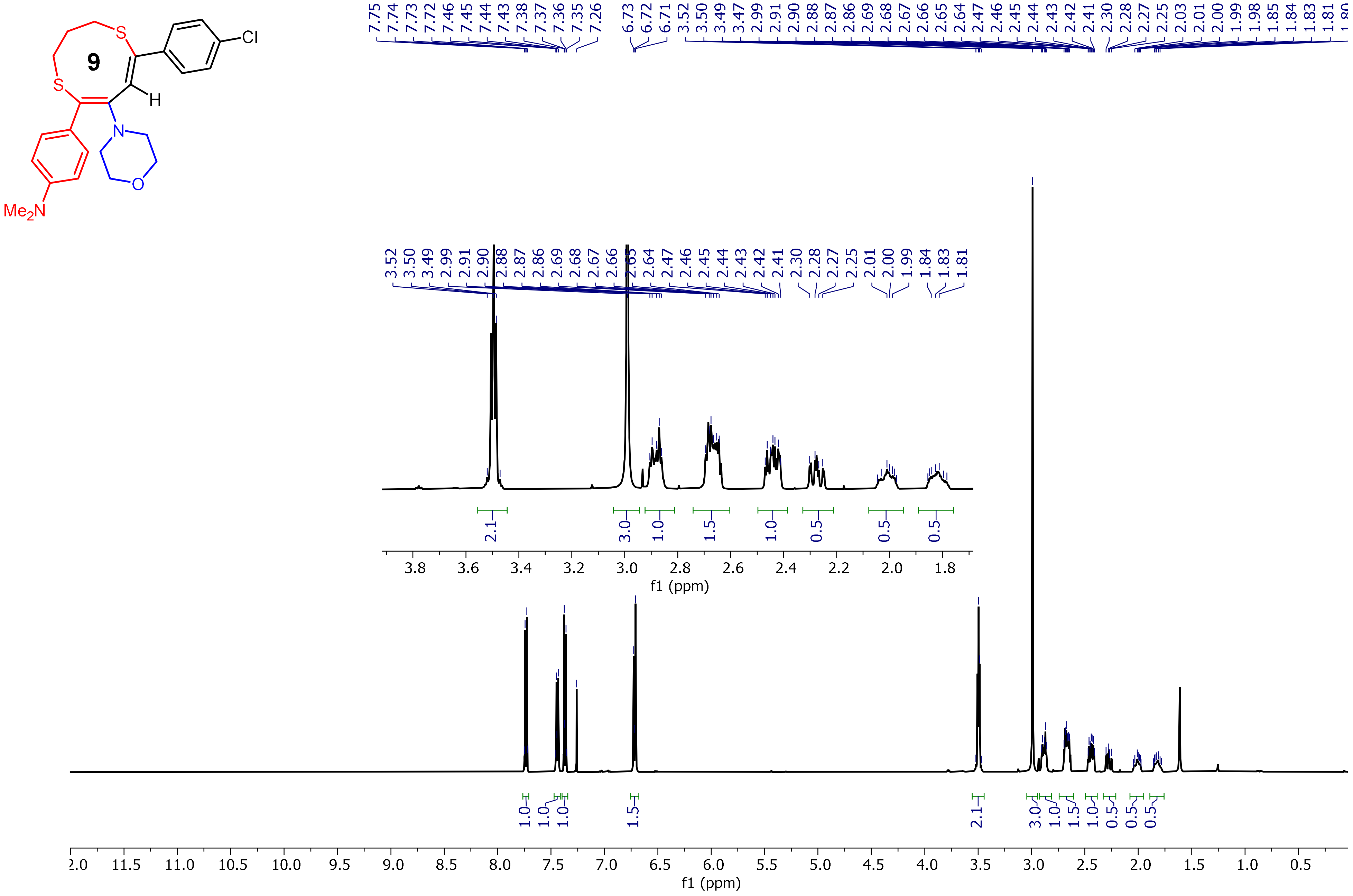

Figure S81. ^{13}C-APT NMR ($CDCl_3$, 125 MHz) spectrum **2m**

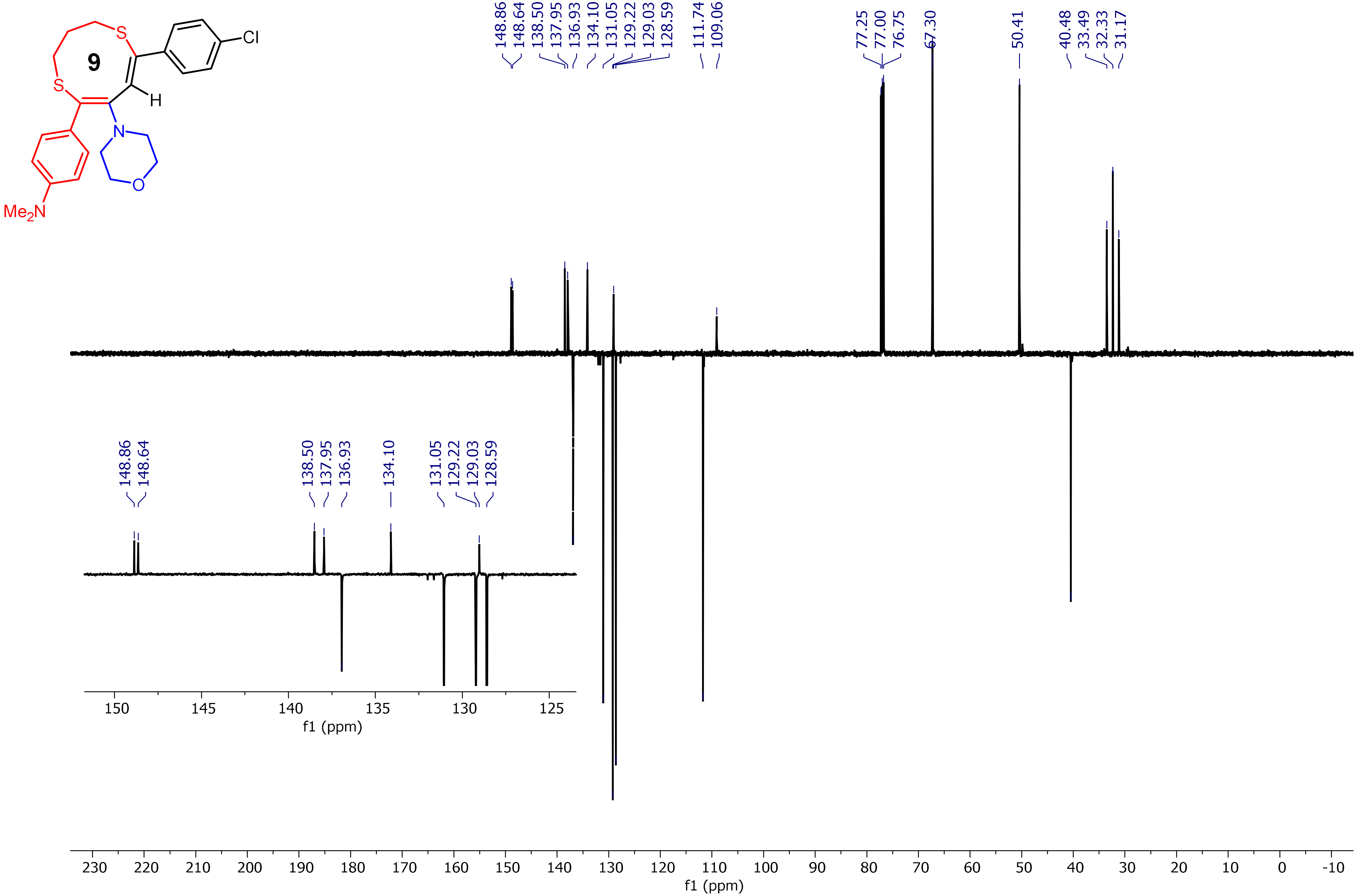

Figure S82. ^{1}H- NMR ($CDCl_3$, 500 MHz) spectrum **2n**

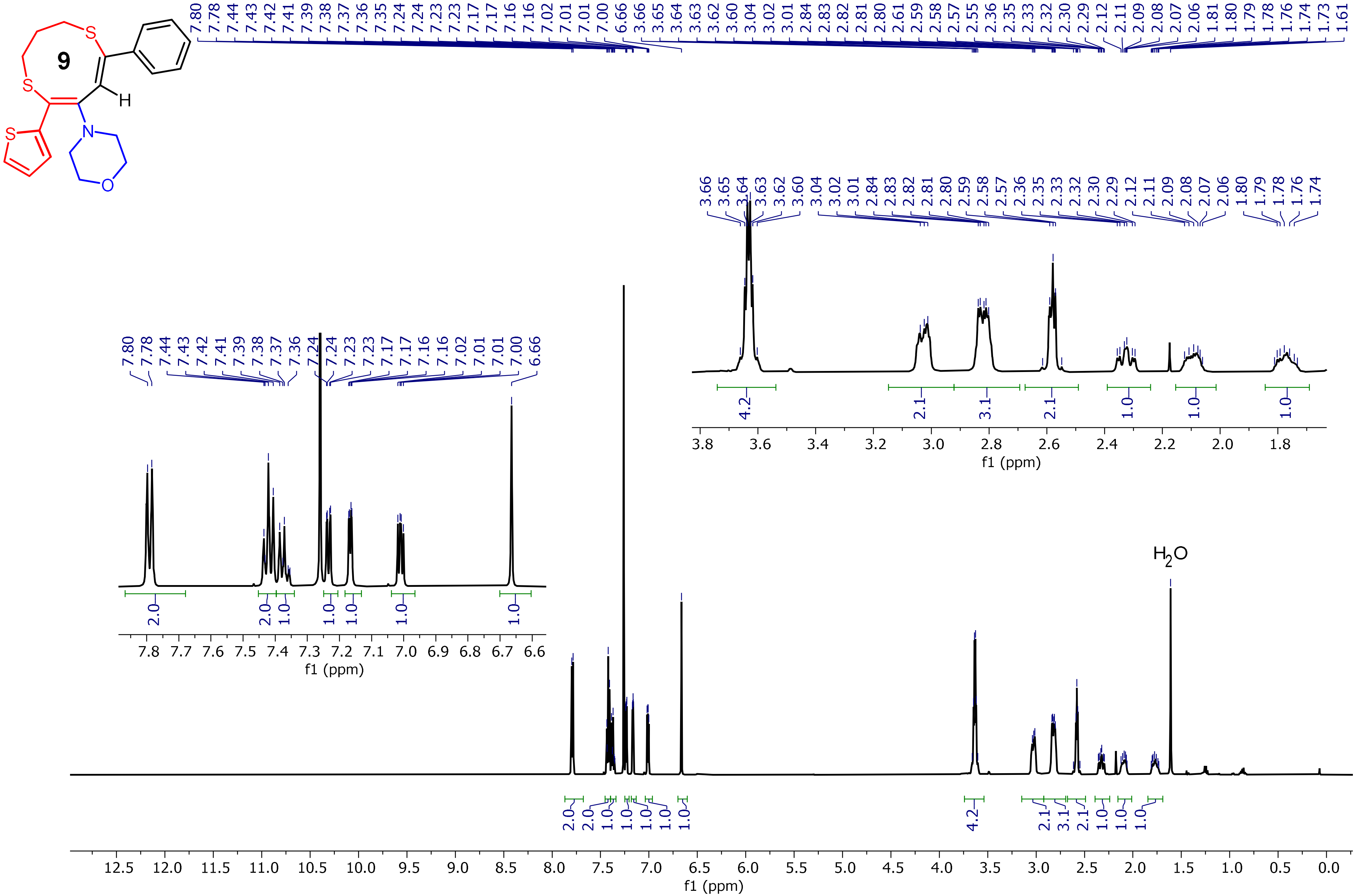

Figure S83. ^{13}C-APT NMR ($CDCl_3$, 125 MHz) spectrum **2n**

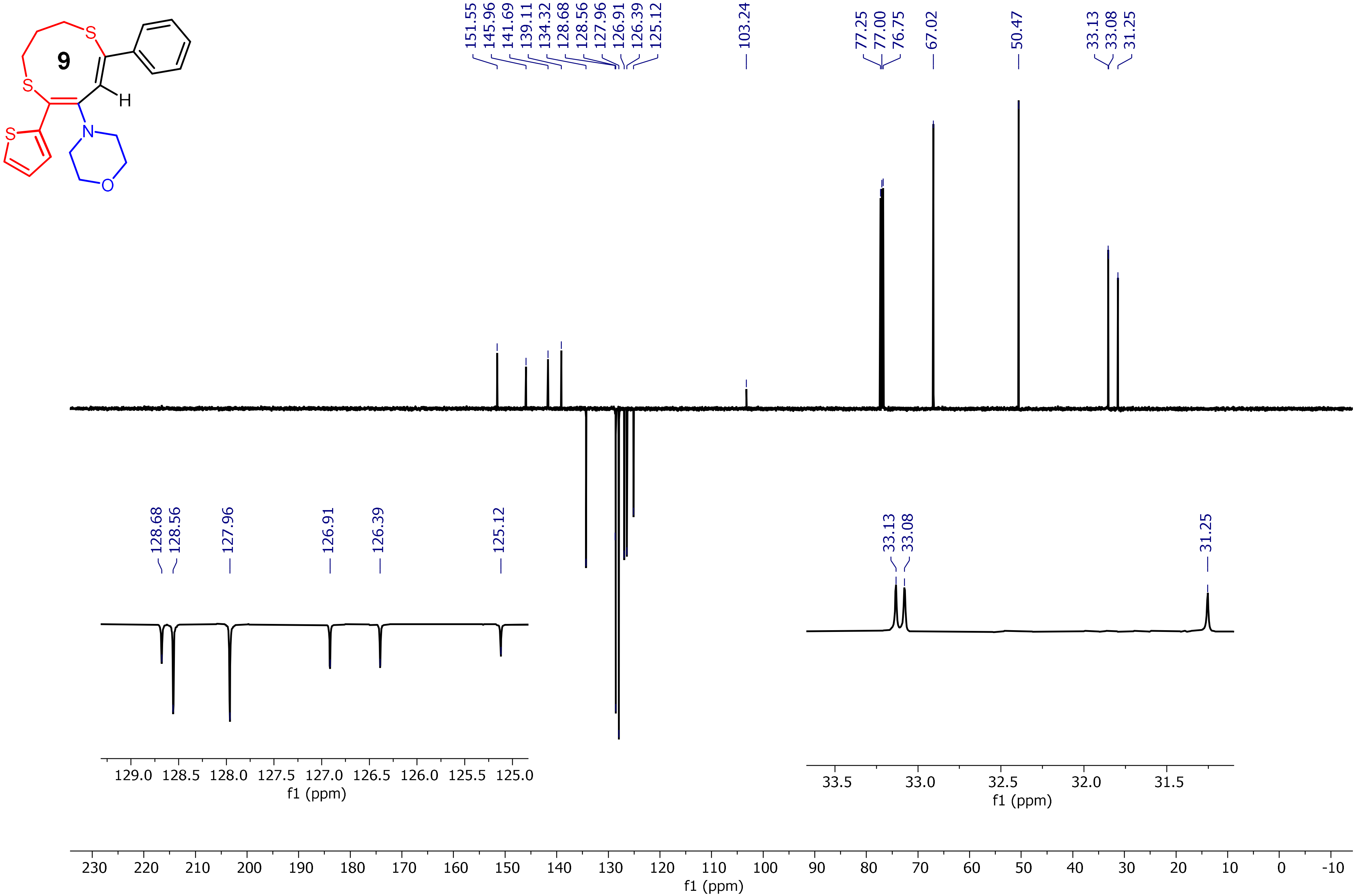

Figure S84. ^{1}H-NMR ($CDCl_3$, 500 MHz) spectrum **2o**

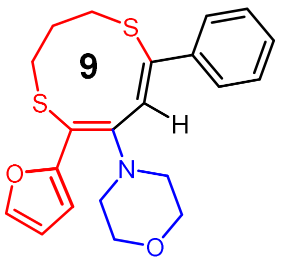

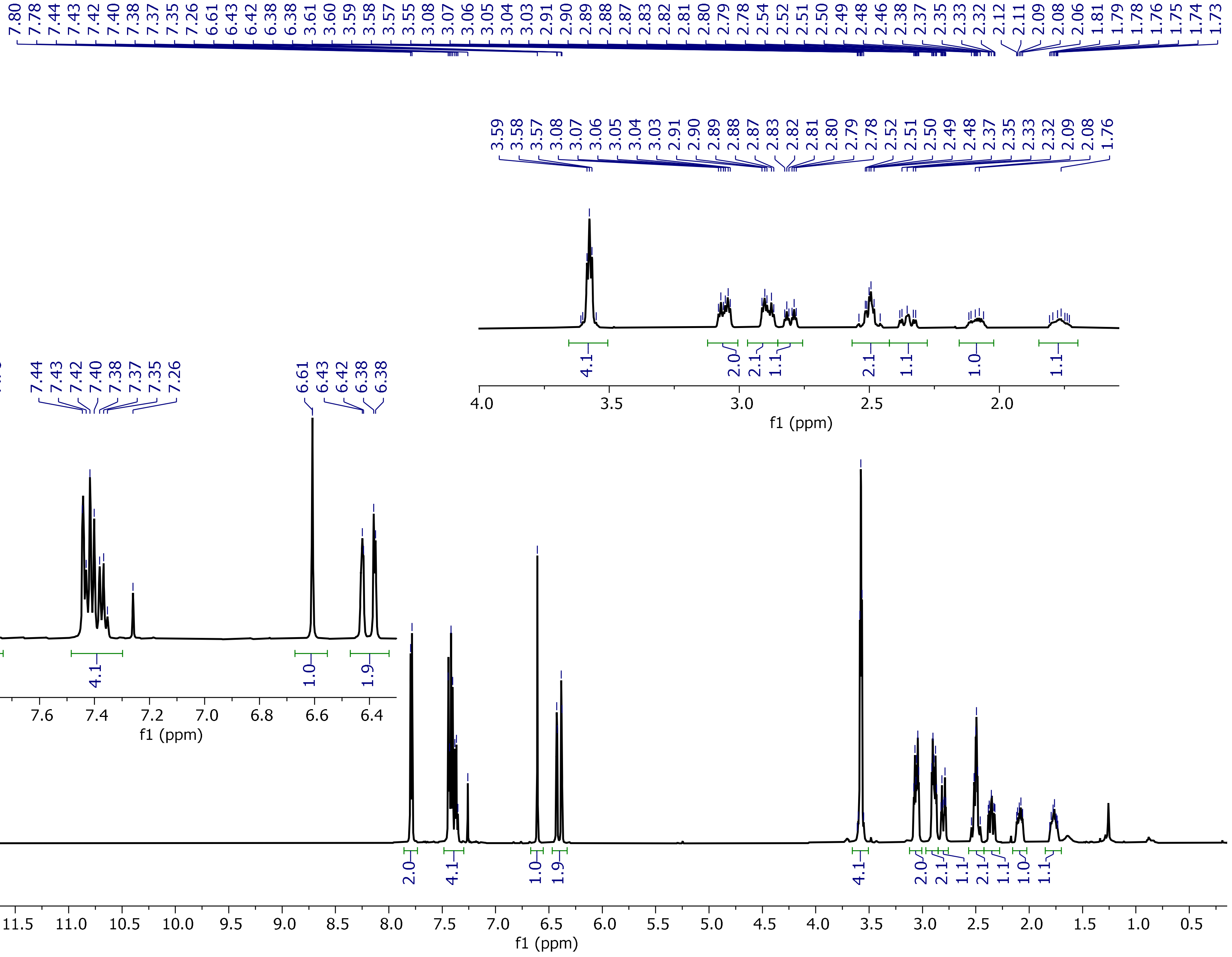

Figure S85. ^{13}C-APT NMR ($CDCl_3$, 125 MHz) spectrum **2o**

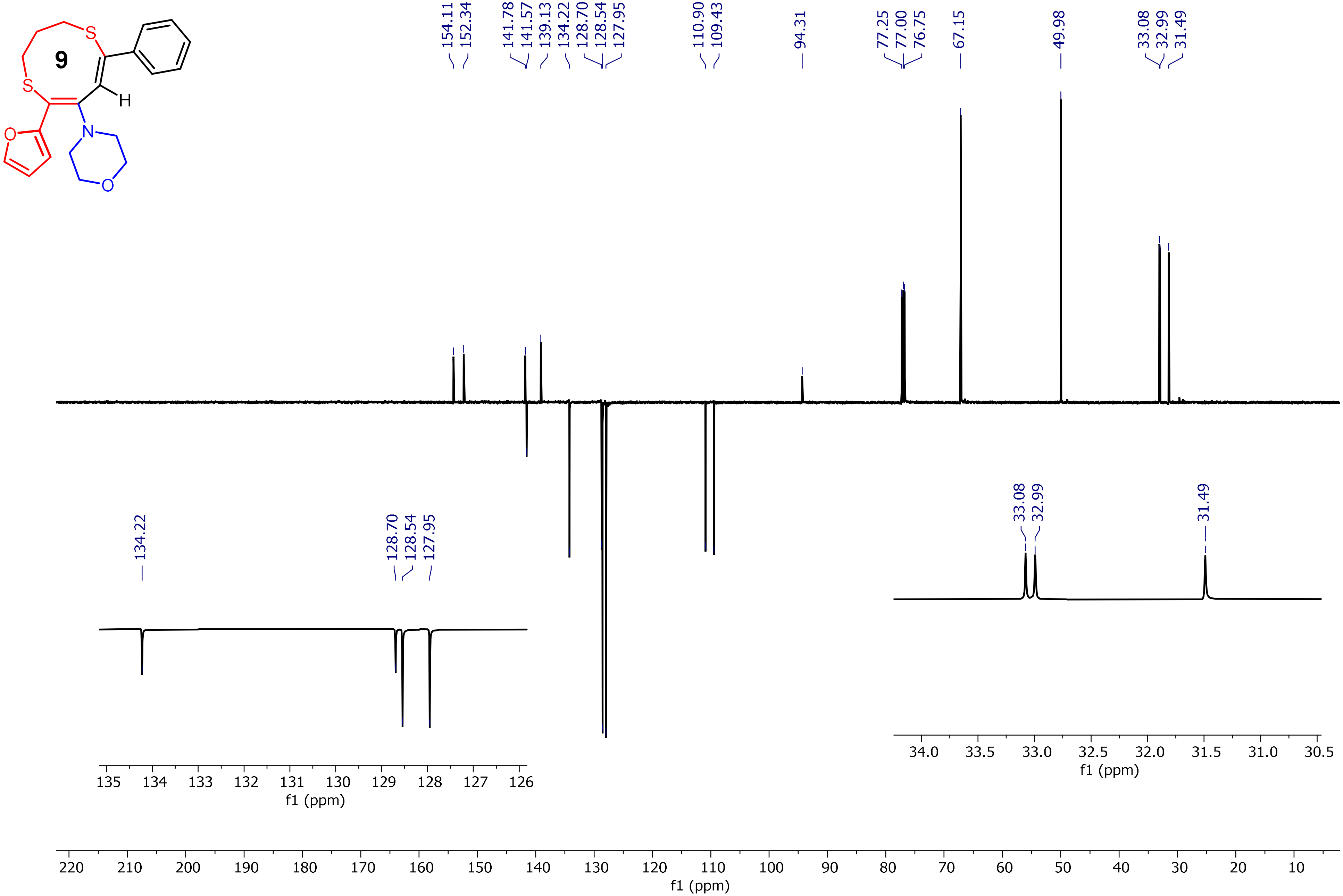

Figure S86. ^{1}H-NMR ($CDCl_3$, 500 MHz) spectrum **2p**

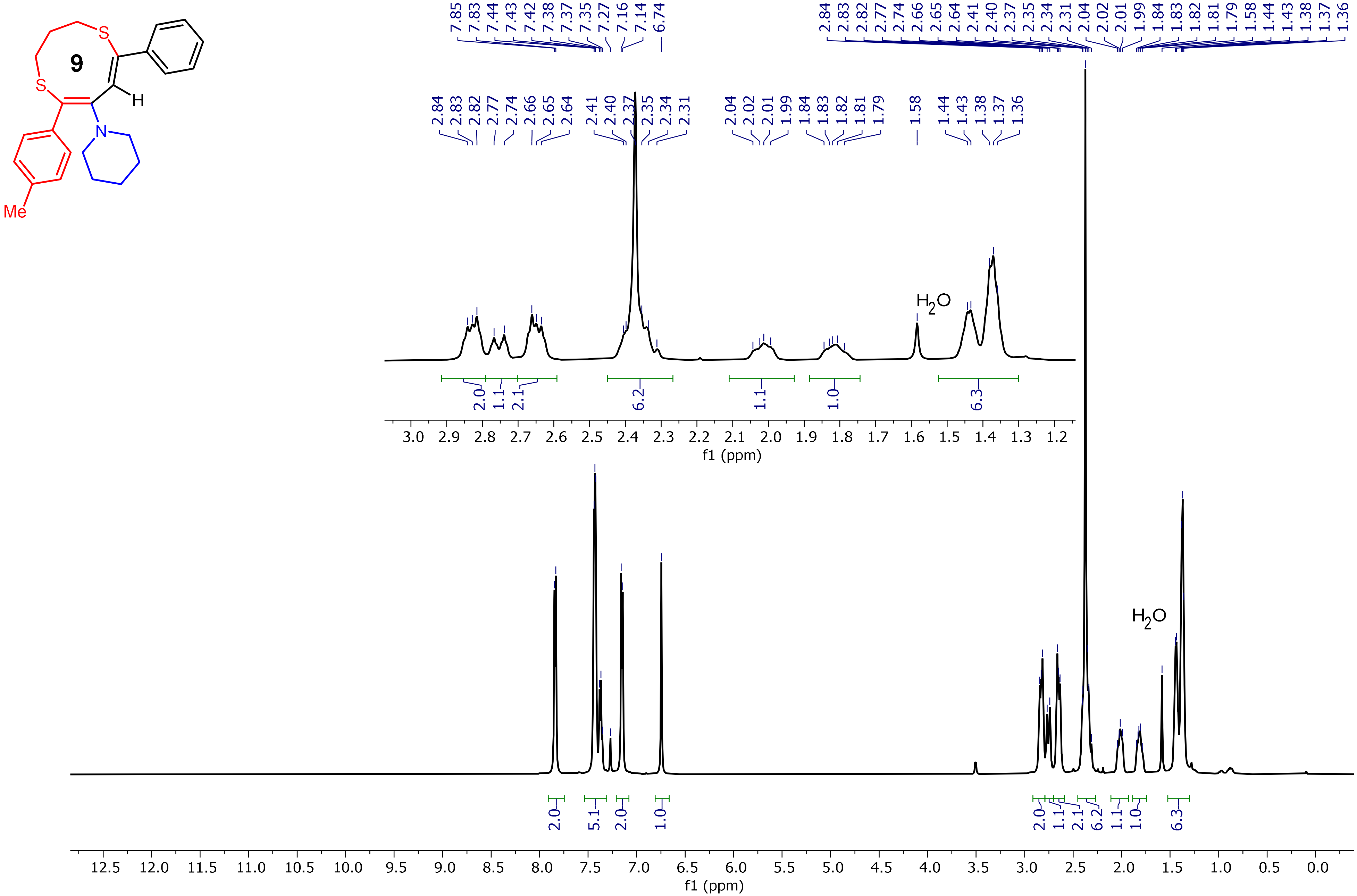

Figure S87. ^{13}C-APT NMR ($CDCl_3$, 125 MHz) spectrum **2p**

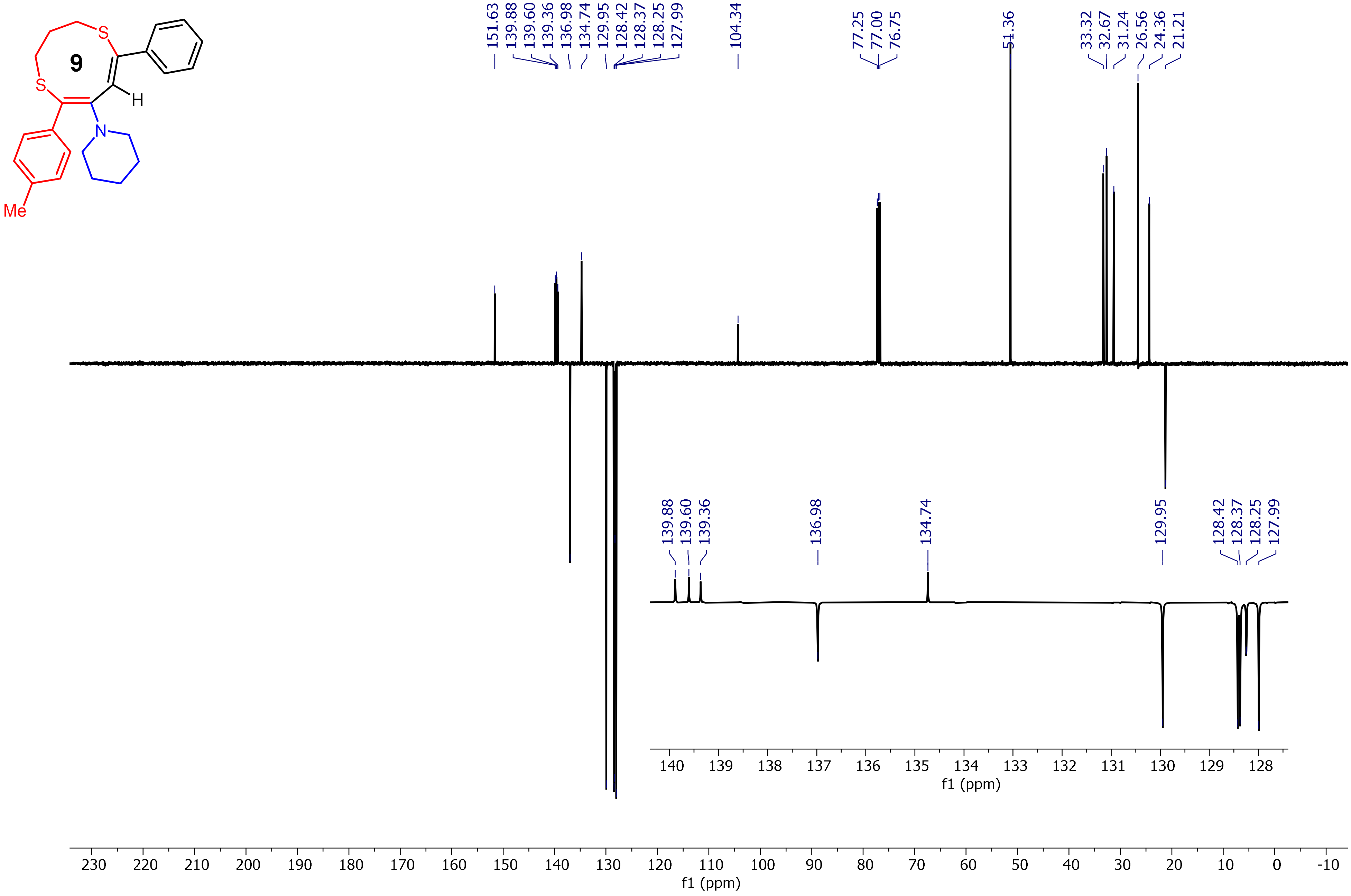

Figure S88. ^{1}H- NMR ($CDCl_3$, 500 MHz) spectrum **2q**

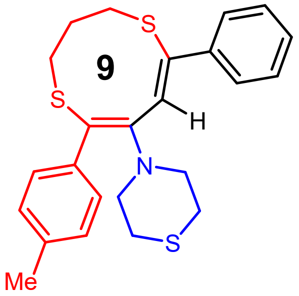

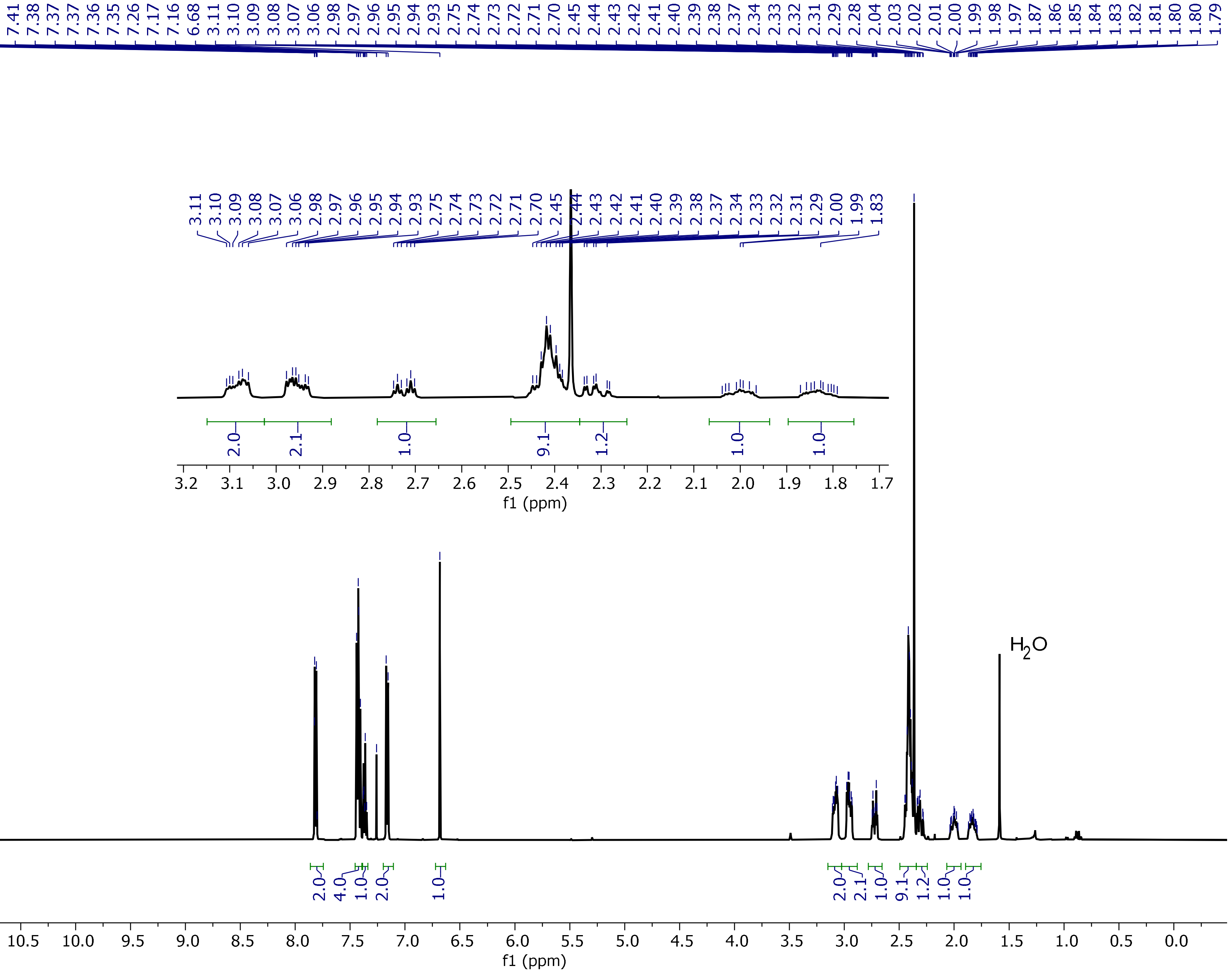

Figure S89. ^{13}C-APT NMR ($CDCl_3$, 125 MHz) spectrum **2q**

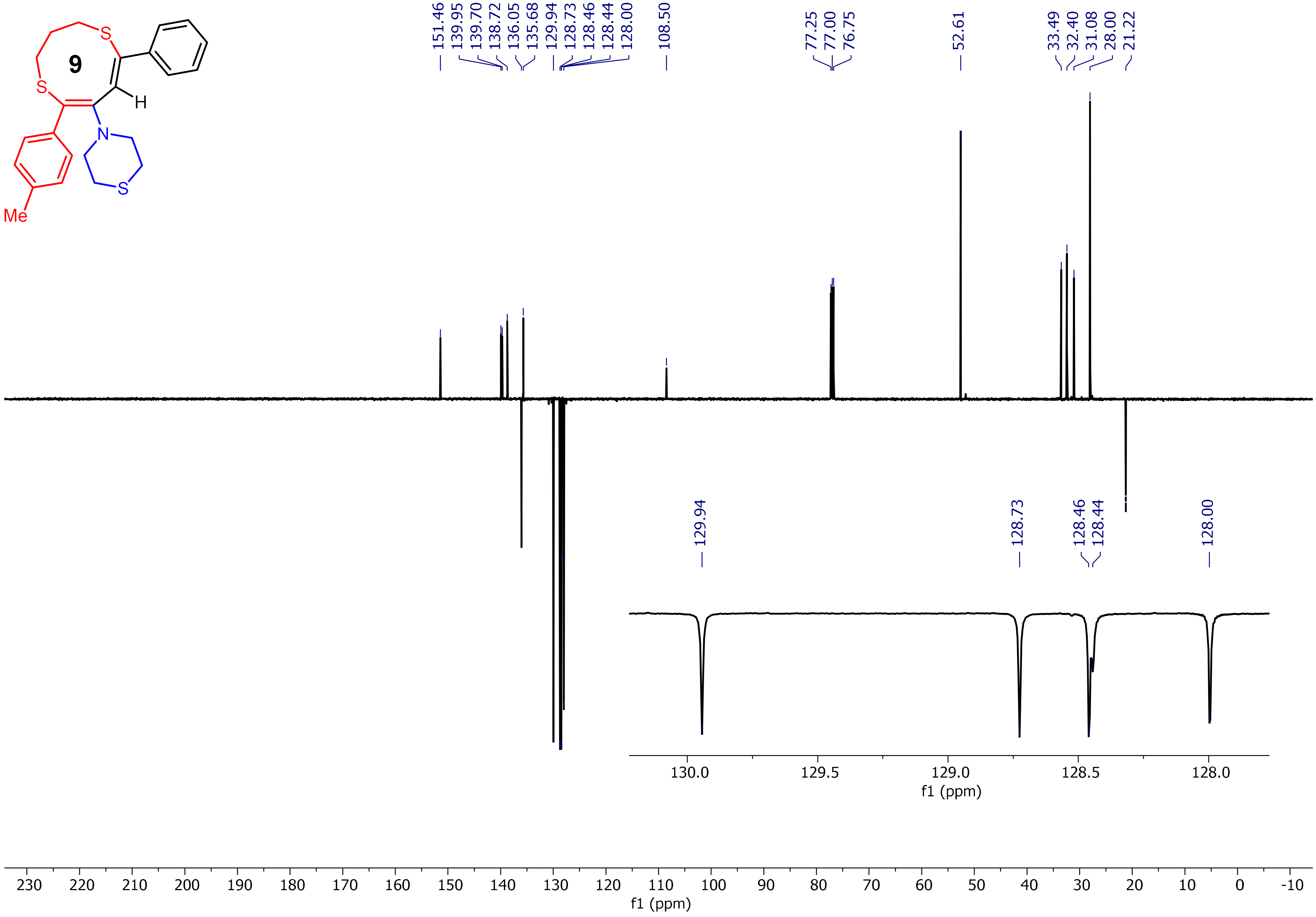

Figure S90. ^{1}H-NMR ($CDCl_3$, 500 MHz) spectrum **2r**

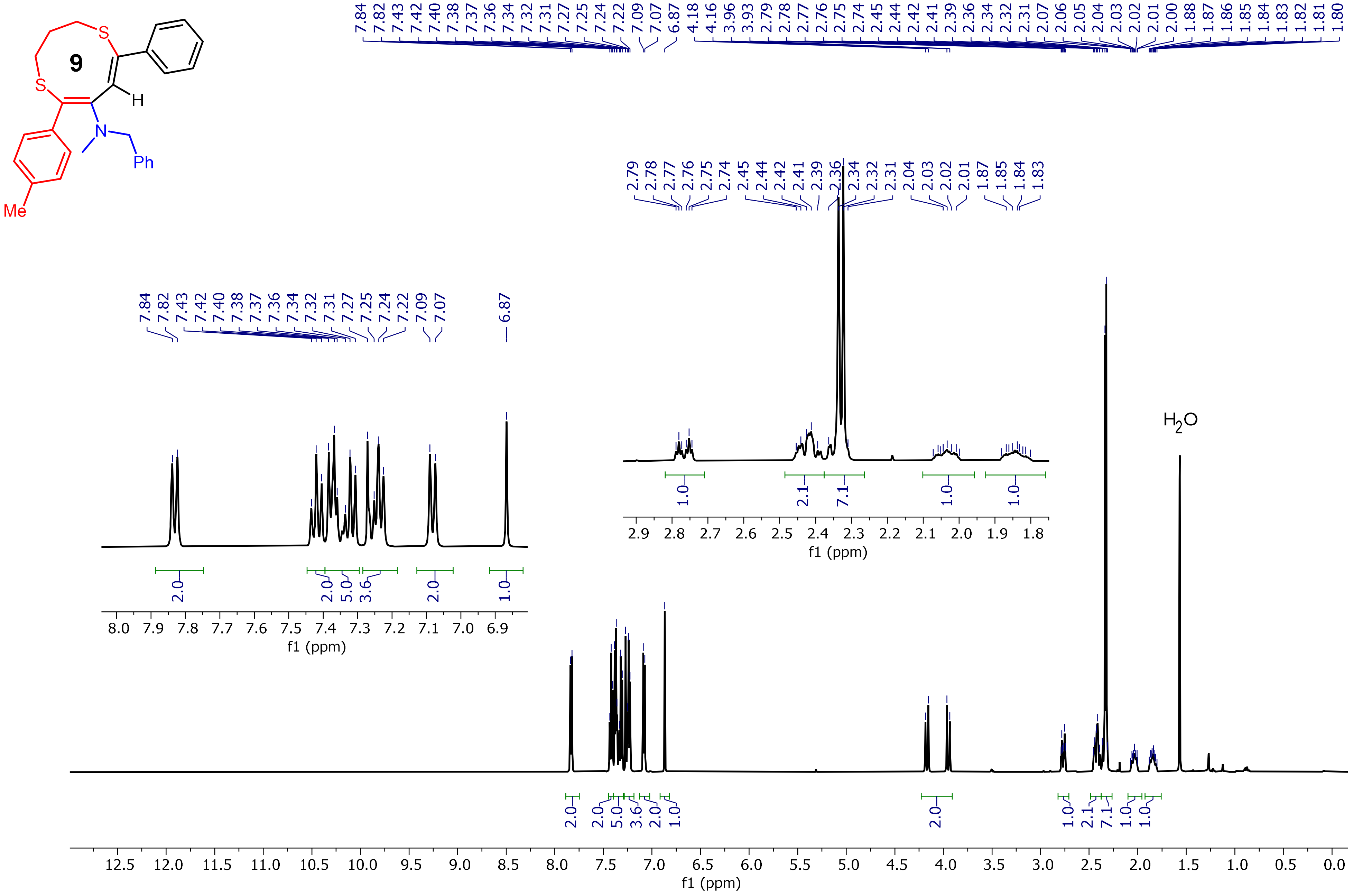

Figure S91. ^{13}C-APT NMR ($CDCl_3$, 125 MHz) spectrum **2r**

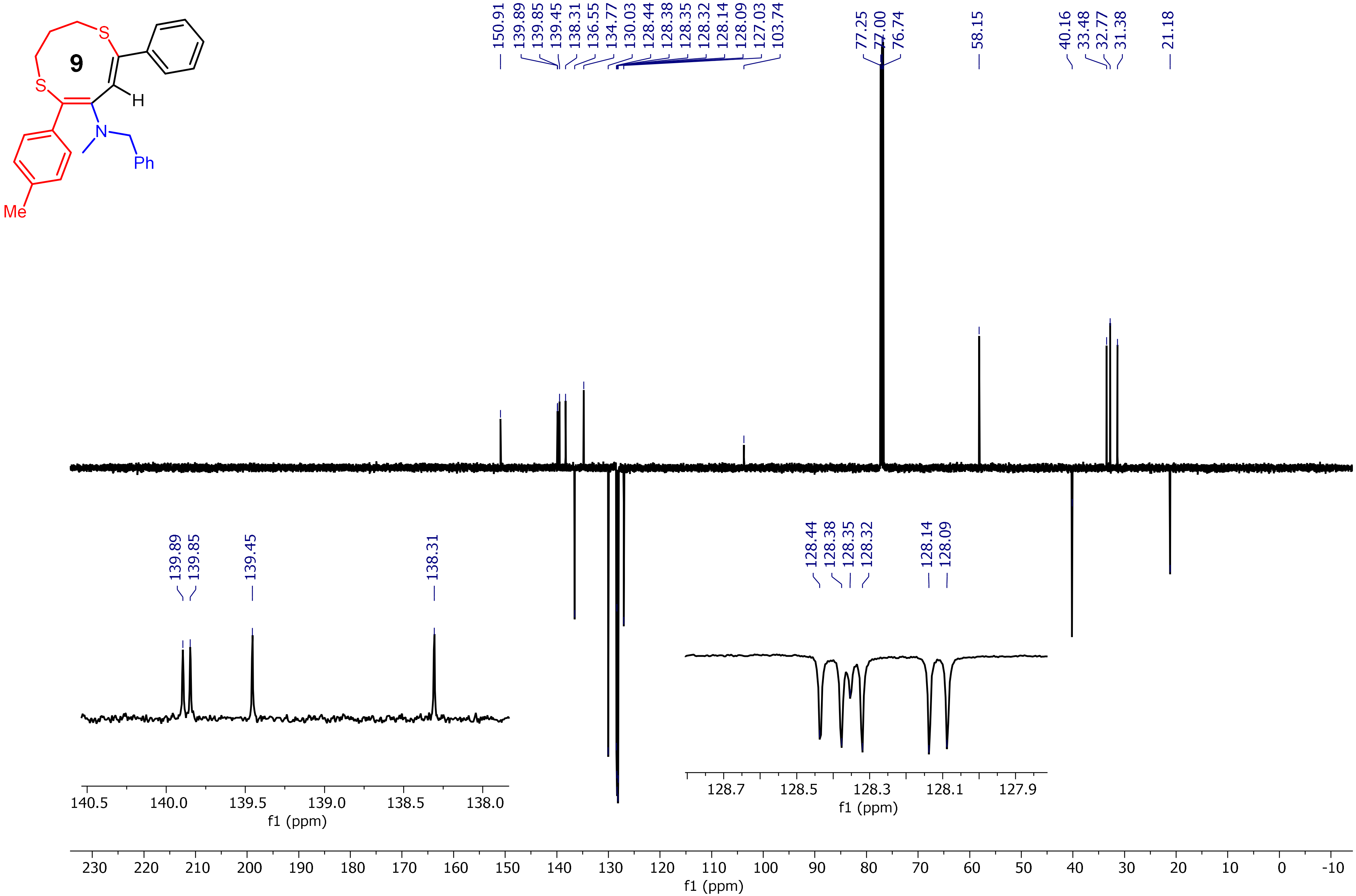

Figure S92. ^{1}H- NMR ($CDCl_3$, 500 MHz) spectrum **2s**

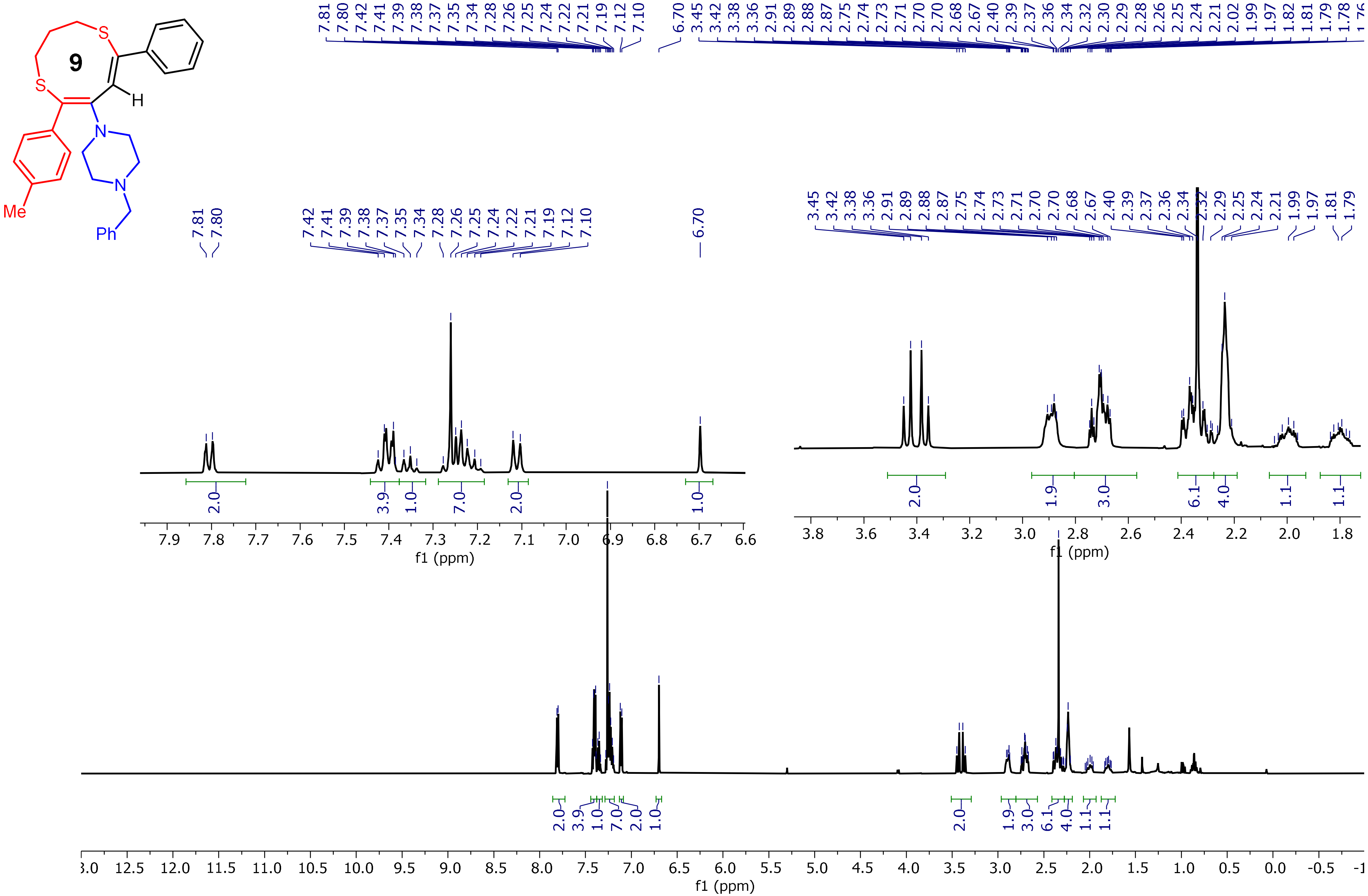

Figure S93. ^{13}C-APT NMR ($CDCl_3$, 125 MHz) spectrum **2s**

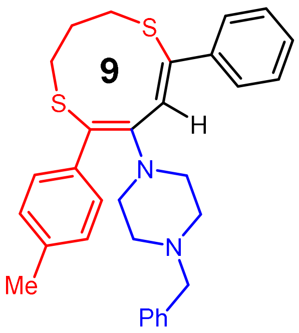

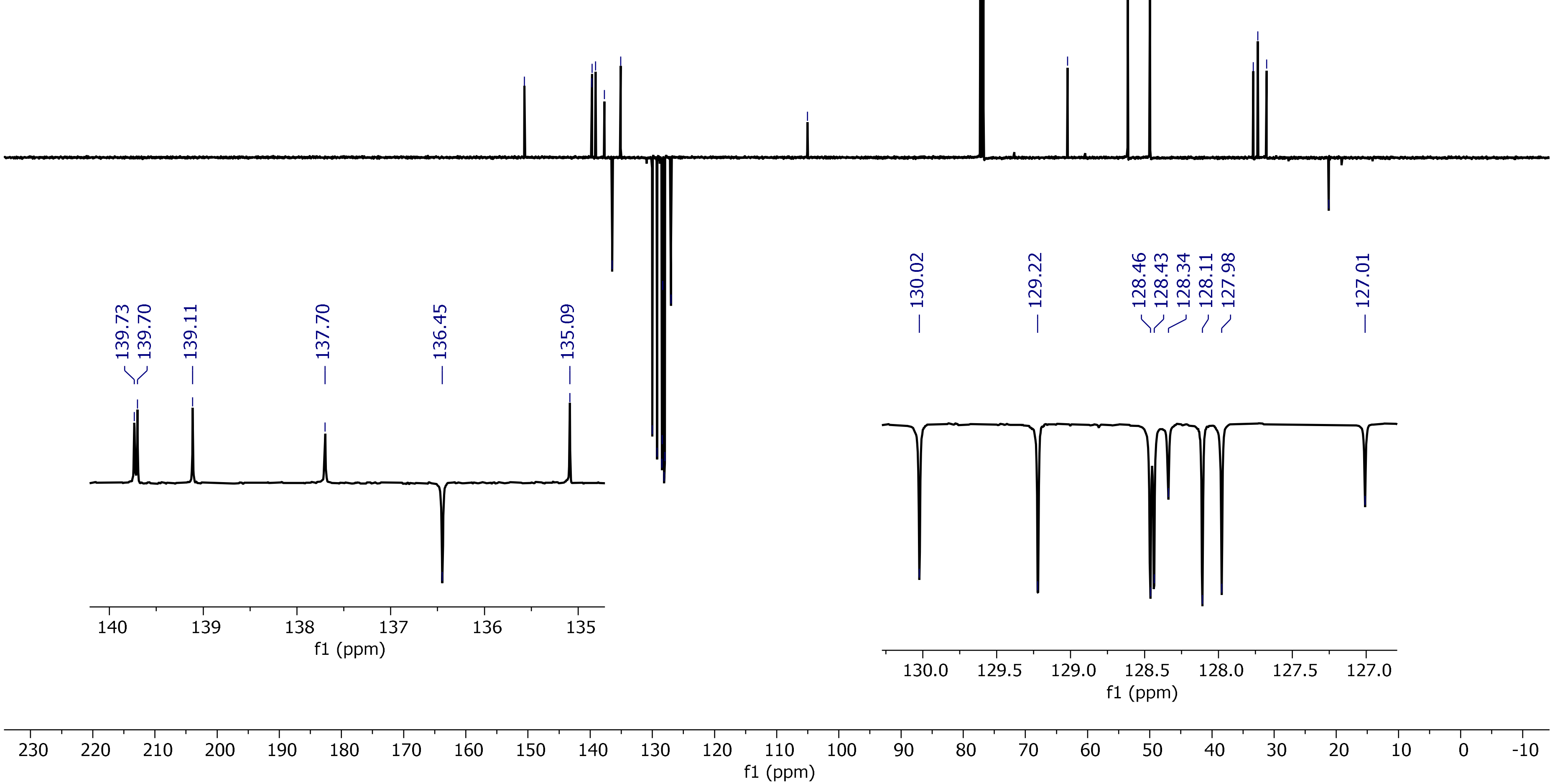

Figure S94. ^{1}H-NMR ($CDCl_3$, 500 MHz) spectrum **2t**

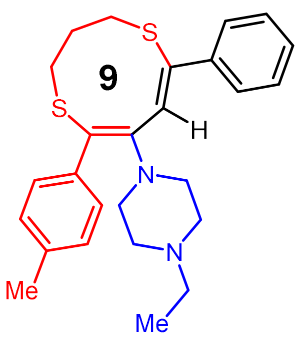

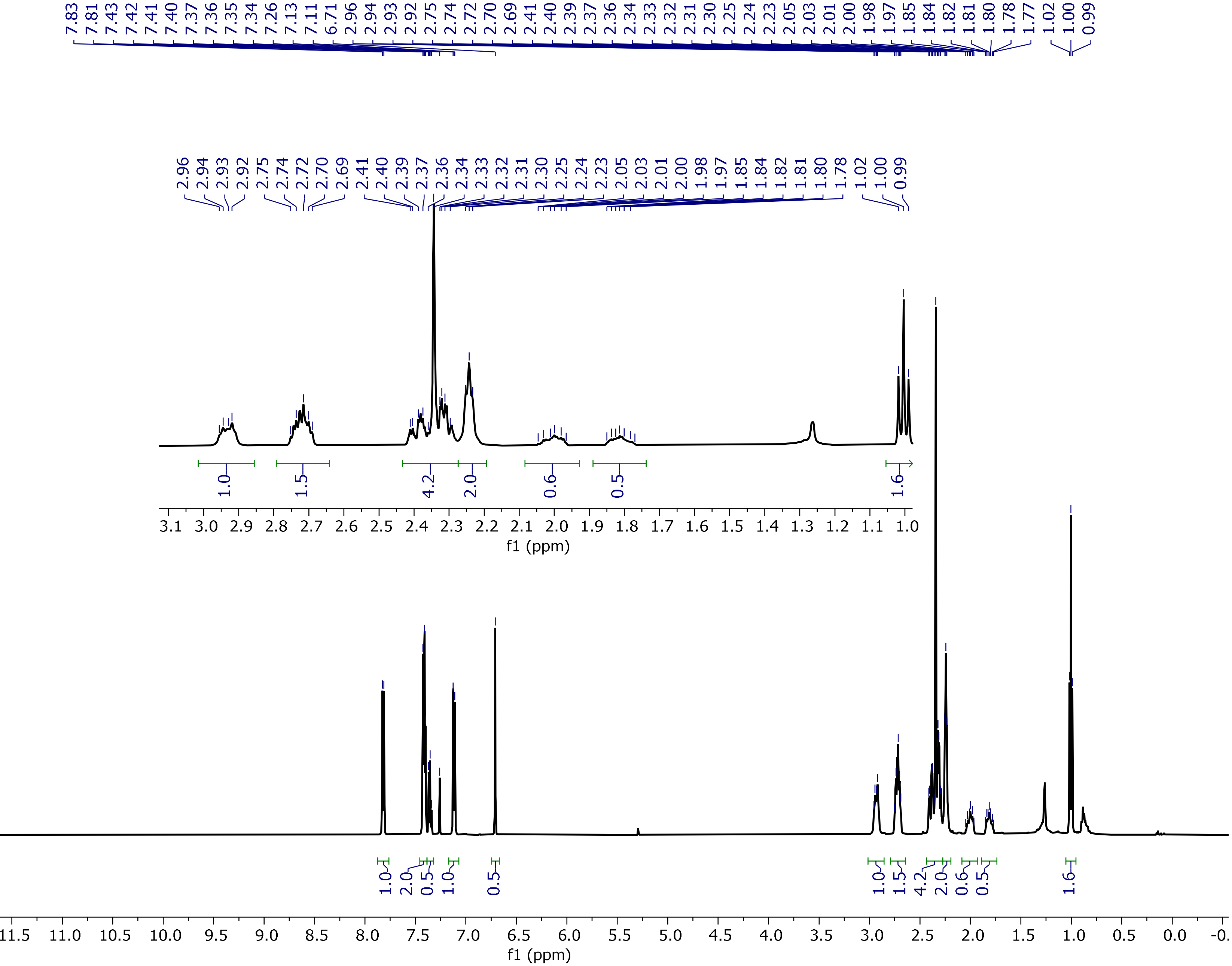

Figure S95. ^{13}C-APT NMR ($CDCl_3$, 125 MHz) spectrum **2t**

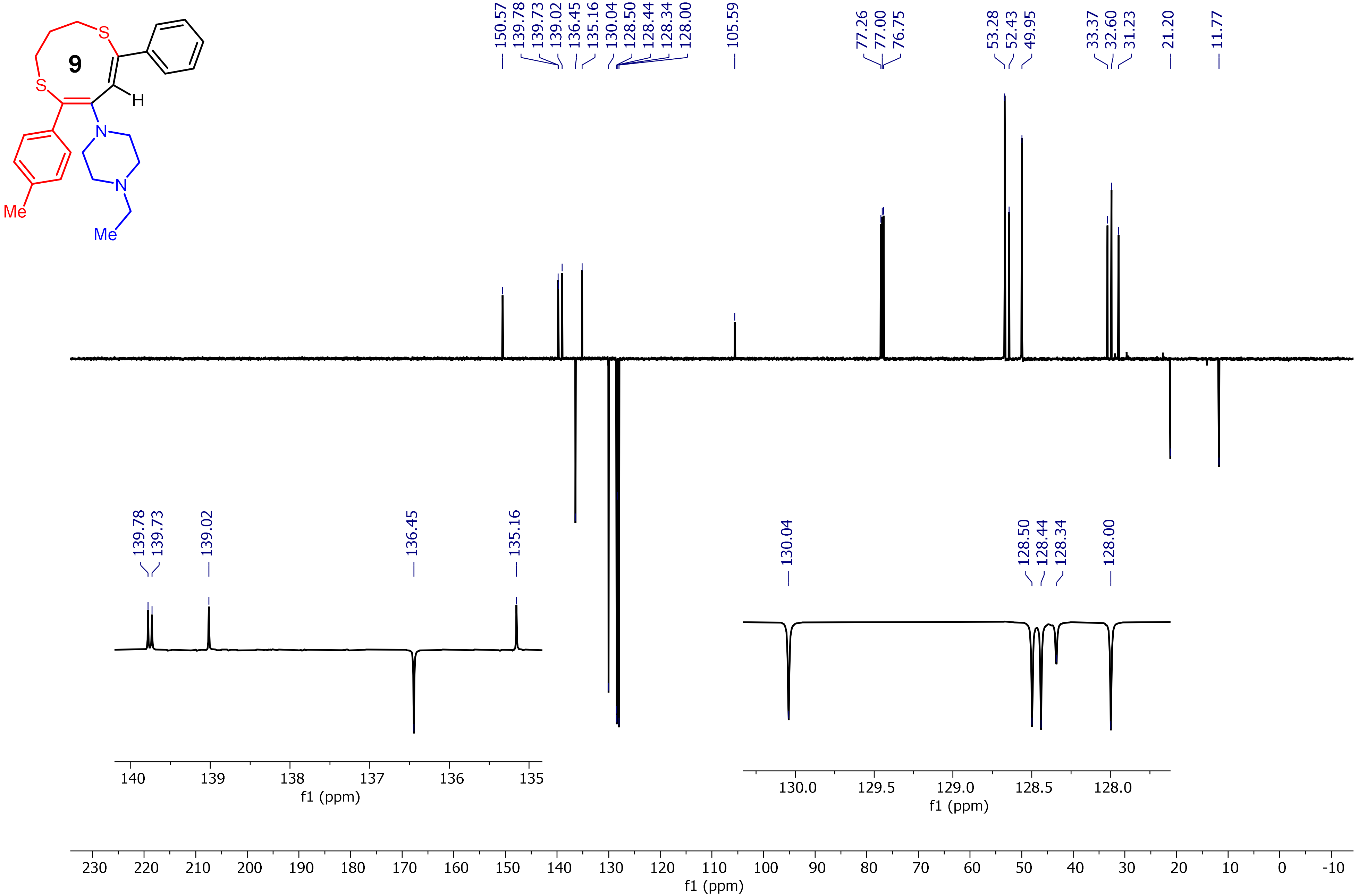

Figure S96. ^{1}H-NMR ($CDCl_3$, 500 MHz) spectrum **2u**

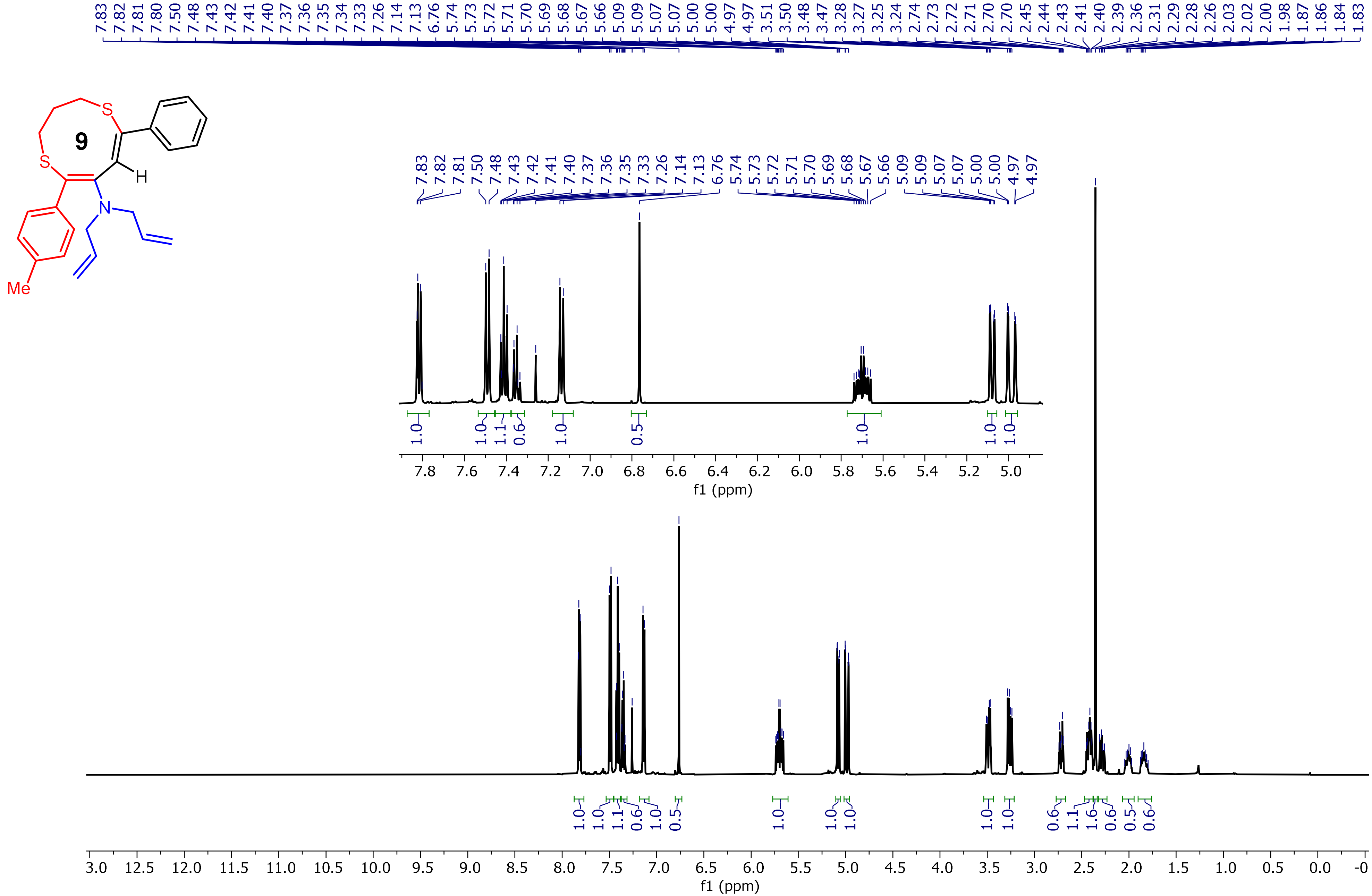

Figure S97. ^{13}C-APT NMR ($CDCl_3$, 125 MHz) spectrum **2u**

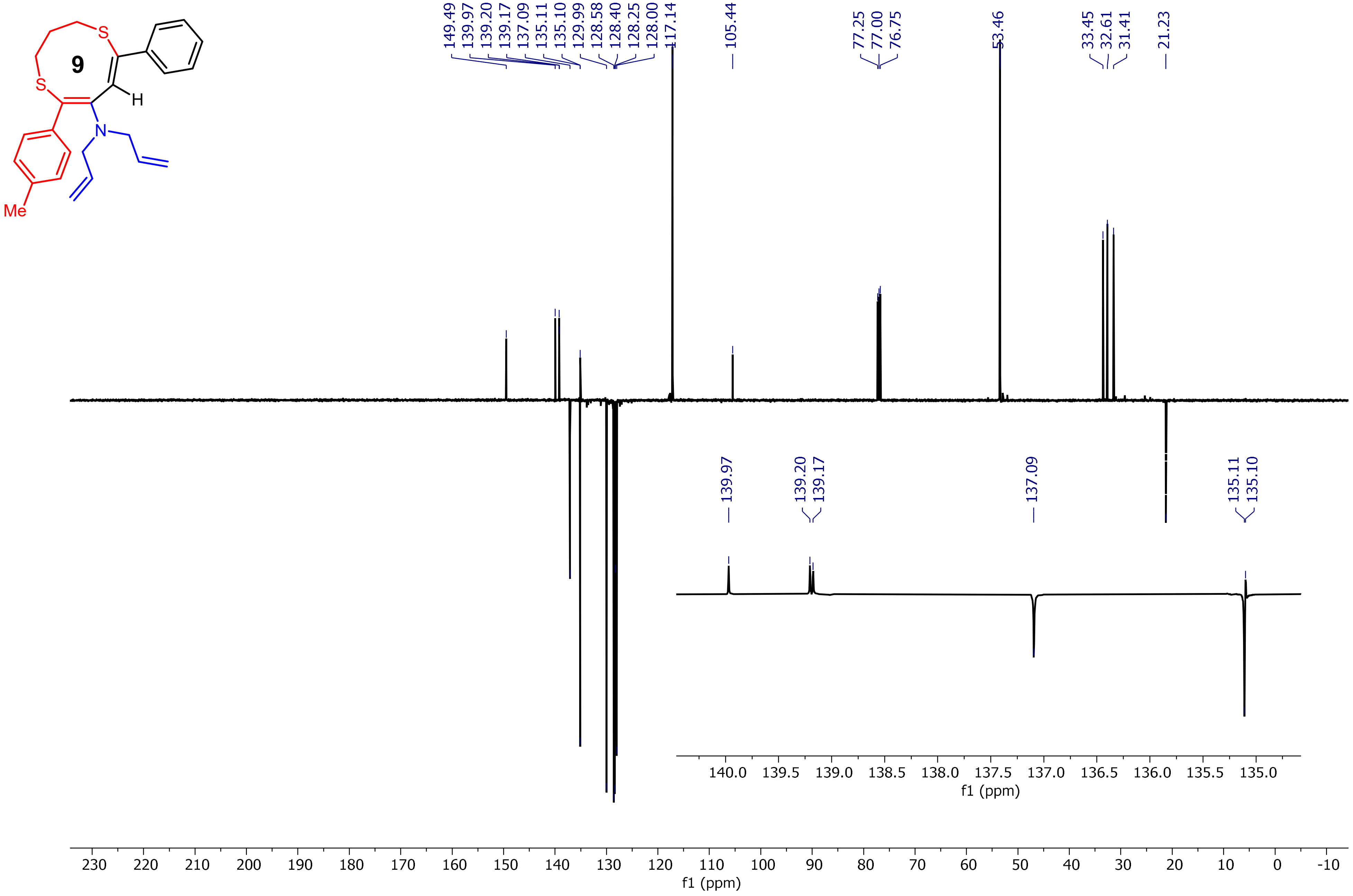

Figure S98. ^{1}H-NMR ($CDCl_3$, 500 MHz) spectrum **2v**

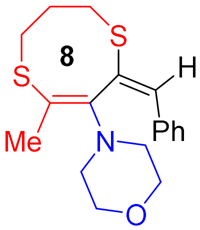

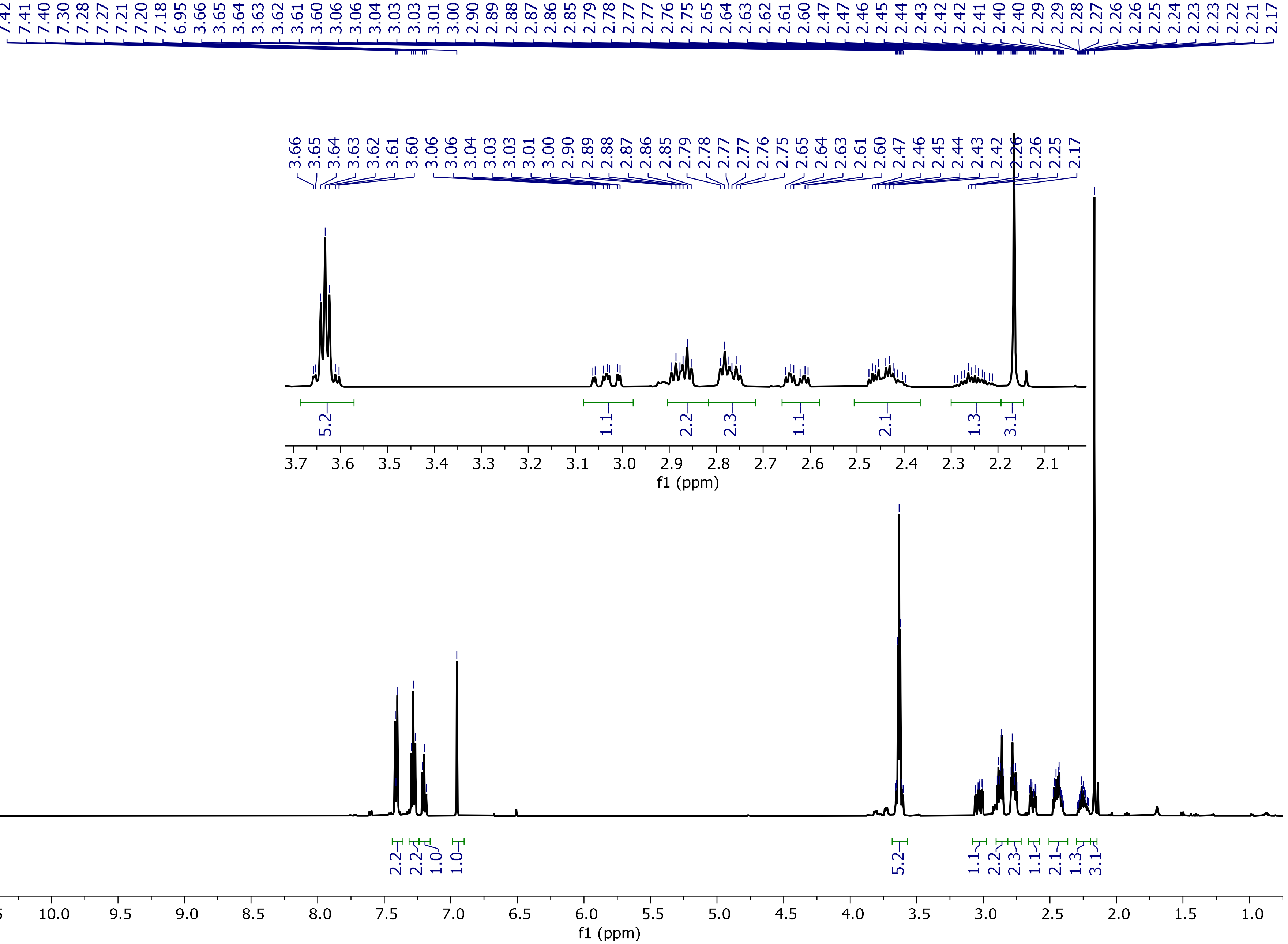

Figure S99. ^{1}H-NMR ($CDCl_3$, 500 MHz) spectrum **D-2v**

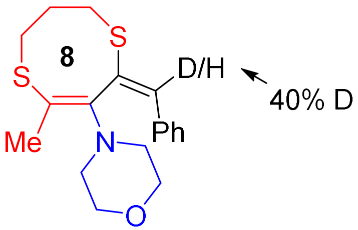

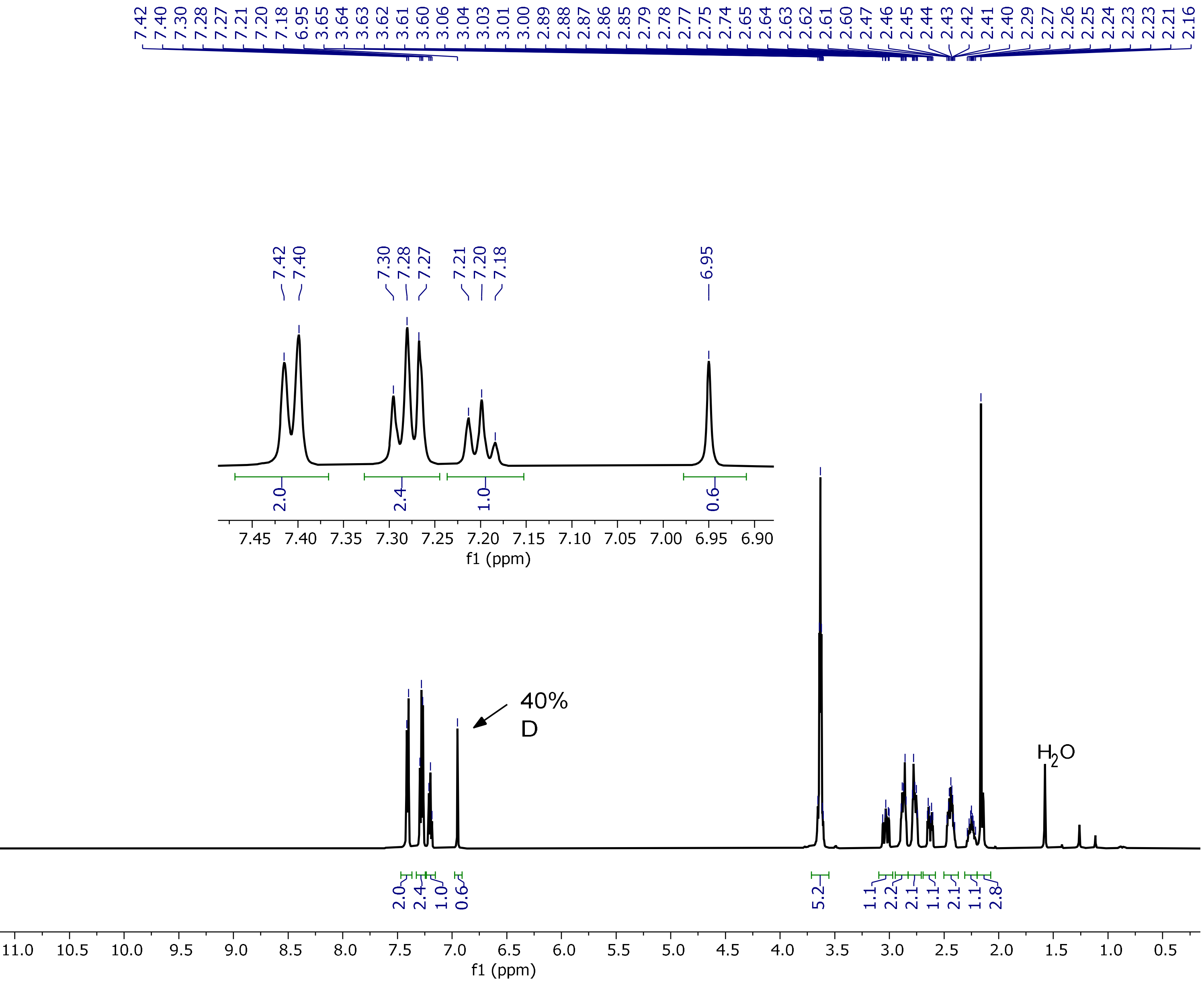

Figure S100. ^{13}C-APT NMR ($CDCl_3$, 125 MHz) spectrum **2v**

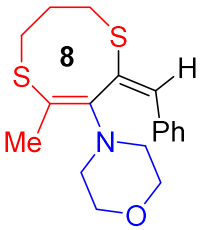

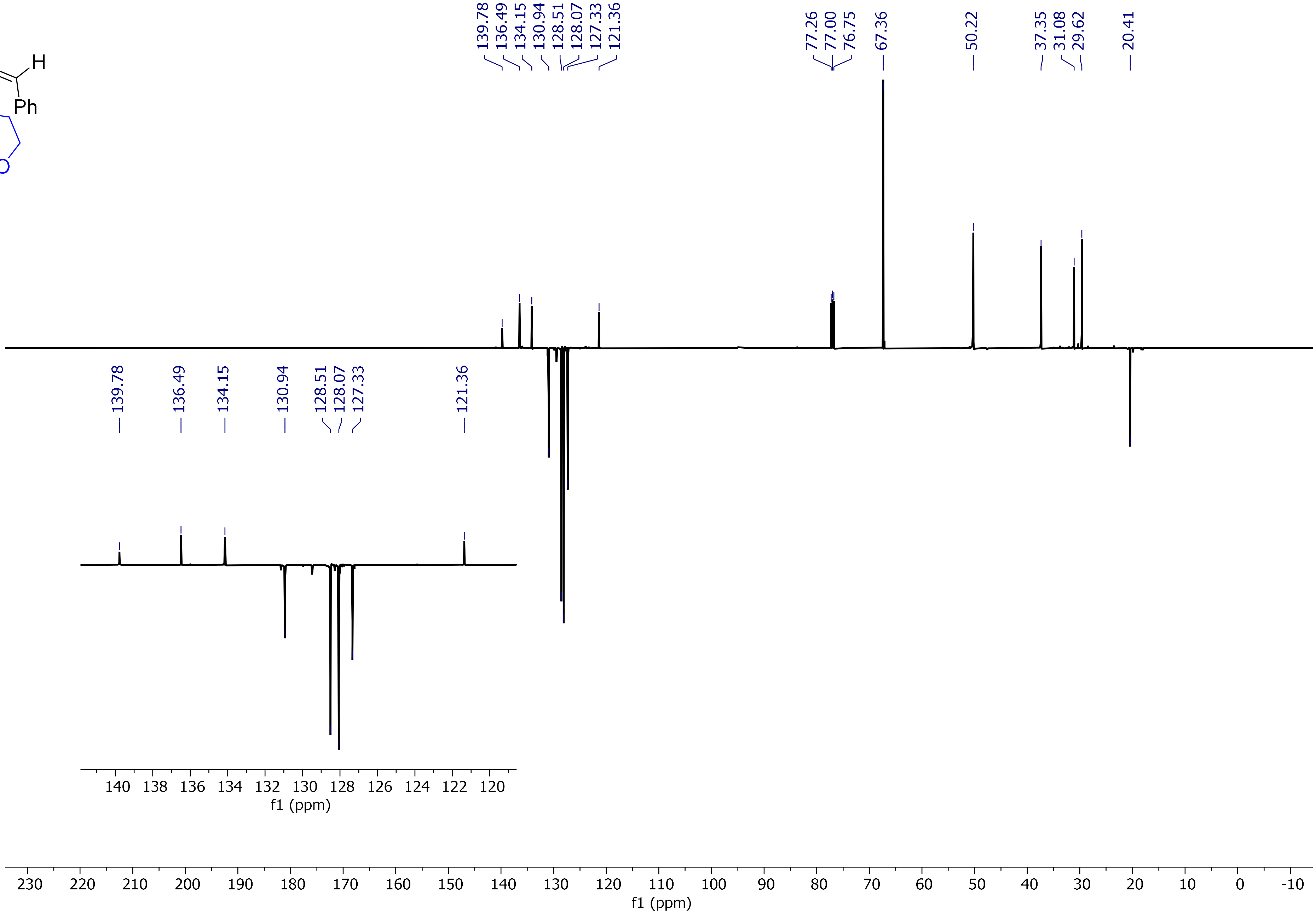

Figure S101. ^{13}C-APT NMR ($CDCl_3$, 125 MHz) spectrum **D-2v**

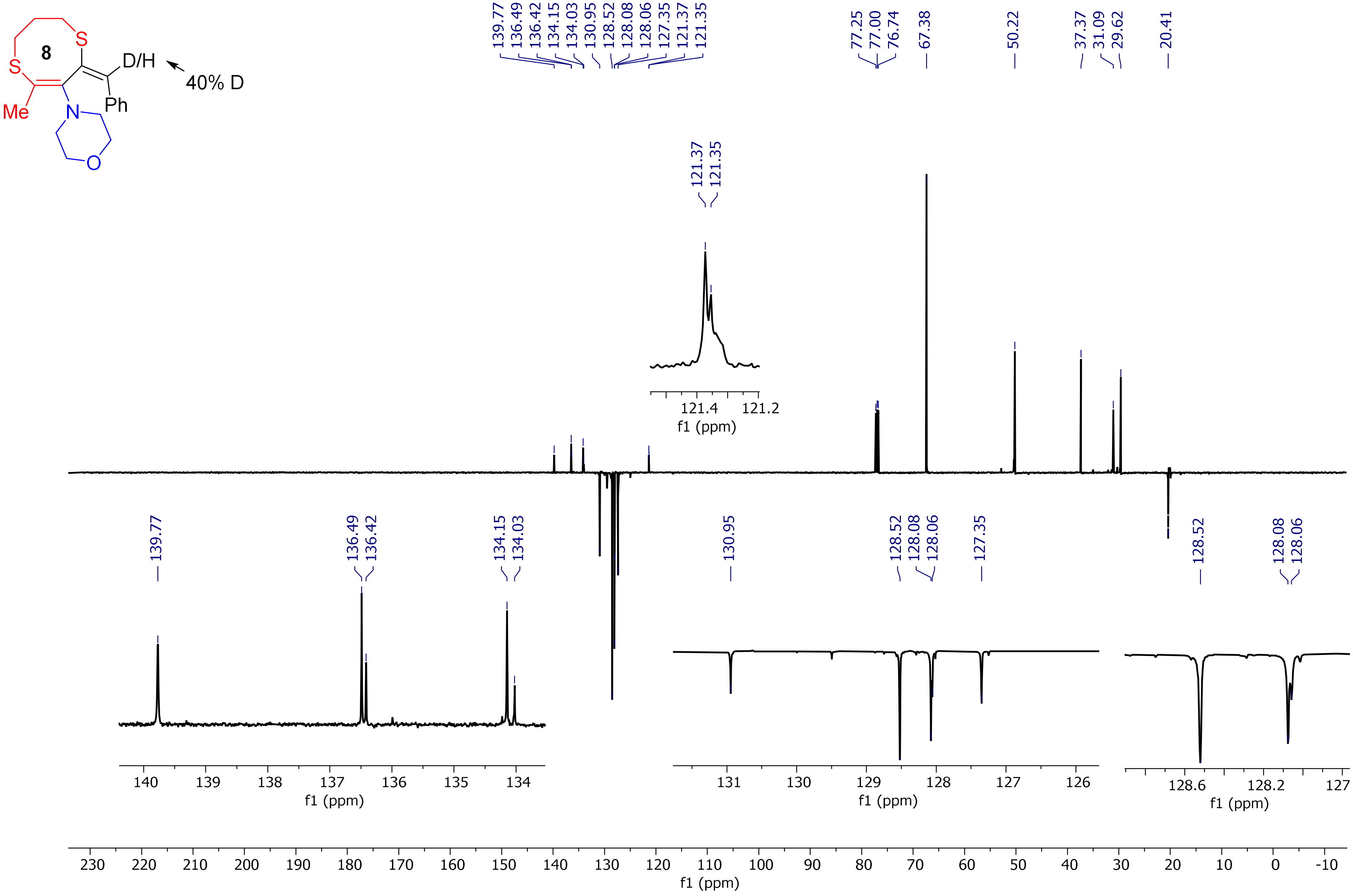

Figure S102. ^{1}H NMR ($CDCl_3$, 500 MHz) spectrum **2y**

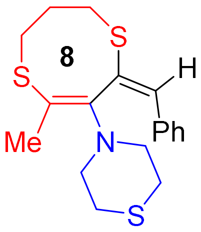

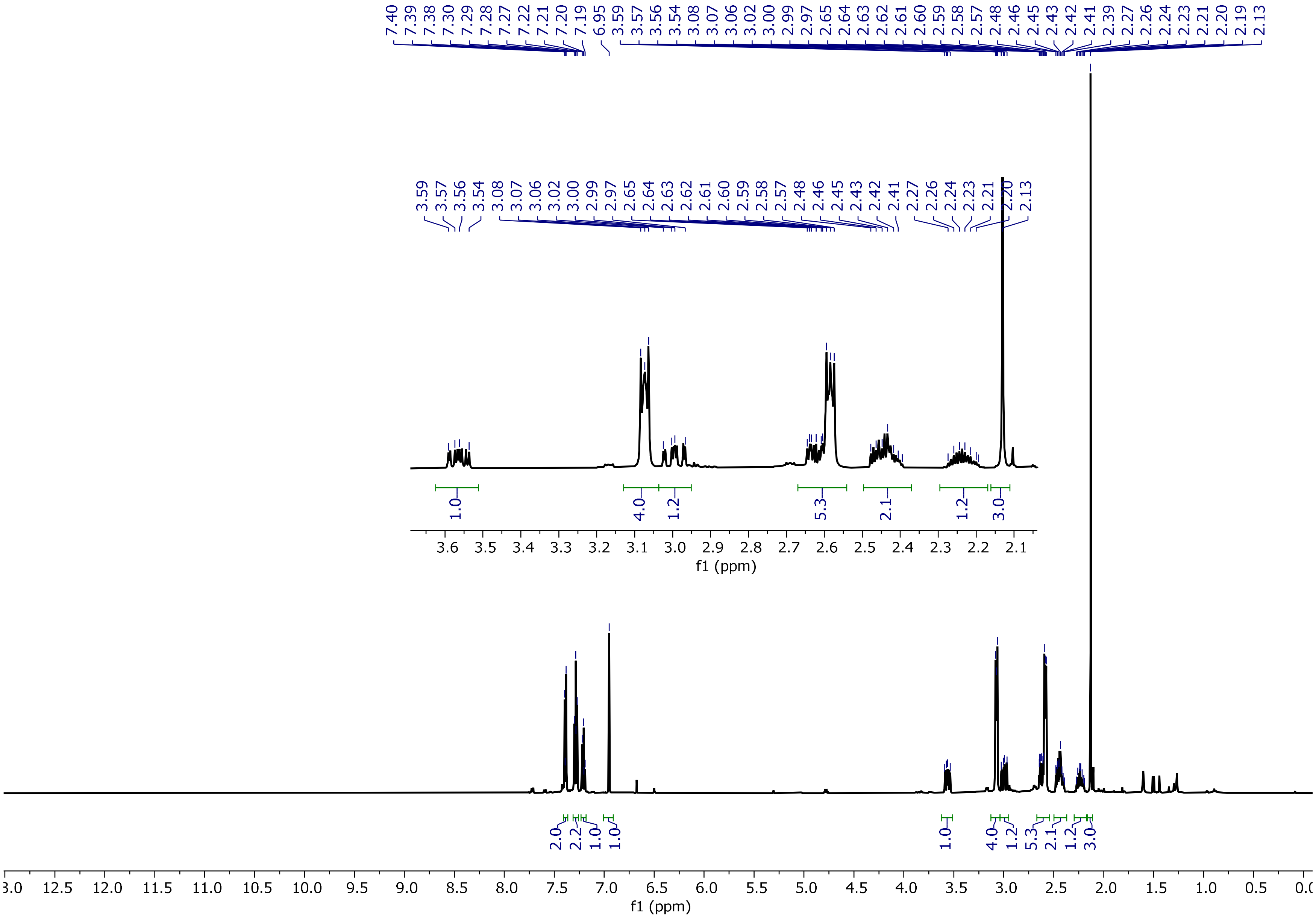

Figure S103. ^{13}C-APT NMR ($CDCl_3$, 125 MHz) spectrum **2y**

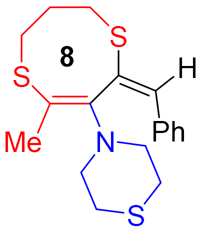

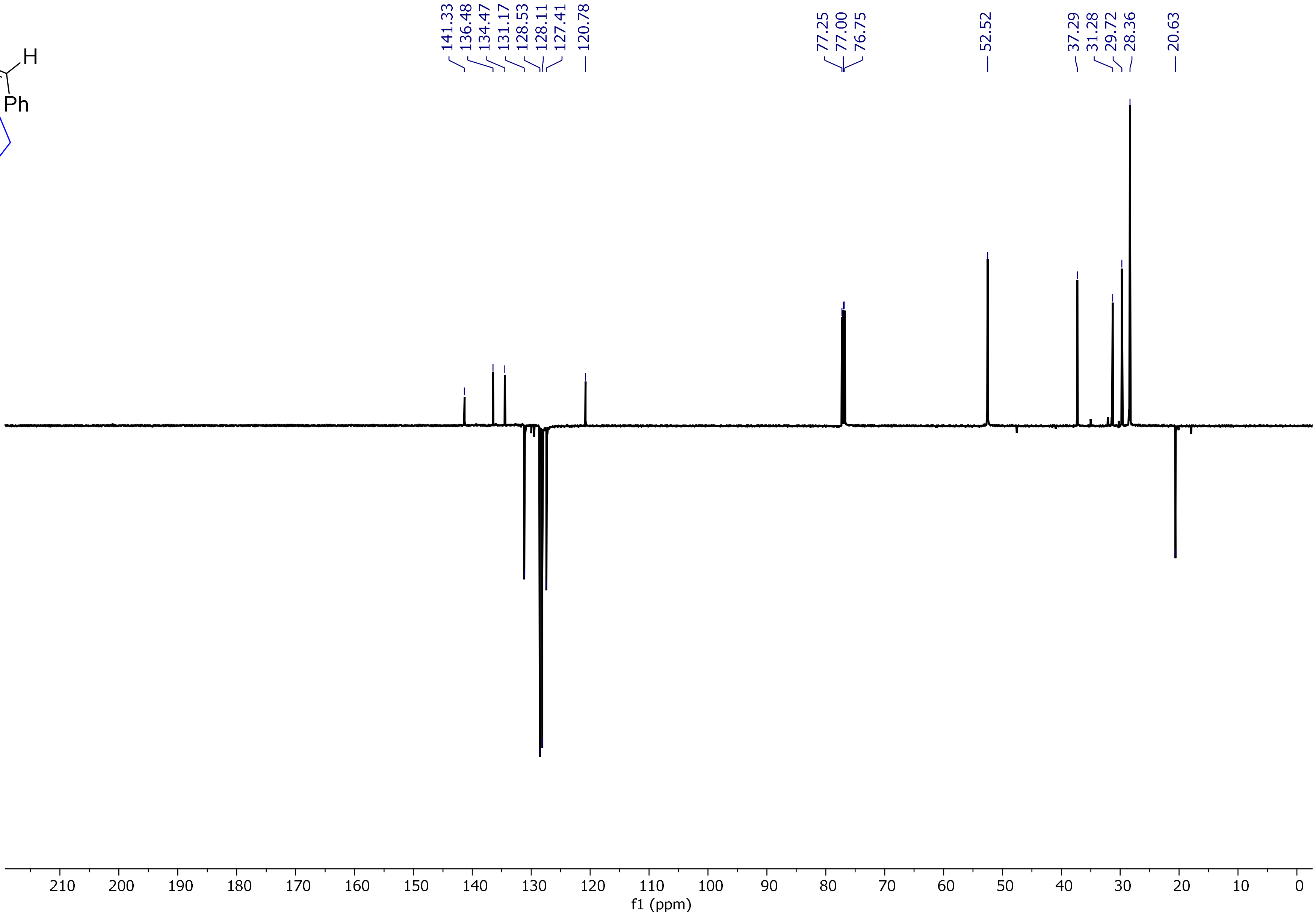

Figure S104. ^{1}H NMR ($CDCl_3$, 500 MHz) spectrum **3a**

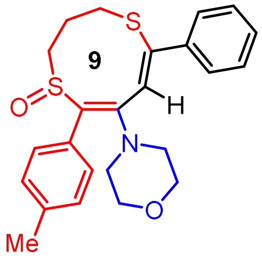

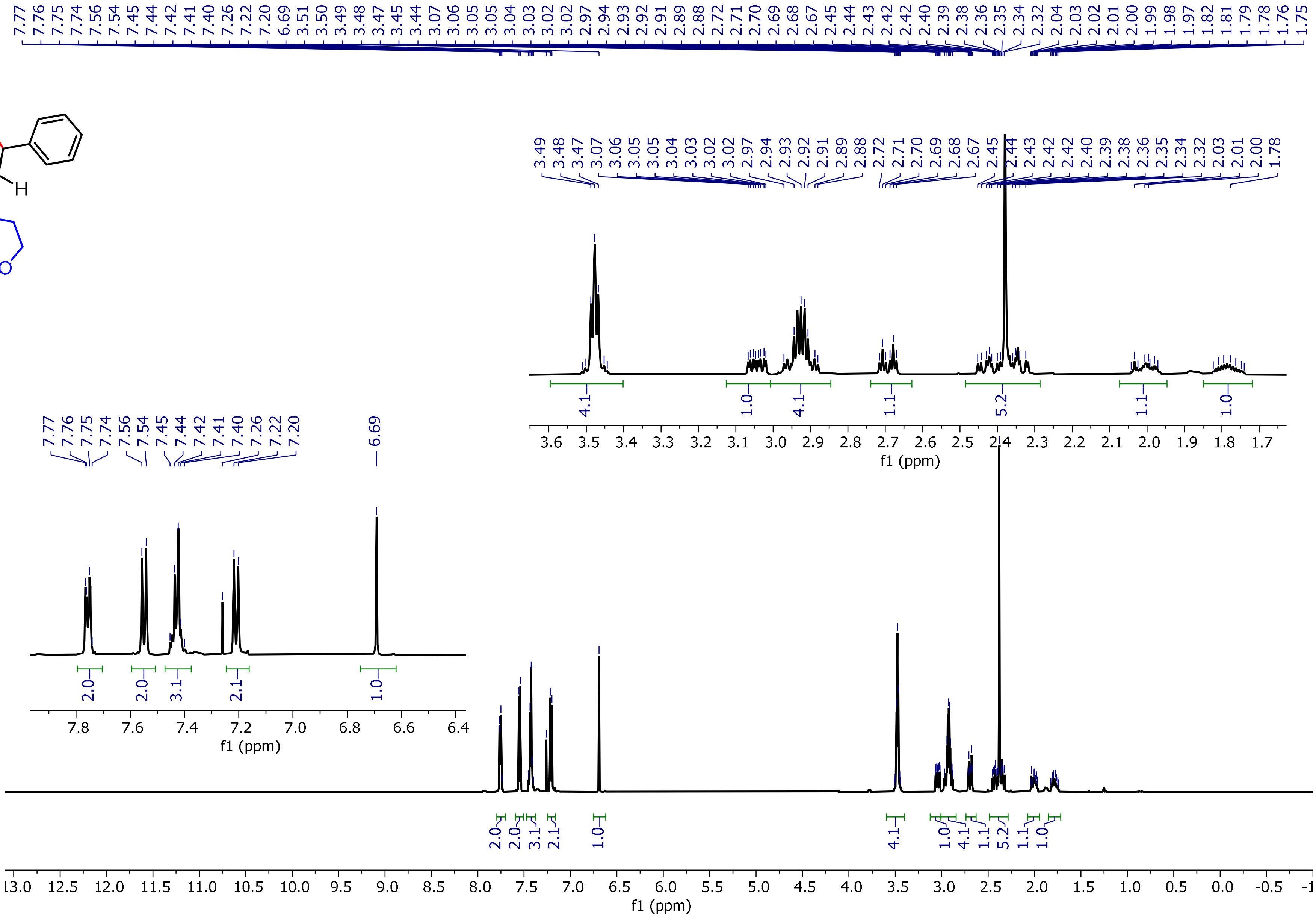

Figure S105. ^{13}C-APT NMR ($CDCl_3$, 125 MHz) spectrum **3a**

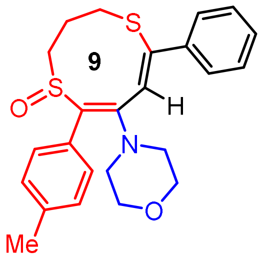

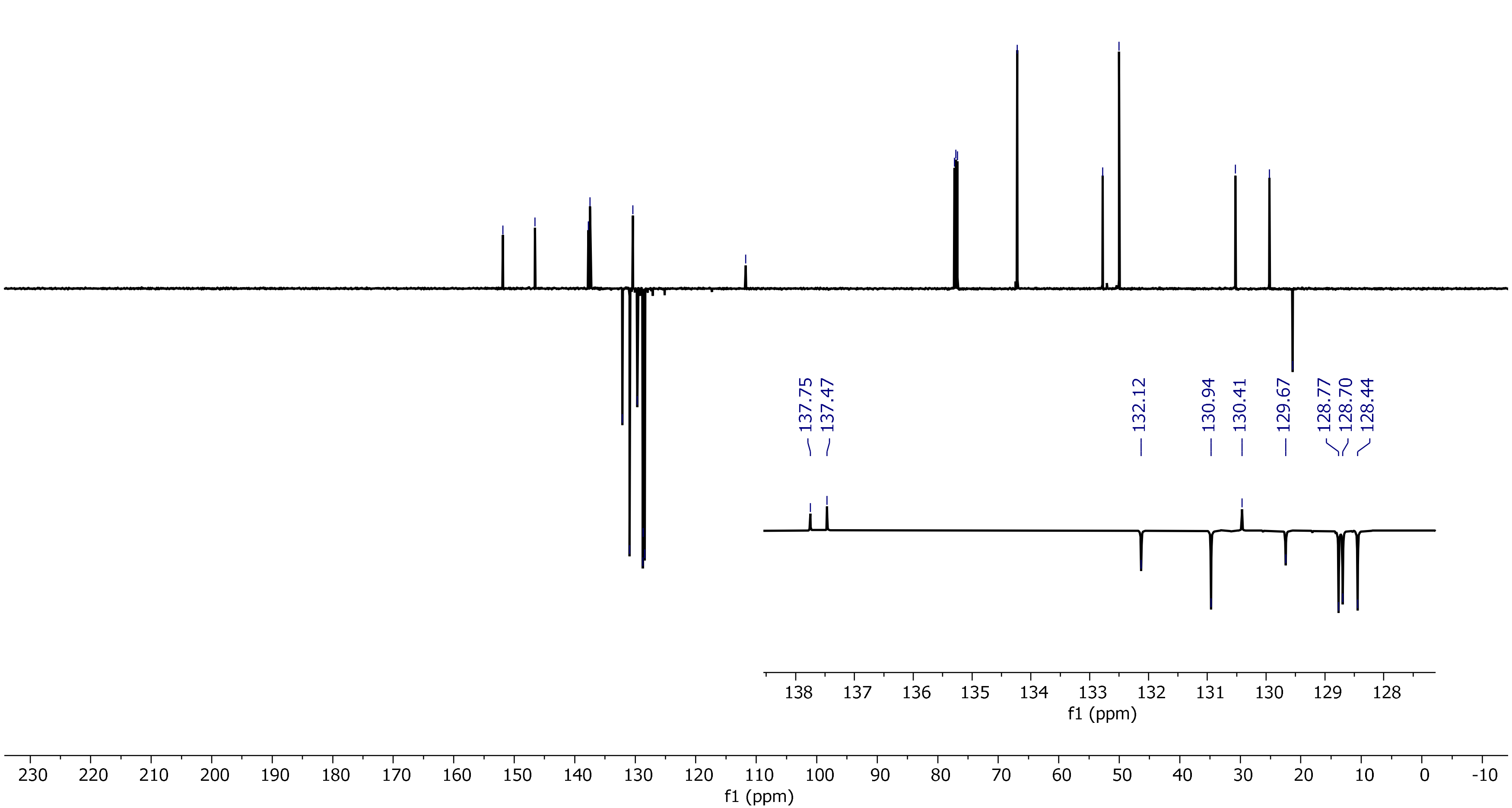

Figure S106. ^{1}H-NMR ($CDCl_3$, 500 MHz) spectrum **5a**

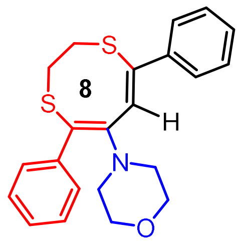

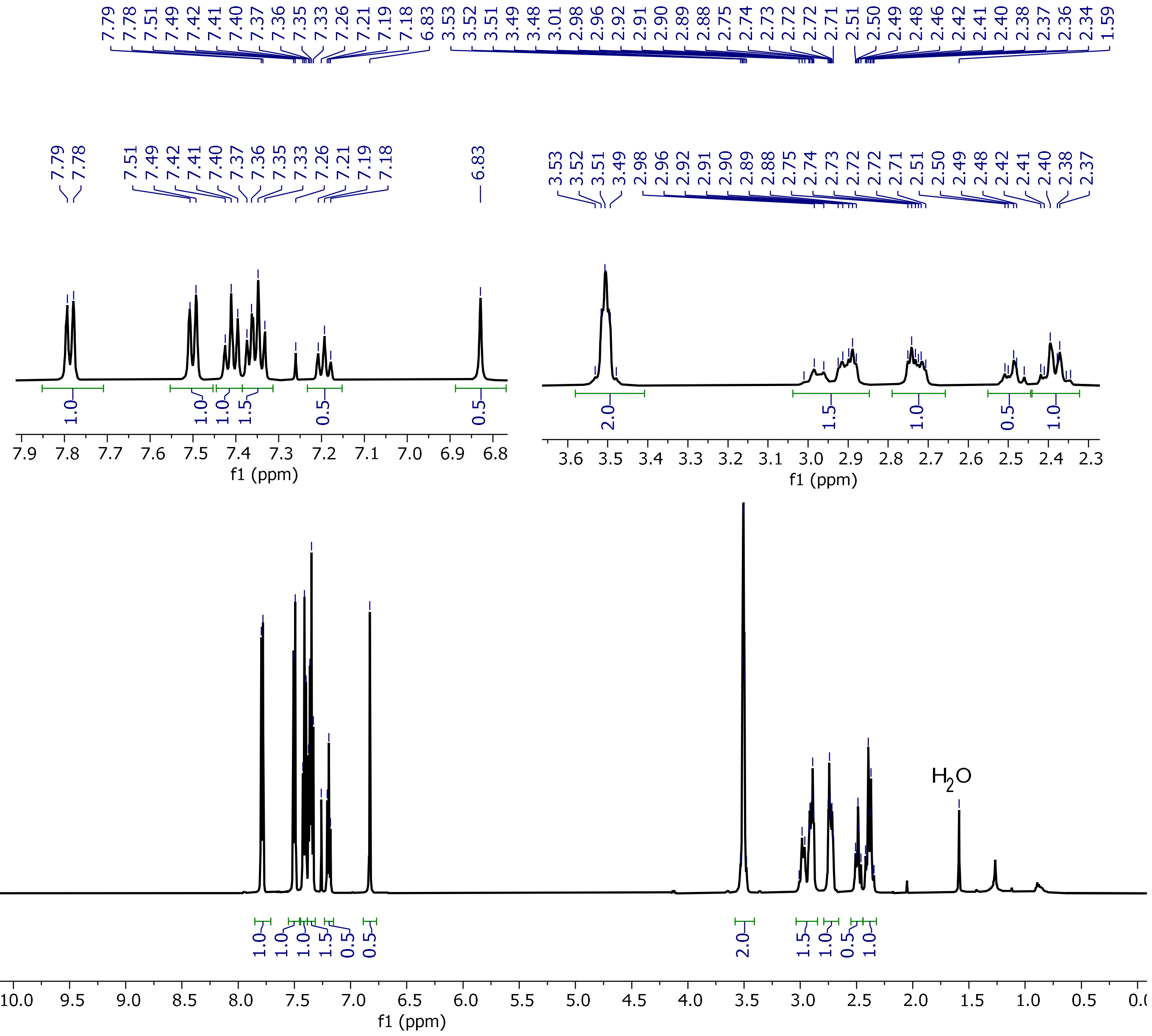

Figure S107. ^{13}C-NMR ($CDCl_3$, 125 MHz) spectrum **5a**

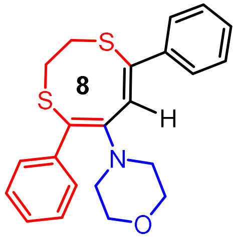

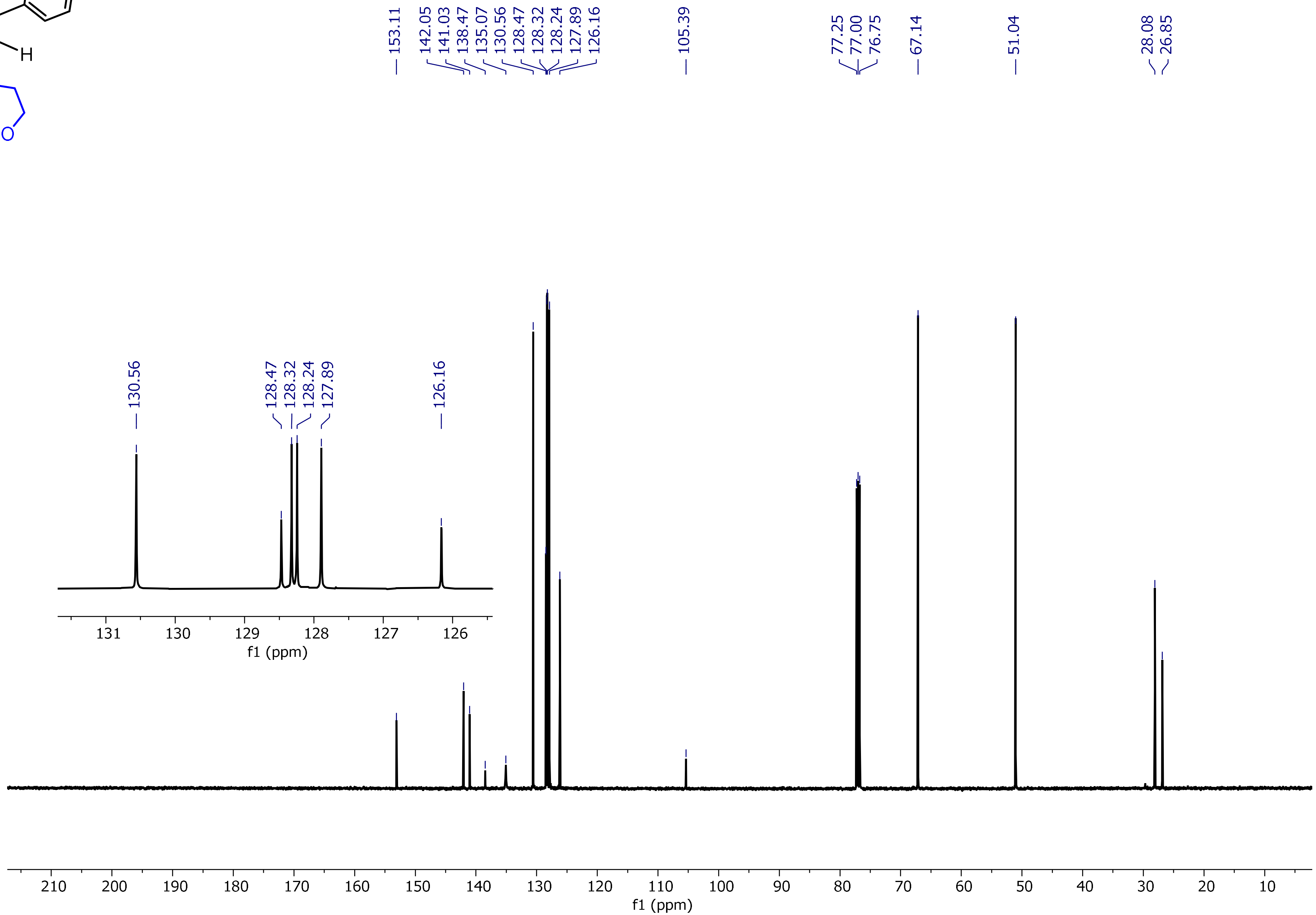

Figure S108. ^{13}C-APT NMR ($CDCl_3$, 125 MHz) spectrum **5a**

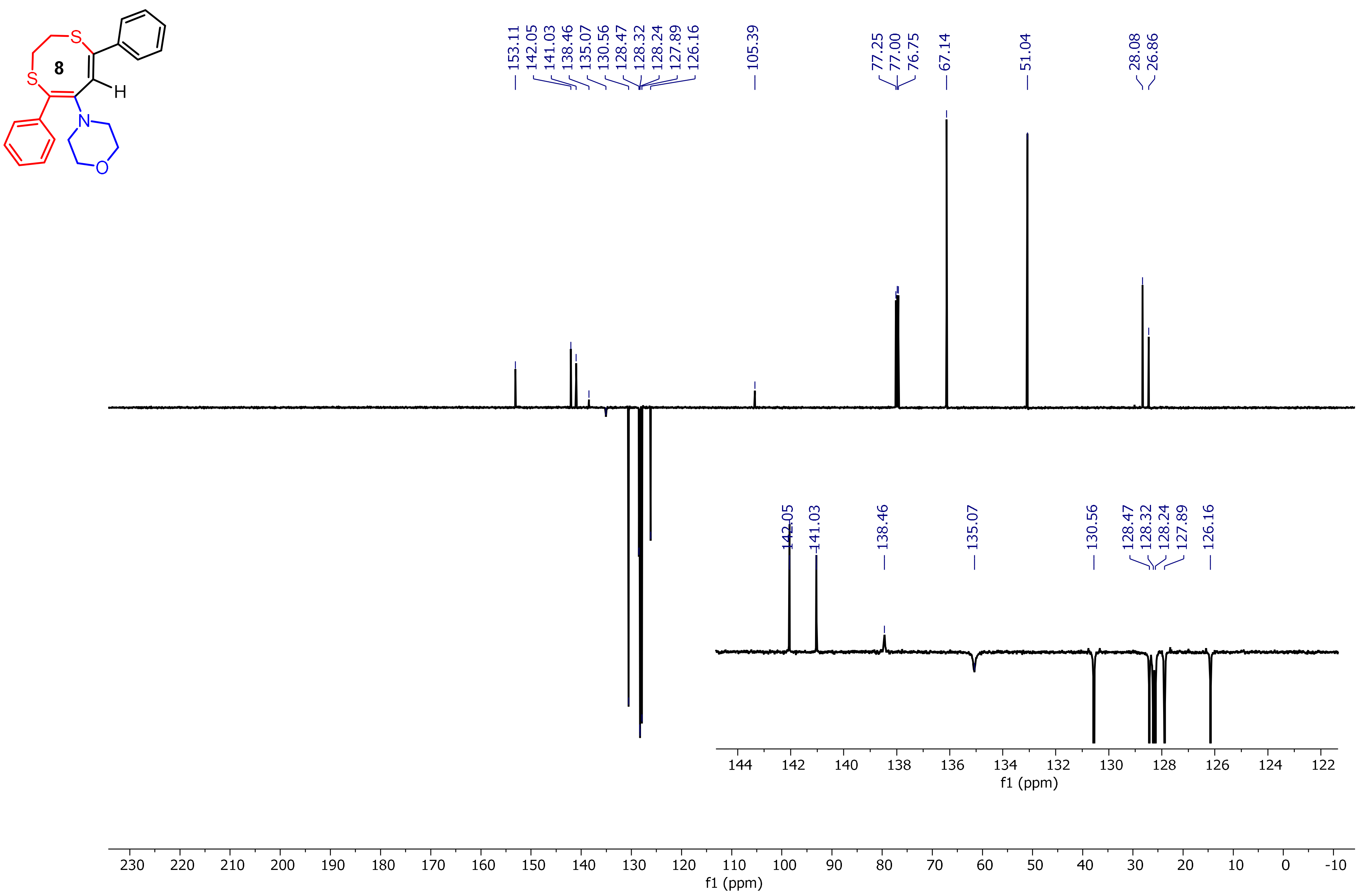

Figure S109. ^{1}H-NMR ($CDCl_3$, 500 MHz) spectrum **5b**

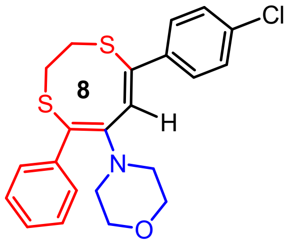

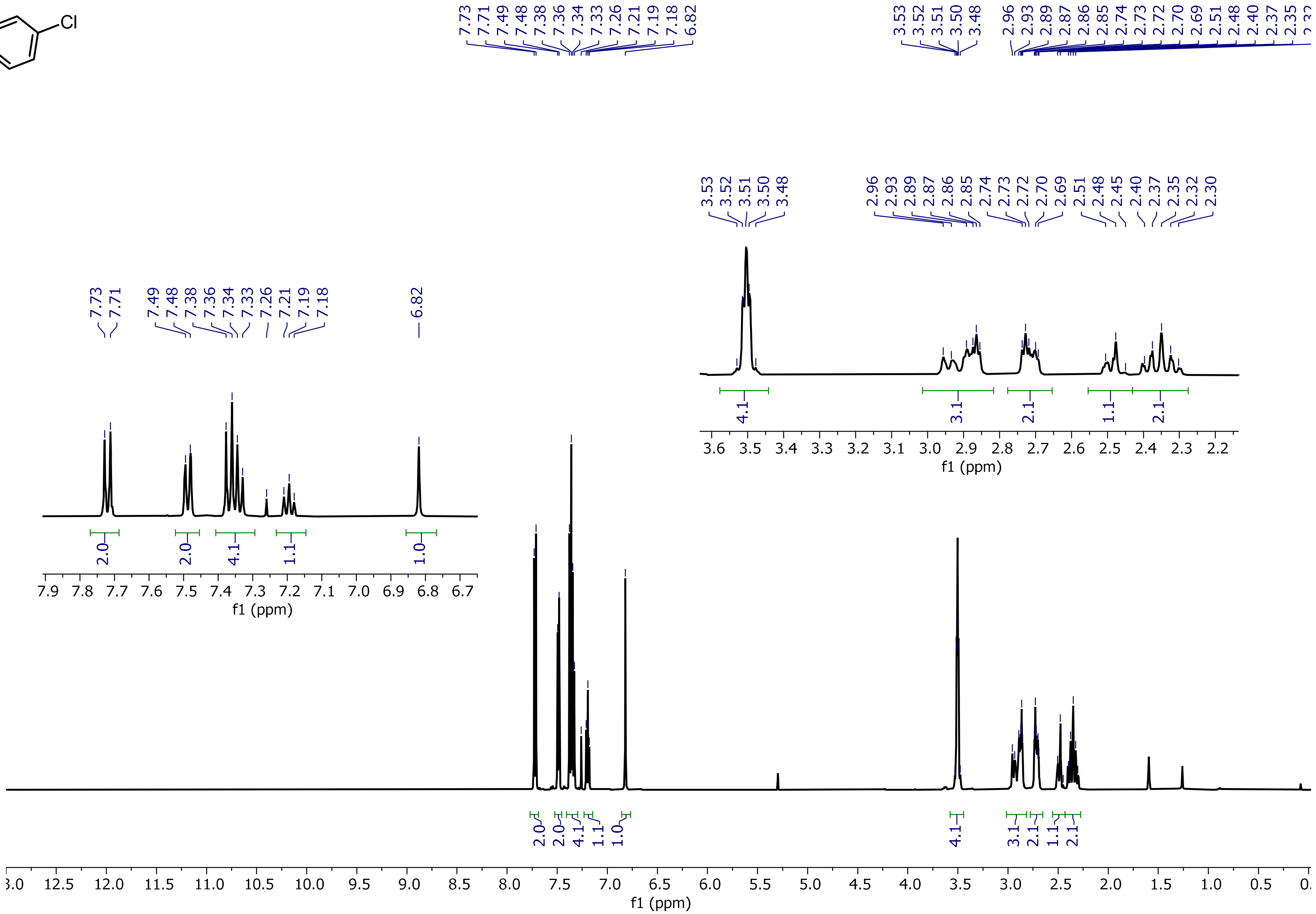

Figure S110. ^{13}C-NMR ($CDCl_3$, 125 MHz) spectrum **5b**

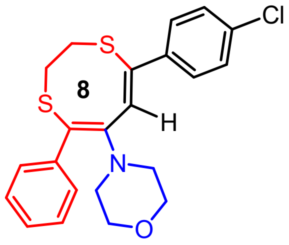

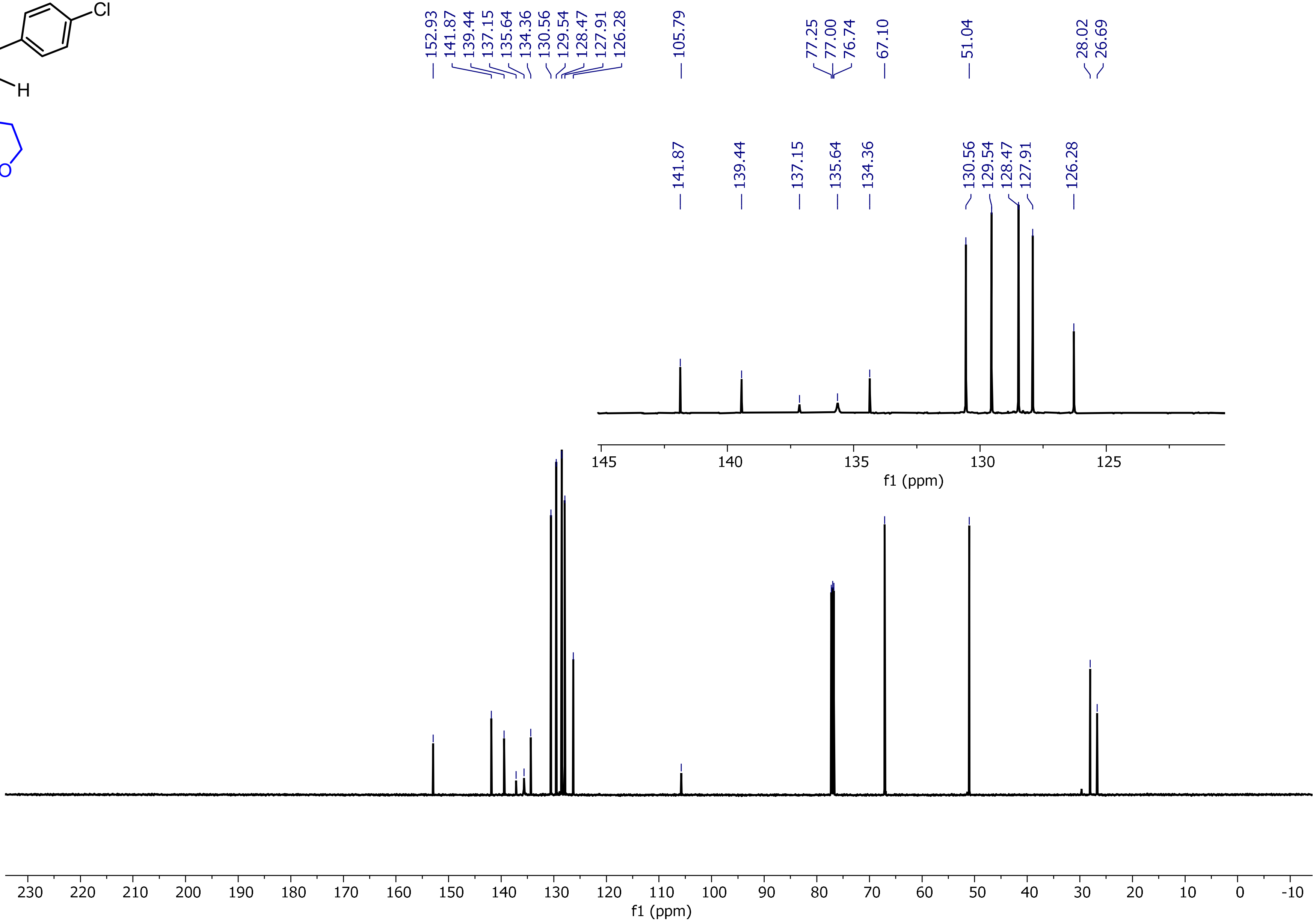

Figure S111. ^{13}C-APT NMR ($CDCl_3$, 125 MHz) spectrum **5b**

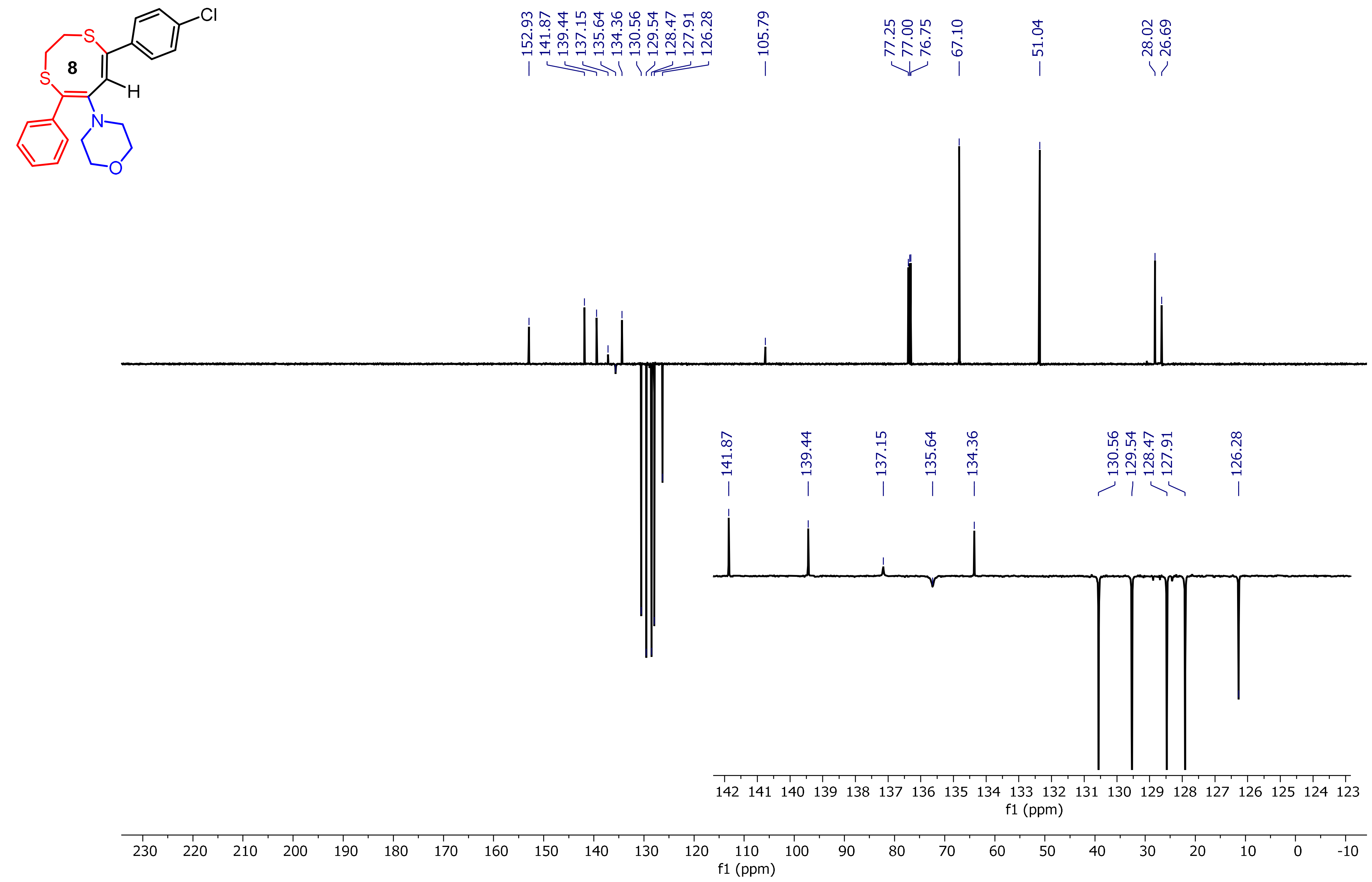

Figure S112. ^{1}H-NMR ($CDCl_3$, 500 MHz) spectrum **5c**

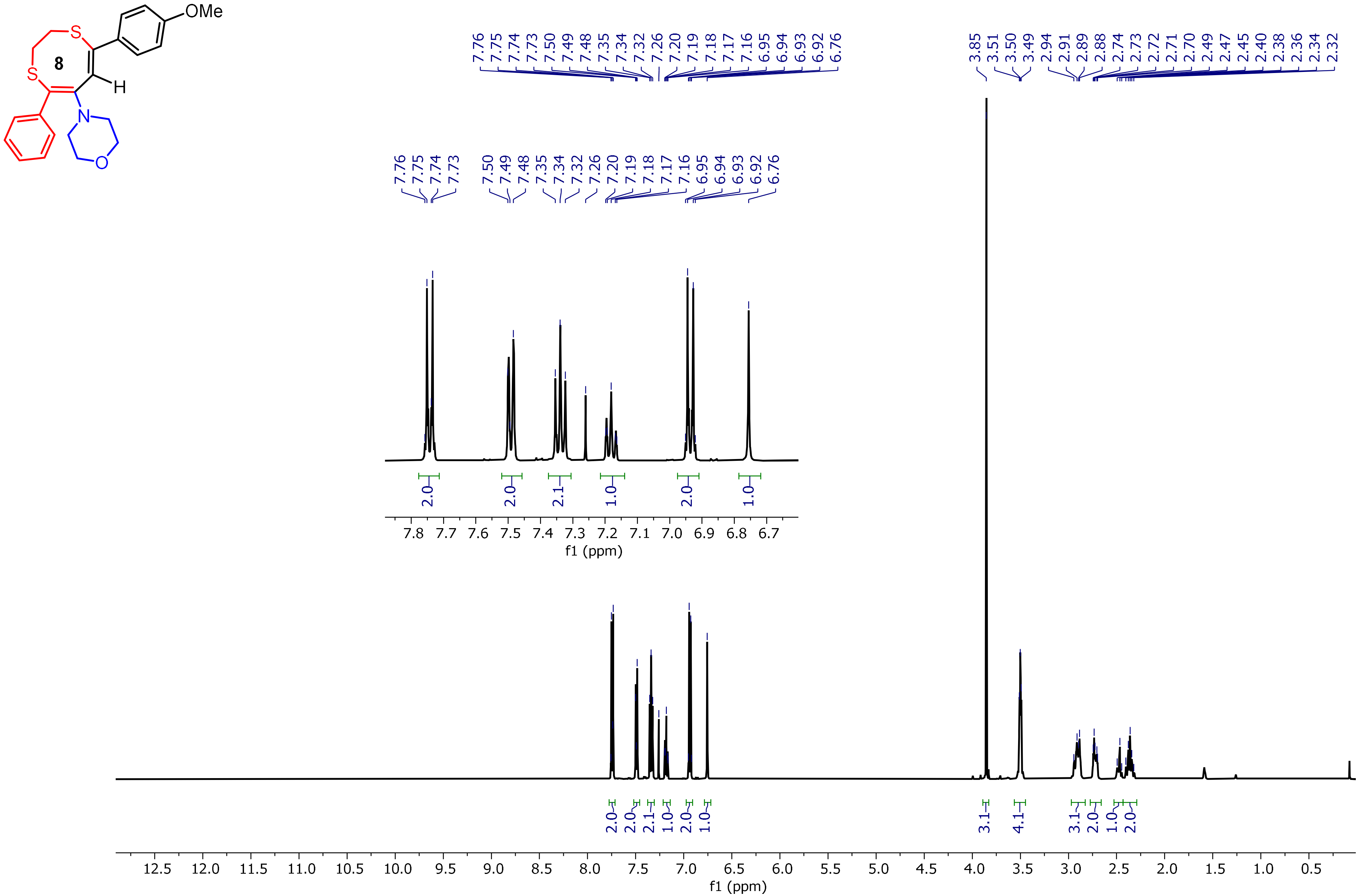

Figure S113. ^{13}C-NMR ($CDCl_3$, 125 MHz) spectrum **5c**

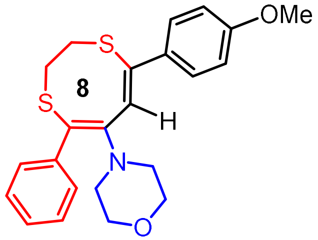

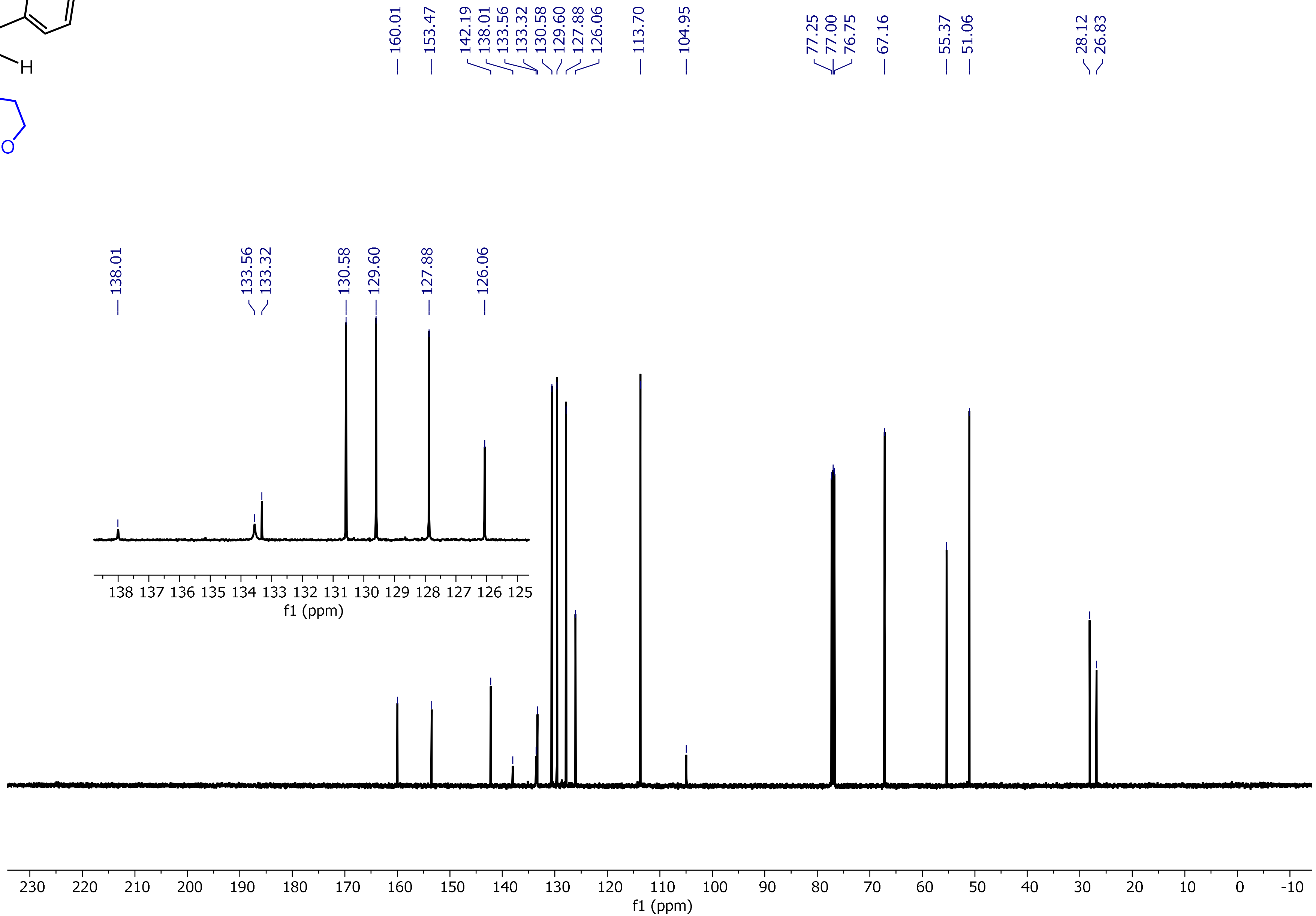

Figure S114. ^{13}C-APT NMR ($CDCl_3$, 125 MHz) spectrum **5c**

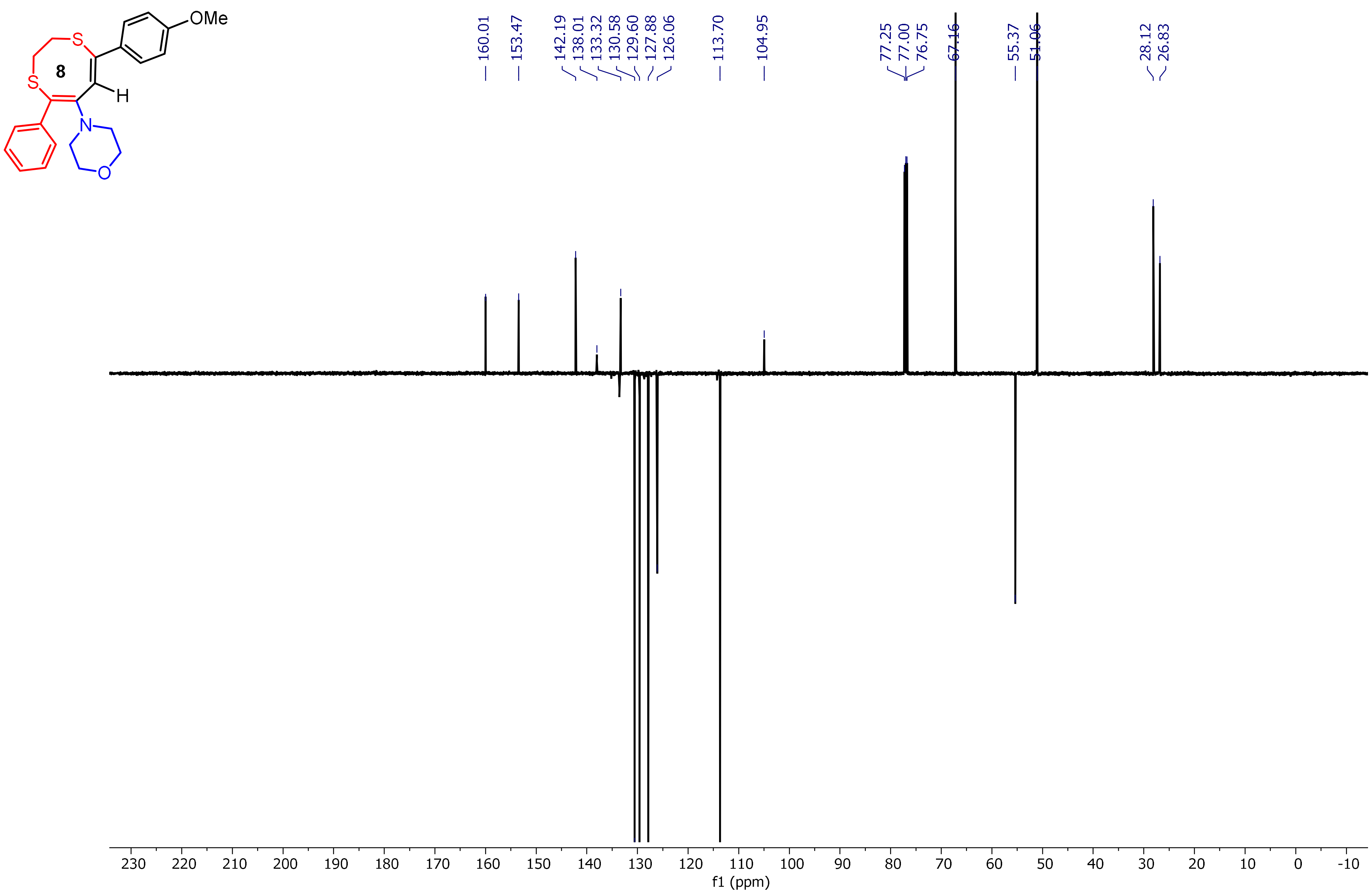

Figure S115. ^{1}H-NMR ($CDCl_3$, 500 MHz) spectrum **5d**

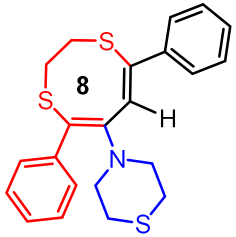

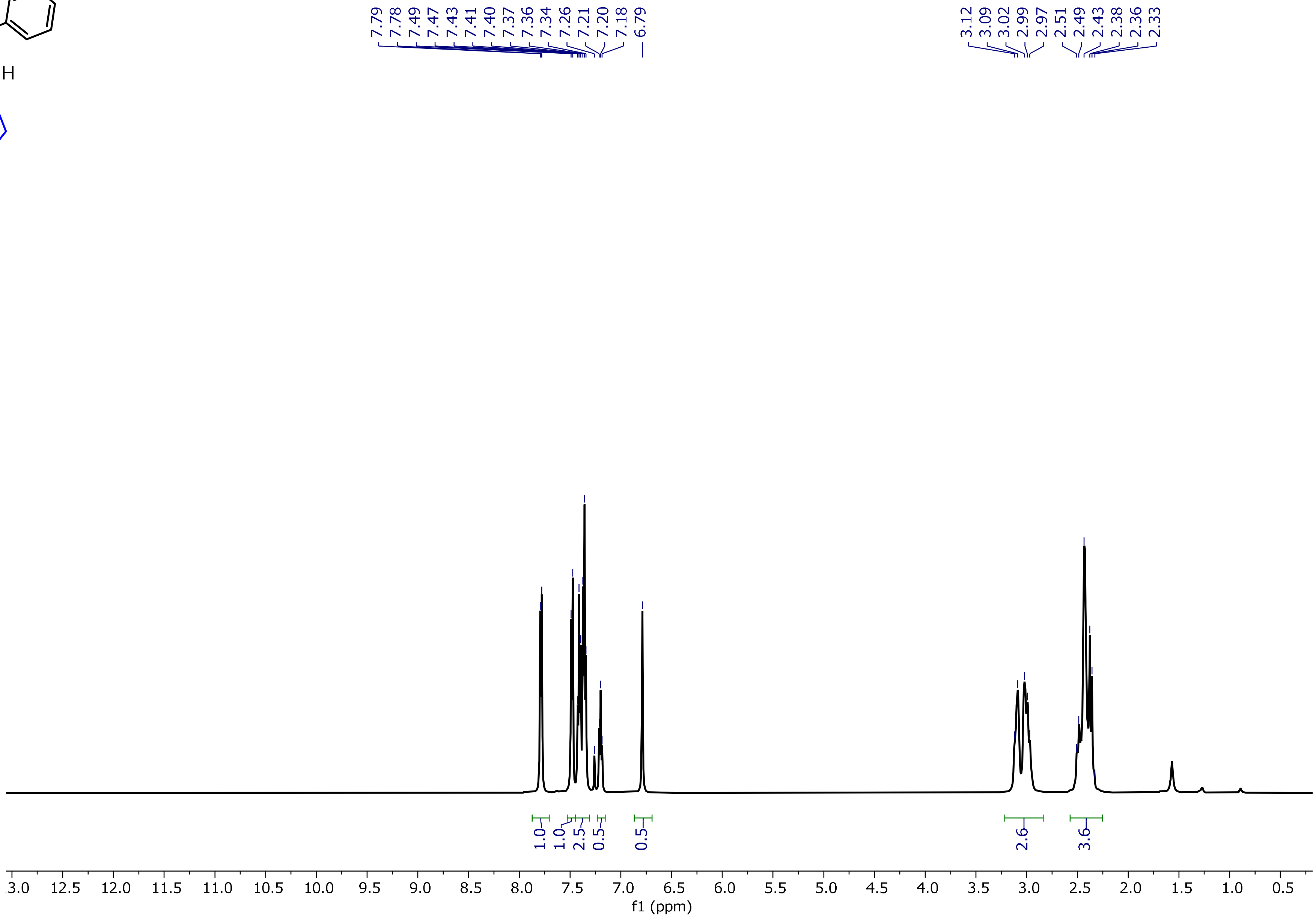

Figure S116. ^{13}C-NMR ($CDCl_3$, 125 MHz) spectrum **5d**

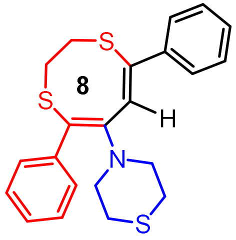

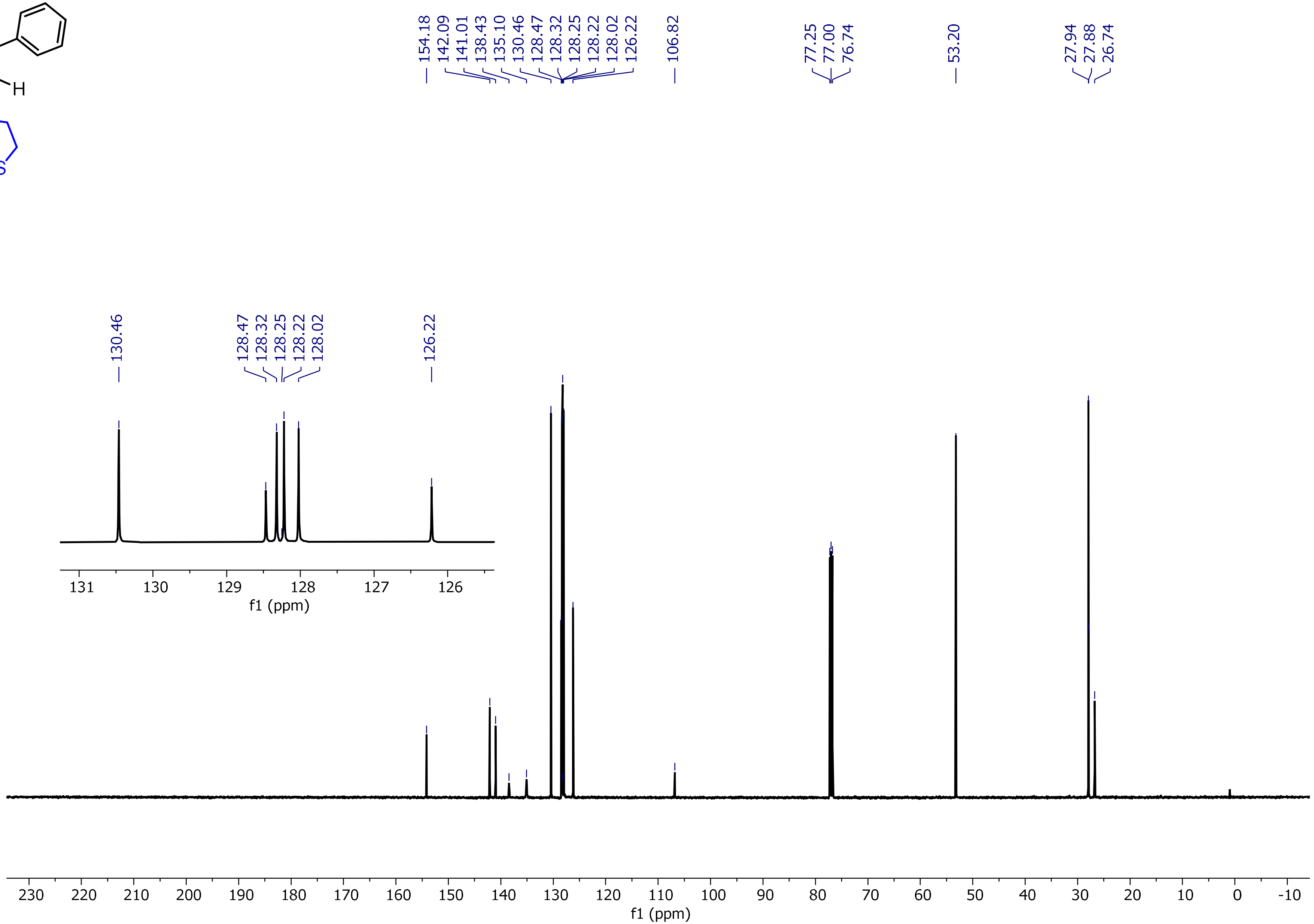

Figure S117. ^{13}C-APT NMR ($CDCl_3$, 125 MHz) spectrum **5d**

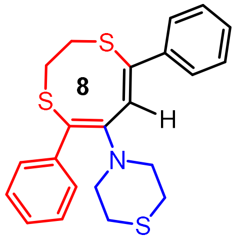

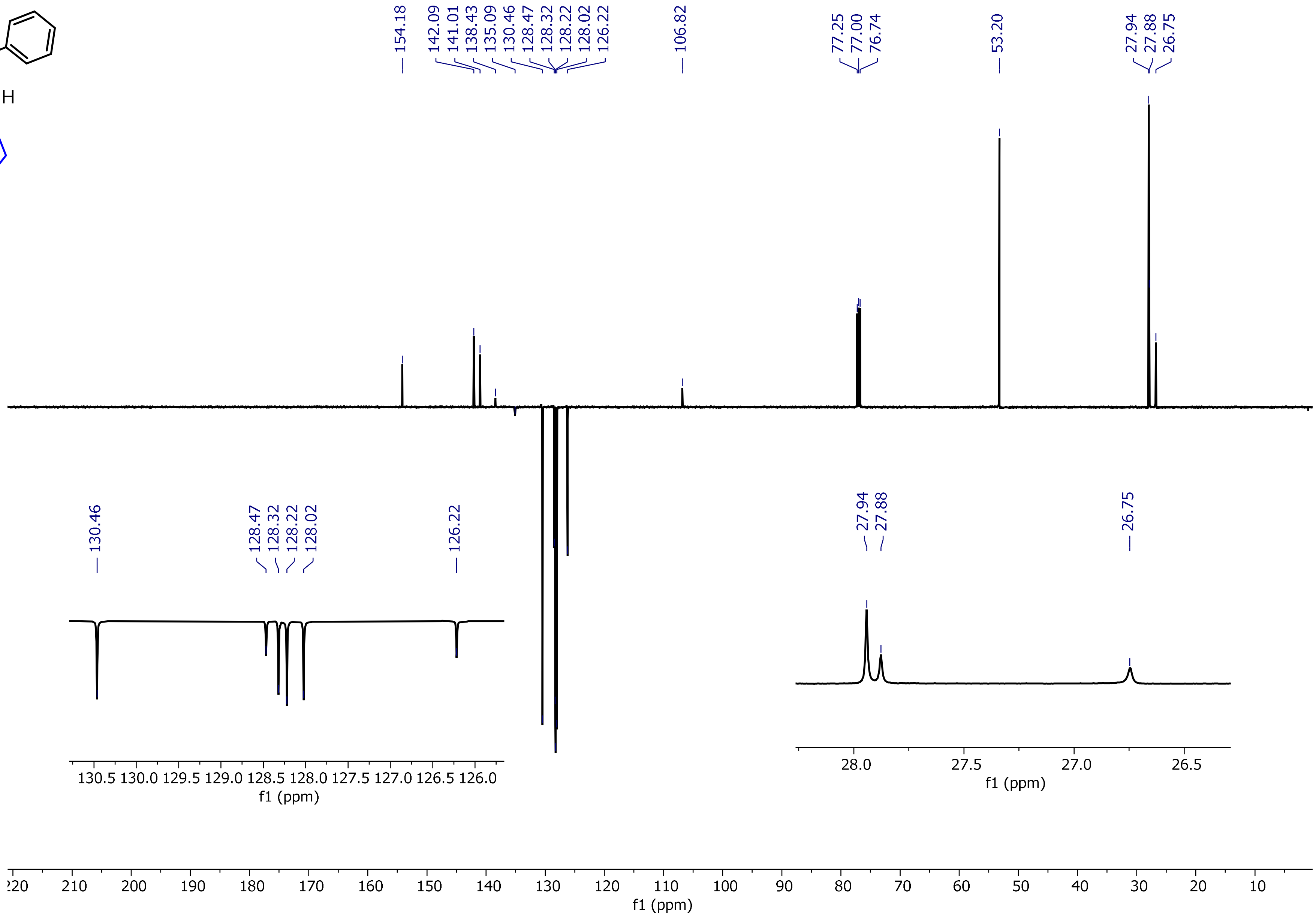

Figure S118. ^{1}H-NMR ($CDCl_3$, 500 MHz) spectrum **5e**

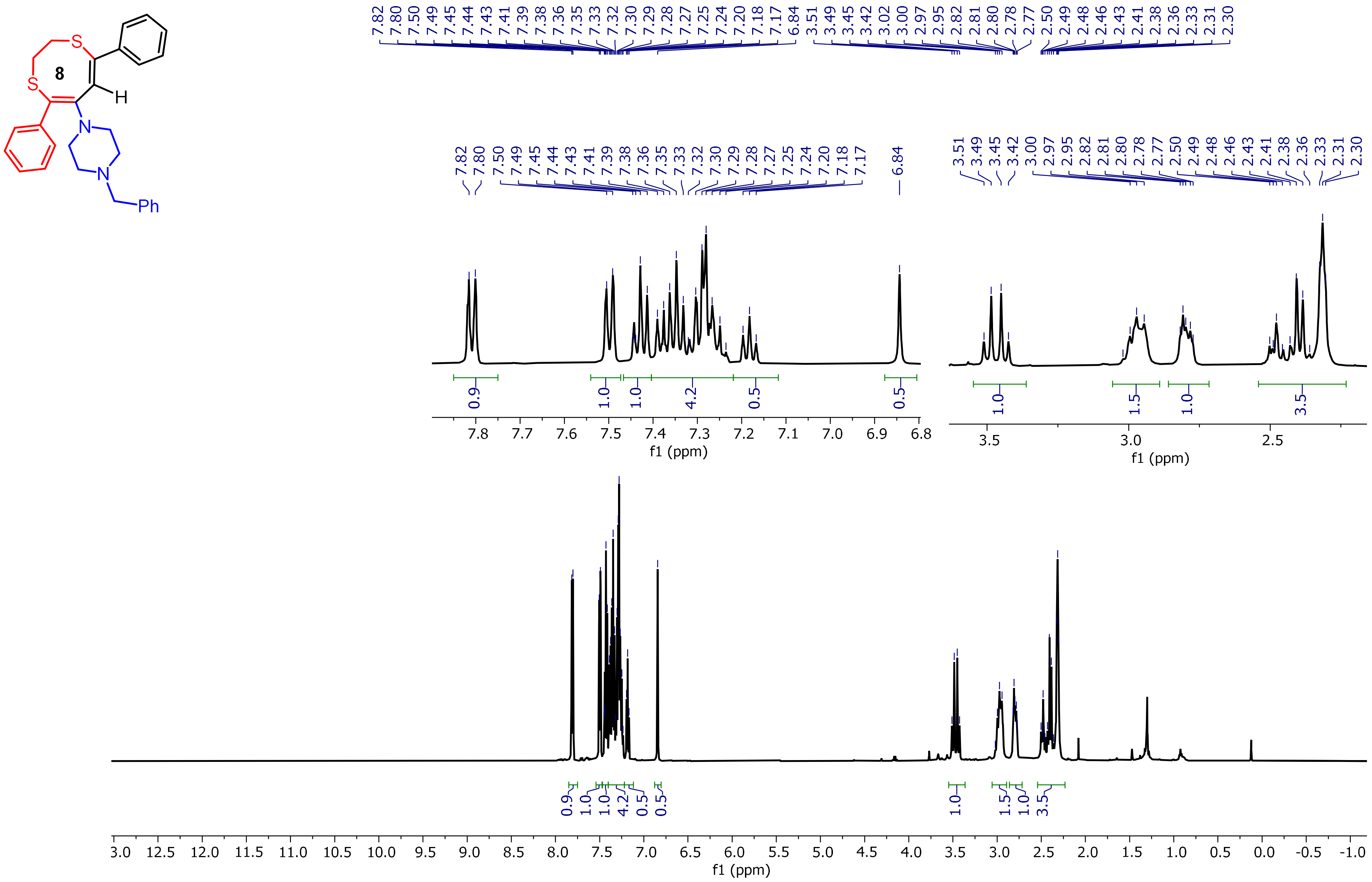

Figure S119. ^{13}C-NMR ($CDCl_3$, 125 MHz) spectrum **5e**

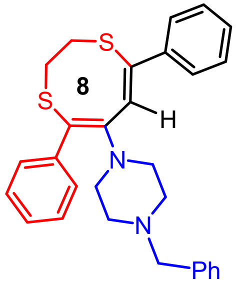

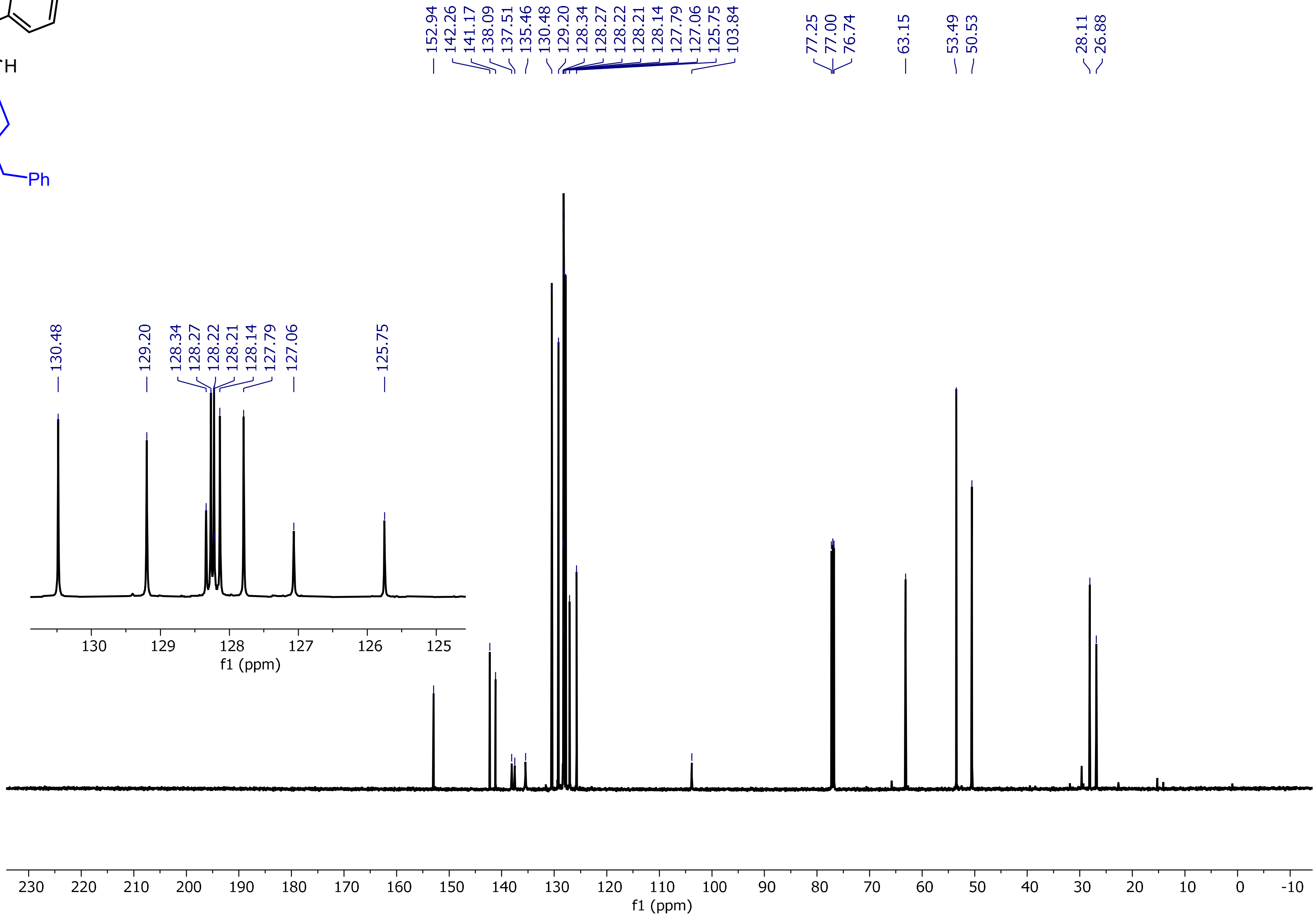

Figure S120. ^{13}C-APT NMR ($CDCl_3$, 125 MHz) spectrum **5e**

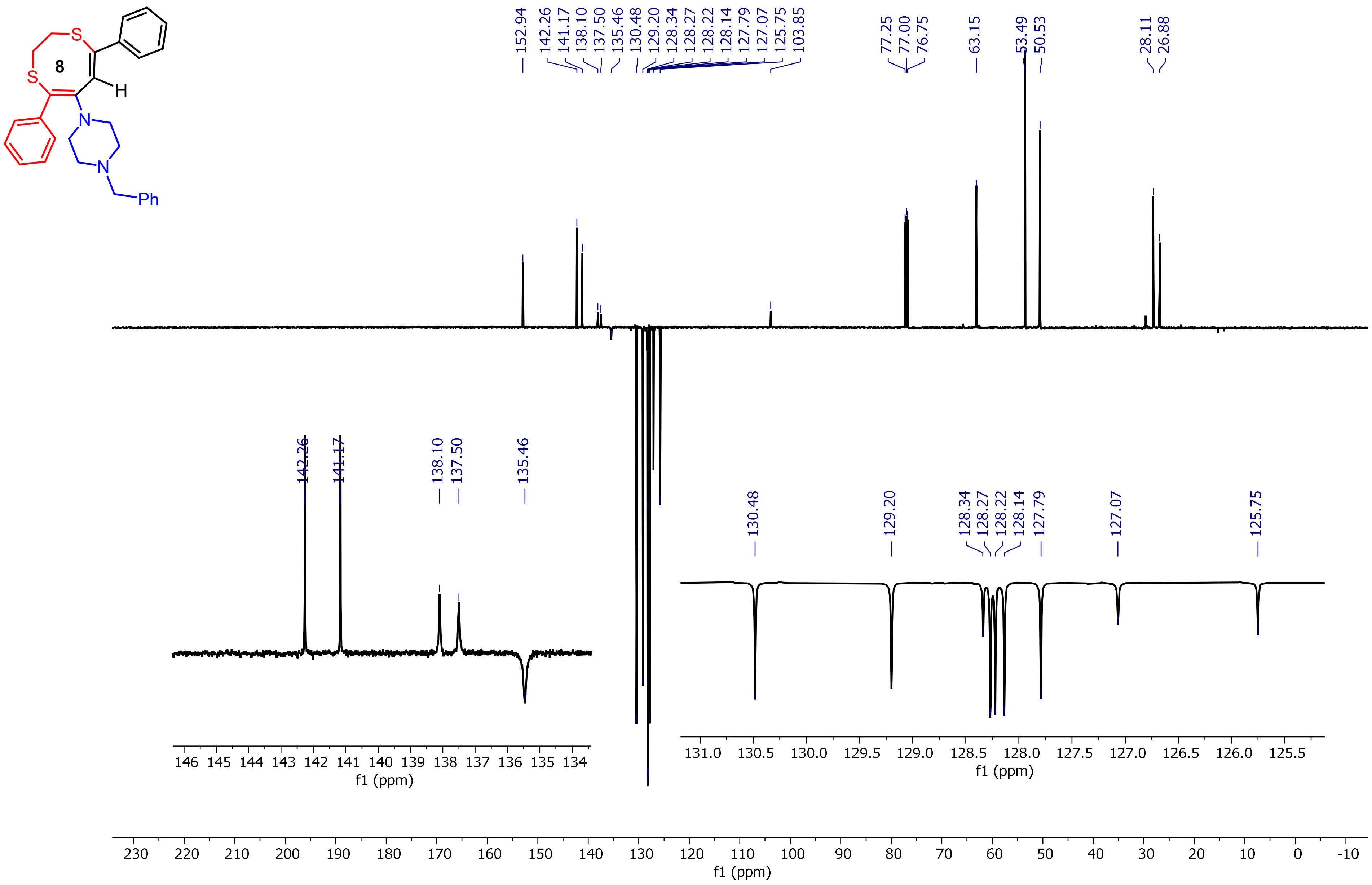

Figure S121. ^{1}H-NMR ($CDCl_3$, 500 MHz) spectrum **7a**

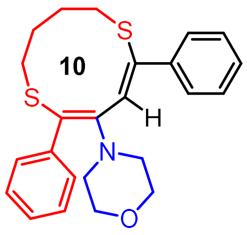

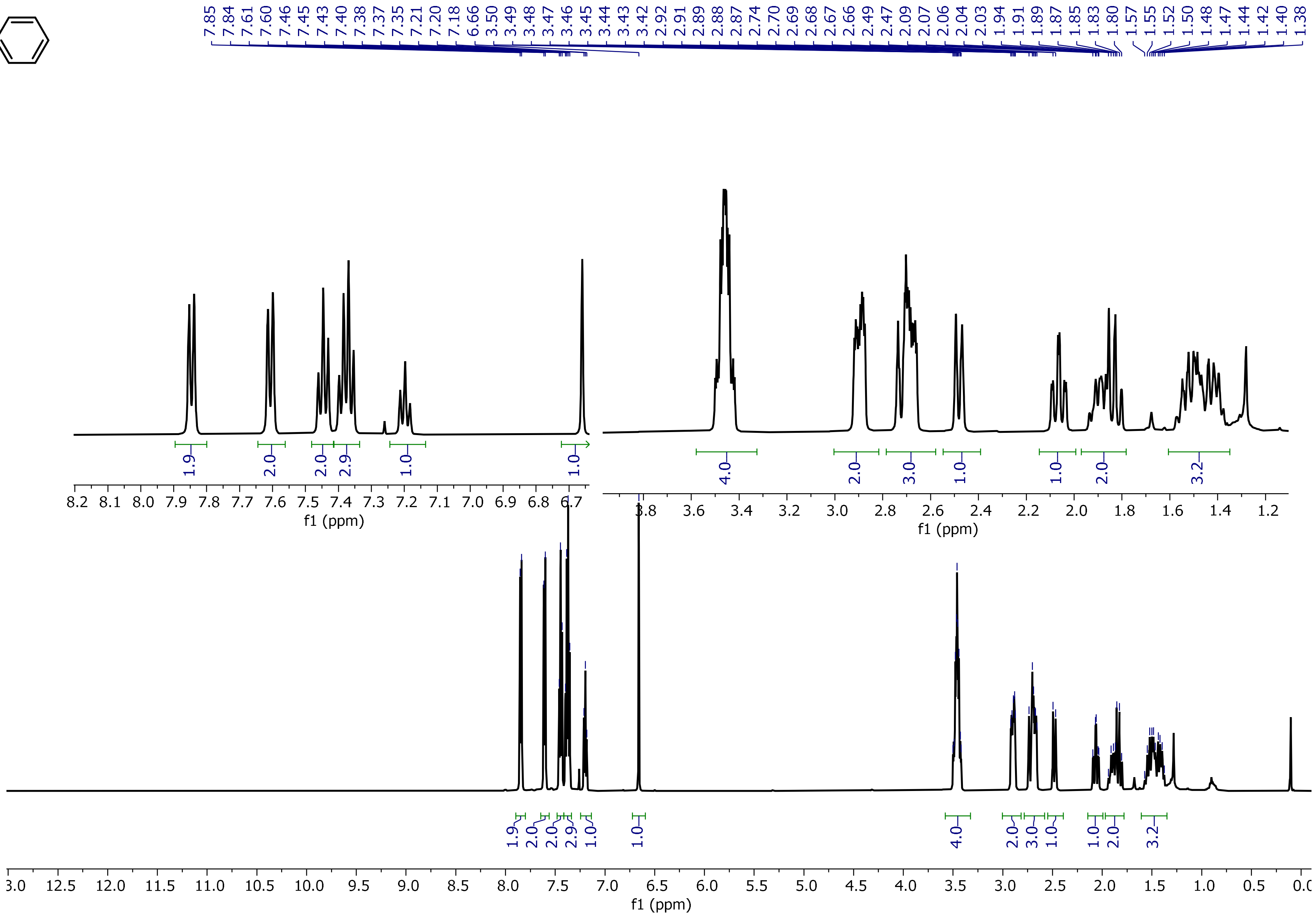

Figure S122. ^{13}C-APT NMR ($CDCl_3$, 125 MHz) spectrum **7a**

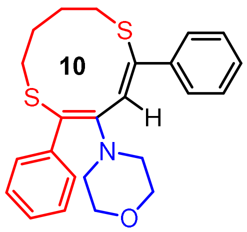

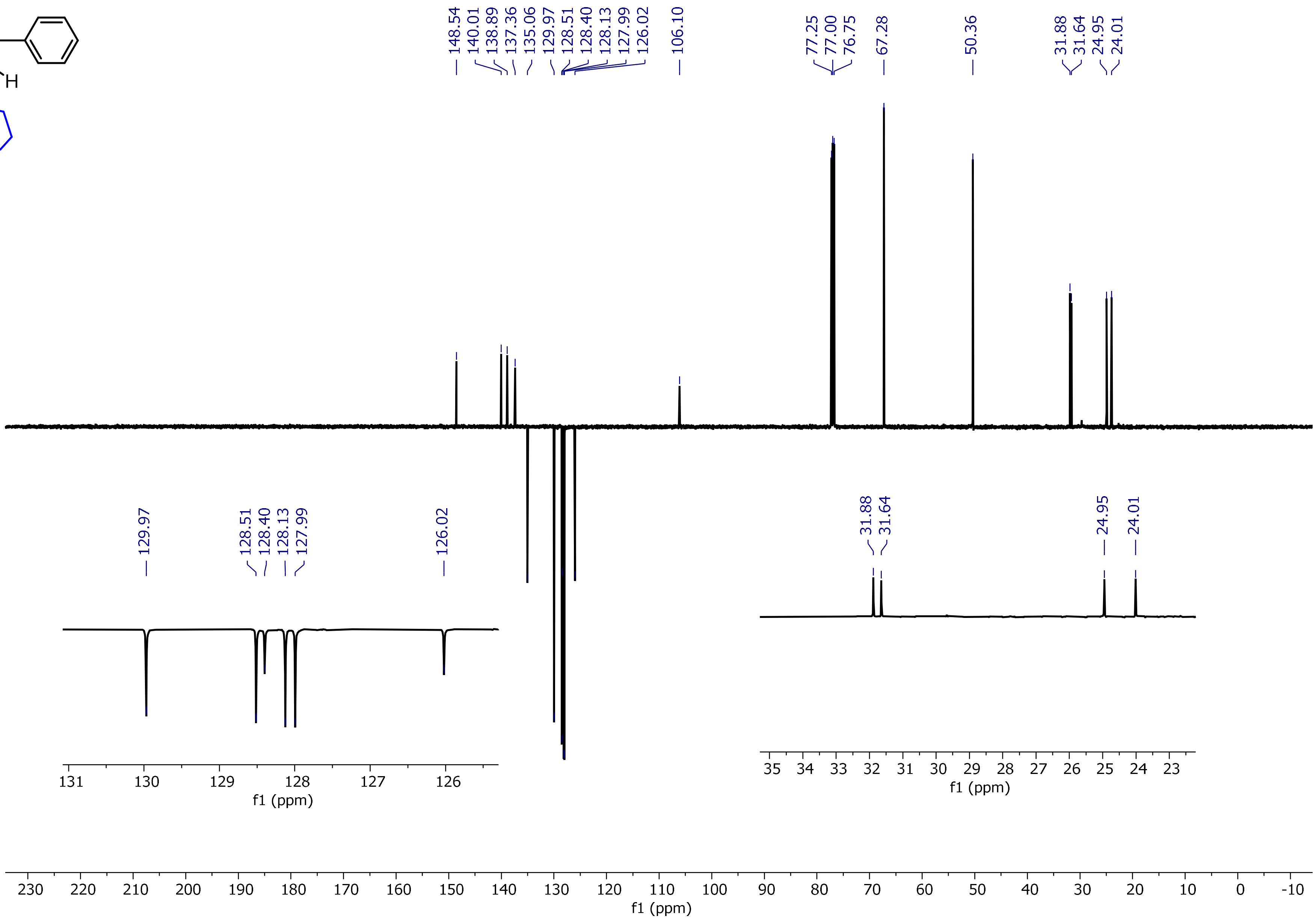

Figure S123. ^{1}H-NMR ($CDCl_3$, 500 MHz) spectrum **7b**

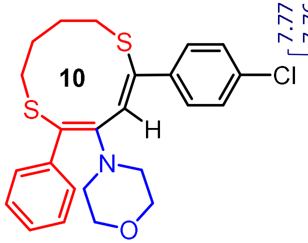

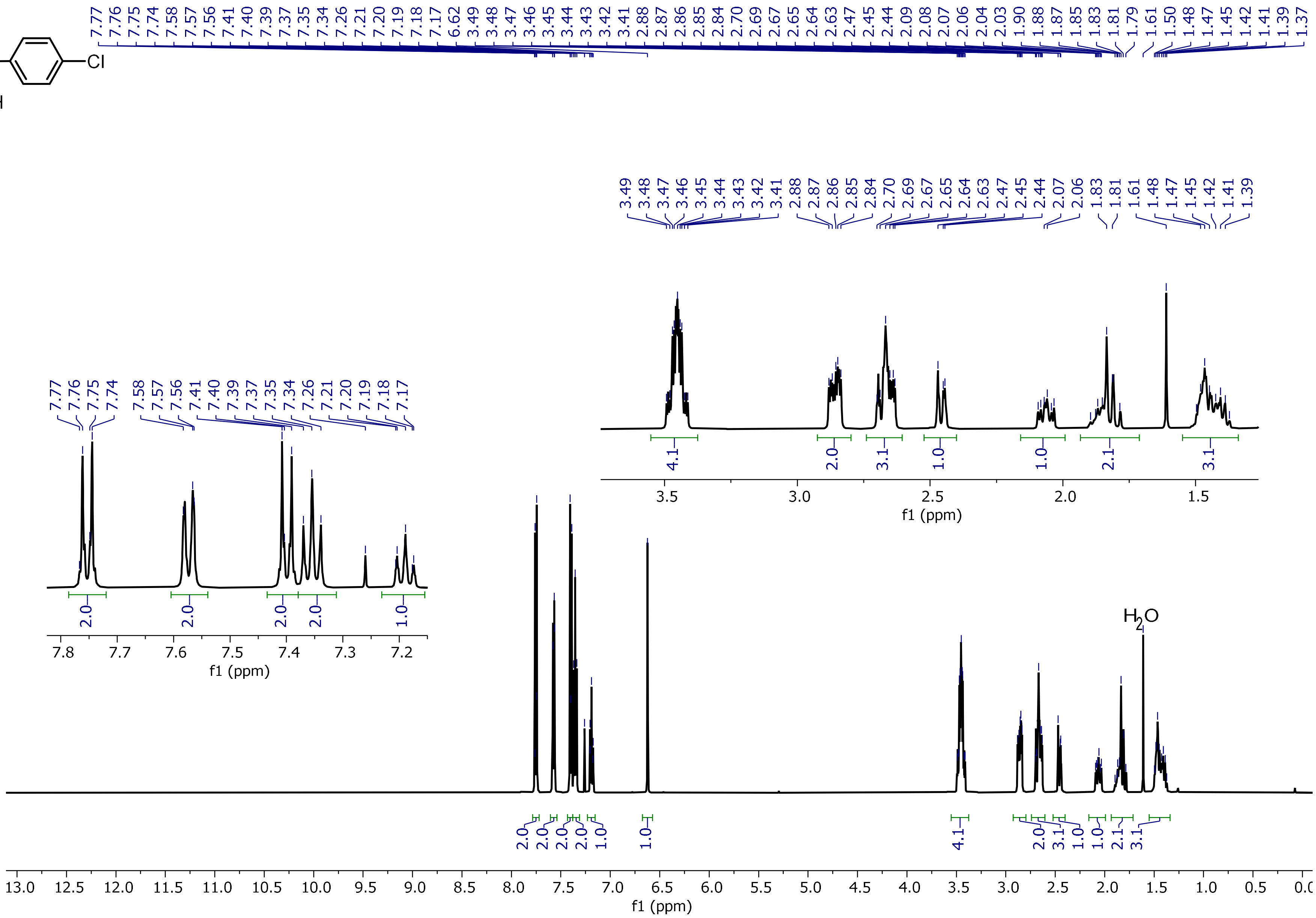

Figure S124. ^{13}C-APT NMR ($CDCl_3$, 125 MHz) spectrum **7b**

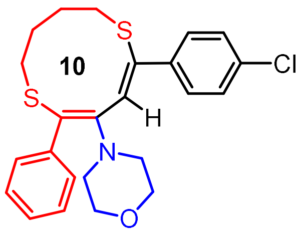

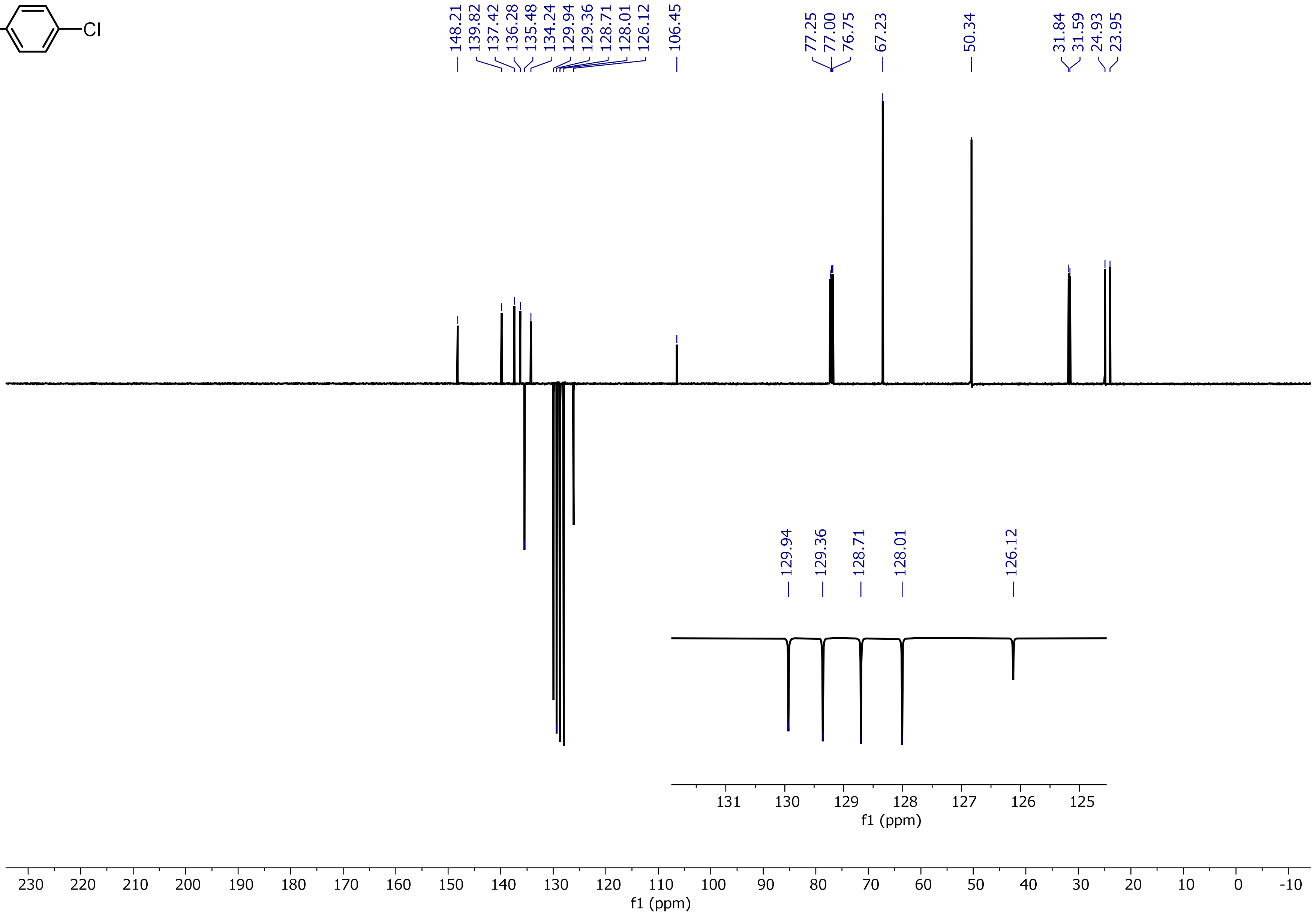

Figure S125. ^{1}H-NMR ($CDCl_3$, 500 MHz) spectrum **7c**

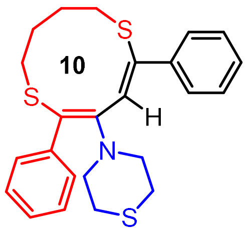

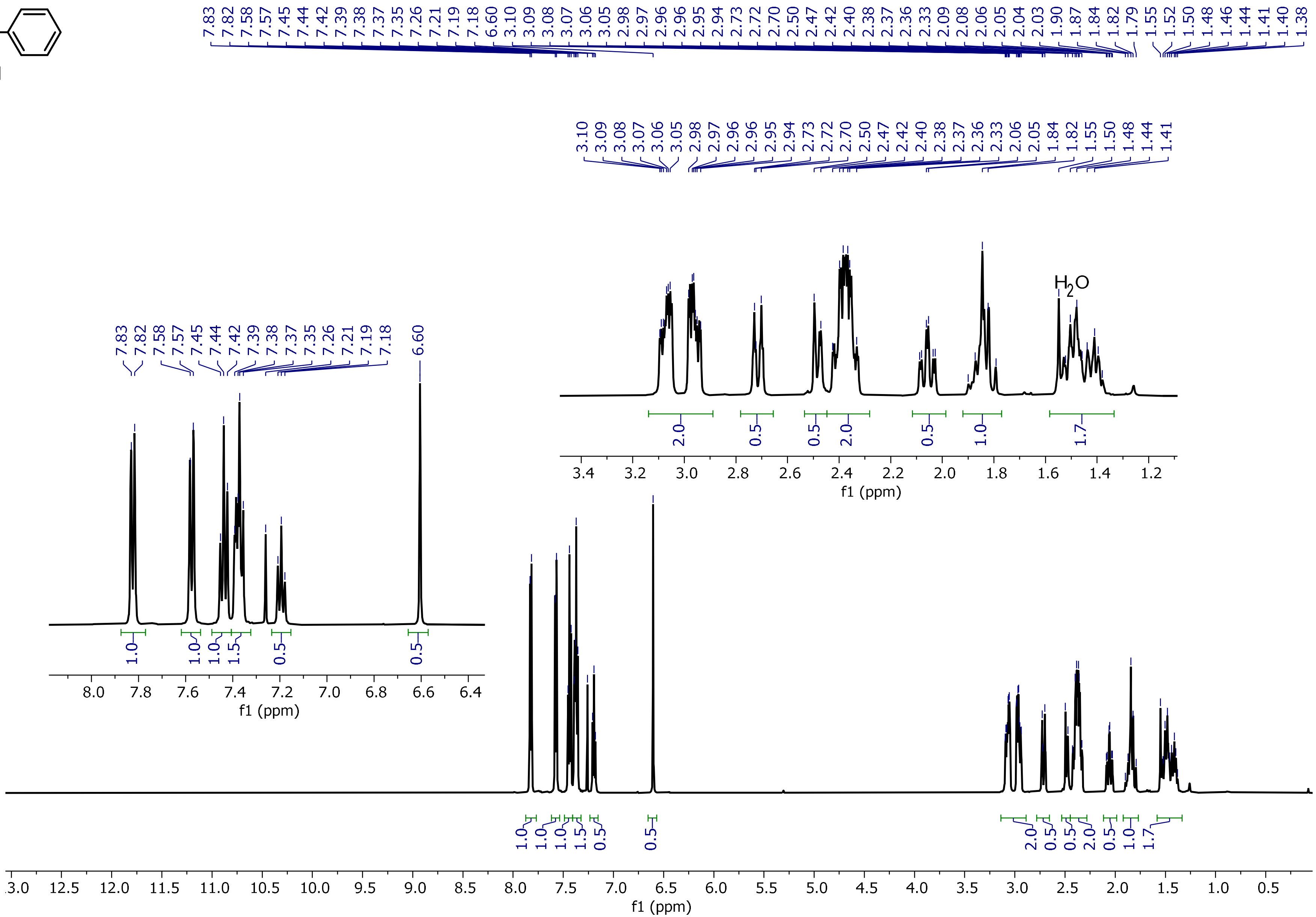

Figure S126. ^{13}C-APT NMR ($CDCl_3$, 125 MHz) spectrum **7c**

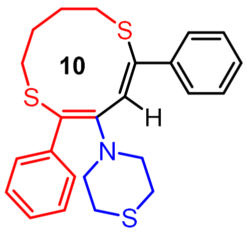

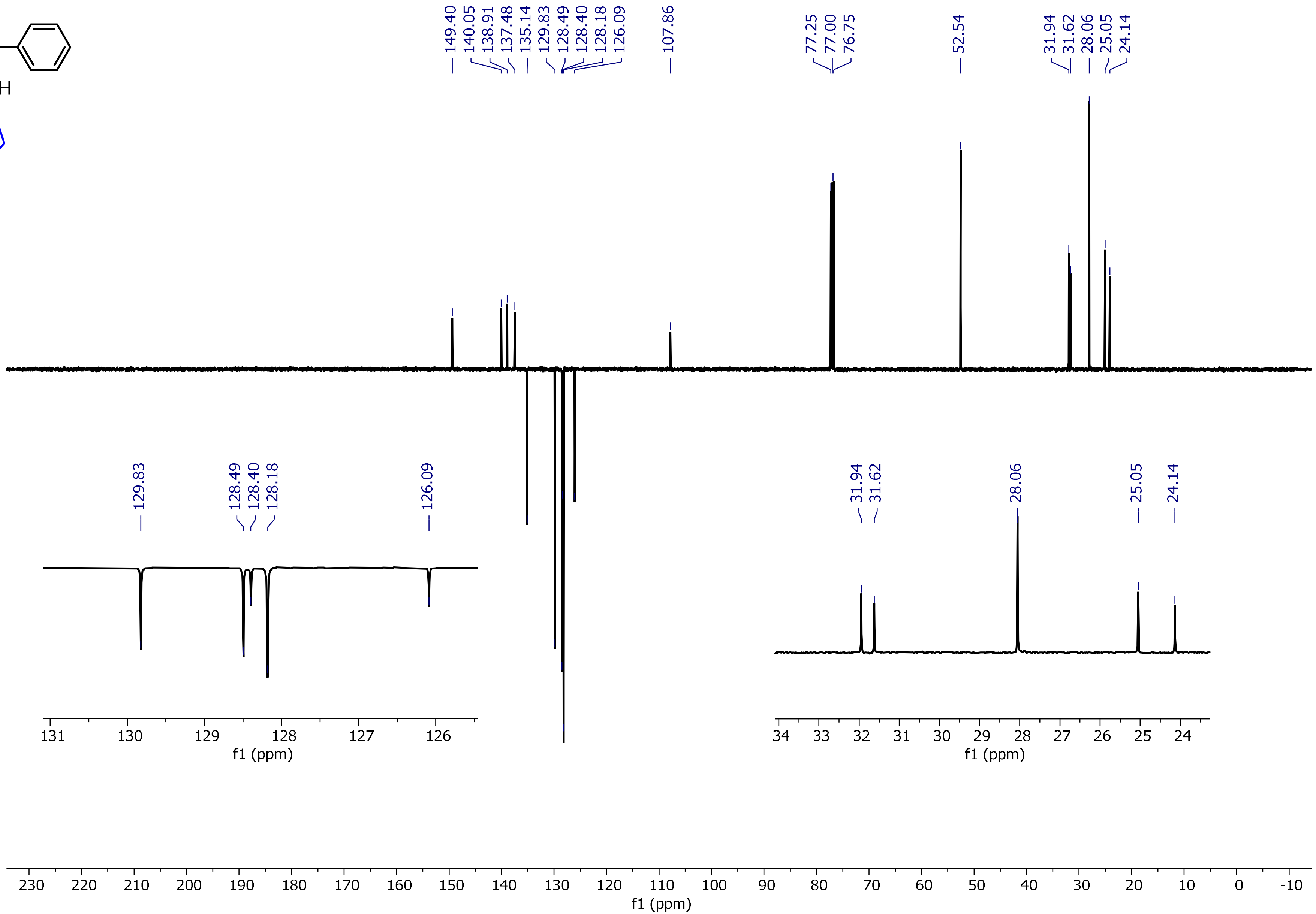

Figure S127. ^{1}H-NMR ($CDCl_3$, 500 MHz) spectrum **7d**

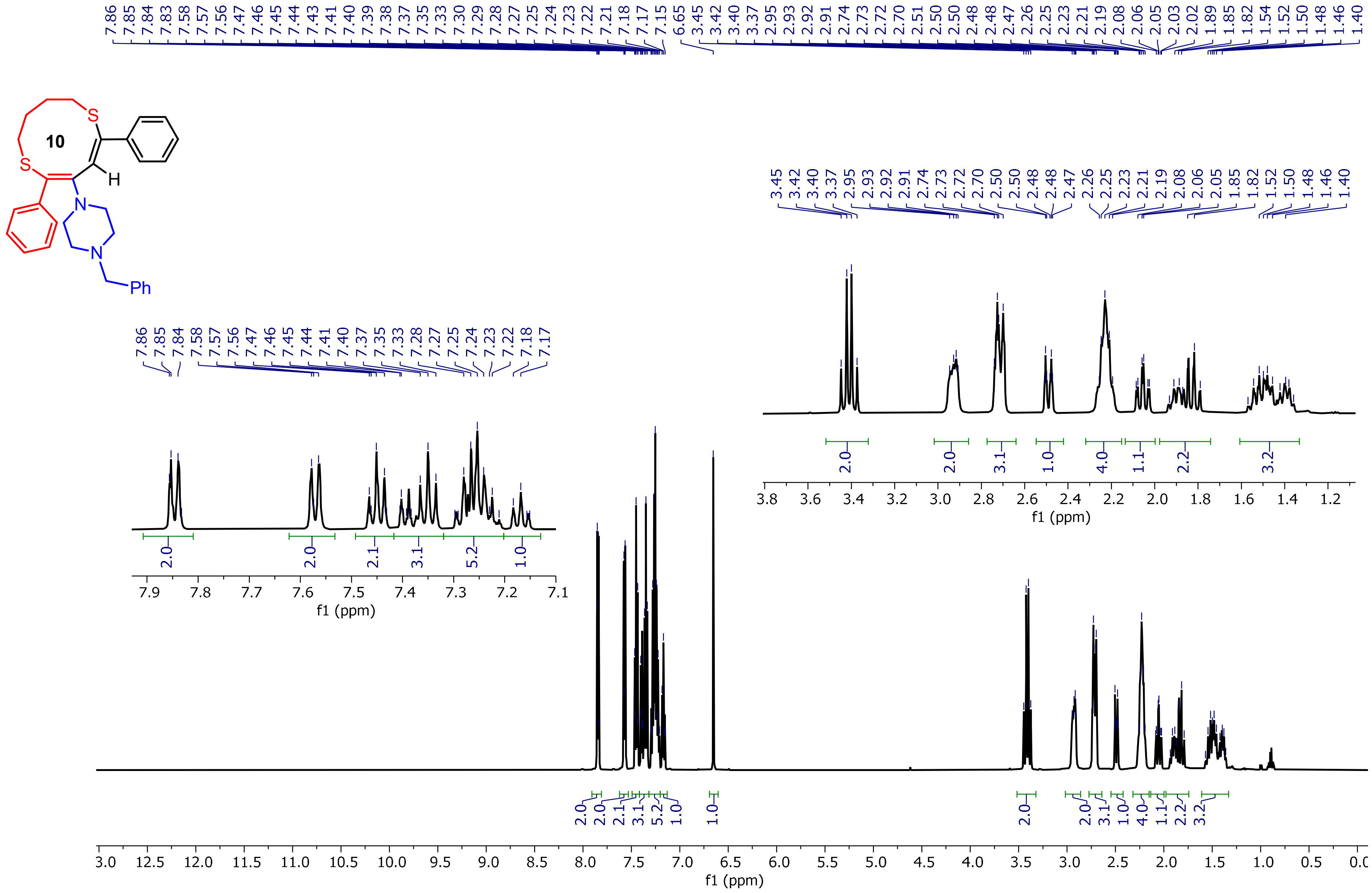

Figure S128. ^{13}C-NMR ($CDCl_3$, 125 MHz) spectrum **7d**

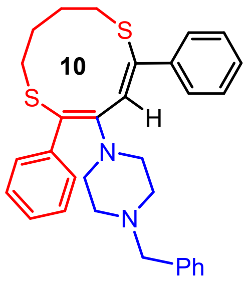

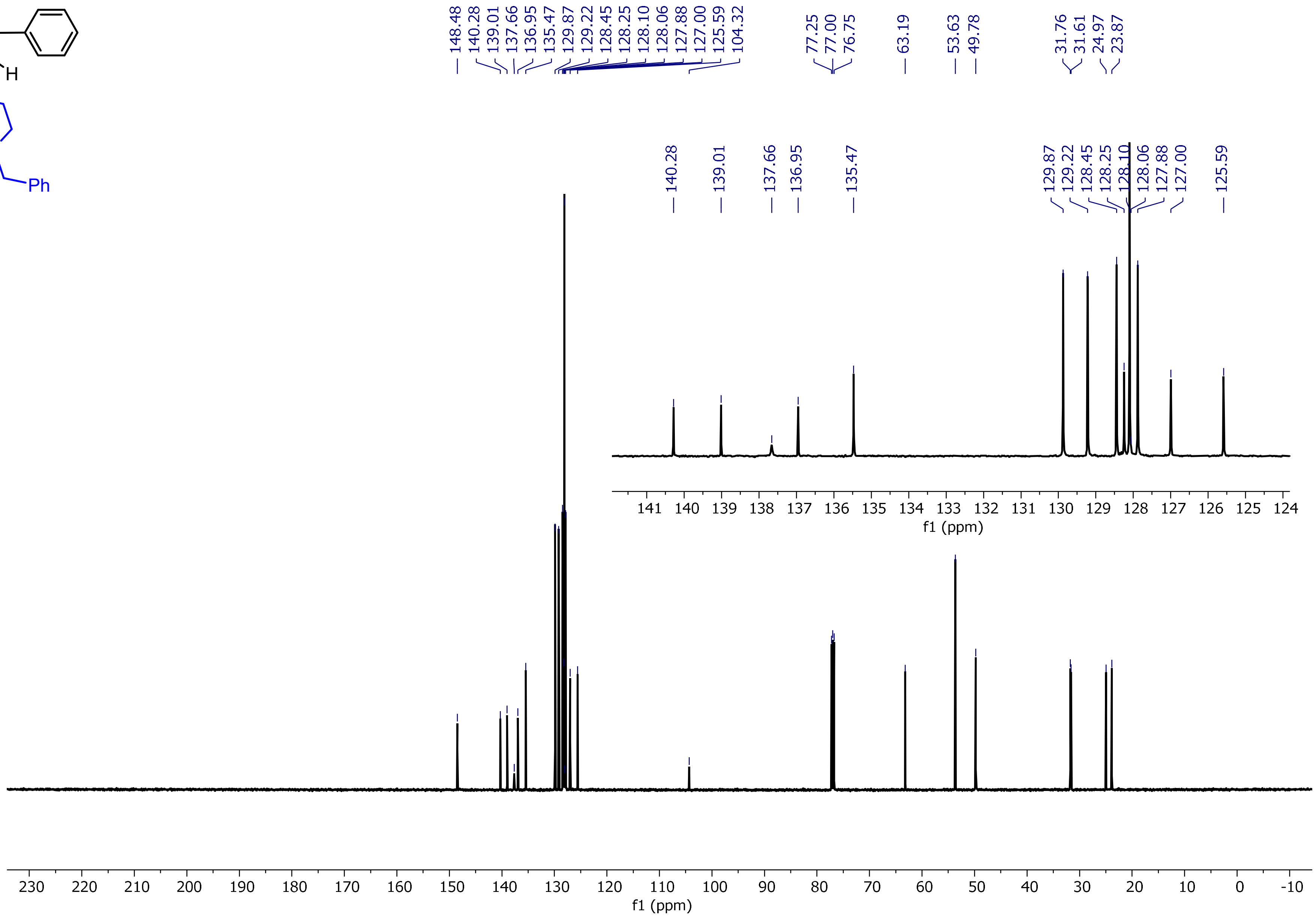

Figure S129. ^{13}C-APT NMR ($CDCl_3$, 125 MHz) spectrum **7d**

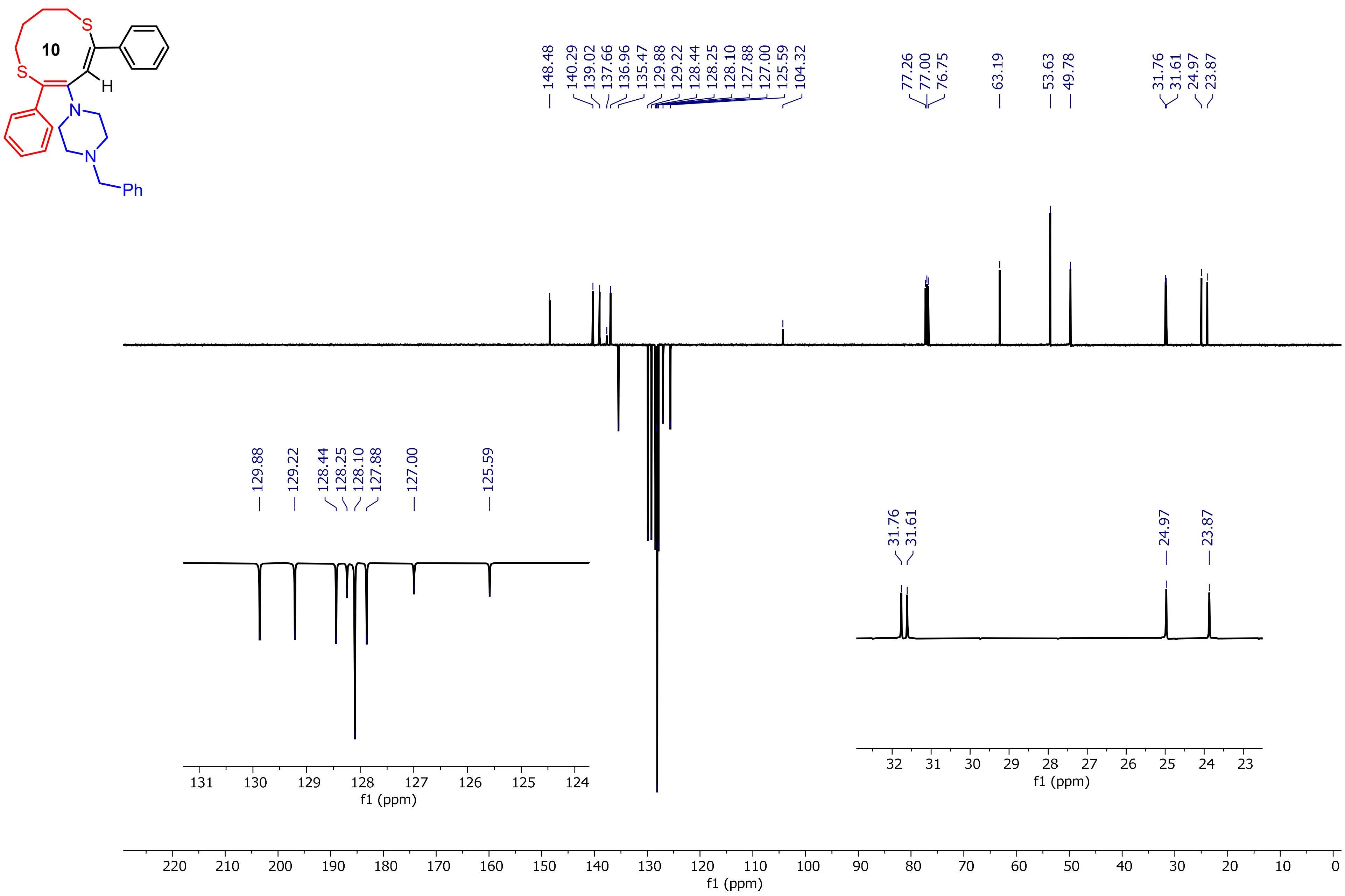

Supplement: Supplementary file 4 — ol3c01118_si_004.pdf [file ol3c01118_si_004.pdf]
